# Discovery of structurally diverse polyprenylated acylphloroglucinols with quorum sensing inhibitory activity from *Hypericum seniawinii* Maxim.

**DOI:** 10.1007/s13659-025-00520-z

**Published:** 2025-06-19

**Authors:** Yulin Duan, Xiaoxia Gu, Xincai Hao, Guosheng Cao, Weiguang Sun, Changxing Qi, Yonghui Zhang

**Affiliations:** 1 Hubei Key Laboratory of Natural Medicinal Chemistry and Resource Evaluation, School of Pharmacy, Tongji Medical College, Huazhong University of Science and Technology, Wuhan, 430030 Hubei People’s Republic of China; 2 Department of Pharmacy, Wuhan No.1 Hospital, Wuhan, 430022 People’s Republic of China; 3 Key Laboratory of Technology of Drug Preparation (Zhengzhou University), Ministry of Education of China; Key Laboratory of Henan Province for Drug Quality and Evaluation; Institute of Pharmaceutical Sciences, Zhengzhou University, Zhengzhou, 450001 China; 4 Hubei Key Laboratory of Wudang Local Chinese Medicine Research, Hubei Engineering Technology Center for Comprehensive Utilization of Medicinal Plants, College of Pharmacy, Hubei University of Medicine, Shiyan, 442000 People’s Republic of China; 5 College of Pharmacy, Hubei University of Chinese Medicine, Wuhan, 430065 People’s Republic of China; 6 Hubei Shizhen Laboratory, Wuhan, 430065 People’s Republic of China; 7 Key Laboratory of Traditional Chinese Medicine Resource and Compound PrescriptionMinistry of Education, Hubei University of Chinese Medicine, Wuhan, 430065 People’s Republic of China

**Keywords:** Polyprenylated Acylphloroglucinols, *Hypericum seniawinii* Maxim., Quorum sensing inhibitory, *Pseudomonas aeruginosa*

## Abstract

Four previously undescribed polyprenylated acylphloroglucinols, hyperisenins A–D (**1**–**4**), along with two known analogues (**5** and **6**), were obtained from the aerial part of *Hypericum seniawinii* Maxim. Compounds **1** and **2** were two highly degraded polyprenylated acylphloroglucinols with a cyclohexanone-monocyclic skeleton, while compound **3** was the first example of *O*-prenylated acylphloroglucinols with a 6/6/6 ring system. Their structures were identified by analyzing NMR, HRESIMS data, and quantum chemical calculations. The biosynthetic pathway of **1** and **2** might originate from bicyclic polyprenylated acylphloroglucinols via a series of complex retro-Claisen, keto − enol tautomerism, and intramolecular cyclization. The bioassay results showed that **4** exhibited quorum sensing inhibitory activity against *Pseudomonas aeruginosa*, which could decrease the activation of the *rhl* system, and significantly reduce rhamnolipid levels at a concentration of 100 µM, and the mechanism might be the ability to bind **4** to lasR and pqsR.

**Graphical Abstract:**

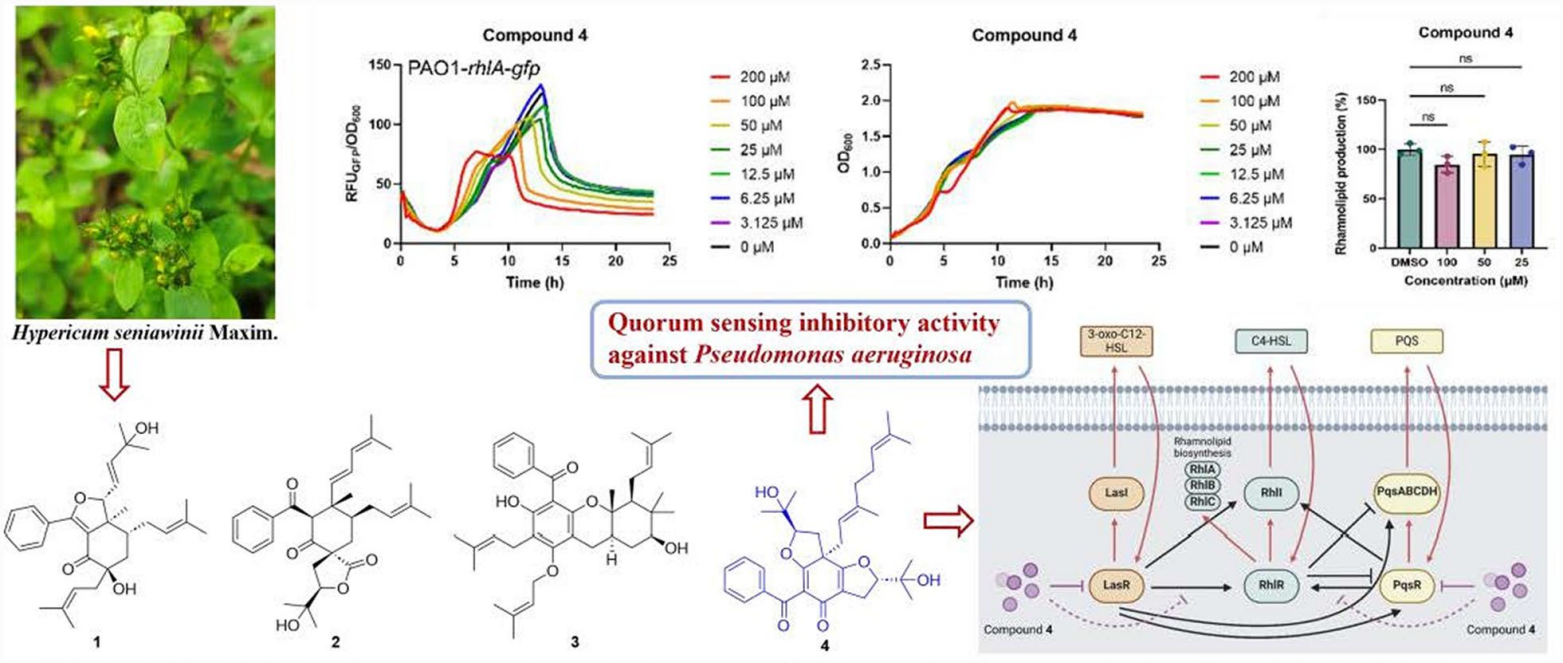

**Supplementary Information:**

The online version contains supplementary material available at 10.1007/s13659-025-00520-z.

## Introduction

The discovery of antibiotics has provided an effective way to control life-threatening infections [1]. However, the long-term excessive usage of antibiotics has led to antimicrobial resistance [2–4]. With at least 700,000 people dying from drug-resistant infectious diseases every year, and the figure is predicted to increase to 10 million deaths per year by 2050 without effective measures taken, there is an urgent required for novel treatment strategies to combat antimicrobial resistance limitation [3–6]. Biofilms mediated by quorum sensing (QS) are known to be one of the vital factors for drug resistance [7]. The QS system is the cell-to-cell communication that controls various collective behaviors, including virulence factor production, biofilm formation, and bioluminescence, resulting in forming a barrier to escape from the harsh environment to assist microbial pathogenesis [8–10]. Inhibiting this system will reduce bacterial virulence, prevent biofilm formation without affecting bacterial growth, and potentially slow the development of resistance [8, 11, 12]. QS inhibition was an alternative therapeutic strategy for antimicrobial therapy [13, 14].

**Fig. 1:**
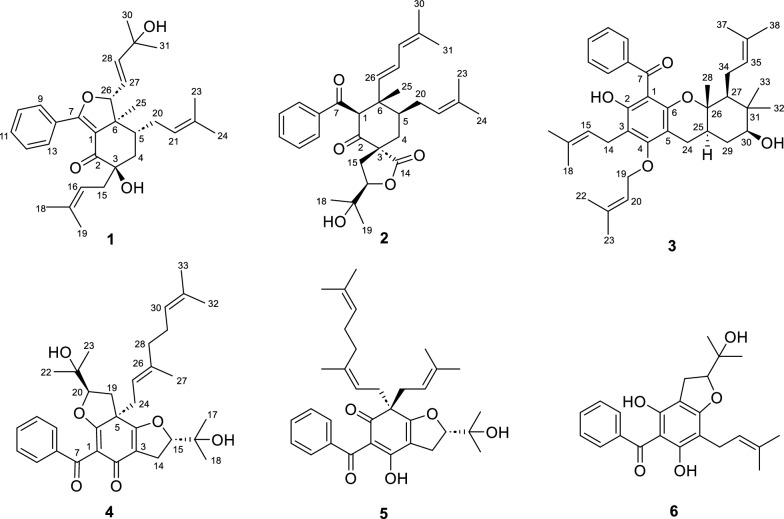
Chemical structures of compounds **1**–**6**

The secondary metabolites from plants, as a chemical defense, can resist the invasion of pathogenic bacteria in the environment and play a role in disease resistance, which are important sources for discovering novel antibiotic drugs [15–20]. Some have been identified as potential QS inhibitors, such as caffeine, hordenine, and iberin [21–25]. To discover more abundant scaffolds of QS inhibitors, we have focused on the plant *Hypericum seniawinii*, a perennial herb widely distributed in the southern region of China, which was a folk medicine used for detoxification, regulating menstruation, and activating blood [26, 27]. The chemical constituents of *H. seniawinii* lead to the isolation of four novel polyprenylated acylphloroglucinols, hyperisenins A–D (**1**–**4**), along with two known analogs (**5** and **6**) (Fig. 1). Compounds **1** and **2** were two highly degraded cyclohexanone-monocyclic polyprenylated acylphloroglucinols that might originate from bicyclic polyprenylated acylphloroglucinols (BPAPs) via a series of complex retro-Claisen, keto − enol tautomerism, and intramolecular cyclization, and compound **3** was a unique *O*-prenylated acylphloroglucinol with a 6/6/6 ring system. All isolates were assayed for QS inhibitory activity against *Pseudomonas aeruginosa*. The results showed that compound **4** could inhibit the QS system by decreasing the activation of the *rhl* system with no effects on the growth of *P. aeruginosa,* and reducing rhamnolipid levels by activating the *las* and *pqs* systems with molecular docking. Herein, the isolation, structural identification, plausible biogenetic pathways, and biological assay of the isolates were reported.

## Results and discussion

Hyperisenin A (**1**) was obtained as a colorless oil, and the molecular formula was defined as C_30_H_40_O_4_ based on its HRESIMS data (*m*/*z* [M + Na]^+^: 487.2802, calcd 487.2819), which possessed eleven degrees of unsaturation. The ^1^H NMR data showed five aromatic protons [*δ*_H_ 8.01 (2H, dd, *J* = 8.5, 1.4 Hz), 7.47 (1H, tt, *J* = 7.3, 1.4 Hz), 7.41 (2H, tt, *J* = 7.4, 1.4 Hz)], four olefinic protons [*δ*_H_ 6.15 (1H, d, *J* = 15.5 Hz), 5.98 (1H, dd, *J* = 15.5, 7.7 Hz), 5.15 (1H, m), 5.13 (1H, m)], and seven methyls [*δ*_H_ 1.74 (3H, s), 1.71 (3H, s), 1.66 (3H, s), 1.60 (3H, s), 1.38 (3H, s), 1.37 (3H, s), 1.07 (3H, s)] (Table 1). The ^13^C NMR spectrum showed 30 carbon signals (Table 1), which could be assigned to one ketone carbonyl (*δ*_C_ 196.0), one monosubstituted phenyl ring [*δ*_C_ 132.0, 130.5 (× 2), 129.4, 127.8 (× 2)], eight olefinic carbons (*δ*_C_ 166.8, 144.8, 135.6, 133.4, 122.3, 121.9, 119.0, 117.6), and three quaternary carbons including two oxygenated carbons (*δ*_C_ 76.3, 71.0), two methines, three methylenes and seven methyls. By analyzing the 1D NMR data and unsaturation, combined with the data reported in the literature [28–30], it is inferred that **1** was a polyprenylated acylphloroglucinol with a bicyclic system.

**Table 1:** The ^1^H and ^13^C NMR data of compounds **1**‒**4** (*δ* in ppm, *J* in Hz)

*No*	**1**^*a*^		**2**^*b*^		**3**^*b*^		**4**^*c*^	
	*δ*_H_	*δ*_C_	*δ*_H_	*δ*_C_	*δ*_H_	*δ*_C_	*δ*_H_	*δ*_C_
1		117.6	5.48 s	63.4		111.2		114.7
2		196.0		202.1		158.8		182.3
3		76.3		61.1		116.0		113.3
4	1.97 dd (15.0, 3.5)	36.3	a 2.46 dd (14.4, 4.4)	40.2		161.4		170.0
	1.73^*d*^		b 1.73^*d*^					
5	2.08 m	41.4	2.67 m	44.8		109.2		50.5
6		50.9		50.5		153.4		175.7
7		166.8		197.2		200.8		193.9
8		129.4		139.8		142.0		138.0
9	8.01 dd (8.5, 1.4)	130.5	7.69 dd (8.5, 1.4)	128.7	7.57 dd (8.2, 1.1)	130.0	7.85 dd (8.5, 1.3)	129.6
10	7.41 tt (7.4, 1.4)	127.8	7.44 tt (7.8, 1.7)	129.8	7.37 t (7.8)	129.3	7.54 tt (7.4, 1.4)	128.5
11	7.47 tt (7.3, 1.4)	132.0	7.55 tt (7.4, 1.3)	134.3	7.52 tt (7.5, 1.3)	133.2	7.42 t (7.7)	133.5
12	7.41 tt (7.4, 1.4)	127.8	7.44 tt (7.8, 1.7)	129.8	7.37 t (7.8)	129.3	7.54 tt (7.4, 1.4)	128.5
13	8.01 dd (8.5, 1.4)	130.5	7.69 dd (8.5, 1.4)	128.7	7.57 dd (8.2, 1.1)	130.0	7.85 dd (8.5, 1.3)	129.6
14				176.7	3.35 m	23.8	a 2.75 dd (14.9, 8.6)	28.0
					3.31^*d*^		b 2.89 dd (15.1, 10.7)	
15	2.49 dd (13.9, 6.8)	39.3	a 2.91 dd (13.0, 7.0)	32.5	5.22 m	124.8	4.70 dd (10.7, 8.6)	93.0
	2.41 dd (13.9, 8.4)		b 1.95 dd (13.1, 9.3)					
16	5.15 m	119.0	4.24 dd (9.3, 7.0)	86.0		131.8		72.2
17		135.6		70.9	1.76 s	18.1	1.22 s	23.8
18	1.66 s	18.3	1.28 s	26.5	1.68 s	25.9	1.28 s	25.5
19	1.74 s	26.0	1.14 s	24.8	4.47 dd (11.4, 7.0),	71.1	a 2.23 dd (12.1, 5.8)	31.6
					4.38 dd (11.4, 7.2)		b 2.32 dd (12.1, 9.3)	
20	2.14 m	28.3	2.15 m	29.4	5.56 m	121.6	4.61 dd (9.3, 5.7)	90.8
	1.69^*d*^		1.69^*d*^					
21	5.13 m	122.3	5.14 m	123.9		138.9		71.2
22		133.4		134.1	1.70 s	18.2	1.09 s	23.4
23	1.60 s	18.1	1.59 s	18.0	1.81 s	26.0	1.28 s	27.6
24	1.71 s	26.2	1.70 s	26.0	2.64 dd (16.1, 4.7),	25.1	2.71 dd (13.8, 8.4)	36.3
					2.31 dd (16.2, 12.8)		2.59 dd (13.8, 7.4)	
25	1.07 s	16.2	1.30 s	13.9	1.58^*d*^	40.7	5.12 m	116.7
26	4.86 d (7.7)	94.0	5.46 d (15.4)	137.7		82.3		141.4
27	5.98 dd (15.5, 7.7)	121.9	6.25 dd (15.4, 10.7)	127.9	1.15 dd (8.1, 1.9)	56.4	1.61 s	16.4
28	6.15 d (15.5)	144.8	5.64 d (10.6)	125.7	0.96 s	14.4	2.07 m	40.1
29		71.0		135.9	1.77^*d*^, 1.38 m	35.2	2.08 m	26.8
30	1.37 s	29.7	1.66 s	18.3	3.27 dd (11.7, 4.3)	78.3	5.08 m	123.8
31	1.38 s	30.0	1.68 s	26.0		41.9		132.1
32					0.76 s	16.1	1.60 s	17.9
33					0.81 s	28.8	1.68 s	25.9
34					1.70^*d*^, 0.90 m	24.1		
35					4.52 m	129.9		
36						128.0		
37					1.48 s	18.1		
38					1.59 s	25.8		

^*a*^^1^H NMR 400 MHz,^13^C NMR 100 MHz, in CDCl_3_

^*b*^^1^H NMR 600 MHz,^13^C NMR 150 MHz, in CD_3_OD

^*c*^^1^H NMR 600 MHz,^13^C NMR 150 MHz, in CDCl_3_

^*d*^Overlapped signal

**Fig. 2:**
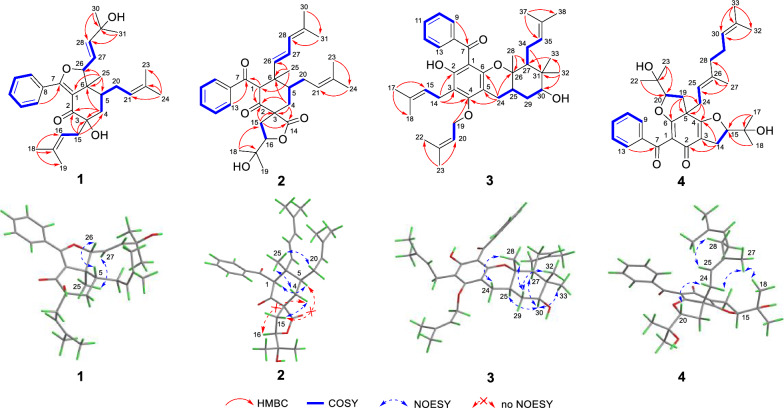
Key 2D NMR correlations of compounds **1**–**4**

Its gross structure was confirmed by analyzing its 1D and 2D NMR data (Fig. 2). The HMBC correlations from H_2_-4 to C-2, C-3, and C-6, from H_3_-25 to C-1, C-5, and C-6, from H-13 to C-7, the ^1^H-^1^H COSY cross-peaks of H_2_-4/H-5 and H-9/H-10/H-11/H-12/H-13, combined with chemical shifts of C-1 (*δ*c 117.6) and C-7 (*δ*c 166.8), indicated that the cyclohexanone system existed, and a benzoyl group converted into enol form was attached at C-1. Then, the HMBC correlations from H_2_-15 to C-2, C-3, and C-4, from H_3_-18 to C-16, C-17, and C-19, from H_3_-24 to C-21, C-22, and C-23, from H_3_-25 to C-6, from H_3_-30 to C-28 and C-29, combined with the ^1^H-^1^H COSY cross-peaks of H_2_-15/H-16, H-5/H_2_-20/H-21, and H-26/H-27/H-28, revealed the existence of three side chains at C-3, C-5, and C-6. Further analysis of the downfield chemical shifts of C-3 (*δ*c 76.3) and C-26 (*δ*c 94.0) indicated that hydroxyl groups were attached to C-3 and C-26, respectively. The above-mentioned groups accounted for ten degrees of unsaturation. The remaining degree of unsaturation was attributed to a furan ring formed between C-7 and C-26, which was consistent with both the downfield chemical shift of C-26 (*δ*c 94.0) and the HRESIMS data. Accordingly, the planar structure, featuring a cyclohexanone-monocyclic skeleton, was confirmed.

**Fig. 3:**
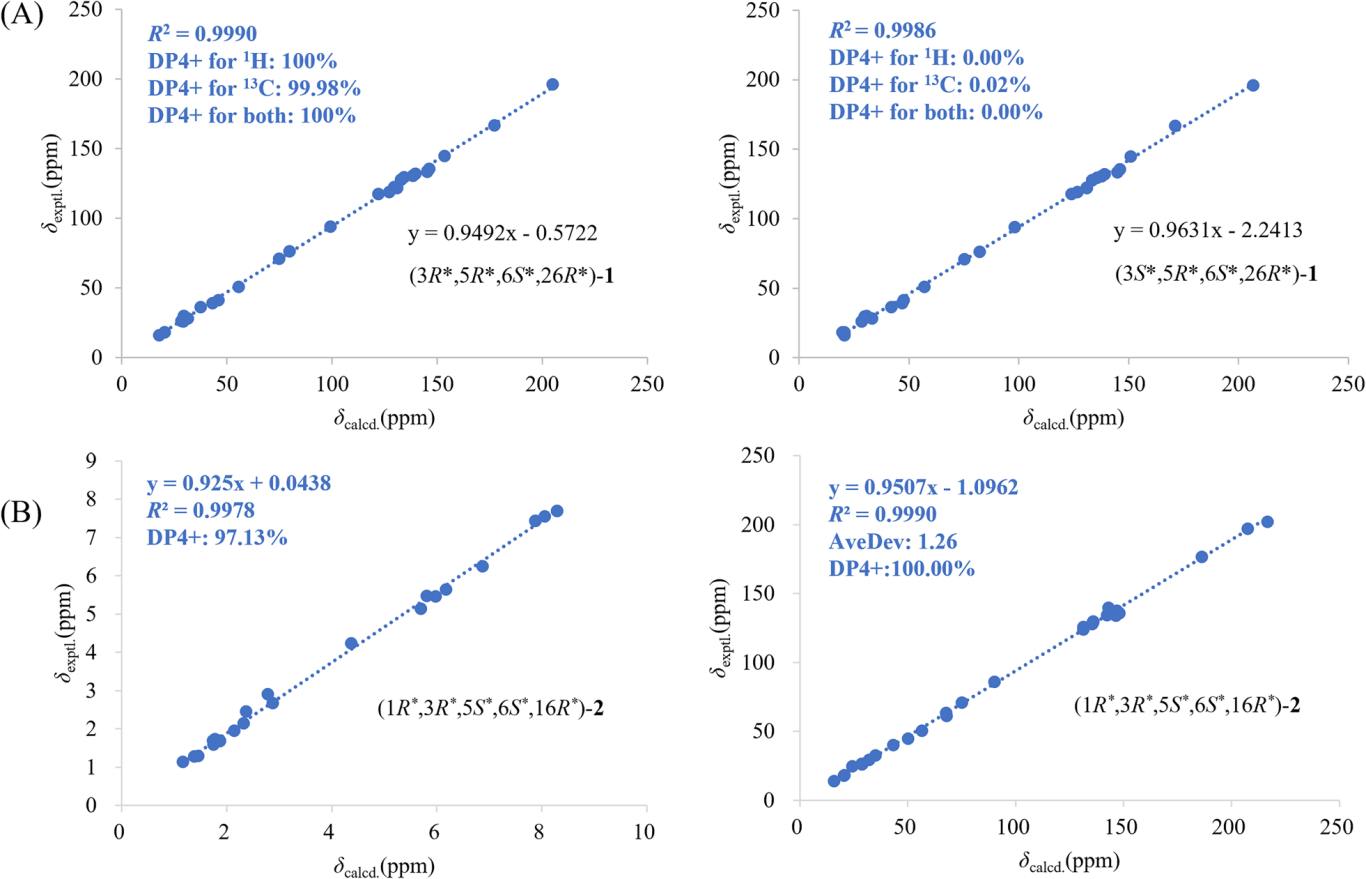
(A) NMR calculations with a DP4+ probability analysis: (3R*,5R*,6S*,26R*)**-1** and (3S*,5R*,6S*,26R*)**-1**. (B) Linear correlation between the experimental and calculated ^1^H (left) and ^13^C NMR (right) chemical shifts, and the results of a DP4+ probability analysis for (1R^*^,3R^*^,5S^*^,6S^*^,16R^*^)-**2**

**Fig. 4:**
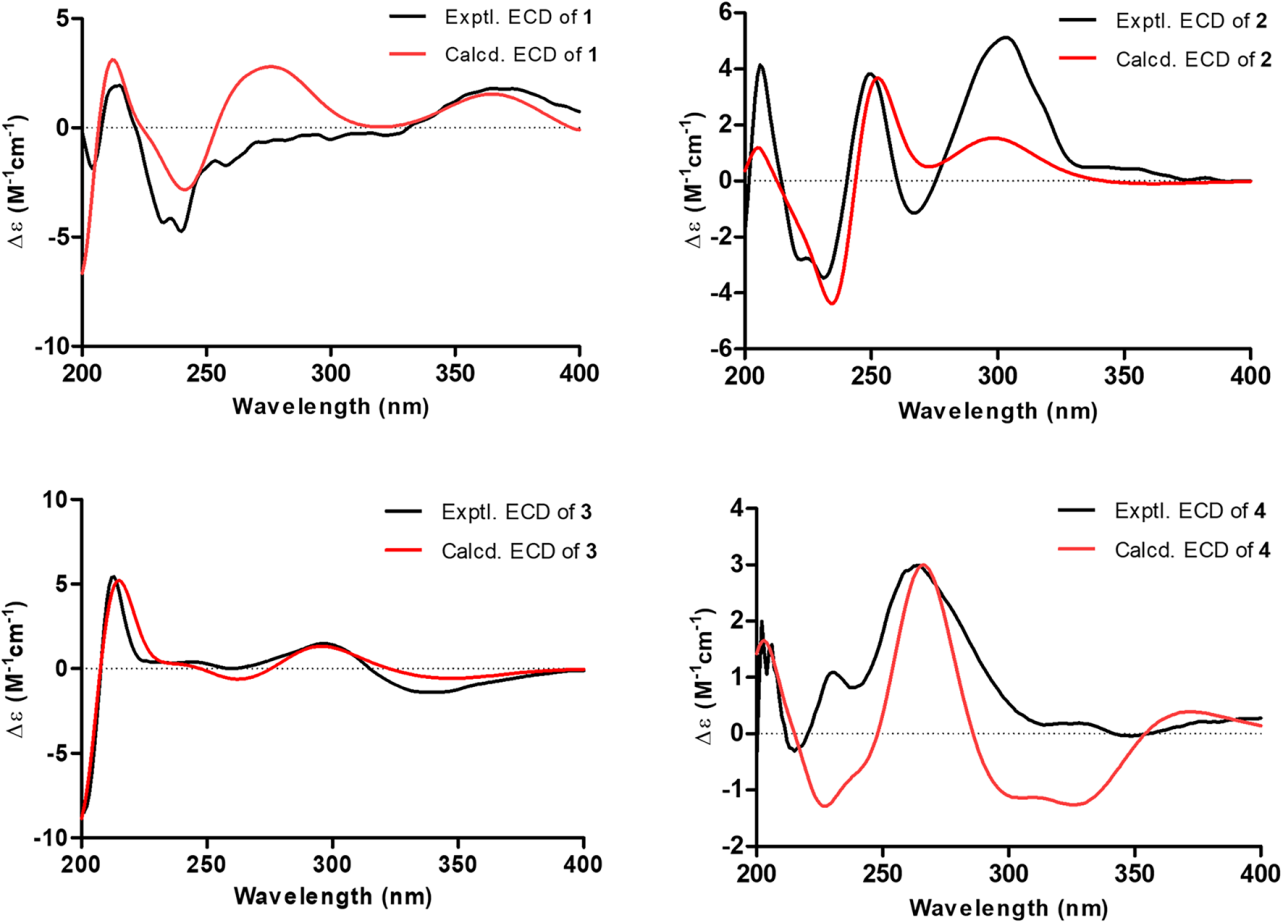
Experimental and calculated ECD spectra of compounds **1–4**

The relative configurations of C-5, C-6, and C-26 in **1** were assigned by the NOESY spectrum, in which the cross-peaks of H-5/H-26 and H_3_-25/H-27 indicated that H_3_-25 was *α*-oriented, while H-5 and H-26 were on the same side with the *β*-orientations. The large coupling constant between H-27 and H-28 (*J* = 15.5 Hz) confirmed the* E* configuration. In order to determine the relative configuration of C-3, the calculated ^13^C NMR data with DP4+ analysis of two configurations (3R*,5R*,6S*,26R*)-**1** and (3S*,5R*,6S*,26R*)-**1** were applied at the mPW1PW91/6–311 + G** level. The results showed that (3R*,5R*,6S*,26R*)-**1** had a better linear correlation with 100% DP4+ probability (R^2^ = 0.9990) (Fig. 3A), suggesting that the relative configuration of **1** was defined as 3R*,5R*,6S*,26R*. The absolute configuration of **1** was identified by ECD calculation at the PBE0/def2-TZVP level, the result showed that the calculated ECD (3*R*,5*R*,6*S*,26*R*)-**1** curve matched well with the experimental one, allowing to assign its absolute configuration as 3*R*,5*R*,6*S*,26*R* (Fig. 4). Thus, the structure of **1** was elucidated.

Hyperisenin B (**2**), a colorless oil, possesses the molecular formula C_31_H_40_O_5_ on the basis of the HRESIMS data (*m*/*z* [M + Na]^+^: 515.2753, calcd 515.2768). Its ^1^H and ^13^C NMR spectra showed the presence of a monosubstituted benzene ring, two ketone carbonyls, one ester carbonyl, six olefin carbons, and seven methyl groups, which were similar to those of spirohypolactone B [31]. Further comparison of NMR data between **2** and spirohypolactone B revealed that they possessed the same skeleton, except for the acyl substituent and the number of the double bond [31]. The presence of a phenyl group at C-7 was verified by the ^1^H-^1^H COSY cross-peaks of H-9/H-10/H-11/H-12/H-13 and the HMBC cross-peak from H-13 to C-7, which was consistent with its 1D NMR data (Fig. 2). Furthermore, the 4-methylpenta-1,3-diene group was attached at C-6, deduced from the HMBC correlations from H_3_-25 to C-6 and C-26, from H_3_-30 to C-31, from H_3_-31 to C-28, and the ^1^H-^1^H COSY cross-peaks of H-26/H-27/H-28. The relative configurations was the same as that of spirohypolactone B and norhyperpalum H assigned as 1R*,3R*,5S*,6S*,16R* via the similar the NOESY cross-peaks of H-1/H-5, H_2_-20/H_3_-25, H_2_-4a/H_2_-15b, and H_2_-4b/H_3_-25, and along with the crucial absence of the NOESY cross-peaks of H_2_-4/H-16 and H-5/H_2_-15 (Fig. 2). To further confirm the relative configurations of C-3 and C-16, a DP4+ probability analysis of four isomers [A: (3R*,16R*), B: (3R*,16S*), C: (3S*,16R*), D: (3S*,16S*)] was conducted, and the calculated data of (1R*,3R*,5S*,6S*,16R*)-**2** has good linear correlations with the experimental data with 100% DP4+ probability (all data) (Fig. 3B and S40). This deduction was further supported by a critical 1D NMR comparison in the same solvent of **2** with similar compounds, spirohypolactones A and B, and norhyperpalum H [31, 32] (Figure S41). The Δ^26(27)^ double bond was assigned as *E* configuration based on the coupling constant (*J*_H-26, H-27_ = 15.4 Hz). Finally, the ECD calculation of **2** was conducted at the PBE0/def2-TZVP level, and its absolute configuration was determined to be 1*R*,3*R*,5*S*,6*S*,16*R* (Fig. 4).

Hyperisenin C (**3**), obtained as a yellow oil, had the molecular formula C_38_H_50_O_5_ based on its HRESIMS data (*m/z* [M + Na]^+^: 609.3572, calcd 609.3550), suggesting fourteen indices of hydrogen deficiency. Its ^1^H NMR spectrum showed the existence of five characteristic protons of the benzene ring [*δ*_H_ 7.57 (2H, dd, *J* = 8.2, 1.1 Hz), 7.52 (1H, tt,* J* = 7.5, 1.3 Hz), 7.37 (2H, t, *J* = 7.8 Hz)], three olefinic protons [*δ*_H_ 5.56, (1H, m), 5.22 (1H, m), 4.52 (1H, m)], and nine methyl groups [*δ*_H_ 1.81 (3H, s), 1.76 (3H, s), 1.70 (3H, s), 1.68 (3H, s), 1.59 (3H, s), 1.48 (3H, s), 0.96 (3H, s), 0.81 (3H, s), 0.76 (3H, s)] (Table 1). Its ^13^C NMR and DEPT data indicated 38 carbons (Table 1). The 1D NMR spectra characteristics of the above analysis indicated that **3** belonged to a polyprenylated acylphloroglucinol derivative.

Detailed analysis of its HSQC, HMBC, and ^1^H–^1^H COSY spectra indicated that **3** had the same skeleton as that of madeleinol A [33], and the main differences were acyl side chain and isopentenyl side chain (Fig. 2). The HMBC correlations from H_2_-14 to C-2, C-3, and C-4, from H_3_-17 to C-15 and C-18, from H_3_-32 to C-30, C-31, and C-33, from H_3_-33 to C-27, from H_3_-37 to C-35 and C-38, combined with the ^1^H–^1^H COSY correlations of H-27/H_2_-34/H-35 and H_2_-14/H-15, suggested the location of the *gem*-dimethyl group, and two isoprenyl groups located at C-3 and C-27, respectively. Furthermore, the *O*-isoprenyl side chain was attached at C-4, which was deduced from the HMBC correlations from H_2_-19 to C-4, from H_3_-23 to C-20 and C-22, along with the ^1^H–^1^H COSY cross-peaks of H_2_-19/H-20, and the downfield chemical shift of C-19 (*δ*_C_ 71.1). In addition, the benzene ring was attached at C-7 by analysis of its 1D NMR data and the HMBC correlation from H-9 to C-7, and the ^1^H–^1^H COSY cross-peaks of H-9/10/H-11/H-12/H-13. In the NOESY spectrum (Fig. 2), the cross-peaks of H-25/H-30, H-27/H-30, and H-30/H_3_-33 indicated that these groups were cofacial, assigned as *α*-orientations, while H-24*β*/H_3_-28, H_3_-28/H-29*β*, and H-29*β*/H_3_-32 clarified that they were *β*-oriented. The calculated ECD method was applied to determine its absolute configuration as 25*R*,26*S*,27*S*,30*S* (Fig. 4). Thus, a unique *O*-prenylated acylphloroglucinol with a 6/6/6 ring system was established.

Hyperisenin D (**4**) was also obtained as a yellow oil and had the molecular formula C_33_H_42_O_6_ based on its HRESIMS at *m/z* 557.2876 [M + Na]^+^ (calcd 557.2874), which possessed thirteen degrees of unsaturation. The 1D NMR and HSQC spectra revealed 33 carbons (Table 1), including a benzoyl group [*δ*c 193.9, 138.0, 133.5, 129.6 (× 2), 128.5 (× 2)], a geranyl group (*δ*c 141.4, 132.1, 123.8, 116.7, 40.1, 36.3, 26.8, 25.9, 17.9, 16.4), two oxidated quaternary carbon (*δ*c 72.2, 71.2), two methines (*δ*c 93.0, 90.8), two methylenes (*δ*c 31.6, 28.0), and four methyl groups (*δ*c 27.6, 25.5, 23.8, 23.4), and the remaining six carbons were characteristic of a dearomatized phloroglucinol core including an enolic 1,3-diketone moiety (*δ*c 182.3, 175.7, 114.7), one oxygen-bearing ene (*δ*c 170.0, 113.3), and one quaternary carbon (*δ*c 50.5). The mentioned groups occupied eleven degrees of unsaturation. Two additional rings should be formed in the structure of **4**. Thus, the aforementioned evidence suggested that **4** should be a tricyclic dearomatized prenylated acylphloroglucinol derivative.

The planar structure was confirmed by analyzing its HSQC, HMBC, and ^1^H-^1^H COSY spectra (Fig. 2), similar to that of hypermonin C (**5**) [34], and the main difference was the isoprenyl side chain at C-5. The HMBC correlations from H_2_-19 to C-5, C-6, and C-24, from H_3_-22 to C-20, from H_3_-23 to C-20 and C-21, combined with the ^1^H-^1^H COSY cross-peaks of H_2_-19/H-20, indicated that the oxidized isopentenyl side chain was located at C-5. Furthermore, the downfield chemical shift of C-20 (*δ*_C_ 90.8), combined with degrees of unsaturation, indicated that C-6 and C-20 were connected via an oxygen atom to form a furan ring. In the NOESY spectrum (Fig. 2), the cross-peaks of H_3_-18/H_3_-27 and H-20/H_2_-24 indicated that H-20 and the geranyl group at C-5 were on the same side, assigned as *β*-orientations, while H-15 was on the opposite side with *α*-orientation. Then, the NOESY cross-peaks of H-25/H_2_-28 revealed the *E*-configuration of C-25/C-26 double bond. Thus, the relative configuration of **4** was determined to be 5S*,15S*,20R*. Finally, the absolute configuration of **4** was defined as 5*S*,15*S*,20*R* by ECD calculation (Fig. 4).

Two known compounds, hypermonin C (**5**) [34] and vismiaguianone B (**6**) [35], were obtained from this plant. Their structures were confirmed by comparing the 1D NMR data with those of the literature.

**Fig. 5:**
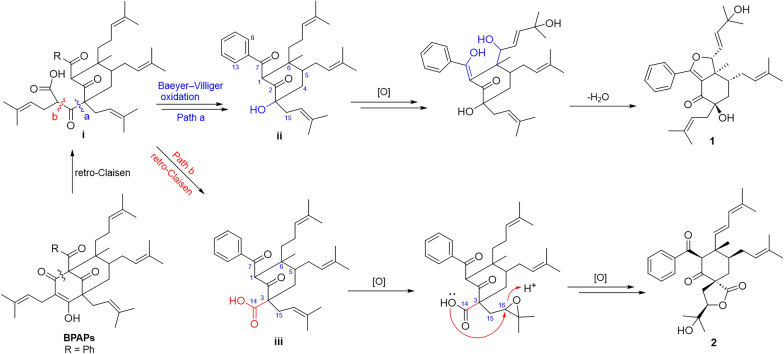
Plausible biogenetic pathway of compounds **1** and **2**

Hyperisenins A (**1**) and B (**2**), possessing a unique cyclohexanone-monocyclic system, were proposed to biogenetically originate from BPAPs (Fig. 5) [28, 36, 37]. It underwent a retro-Claisen reaction to yield the crucial intermediate **i**, followed by two distinct pathways to form intermediates **ii** and **iii**. Subsequently, compound **1** was constructed from **ii** via oxidation, keto − enol tautomerism, and intramolecular cyclization. On the other hand, **iii** underwent oxidation and intramolecular cyclization to obtain compound **2**.

**Fig. 6:**
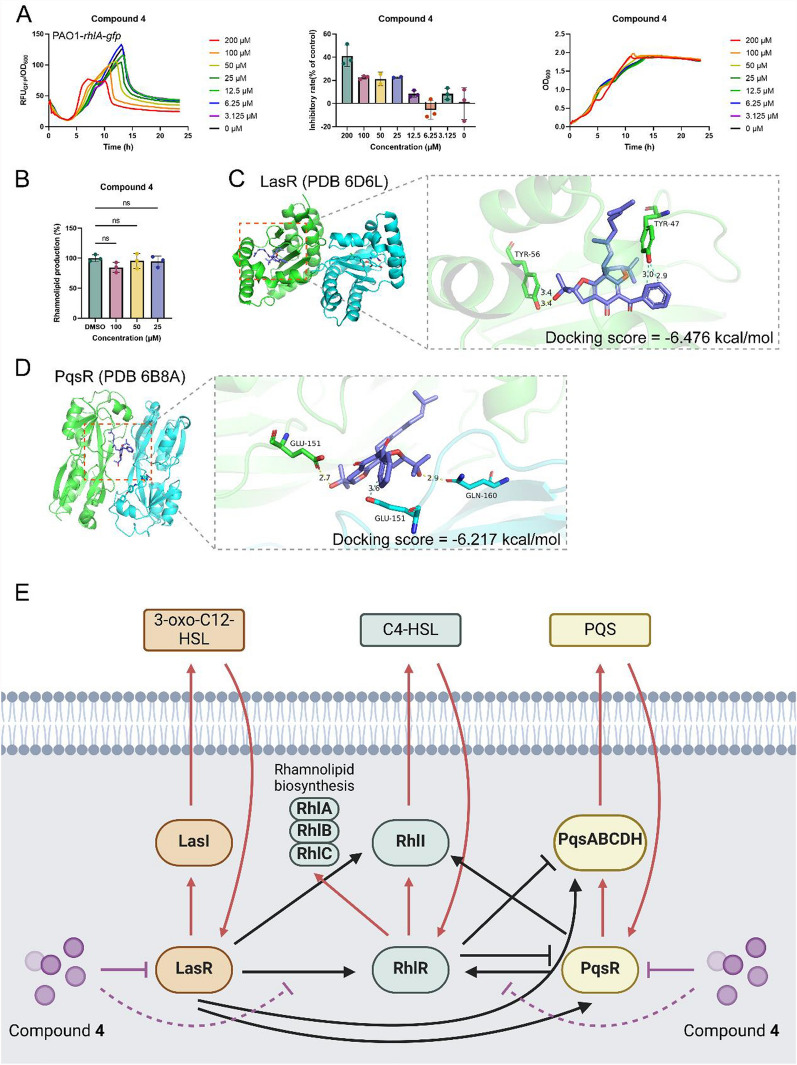
Compound **4** as a quorum sensing inhibitor against *P. aeruginosa*. **A** Compound** 4** inhibited the activation of the *rhl* pathway without affecting bacterial growth. **B** Compound **4** reduced the expression of the virulence factor rhamnolipid in CRPA. **C** Docking results of compound **4** with lasR (PDB 6D6L, colored by chain), depicted as slate sticks. **D** Docking results of compound **4** with pqsR (PDB 6B8A, colored by chain), shown as slate sticks. **E** Proposed mechanism of inhibition by compound **4** against rhamnolipid production, potentially through competitive inhibition of the lasR and pqsR receptors, both of which enhance the activation of the *rhl* system and rhamnolipid production. Data are presented as mean ± SD (*n* = 3). Significance levels are indicated as *p ≤ 0.05, **p ≤ 0.01, ***p ≤ 0.001, ****p ≤ 0.0001

Considering QS is the vital target for antimicrobial therapy [13, 14] and *Pseudomonas aeruginosa* is an opportunistic pathogen that is typically resistant to multiple clinically available antibiotics [38], compounds **1**–**6** were evaluated for the QS inhibitory activity against *P. aeruginosa* (Figure S46). The results showed that compound **4** was a potential QS inhibitor that decreased the activation of the *rhl* system, as evidenced by the reduced fluorescence density of the reporter strain PAO1-*rhlA*-*gfp* (Fig. 6A). As expected, compound **4** did not affect the growth of *P. aeruginosa*, consistent with its role as a QS inhibitor. We further examined the production of rhamnolipids, a virulence factor regulated by the *rhl* system, in a clinically isolated carbapenem-resistant *P. aeruginosa* (CRPA) strain. Compound **4** significantly reduced rhamnolipid levels at a concentration of 100 µM (Fig. 6B). *P. aeruginosa*'s QS system includes two other well-defined pathways apart from the *rhl* system: the *las* and *pqs* systems [8]. Due to the limited yield of compound **4**, its potential mechanism was explored through molecular docking with three QS receptors (lasR, rhlR, and pqsR).

Unexpectedly, compound **4** failed to dock with rhlR but successfully engaged with lasR and pqsR, demonstrating significant affinity within their ligand-binding pockets. For lasR, it is proposed that compound **4** forms two aromatic hydrogen bonds with Tyr47 and a combination of a hydrogen bond and an aromatic bond with Tyr56, yielding a docking score of -6.476 kcal/mol (Fig. 6C). Regarding pqsR, compound **4** appears to bind to the ligand-binding site, albeit slightly shifted towards the dimer interface, forming a hydrogen bond with Glu151 in chain A and both a hydrogen bond and an aromatic bond with Glu151 in chain B, resulting in a docking score of -6.217 kcal/mol (Fig. 6D). Given that both the *las* and *pqs* systems enhance the activation of the *rhl* system [8], we hypothesize that the binding of compound **4** to lasR and pqsR may competitively inhibit their contribution to the *rhl* system, thereby regulating rhamnolipid production (Fig. 6E).

In summary, the phytochemical investigation of the dried aerial parts of *H. seniawinii* Maxim. resulted in the isolation of four undescribed polyprenylated acylphloroglucinols (**1**–**4**), as well as two known analogs (**5** and **6**). All isolates were obtained from this plant for the first time. Compounds **1** and **2** were two degraded polyprenylated acylphloroglucinols bearing the unique cyclohexanone-monocyclic system, and their plausible biosynthetic pathway was proposed. Furthermore, compound **4** was a potential QS inhibitor that decreased the activation of the *rhl* system and reduced rhamnolipid levels. Its mechanism might be the ability to bind between **4** lasR and pqsR. Our findings might provide a potential candidate as QS inhibitors to treat infectious diseases for further research.

## Experimental section

### General experimental procedures

Optical rotations were recorded with a PerkinElmer 341 polarimeter (PerkinElmer Inc., Fremont, California, USA). UV spectra were obtained in CH_3_OH using a Lambda 35 instrument (PerkinElmer Inc., Fremont, California, USA). ECD spectra in CH_3_OH were detected on a JASCO-810 spectrometer (JASCO, Tokyo, Japan). A Bruker Vertex 70 FT-IR spectrophotometer (Bruker, Karlsruhe, Germany) was used to acquire IR spectra. 1D and 2D NMR spectra were collected using Bruker AM-400 and AV-600 spectrometers. HRESIMS data were measured with a Bruker micOTOF II and SolariX 7.0 spectrometer (Bruker, Karlsruhe, Germany). Analytical HPLC was performed on a Dionex HPLC system with a DAD detector, and semipreparative HPLC was performed on an Agilent 1200 system equipped with a reversed-phase (RP) C_18_ column (5 µm, 10 × 250 mm, Welch Ultimate XB-C_18_). Column chromatography (CC) including Silica gel (100–200 and 200–300 mesh; Qingdao Marine Chemical Inc., China), ODS (50 μm, YMC Co. Ltd., Japan), Sephadex LH-20 (Pharmacia Biotech AB, Uppsala, Sweden), and MCI gel (75–150 μm, Merck, Germany) was used to separation and purification of the sample.

### Plant material

The aerial parts of *H. seniawinii* Maxim. were collected from the Shennongjia area in Hubei Province, People’s Republic of China. The plants were identified by Prof. C. G. Zhang of Huazhong University of Science and Technology (HUST). A voucher specimen (no. HP20230824) was deposited in the herbarium of Tongji Medical College, HUST.

### Extraction and isolation

The dried aerial parts of *H*. *seniawinii* Maxim. (20.0 kg) were powered and extracted with 95% EtOH (3 × 25 L) at room temperature, removing the solvents in *vacuo* to yield the crude extract (0.4 kg). The crude extract was then suspended in water and successively partitioned with CH_2_Cl_2_ and EtOAc. The CH_2_Cl_2_ extract was separated into seven fractions (A–G) by a silica gel CC (100–200 mesh), eluted with a gradient of petroleum ether–ethyl acetate (80:1–0:1). Fr. E was subsequently further chromatographed on an RP-C_18_ column (CH_3_OH–H_2_O, 50:50 to 100:0) to yield eight subfractions, E3a–E3h. Fr. E3b (1.0 g) was applied to Sephadex LH-20 (CH_3_OH), obtaining three subfractions, Fr. E3b1–E3b3. Fr. E3b2 (500 mg) was then purified on semipreparative HPLC to afford **2** (3.3 mg, *t*_R_ 19 min, CH_3_OH-H_2_O, 91:9, v/v, 2 mL/min), and **6** (3.6 mg, *t*_R_ 27 min, CH_3_OH-H_2_O, 80:20, v/v, 2 mL/min). Fr. E3c (1.2 g) was conducted on Sephadex LH-20 (CH_3_OH) and further purified by semi-preparative HPLC to afford **1** (7.5 mg, *t*_R_ 15 min, CH_3_OH-H_2_O, 88:12, v/v, 2 mL/min), **4** (1.7 mg, *t*_R_ 45 min, CH_3_OH-H_2_O, 77:23, v/v, 2 mL/min), and **5** (6.0 mg, *t*_R_ 12 min, CH_3_OH-H_2_O, 97:3, v/v, 2 mL/min). Compound **3** was isolated from Fr. E3e by silica gel CC (100–200 mesh), Sephadex LH-20 (CH_3_OH), and HPLC (2.4 mg, *t*_R_ 32 min, CH_3_OH-H_2_O, 94:6, v/v, 2 mL/min).

Hyperisenin A (**1**): Colorless oil; $[\alpha]_D^{29}$ +42.0 (*c* 0.1, CH_3_OH); UV (CH_3_OH) *λ*_max_ (log *ε*) = 201 (4.40) nm, 231 (4.06), 321 (3.77); IR (KBr) *v*_max_ 3424, 2973, 2929, 2873, 1721, 1634, 1603, 1553, 1490, 1449, 1382, 1363, 1328, 1233, 1180, 1148, 1066, 1027, 974, 838, 761, 694 cm^–1^; ECD (CH_3_OH) *λ*_max_ (Δ*ε*) 215 (+ 1.95), 241 (− 4.60), 365 (+ 1.81) nm. ^1^H and ^13^C NMR data see Table 1; positive HRESIMS: *m/z* 487.2802 [M + Na]^+^ (calcd. for C_30_H_40_O_4_Na^+^, 487.2819).

Hyperisenin B (**2**): Colorless oil; $[\alpha]_D^{29}$ +48.8 (*c* 0.3, CH_3_OH); UV (CH_3_OH) *λ*_max_ (log *ε*) = 201 (4.49), 240 (4.28) nm; IR (KBr) *v*_max_ 3458, 2972, 2928, 1756, 1715,1597, 1447, 1383, 1226, 1180, 988, 690 cm^–1^; ECD (CH_3_OH) *λ*_max_ (Δ*ε*) 206 (+ 4.15), 231 (− 3.45), 249 (+ 3.83), 267 (− 1.15), 303 (+ 5.13) nm. ^1^H and ^13^C NMR data, see Table 1; positive HRESIMS: *m/z* 515.2753 [M + Na]^+^ (calcd for C_31_H_40_O_5_Na^+^, 515.2768).

Hyperisenin C (**3**): Yellow oil; $[\alpha]_D^{29}$ +33.0 (*c* 0.4, CH_3_OH); UV (CH_3_OH) *λ*_max_ (log *ε*) = 205 (4.29) nm; IR (KBr) *v*_max_ 3409, 2970, 2927, 2874, 1722, 1666, 1603, 1449, 1418, 1382, 1326, 1292, 1219, 1123, 1106, 1074, 1019, 955 cm^–1^; ECD (CH_3_OH) *λ*_max_ (Δ*ε*) 213 (+ 5.45), 260 (+ 0.02), 296 (+ 1.48), 340 (− 1.39) nm. ^1^H and ^13^C NMR data; positive HRESIMS: *m/z* 609.3572 [M + Na]^+^ (calcd. for C_38_H_50_O_5_Na^+^, 609.3550).

Hyperisenin D (**4**): Yellow oil; $[\alpha]_D^{29}$ +22.1 (*c* 0.1, CH_3_OH); UV (CH_3_OH) *λ*_max_ (log *ε*) = 202 (4.60) nm; IR (KBr) *v*_max_ 3429, 2973, 2927, 2854, 1715, 1668, 1618, 1449, 1412, 1384, 1237, 1175, 1027, 961 cm^–1^; ECD (CH_3_OH) *λ*_max_ (Δ*ε*) 206 (+ 1.58), 215 (− 0.31), 264 (+ 2.99), 349 (− 0.04) nm. ^1^H and ^13^C NMR data; positive HRESIMS: *m/z* 557.2876 [M + Na]^+^ (calcd. for C_33_H_42_O_6_Na^+^, 557.2874).

### NMR and ECD calculations

The details of NMR and ECD calculations were put in the Supporting Information (SI).

### Strains and culture conditions

The GFP reporter strains were provided by Prof. Pinghua Sun from Jinan University and were cultured in Luria–Bertani (LB) medium (1% w/v NaCl, 1% w/v tryptone, and 0.5% w/v yeast extract) supplemented with 100 μg/mL ampicillin and 20 μg/mL gentamicin at 37 °C. The CRPA strain was provided by Prof. Yan He from Tongji Hospital at Huazhong University of Science and Technology and cultured in LB medium at 37 °C.

### Screening for QS inhibitors

GFP reporter strains were cultured overnight in LB medium supplemented with 100 μg/mL ampicillin and 20 μg/mL gentamicin at 37 °C. The cultures were then diluted 1:5 in ABTGC medium (2 g/L (NH_4_)_2_SO_4_, 6 g/L Na_2_HPO_4_, 3 g/L NaCl, 1 mM MgCl_2_, 0.1 mM CaCl_2_, 0.01 mM FeCl_3_, 0.025% thiamine, 0.5% glucose, 0.5% casamino acids). For the assay, 100 μL of either a 200 μM solution of the compound for high-throughput screening or gradient-diluted concentrations for secondary confirmation, or an equivalent volume of DMSO, was mixed with the diluted GFP reporter strain (100 μL). This mixture was transferred to a 96-well plate (200 μL per well) and incubated at 37 °C. A microplate reader continuously monitored bacterial growth at OD_600_ and GFP fluorescence (excitation at 485 nm, emission at 535 nm) every 15 min for approximately 24 h.

### Determination of virulence factor production

CRPA was cultured overnight in LB medium at 37 °C and then 1:10 in ABTGC medium successively. A 100 μL volume of either gradient-diluted compound or an equivalent volume of DMSO was combined with the diluted CRPA (100 μL). This mixture was incubated for 24 h at 37 °C with agitation at 200 rpm in a 96-well plate (200 μL per well). The OD_600_ of the culture was measured to normalize the virulence factor content. Virulence factors were then extracted from the supernatant after centrifugation at 4000 rpm for 15 min. Rhamnolipids were extracted with a three-fold volume of ethyl acetate three times. The ethyl acetate fraction was evaporated and redissolved in a freshly prepared reagent (0.19% orcinol in 60% H_2_SO_4_, 900 μL), incubated at 80 °C for 30 min, and then the solution (200 μL) was used to quantify rhamnolipid levels at OD_421_ after cooling to room temperature.

### Molecular docking

Molecular docking was performed using Schrödinger Maestro (Schrödinger Release 2023–1: Maestro, Schrödinger, LLC, New York, NY, 2023). Protein models were sourced from the Protein Data Bank (PDB) and prepared via the Protein Preparation Workflow module. Compound structures were pre-processed using the LigPrep module. Interactions and alignments were visualized and analyzed using PyMOL software.

## Supplementary Information

Supplementary material 1.

## Data Availability

The data that support the findings of this study are openly available in the Science Data Bank at.

## References

[1] Hutchings MI, Truman AW, Wilkinson B. Antibiotics: past, present and future. Curr Opin Microbiol. 2019;51:72–80. 31733401 10.1016/j.mib.2019.10.008

[2] de la Fuente-Nunez C, Cesaro A, Hancock REW. Antibiotic failure: beyond antimicrobial resistance. Drug Resist Updates. 2023;71:101012–21. 10.1016/j.drup.2023.101012 PMC12224857 37924726

[3] Okeke IN, de Kraker MEA, Van Boeckel TP, Kumar CK, Schmitt H, Gales AC, Bertagnolio S, Sharland M, Laxminarayan R. The scope of the antimicrobial resistance challenge. Lancet. 2024;403(10442):2426–38. 38797176 10.1016/S0140-6736(24)00876-6

[4] Ho CS, Wong CTH, Aung TT, Lakshminarayanan R, Mehta JS, Rauz S, McNally A, Kintses B, Peacock SJ, de la Fuente-Nunez C, Hancock REW, Ting DSJ. Antimicrobial resistance: a concise update. Lancet Microbe. 2025;6(1):100947–60. 39305919 10.1016/j.lanmic.2024.07.010

[5] Antimicrobial Resistance C. Global burden of bacterial antimicrobial resistance in 2019: a systematic analysis. Lancet. 2022;399(10325):629–55. 35065702 10.1016/S0140-6736(21)02724-0 PMC8841637

[6] Morrison L, Zembower TR. Antimicrobial resistance. Gastrointest Endosc Clin N Am. 2020;30(4):619–35. 32891221 10.1016/j.giec.2020.06.004

[7] Vashistha A, Sharma N, Nanaji Y, Kumar D, Singh G, Barnwal RP, Yadav AK. Quorum sensing inhibitors as therapeutics: bacterial biofilm inhibition. Bioorg Chem. 2023;136:106551–70. 37094480 10.1016/j.bioorg.2023.106551

[8] Papenfort K, Bassler BL. Quorum sensing signal–response systems in Gram-negative bacteria. Nat Rev Microbiol. 2016;14(9):576–88. 27510864 10.1038/nrmicro.2016.89 PMC5056591

[9] Mukherjee S, Bassler BL. Bacterial quorum sensing in complex and dynamically changing environments. Nat Rev Microbiol. 2019;17(6):371–82. 30944413 10.1038/s41579-019-0186-5 PMC6615036

[10] Azimi S, Klementiev AD, Whiteley M, Diggle SP. Bacterial quorum sensing during infection. Annu Rev Microbiol. 2020;74(1):201–19. 32660382 10.1146/annurev-micro-032020-093845 PMC13064819

[11] Maiga A, Ampomah-Wireko M, Li H, Fan Z, Lin Z, Zhen H, Kpekura S, Wu C. Multidrug-resistant bacteria quorum-sensing inhibitors: a particular focus on *Pseudomonas aeruginosa*. Eur J Med Chem. 2025;281:117008–31. 39500066 10.1016/j.ejmech.2024.117008

[12] Whiteley M, Diggle SP, Greenberg EP. Progress in and promise of bacterial quorum sensing research. Nature. 2017;551(7680):313–20. 29144467 10.1038/nature24624 PMC5870893

[13] Kalia VC, Patel SKS, Kang YC, Lee J-K. Quorum sensing inhibitors as antipathogens: biotechnological applications. Biotechnol Adv. 2019;37(1):68–90. 30471318 10.1016/j.biotechadv.2018.11.006

[14] Defoirdt T. Quorum-sensing systems as targets for antivirulence therapy. Trends Microbiol. 2018;26(4):313–28. 29132819 10.1016/j.tim.2017.10.005

[15] Newman DJ, Cragg GM. Natural products as sources of new drugs over the nearly four decades from 01/1981 to 09/2019. J Nat Prod. 2020;83(3):770–803. 32162523 10.1021/acs.jnatprod.9b01285

[16] Lewis K, Lee RE, Brötz-Oesterhelt H, Hiller S, Rodnina MV, Schneider T, Weingarth M, Wohlgemuth I. Sophisticated natural products as antibiotics. Nature. 2024;632(8023):39–49. 39085542 10.1038/s41586-024-07530-w PMC11573432

[17] Teplitski M, Robinson JB, Bauer WD. Plants secrete substances that mimic bacterial *N*-Acyl homoserine lactone signal activities and affect population density-dependent behaviors in associated bacteria. Mol Plant-Microbe In. 2000;13(6):637–48. 10.1094/MPMI.2000.13.6.637 10830263

[18] Xu S, Kang A, Tian Y, Li X, Qin S, Yang R, Guo Y. Plant flavonoids with antimicrobial activity against methicillin-resistant *Staphylococcus aureus* (MRSA). ACS Infect Dis. 2024;10(9):3086–97. 38833551 10.1021/acsinfecdis.4c00292

[19] Schiavone BI, Rosato A, Marilena M, Gibbons S, Bombardelli E, Verotta L, Franchini C, Corbo F. Biological evaluation of hyperforin and its hydrogenated analogue on bacterial growth and biofilm production. J Nat Prod. 2013;76(9):1819–23. 23981190 10.1021/np400394c

[20] Guttroff C, Baykal A, Wang H, Popella P, Kraus F, Biber N, Krauss S, Gotz F, Plietker B. Polycyclic polyprenylated acylphloroglucinols: an emerging class of non-peptide-based MRSA- and VRE-active antibiotics. Angew Chem Int Ed. 2017;56(50):15852–6. 10.1002/anie.201707069 28985019

[21] Deryabin D, Galadzhieva A, Kosyan D, Duskaev G. Plant-derived inhibitors of AHL-mediated quorum sensing in bacteria: modes of action. Int J Mol Sci. 2019;20(22):5588–609. 31717364 10.3390/ijms20225588 PMC6888686

[22] Norizan S, Yin W-F, Chan K-G. Caffeine as a potential quorum sensing inhibitor. Sensors. 2013;13(4):5117–29. 23598500 10.3390/s130405117 PMC3673129

[23] Zhou J-W, Luo H-Z, Jiang H, Jian T-K, Chen Z-Q, Jia A-Q. Hordenine: a novel quorum sensing inhibitor and antibiofilm agent against *Pseudomonas aeruginosa*. J Agric Food Chem. 2018;66(7):1620–8. 29353476 10.1021/acs.jafc.7b05035

[24] Jakobsen TH, Bragason SK, Phipps RK, Christensen LD, van Gennip M, Alhede M, Skindersoe M, Larsen TO, Høiby N, Bjarnsholt T, Givskov M. Food as a source for quorum sensing inhibitors: Iberin from horseradish revealed as a quorum sensing inhibitor of *Pseudomonas aeruginosa*. Appl Environ Microbiol. 2012;78(7):2410–21. 22286987 10.1128/AEM.05992-11 PMC3302586

[25] Lan X, Gu X, Zhang Y, Hu H, Shi Z, Xiong C, Huang X, Song B, Qiao Y, Sun W, Qi C, Zhang Y. Discovery of quorum sensing inhibitors against *Pseudomonas aeruginosa* from *Aspergillus* sp. NB12. Bioorg Chem. 2025;156:108230–40. 39914030 10.1016/j.bioorg.2025.108230

[26] Zhang R, Ji Y, Zhang X, Kennelly EJ, Long C. Ethnopharmacology of *Hypericum* species in China: a comprehensive review on ethnobotany, phytochemistry and pharmacology. J Ethnopharmacol. 2020;254:112686–97. 32101776 10.1016/j.jep.2020.112686

[27] Xia J, Hu B, Qian M, Zhang J, Wu L. Benzophenone Rhamnosides and Chromones from *Hypericum seniawinii* Maxim. Molecules. 2022;27(20):7056–65. 36296651 10.3390/molecules27207056 PMC9609419

[28] Yang XW, Grossman RB, Xu G. Research progress of polycyclic polyprenylated acylphloroglucinols. Chem Rev. 2018;118(7):3508–58. 29461053 10.1021/acs.chemrev.7b00551

[29] Xie S, Zhou Y, Tan X, Sun W, Duan Y, Feng H, Sun L, Guo Y, Shi Z, Hao X, Chen G, Qi C, Zhang Y, Norwilsonnol A. an immunosuppressive polycyclic polyprenylated acylphloroglucinol with a spiro[5-oxatricyclododecane-[6.4.0.0^3,7^]dodecane-6′,1-1′,2′-dioxane] system from *Hypericum wilsonii*. Org Chem Front. 2021;8(10):2280–6.

[30] Sun H, Wang J, Zhen B, Wang X, Suo X, Lin M, Jiang J, Ji T. Polycyclic polyprenylated acylphloroglucinol derivatives from *Hypericum pseudohenryi*. Phytochemistry. 2021;187:112761–8. 33933827 10.1016/j.phytochem.2021.112761

[31] Guo Y, Tong Q, Zhang N, Duan X, Cao Y, Zhu H, Xie S, Yang J, Zhang J, Liu Y, Xue Y, Zhang Y. Highly functionalized cyclohexanone-monocyclic polyprenylated acylphloroglucinols from *Hypericum perforatum* induce leukemia cell apoptosis. Org Chem Front. 2019;6(6):817–24.

[32] Duan YL, Deng YF, Bu PF, Xie S, Guo Y, Shi Z, Guo Y, Cao Y, Qi C, Zhang Y. Discovery of *nor*-bicyclic polyprenylated acylphloroglucinols possessing diverse architectures with anti-hepatoma activities from *Hypericum patulum*. Bioorg Chem. 2021;111:104902–12. 33894431 10.1016/j.bioorg.2021.104902

[33] Fobofou SA, Franke K, Sanna G, Porzel A, Bullita E, La Colla P, Wessjohann LA. Isolation and anticancer, anthelminthic, and antiviral (HIV) activity of acylphloroglucinols, and regioselective synthesis of empetrifranzinans from *Hypericum roeperianum*. Bioorg Med Chem. 2015;23(19):6327–34. 26358281 10.1016/j.bmc.2015.08.028

[34] Zeng YR, Li YN, Lou HY, Jian JY, Gu W, Huang LJ, Du GH, Yuan CM, Hao XJ. Polycyclic polyprenylated acylphloroglucinol derivatives with neuroprotective effects from *Hypericum monogynum*. J Asian Nat Prod Res. 2021;23(1):73–81. 31838892 10.1080/10286020.2019.1698551

[35] Seo E-K, Wani MC, Wall ME, Navarro H, Mukherjee R, Farnsworth NR, Kinghorn AD. New bioactive aromatic compounds from *Vismia guianensis*. Phytochemistry. 2000;55(1):35–42. 11021642 10.1016/s0031-9422(00)00208-9

[36] Duan Y, Guo Y, Deng Y, Bu P, Shi Z, Cao Y, Zhang Y, Hu H, Sun W, Qi C, Zhang Y, Norprzewalsone A. a Rearranged Polycyclic Polyprenylated Acylphloroglucinol with a Spiro[cyclopentane-1,3’-tricyclo[7.4.0.0.^1,6^]tridecane] Core from *Hypericum przewalskii*. J Org Chem. 2022;87(10):6824–31. 35545918 10.1021/acs.joc.2c00503

[37] Duan Y, Sun W, Li Y, Shi Z, Li L, Zhang Y, Huang K, Zhang Z, Qi C, Zhang Y. Spirohypertones A and B as potent antipsoriatics: Tumor necrosis factor-*α* inhibitors with unprecedented chemical architectures. Acta Pharm Sin B. 2024;14(6):2646–56. 38828134 10.1016/j.apsb.2024.02.002 PMC11143743

[38] Lee J, Zhang L. The hierarchy quorum sensing network in *Pseudomonas aeruginosa*. Protein Cell. 2015;6(1):26–41. 25249263 10.1007/s13238-014-0100-x PMC4286720

