# Supplementary material for: Discovery of structurally diverse polyprenylated acylphloroglucinols with quorum sensing inhibitory activity from Hypericum seniawinii Maxim
Source: Nat Prod Bioprospect. 2025 Jun 19;15(1):40. doi: 10.1007/s13659-025-00520-z (PMC12179027; doi:10.1007/s13659-025-00520-z)
Supplement: Supplementary file 1 — Supplementary material 1. [file 13659_2025_520_MOESM1_ESM.docx]

**Supporting Information**

**Discovery of Structurally Diverse Polyprenylated Acylphloroglucinols with** **Quorum Sensing Inhibitory Activity from** ***Hypericum seniawinii* Maxim.**

Yulin Duan,^a,b,1^ Xiaoxia Gu,^a,1^ Xincai Hao,^d^ Guosheng Cao,^e,f,g^* Weiguang Sun,^a^,* Changxing Qi,^a,c^* and Yonghui Zhang^a^,*

*^a^**Hubei Key Laboratory of Natural Medicinal Chemistry and Resource Evaluation, School of Pharmacy, Tongji Medical College, Huazhong University of Science and Technology, Wuhan 430030,* *Hubei Province, People’s Republic of China*

*^b^Department of Pharmacy, Wuhan No.1 Hospital, Wuhan 430022, Hubei, People’s Republic of China*

*^c^Key Laboratory of Technology of Drug Preparation (Zhengzhou University), Ministry of Education of China; Key Laboratory of Henan Province for Drug Quality and Evaluation; Institute of Pharmaceutical Sciences, Zhengzhou University, Zhengzhou 450001, China*

*^d^Hubei Key Laboratory of Wudang Local Chinese Medicine Research, Hubei Engineering Technology Center for Comprehensive Utilization of Medicinal Plants, College of Pharmacy, Hubei University of Medicine, Shiyan 442000, People’s Republic of China*

*^e^College of Pharmacy, Hubei University of Chinese Medicine, Wuhan, 430065, People’s Republic of China*

*^f^Hubei Shizhen Laboratory, Wuhan, 430065, People’s Republic of China*

*^g^Key Laboratory of Traditional Chinese Medicine Resource and Compound Prescription, Ministry of Education, Hubei University of Chinese Medicine, Wuhan, 430065, People’s Republic of China*

*Corresponding Author: zhangyh@mails.tjmu.edu.cn (Y. Zhang); qichangxing@hust.edu.cn (C. Qi); sunweiguang@hust.edu.cn (W. Sun); caoguosheng2006@163.com (G. Cao)

^1^These authors contributed equally.

| **Contents** | **Pages** |
| --- | --- |
| **1. NMR, HRESIMS, IR, and UV spectra for compounds 1–4** | 3 |
| **Figure S1–S6.** 1D and 2D NMR (CDCl_3_) spectra of compound **1**. | 3 |
| **Figure S7–S9.** HRMS (ESI-TOF), UV, and IR spectra of compound **1**. | 6 |
| **Figure S10–S15.** 1D and 2D NMR (CD_3_OD) spectra of compound **2**. | 7 |
| **Figure S16–S18.** HRMS (ESI-TOF), UV, and IR spectra of compound **2**. | 10 |
| **Figure S19–S24.** 1D and 2D NMR (CD_3_OD) spectra of compound **3**. | 12 |
| **Figure S25–S27.** HRMS (ESI-TOF), UV, and IR spectra of compound **3**. | 15 |
| **Figure S28–S33.** 1D and 2D NMR (CDCl_3_) spectra of compound **4**. | 16 |
| **Figure S34–S36.** HRMS (ESI-TOF), UV, and IR spectra of compound **4**. | 19 |
| **2. The details of NMR calculations for compounds 1 and 2.** | 20 |
| **Figure S37.** The DP4+ analysis of experimental NMR chemical shifts of **1** and calculated experimental NMR chemical shifts. | 22 |
| Gibbs free energies, equilibrium populations, and cartesian coordinates of low-energy conformers in **1**. | 23 |
| **Figure S40.** The DP4+ analysis of experimental NMR chemical shifts of **2** and calculated experimental NMR chemical shifts. | 42 |
| **Figure S41.** The comparison of partial NMR data between **2**, spirohypolactones A and B, and norhyperpalum H. | 43 |
| Gibbs free energies, equilibrium populations, and cartesian coordinates of low-energy conformers in **2**. | 43 |
| **3. The details of ECD calculations for compounds 1–4** | 120 |
| Gibbs free energies, equilibrium populations, and cartesian coordinates of low-energy conformers in **1**. | 121 |
| Gibbs free energies, equilibrium populations, and cartesian coordinates of low-energy conformers in **2**. | 132 |
| Gibbs free energies, equilibrium populations, and cartesian coordinates of low-energy conformers in **3**. | 141 |
| Gibbs free energies, equilibrium populations, and cartesian coordinates of low-energy conformers in **4**. | 152 |
| **Figure 46. The quorum sensing inhibitory activity against *Pseudomonas aeruginosa* of compounds 1–6.** | 184 |

**1**. **NMR, HRESIMS, IR and UV spectra for compounds 1–4.**

**Figure S1.** ^1^H-NMR spectrum (400 MHz, CDCl_3_) of compound **1**.

**Figure S2.** ^13^C and DEPT NMR spectra (100 MHz, CDCl_3_) of compound **1**.

**Figure S3.** HSQC spectrum of compound **1**.

**Figure S4.** HMBC spectrum of compound **1.**

**Figure S5.** ^1^H–^1^H COSY spectrum of compound **1**.

**Figure S6.** NOESY spectrum of compound **1**.

**Figure S7.** HR-ESI-MS spectrum of compound **1.**

**Figure S8.** UV spectrum of compound **1.**

**Figure S9.** IR spectrum of compound **1.**

**Figure S10.** ^1^H-NMR spectrum (600 MHz, CD_3_OD) of compound **2**.

**Figure S11.** ^13^C and DEPT NMR spectra (150 MHz, CD_3_OD) of compound **2**.

**Figure S12.** HSQC spectrum of compound **2**.

**Figure S13.** HMBC spectrum of compound **2**.

**Figure S14.** ^1^H–^1^H COSY spectrum of compound **2**.

**Figure S15.** NOESY spectrum of compound **2**.

**Figure S16.** HR-ESI-MS spectrum of compound **2**.

**Figure S17.** UV spectrum of compound **2**.

**Figure S18.** IR spectrum of compound **2**.

**Figure S19.** ^1^H-NMR spectrum (600 MHz, CD_3_OD) of compound **3**.

**Figure S20.** ^13^C and DEPT NMR spectra (150 MHz, CD_3_OD) of compound **3**.

**Figure S21.** HSQC spectrum of compound **3**.

**Figure S22.** HMBC spectrum of compound **3**.

**Figure S23.** ^1^H–^1^H COSY spectrum of compound **3**.

**Figure S24.** NOESY spectrum of compound **3**.

**Figure S25.** HR-ESI-MS spectrum of compound **3**.

**Figure S26.** UV spectrum of compound **3**.

**Figure S27.** IR spectrum of compound **3**.

**Figure S28.** ^1^H-NMR spectrum (600 MHz, CDCl_3_) of compound **4**.

**Figure S29.** ^13^C and DEPT NMR spectra (150 MHz, CDCl_3_) of compound **4**.

**Figure S30.** HSQC spectrum of compound **4**.

**Figure S31.** HMBC spectrum of compound **4**.

**Figure S32.** ^1^H–^1^H COSY spectrum of compound **4**.

**Figure S33.** NOESY spectrum of compound **4**.

**Figure S34.** HR-ESI-MS spectrum of compound **4**.

**Figure S35.** UV spectrum of compound **4**.

**Figure S36.** IR spectrum of compound **4**.

**2. The details of NMR calculations for compounds 1 and 2.**

Conformational analyses were carried out via random searching in the Sybyl-X 2.0 using the MMFF94S force field with an energy cutoff of 5.0 kcal/mol.^[1]^ Subsequently, the conformers were re-optimized at the B3LYP-D3(BJ)/6-31G* level by the GAUSSIAN09 program.^[2]^All conformers used for property calculations in this work were characterized to be stable point on potential energy surface (PES) with no imaginary frequencies. NMR shielding constants were computed using the GIAO method at the mPW1PW91/6-311+G** level by the GAUSSIAN09 program.^[2]^ Gibbs free energies for conformers were determined by using thermal correction at B3LYP-D3(BJ)/6-31G* level and electronic energies evaluated at the wB97M-V/def2-TZVP level in PCM chloroform using ORCA.^[3][4]^ Boltzmann weights were computed using relative gibbs free energies.^[5]^ The unscaled chemical shifts (δ_u_) were computed using TMS (Tetramethylsilane) as a reference standard according to δ_u_ = σ_0_ - σ_x_, where σ_x_ is the Boltzmann averaged shielding tensor (over all significantly populated conformations) and σ_0_ is the shielding tensor of the TMS computed at the same level of theory employed for σ_x_. The scaled chemical shifts (δ_s_) were calculated as δ_s_ = (δ_u_ - b)/m, where m and b are the slope and intercept, respectively, deduced from a linear regression calculation on a plot of δ_u_ against δ_exp_. The DP4+ calculations were run by the Excel spreadsheet available for free. ^[5]^

**References**

[1] Sybyl Software, version X 2.0; Tripos Associates Inc.: St. Louis, MO, 2013.

[2] Gaussian 09, Revision E.01, M. J. Frisch, G. W. Trucks, H. B. Schlegel, G. E. Scuseria, M. A. Robb, J. R. Cheeseman, G. Scalmani, V. Barone, B. Mennucci, G. A. Petersson, H. Nakatsuji, M. Caricato, X. Li, H. P. Hratchian, A. F. Izmaylov, J. Bloino, G. Zheng, J. L. Sonnenberg, M. Hada, M. Ehara, K. Toyota, R. Fukuda, J. Hasegawa, M. Ishida, T. Nakajima, Y. Honda, O. Kitao, H. Nakai, T. Vreven, J. A. Montgomery, Jr., J. E. Peralta, F. Ogliaro, M. Bearpark, J. J. Heyd, E. Brothers, K. N. Kudin, V. N. Staroverov, R. Kobayashi, J. Normand, K. Raghavachari, A. Rendell, J. C. Burant, S. S. Iyengar, J. Tomasi, M. Cossi, N. Rega, J. M. Millam, M. Klene, J. E. Knox, J. B. Cross, V. Bakken, C. Adamo, J. Jaramillo, R. Gomperts, R. E. Stratmann, O. Yazyev, A. J. Austin, R. Cammi, C. Pomelli, J. W. Ochterski, R. L. Martin, K. Morokuma, V. G. Zakrzewski, G. A. Voth, P. Salvador, J. J. Dannenberg, S. Dapprich, A. D. Daniels, Ö. Farkas, J. B. Foresman, J. V. Ortiz, J. Cioslowski, and D. J. Fox, Gaussian, Inc., Wallingford CT, 2009.

[3] F. Neese, The ORCA program system, Wiley Interdiscip. Comput. Mol. Sci. 2 (2012) 73-78.
[4] F. Neese, Software update: the ORCA program system, version 4.0, Wiley Interdiscip. Comput. Mol. Sci. 8 (2017) e1327.

[5] Nicolás Grimblat, María M. Zanardi, and Ariel M. Sarotti J. Org. Chem. 2015, 80, 12526−12534.

**For compound 1**

**
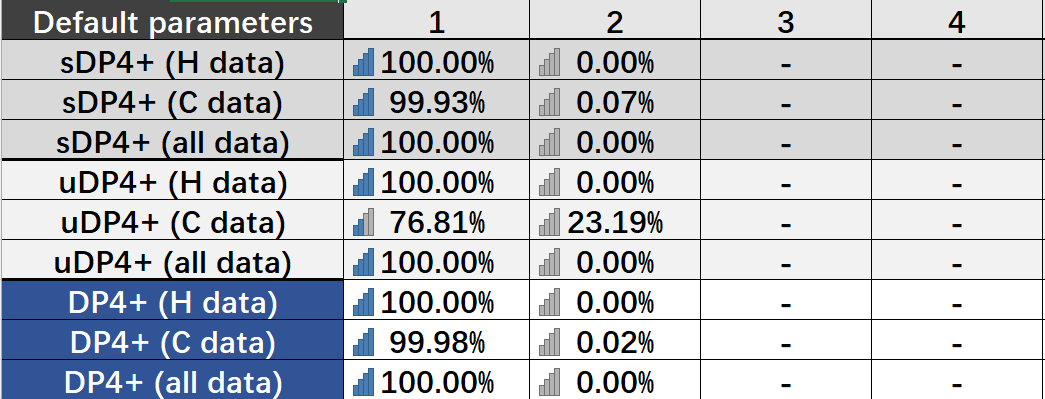
**

**
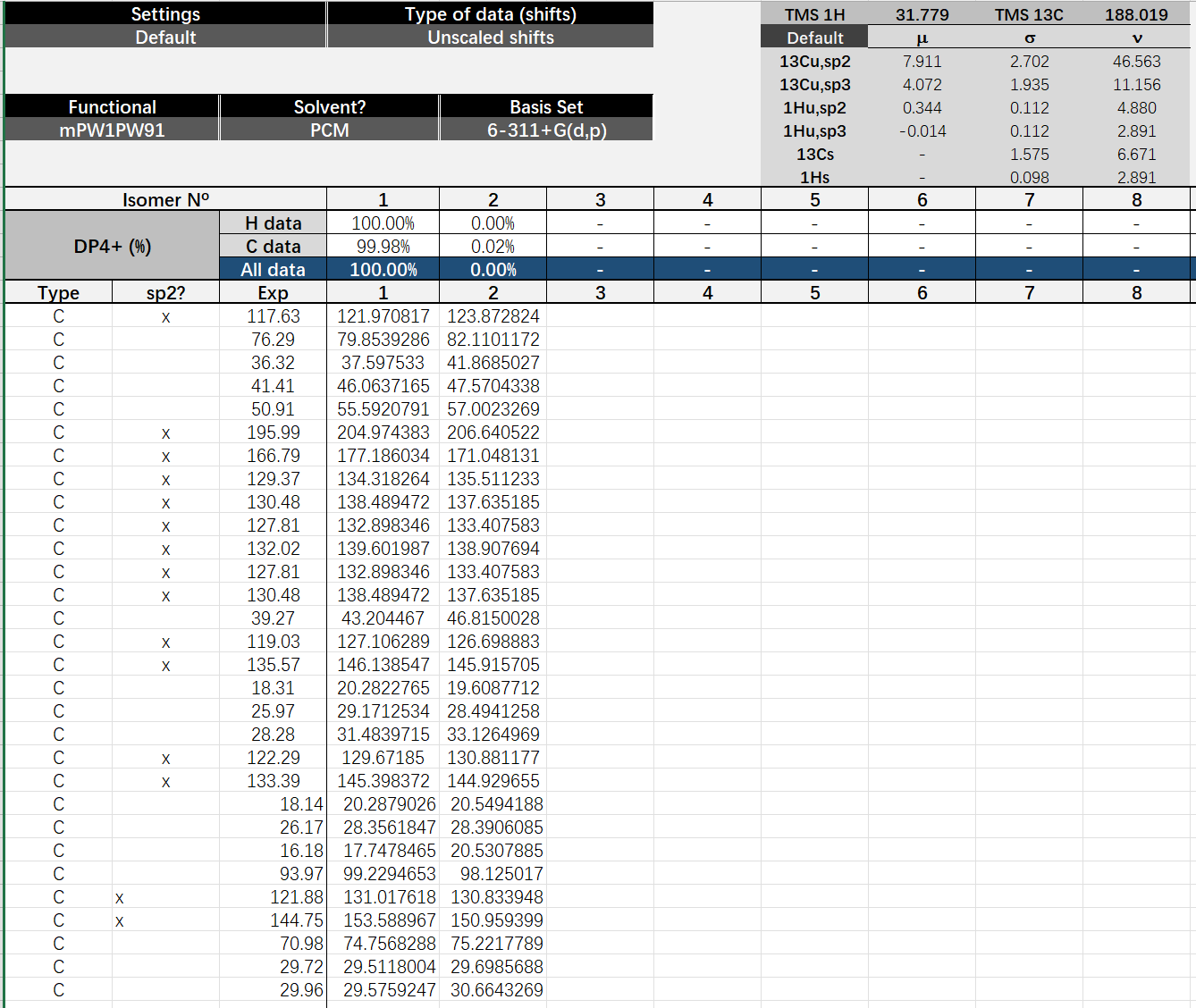
**

**
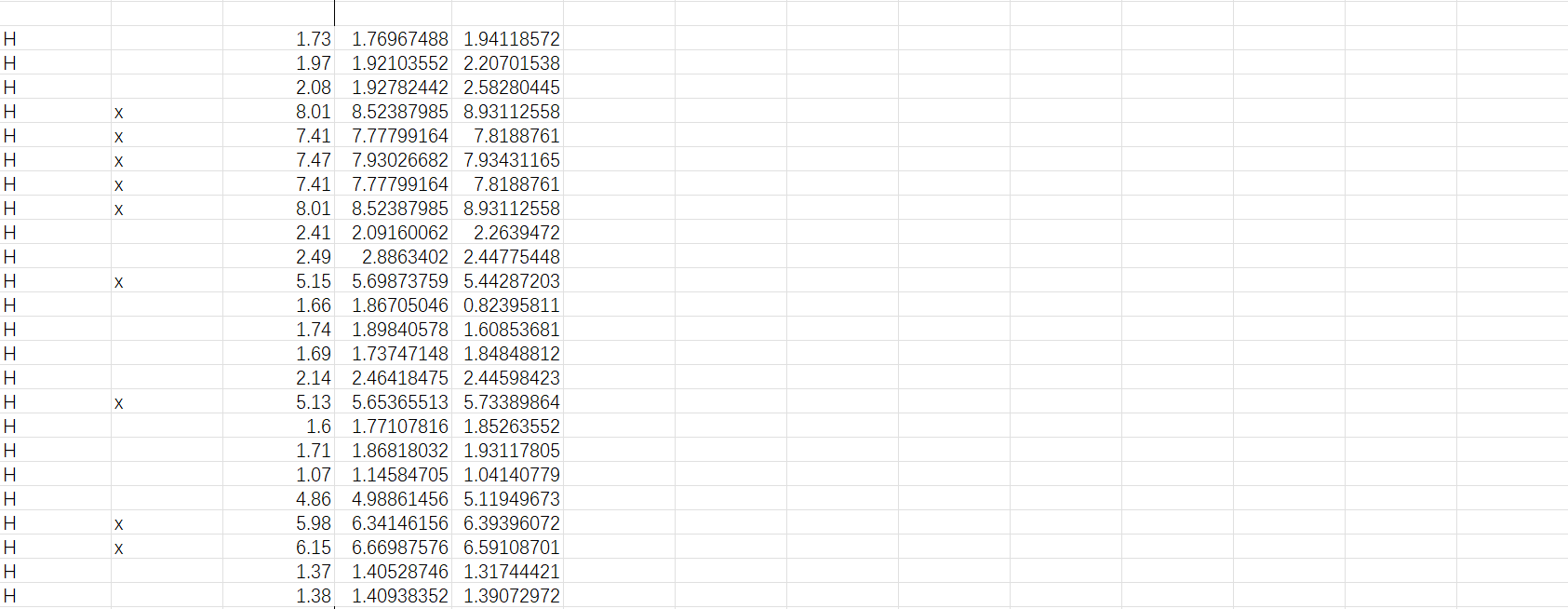
**

**Figure S37.** The DP4+ analysis of experimental NMR chemical shifts of **1** and calculated experimental NMR chemical shifts of Isomer 1: (3*R**,5*R**,6*S**,26*R**)-**1** (**1A**), and Isomer 2: (3*S**,5*R**,6*S**,26*R**)-**1** (**1B**).

**Figure S38**. Optimized geometries of predominant conformers for **1A** at the B3LYP-D3(BJ)/6-31G^*^ level in PCM chloroform by the Gaussian09 program.

**Table S1**. Gibbs free energies^a^ and equilibrium populations^b^ of low-energy conformers of **1A**.

| Conformers | ∆G(a.u.) | P(%)/100 | G(a.u.) |
| --- | --- | --- | --- |
| **1A-1** | 0.00452 | 0.22 | -1467.538155 |
| **1A-2** | 0.00133 | 6.46 | -1467.541341 |
| **1A-3** | 0.00364 | 0.56 | -1467.539029 |
| **1A-4** | 0.00025 | 20.37 | -1467.542424 |
| **1A-5** | 0.00245 | 1.98 | -1467.540225 |
| **1A-6** | 0.0 | 26.52 | -1467.542673 |
| **1A-7** | 0.00086 | 10.68 | -1467.541814 |
| **1A-8** | 0.00082 | 11.12 | -1467.541853 |
| **1A-9** | 0.00017 | 22.09 | -1467.542501 |

^a^wB97M-V/def2-TZVP, in a.u.
^b^From ∆G values at 298.15K.

**Figure S39**. Optimized geometries of predominant conformers for **1B** at the B3LYP-D3(BJ)/6-31G^*^ level in PCM chloroform by the Gaussian09 program.

**Table S2**. Gibbs free energies^a^ and equilibrium populations^b^ of low-energy conformers of **1B**.

| Conformers | ∆G(a.u.) | P(%)/100 | G(a.u.) |
| --- | --- | --- | --- |
| **1B-1** | 0.00238 | 3.12 | -1467.542228 |
| **1B-2** | 0.0 | 38.85 | -1467.544609 |
| **1B-3** | 0.0026 | 2.48 | -1467.542011 |
| **1B-4** | 0.00186 | 5.4 | -1467.542745 |
| **1B-5** | 0.00276 | 2.09 | -1467.541849 |
| **1B-6** | 0.00241 | 3.03 | -1467.542201 |
| **1B-7** | 0.00038 | 25.95 | -1467.544228 |
| **1B-8** | 0.00067 | 19.07 | -1467.543937 |

^a^wB97M-V/def2-TZVP, in a.u.
^b^From ∆G values at 298.15K.

**Table S3.** Cartesian coordinates for the low-energy reoptimized random research conformers of **1A** at B3LYP-D3(BJ)/6-31G* level of theory in chloroform.

| **1A-1** | | | | **1A-2** | | | |
| --- | --- | --- | --- | --- | --- | --- | --- |
| Atom | X | Y | Z | Atom | X | Y | Z |
| C | -1.769079 | 4.653587 | -5.450096 | C | -1.81449 | 3.78196 | -6.24983 |
| C | -2.684271 | -1.578693 | 0.124941 | C | -2.33165 | -3.14806 | -1.09898 |
| C | -5.010696 | -1.523431 | 8.618101 | C | -1.74818 | 5.84601 | 8.49748 |
| C | -4.284241 | 3.170264 | 8.114741 | C | 1.92539 | 3.77707 | 6.24019 |
| C | -3.454586 | 0.541925 | 7.437949 | C | -0.87695 | 3.8292 | 6.69791 |
| C | -1.518509 | -0.0088 | 5.904878 | C | -2.51579 | 2.25075 | 5.59556 |
| C | 0.238368 | 1.782161 | 4.583119 | C | -1.87075 | 0.21216 | 3.72255 |
| C | -7.585691 | 2.509938 | -6.592067 | C | -10.997 | -0.9825 | -1.89609 |
| C | -9.778981 | -0.440156 | -3.547354 | C | -13.0883 | 2.93601 | -0.15929 |
| O | -8.199768 | 1.8982 | -3.890364 | O | -10.9057 | 1.84332 | -1.61745 |
| C | -7.451327 | 3.252865 | -1.887295 | C | -9.08905 | 3.34191 | -2.54079 |
| O | -1.723429 | 7.487011 | -1.103355 | O | -4.15086 | 5.99609 | -2.29892 |
| C | -5.838474 | 5.584638 | -1.864257 | C | -6.78303 | 2.59218 | -3.98602 |
| C | -3.03505 | 5.108649 | -1.173715 | C | -4.31056 | 3.31079 | -2.56582 |
| C | -2.748673 | 3.983656 | 1.479178 | C | -4.28265 | 2.17076 | 0.09649 |
| C | -0.337867 | 2.432587 | 1.788346 | C | -1.71057 | 1.204 | 0.98452 |
| C | -0.405404 | 0.218586 | -0.105449 | C | -0.699 | -0.75013 | -0.92046 |
| C | -0.449915 | 1.295367 | -2.781673 | C | -0.49966 | 0.46087 | -3.53331 |
| C | -1.751118 | 3.612807 | -3.360956 | C | -2.10758 | 2.51174 | -4.31339 |
| C | 2.120514 | -1.243424 | -0.197623 | C | 2.11881 | -1.41772 | -0.49337 |
| O | 2.404099 | -1.896874 | -2.888651 | O | 3.09007 | -1.8253 | -3.07597 |
| C | 1.101205 | -0.163797 | -4.257517 | C | 1.6534 | -0.42997 | -4.66796 |
| C | 1.626659 | -0.264642 | -6.961464 | C | 2.67457 | -0.21997 | -7.21633 |
| C | -0.167067 | 0.501 | -8.751071 | C | 1.09088 | 0.15728 | -9.30367 |
| C | 0.368121 | 0.31224 | -11.310523 | C | 2.10689 | 0.27432 | -11.7163 |
| C | 2.69086 | -0.624168 | -12.11987 | C | 4.70307 | 0.02954 | -12.0801 |
| C | 4.479259 | -1.399714 | -10.352107 | C | 6.28709 | -0.35941 | -10.0156 |
| C | 3.950936 | -1.239708 | -7.789234 | C | 5.28386 | -0.49984 | -7.59698 |
| C | 2.311267 | -3.577097 | 1.353038 | C | 2.76722 | -3.67327 | 1.04927 |
| C | 3.998345 | -3.844721 | 3.201253 | C | 4.25708 | -3.53023 | 3.06785 |
| C | 4.169917 | -5.990346 | 5.06944 | C | 4.87865 | -5.63392 | 4.87883 |
| C | 6.787704 | -7.196372 | 4.941002 | C | 4.28729 | -8.26334 | 3.85053 |
| C | 2.105622 | -7.977344 | 4.74788 | C | 3.45527 | -5.14923 | 7.36294 |
| O | 4.01968 | -4.894148 | 7.569985 | O | 7.55982 | -5.38007 | 5.30036 |
| H | -4.43417 | -0.57976 | -0.328803 | H | -1.50549 | -4.46727 | -2.46328 |
| H | -2.49105 | -3.10012 | -1.266756 | H | -2.49785 | -4.11463 | 0.72341 |
| H | -2.875753 | -2.41283 | 2.002315 | H | -4.23258 | -2.68984 | -1.76583 |
| H | -4.371003 | -3.400447 | 8.035045 | H | -3.79756 | 5.80951 | 8.75495 |
| H | -4.927387 | -1.421613 | 10.68837 | H | -0.85115 | 5.61805 | 10.3544 |
| H | -7.009951 | -1.327784 | 8.09835 | H | -1.21308 | 7.72806 | 7.80762 |
| H | -4.442669 | 3.369431 | 10.172588 | H | 2.51206 | 2.22735 | 5.00998 |
| H | -3.010245 | 4.632937 | 7.416154 | H | 2.54495 | 5.55299 | 5.36428 |
| H | -6.170515 | 3.552136 | 7.338204 | H | 2.95647 | 3.61187 | 8.03251 |
| H | -1.139928 | -2.008397 | 5.572135 | H | -4.51205 | 2.4896 | 6.04042 |
| H | 0.329612 | 3.585231 | 5.599293 | H | -0.07976 | -0.69444 | 4.22872 |
| H | 2.151208 | 0.985061 | 4.673905 | H | -3.31837 | -1.27029 | 3.81618 |
| H | -6.650606 | 0.88784 | -7.487828 | H | -12.7666 | -1.56132 | -2.81133 |
| H | -9.332049 | 2.839579 | -7.663412 | H | -9.4336 | -1.75432 | -2.99833 |
| H | -6.351683 | 4.146507 | -6.803917 | H | -10.98 | -1.89274 | -0.03074 |
| H | -10.223334 | -0.788829 | -1.559504 | H | -13.1729 | 2.13214 | 1.7524 |
| H | -11.560985 | -0.291086 | -4.598952 | H | -12.9465 | 4.99003 | 0.00652 |
| H | -8.789885 | -2.104253 | -4.295905 | H | -14.8943 | 2.47742 | -1.07247 |
| H | -8.071255 | 2.584115 | -0.038271 | H | -9.23967 | 5.36358 | -2.17989 |
| H | -1.530462 | 8.002683 | -2.849655 | H | -3.60738 | 6.60965 | -3.93955 |
| H | -6.559784 | 6.926521 | -0.455173 | H | -6.72062 | 3.58049 | -5.81114 |
| H | -5.862805 | 6.544106 | -3.6966 | H | -6.74459 | 0.56208 | -4.40106 |
| H | -4.385563 | 2.818277 | 1.967229 | H | -5.66177 | 0.62615 | 0.20344 |
| H | -2.741868 | 5.582504 | 2.796878 | H | -4.96745 | 3.63219 | 1.39044 |
| H | 1.240044 | 3.642268 | 1.171224 | H | -0.38063 | 2.80576 | 0.97266 |
| H | 3.683914 | 0.05604 | 0.245881 | H | 3.10912 | 0.26335 | 0.23239 |
| H | -1.960228 | 1.254339 | -8.120747 | H | -0.92505 | 0.35963 | -9.01933 |
| H | -1.036426 | 0.905622 | -12.683942 | H | 0.86628 | 0.5612 | -13.3254 |
| H | 3.105058 | -0.753457 | -14.126393 | H | 5.48921 | 0.13532 | -13.9736 |
| H | 6.291955 | -2.13356 | -10.974178 | H | 8.31097 | -0.55597 | -10.2919 |
| H | 5.334515 | -1.851089 | -6.406747 | H | 6.50829 | -0.81174 | -5.98325 |
| H | 0.934623 | -5.05016 | 0.957124 | H | 1.96073 | -5.46467 | 0.44751 |
| H | 5.377358 | -2.347007 | 3.538999 | H | 5.0891 | -1.71799 | 3.59445 |
| H | 7.074988 | -8.101663 | 3.105437 | H | 2.25816 | -8.52016 | 3.539 |
| H | 8.25211 | -5.763907 | 5.217517 | H | 5.2918 | -8.58793 | 2.07399 |
| H | 6.975003 | -8.616382 | 6.433099 | H | 4.89033 | -9.69703 | 5.21777 |
| H | 2.244238 | -8.92156 | 2.911834 | H | 3.91465 | -3.27167 | 8.09821 |
| H | 0.222228 | -7.129545 | 4.903469 | H | 3.99286 | -6.56091 | 8.7835 |
| H | 2.298304 | -9.399956 | 6.234037 | H | 1.40945 | -5.26602 | 7.07776 |
| H | 2.455674 | -3.956919 | 7.676102 | H | 8.02874 | -6.56718 | 6.60219 |
| **1A-3** | | | | **1A-4** | | | |
| Atom | X | Y | Z | Atom | X | Y | Z |
| C | -2.25252 | 4.37963 | -4.69006 | C | -2.35715 | 4.80317 | -4.63107 |
| C | -3.05546 | -2.50538 | 0.182969 | C | -3.02256 | -2.83255 | -0.80697 |
| C | -2.11931 | 5.815199 | 10.42542 | C | -1.14904 | 3.73988 | 10.4367 |
| C | 1.546821 | 3.663392 | 8.243309 | C | 2.18241 | 1.87958 | 7.55561 |
| C | -1.26994 | 3.877863 | 8.530707 | C | -0.57746 | 2.10869 | 8.1834 |
| C | -2.9402 | 2.497031 | 7.228861 | C | -2.42243 | 0.99151 | 6.86446 |
| C | -2.33585 | 0.532581 | 5.267614 | C | -2.08365 | -0.63252 | 4.55542 |
| C | -7.15103 | 1.749516 | -7.41528 | C | -7.58887 | 2.89927 | -7.54659 |
| C | -9.36927 | -1.90948 | -5.3027 | C | -9.53009 | -1.20312 | -6.06071 |
| O | -8.20938 | 0.669625 | -5.01474 | O | -8.38625 | 1.29771 | -5.34199 |
| C | -8.18399 | 1.825752 | -2.76275 | C | -8.17315 | 1.96825 | -2.91031 |
| O | -3.60557 | 6.719841 | -0.22187 | O | -3.49901 | 6.43368 | 0.18184 |
| C | -7.076 | 4.353744 | -2.1096 | C | -7.06749 | 4.3573 | -1.85579 |
| C | -4.46647 | 4.206669 | -0.79812 | C | -4.39102 | 4.03279 | -0.71577 |
| C | -4.61283 | 2.829301 | 1.735274 | C | -4.42115 | 2.28166 | 1.57894 |
| C | -2.12036 | 1.641919 | 2.581052 | C | -1.92608 | 0.91977 | 2.09136 |
| C | -1.24113 | -0.26928 | 0.565082 | C | -1.18543 | -0.64971 | -0.24405 |
| C | -0.90458 | 1.095316 | -1.95017 | C | -1.03204 | 1.04527 | -2.57554 |
| C | -2.49524 | 3.169371 | -2.71002 | C | -2.54991 | 3.28266 | -2.87053 |
| C | 1.51404 | -1.20564 | 1.003883 | C | 1.58498 | -1.58892 | -0.15752 |
| O | 2.631017 | -1.28187 | -1.51419 | O | 2.37088 | -1.54173 | -2.83347 |
| C | 1.284546 | 0.26286 | -3.04867 | C | 0.96046 | 0.23585 | -4.01976 |
| C | 2.460392 | 0.676009 | -5.50749 | C | 1.83656 | 0.85583 | -6.55623 |
| C | 5.098786 | 0.528086 | -5.72261 | C | 4.39583 | 0.53006 | -7.16779 |
| C | 6.253024 | 0.854214 | -8.05398 | C | 5.24992 | 1.05553 | -9.59086 |
| C | 4.793332 | 1.292004 | -10.1992 | C | 3.56283 | 1.87623 | -11.436 |
| C | 2.171167 | 1.400516 | -10.0029 | C | 1.01314 | 2.16761 | -10.8482 |
| C | 1.003587 | 1.101634 | -7.67483 | C | 0.14856 | 1.66878 | -8.4251 |
| C | 1.859924 | -3.69849 | 2.269623 | C | 2.11397 | -4.14945 | 0.86857 |
| C | 2.760802 | -5.77119 | 1.176343 | C | 3.7261 | -4.53984 | 2.76187 |
| C | 3.190426 | -8.2728 | 2.476531 | C | 4.31981 | -7.02202 | 4.027 |
| C | 1.282044 | -8.80904 | 4.577294 | C | 3.06562 | -9.29815 | 2.77614 |
| C | 5.90517 | -8.37366 | 3.494689 | C | 3.59087 | -6.85112 | 6.8161 |
| O | 2.893383 | -10.113 | 0.485965 | O | 7.03283 | -7.33766 | 4.02251 |
| H | -4.85085 | -1.85751 | -0.61117 | H | -3.21534 | -4.12146 | 0.80038 |
| H | -2.23251 | -3.84776 | -1.15861 | H | -4.88765 | -2.09441 | -1.30299 |
| H | -3.41735 | -3.53046 | 1.94388 | H | -2.35016 | -3.91641 | -2.4374 |
| H | -1.32168 | 5.415987 | 12.29874 | H | -0.20718 | 3.00942 | 12.135 |
| H | -1.45672 | 7.707158 | 9.89138 | H | -0.43953 | 5.66796 | 10.148 |
| H | -4.17738 | 5.873351 | 10.59307 | H | -3.17774 | 3.84566 | 10.812 |
| H | 2.357056 | 5.500765 | 7.724659 | H | 3.24859 | 1.18259 | 9.19298 |
| H | 2.424268 | 3.121173 | 10.0437 | H | 2.54386 | 0.61764 | 5.96348 |
| H | 2.119704 | 2.295287 | 6.808555 | H | 2.97068 | 3.74039 | 7.0857 |
| H | -4.93852 | 2.847576 | 7.580045 | H | -4.36009 | 1.31737 | 7.48097 |
| H | -0.56548 | -0.42371 | 5.75644 | H | -0.38592 | -1.8029 | 4.73782 |
| H | -3.80948 | -0.92684 | 5.294989 | H | -3.68137 | -1.94875 | 4.42243 |
| H | -8.6563 | 1.947826 | -8.82987 | H | -6.48451 | 4.55113 | -7.00091 |
| H | -6.22166 | 3.56958 | -7.15843 | H | -6.45088 | 1.77585 | -8.8681 |
| H | -5.76077 | 0.439589 | -8.22715 | H | -9.25851 | 3.52689 | -8.60868 |
| H | -10.1694 | -2.6013 | -3.52751 | H | -10.1721 | -2.265 | -4.40815 |
| H | -10.8709 | -1.88976 | -6.73366 | H | -11.1466 | -0.93683 | -7.33375 |
| H | -7.95224 | -3.27815 | -5.95511 | H | -8.15417 | -2.36646 | -7.09031 |
| H | -9.06039 | 0.808609 | -1.19857 | H | -8.88911 | 0.62685 | -1.51824 |
| H | -3.15838 | 7.475878 | -1.82773 | H | -3.09159 | 7.40367 | -1.31654 |
| H | -8.33285 | 5.364412 | -0.80355 | H | -8.27151 | 5.08464 | -0.32926 |
| H | -6.84989 | 5.539252 | -3.78854 | H | -6.94458 | 5.83288 | -3.29881 |
| H | -6.0685 | 1.360118 | 1.666621 | H | -5.92098 | 0.86907 | 1.37431 |
| H | -5.23693 | 4.225877 | 3.130955 | H | -4.92576 | 3.45048 | 3.21261 |
| H | -0.69066 | 3.152875 | 2.651769 | H | -0.45292 | 2.3667 | 2.35053 |
| H | 2.551943 | 0.26866 | 2.056028 | H | 2.75477 | -0.16334 | 0.80812 |
| H | 6.226617 | 0.174462 | -4.04794 | H | 5.70012 | -0.11879 | -5.72591 |
| H | 8.297859 | 0.762502 | -8.20071 | H | 7.23798 | 0.82281 | -10.0434 |
| H | 5.698669 | 1.540057 | -12.0249 | H | 4.23327 | 2.28291 | -13.3332 |
| H | 1.027138 | 1.722251 | -11.6757 | H | -0.30938 | 2.79171 | -12.2879 |
| H | -1.03255 | 1.202138 | -7.5147 | H | -1.8281 | 1.90908 | -7.95775 |
| H | 1.309685 | -3.7222 | 4.251431 | H | 1.09536 | -5.71363 | 0.01001 |
| H | 3.341546 | -5.72737 | -0.79539 | H | 4.75387 | -2.93898 | 3.56243 |
| H | 1.622653 | -10.6888 | 5.378563 | H | 3.61063 | -9.45314 | 0.7843 |
| H | 1.444323 | -7.44563 | 6.124671 | H | 3.66769 | -11.0184 | 3.74951 |
| H | -0.64156 | -8.74488 | 3.825634 | H | 1.00267 | -9.17611 | 2.87783 |
| H | 6.185521 | -6.9439 | 4.962882 | H | 1.5465 | -6.6155 | 7.01434 |
| H | 7.254687 | -8.02641 | 1.96794 | H | 4.53527 | -5.24247 | 7.70796 |
| H | 6.30011 | -10.2363 | 4.317347 | H | 4.17343 | -8.57645 | 7.79714 |
| H | 3.267462 | -11.7519 | 1.190461 | H | 7.58017 | -7.32211 | 2.28259 |
| **1A-5** | | | | **1A-6** | | | |
| Atom | X | Y | Z | Atom | X | Y | Z |
| C | -2.81872 | 2.162762 | -6.33391 | C | -2.21443 | 4.34582 | -4.944 |
| C | -1.65022 | -4.19241 | -0.6915 | C | -2.48047 | -2.77839 | -0.20536 |
| C | -2.54895 | 4.995769 | 8.633174 | C | -2.83517 | 4.63396 | 10.3675 |
| C | 1.366497 | 3.434159 | 6.376221 | C | -5.69052 | 1.37235 | 8.37154 |
| C | -1.41005 | 3.0912 | 6.861037 | C | -3.12625 | 2.59287 | 8.41159 |
| C | -2.81135 | 1.270533 | 5.806398 | C | -1.22121 | 2.00119 | 6.85798 |
| C | -1.89759 | -0.68702 | 3.957631 | C | -1.1898 | 0.0726 | 4.78352 |
| C | -10.117 | 6.660041 | -3.6509 | C | -7.63321 | 2.23603 | -7.23707 |
| C | -12.8447 | 5.068037 | -0.06986 | C | -9.44724 | -1.67176 | -5.17937 |
| O | -10.7413 | 4.51638 | -1.89913 | O | -8.25507 | 0.88362 | -4.81973 |
| C | -9.55512 | 2.283291 | -1.88142 | C | -7.85968 | 1.80705 | -2.49612 |
| O | -4.37991 | 4.64836 | -1.92662 | O | -2.95847 | 6.52445 | -0.24532 |
| C | -7.39061 | 1.499431 | -3.52748 | C | -6.6783 | 4.28545 | -1.78484 |
| C | -4.77509 | 1.989572 | -2.30442 | C | -3.92826 | 4.05354 | -0.81464 |
| C | -4.59629 | 0.768543 | 0.299391 | C | -3.79364 | 2.55869 | 1.64884 |
| C | -1.89843 | 0.278255 | 1.203755 | C | -1.24038 | 1.30427 | 2.14088 |
| C | -0.55706 | -1.49788 | -0.67013 | C | -0.64053 | -0.53369 | -0.03482 |
| C | -0.69142 | -0.42671 | -3.3503 | C | -0.67605 | 0.87907 | -2.55191 |
| C | -2.7111 | 1.181468 | -4.21957 | C | -2.25018 | 3.05229 | -3.00142 |
| C | 2.348543 | -1.54369 | -0.32392 | C | 2.14202 | -1.44118 | -0.02852 |
| O | 3.304148 | -1.90764 | -2.91697 | O | 2.76277 | -1.66692 | -2.73814 |
| C | 1.561328 | -0.9443 | -4.52466 | C | 1.23967 | -0.05902 | -4.02135 |
| C | 2.455452 | -0.68665 | -7.11738 | C | 1.94681 | 0.28932 | -6.66065 |
| C | 0.796558 | -0.86796 | -9.17172 | C | 0.13002 | 0.84554 | -8.50257 |
| C | 1.717318 | -0.6917 | -11.6197 | C | 0.83944 | 1.09108 | -11.0137 |
| C | 4.287231 | -0.3202 | -12.0513 | C | 3.35956 | 0.79938 | -11.7173 |
| C | 5.949491 | -0.15282 | -10.0181 | C | 5.17383 | 0.23151 | -9.89784 |
| C | 5.04823 | -0.35222 | -7.56422 | C | 4.47582 | -0.04066 | -7.38477 |
| C | 3.490127 | -3.51608 | 1.316322 | C | 2.76921 | -3.88649 | 1.20594 |
| C | 4.960849 | -2.94872 | 3.273721 | C | 4.47275 | -4.07909 | 3.04888 |
| C | 6.024564 | -4.76753 | 5.184894 | C | 5.16168 | -6.42637 | 4.51113 |
| C | 6.061147 | -7.50701 | 4.281668 | C | 7.9918 | -6.92949 | 4.25584 |
| C | 4.495055 | -4.50403 | 7.639264 | C | 3.63388 | -8.75466 | 3.76519 |
| O | 8.57158 | -3.89027 | 5.598553 | O | 4.78149 | -5.87781 | 7.16281 |
| H | -0.61259 | -5.36638 | -2.04404 | H | -1.90729 | -4.0404 | -1.74281 |
| H | -1.54335 | -5.09175 | 1.169209 | H | -2.54001 | -3.877 | 1.54833 |
| H | -3.62636 | -4.17244 | -1.29456 | H | -4.3891 | -2.12266 | -0.64947 |
| H | -4.56944 | 4.669607 | 8.914406 | H | -3.16188 | 3.88304 | 12.2737 |
| H | -1.61299 | 4.936813 | 10.48409 | H | -0.95326 | 5.48394 | 10.3166 |
| H | -2.29699 | 6.921104 | 7.902467 | H | -4.23576 | 6.13656 | 10.075 |
| H | 2.423453 | 3.442578 | 8.161441 | H | -5.85434 | -0.15241 | 6.99231 |
| H | 2.158955 | 1.96481 | 5.163586 | H | -6.16101 | 0.6089 | 10.2417 |
| H | 1.720629 | 5.26785 | 5.472348 | H | -7.14479 | 2.78548 | 7.92989 |
| H | -4.81566 | 1.224616 | 6.276413 | H | 0.52871 | 3.07428 | 7.06025 |
| H | 0.00404 | -1.33434 | 4.460391 | H | 0.53868 | -1.06268 | 4.95908 |
| H | -3.12799 | -2.35249 | 4.084886 | H | -2.77887 | -1.2432 | 4.95623 |
| H | -9.28733 | 8.230533 | -2.57996 | H | -6.58545 | 0.97593 | -8.50933 |
| H | -8.77776 | 6.134987 | -5.12827 | H | -9.38142 | 2.7439 | -8.23453 |
| H | -11.8382 | 7.387069 | -4.55136 | H | -6.50307 | 3.93566 | -6.95689 |
| H | -14.602 | 5.520085 | -1.07607 | H | -9.95848 | -2.54872 | -3.37923 |
| H | -13.2088 | 3.471967 | 1.191086 | H | -11.1573 | -1.53087 | -6.3458 |
| H | -12.3864 | 6.725153 | 1.092243 | H | -8.15331 | -2.9471 | -6.18232 |
| H | -10.1746 | 0.899413 | -0.48582 | H | -8.46618 | 0.62445 | -0.92005 |
| H | -4.12369 | 5.359791 | -3.59266 | H | -2.66136 | 7.33038 | -1.86186 |
| H | -7.40274 | 2.466167 | -5.35492 | H | -7.76713 | 5.18117 | -0.26134 |
| H | -7.5218 | -0.53347 | -3.93986 | H | -6.65777 | 5.59815 | -3.38222 |
| H | -5.65373 | -1.01576 | 0.299139 | H | -5.26852 | 1.10717 | 1.67435 |
| H | -5.5677 | 2.026758 | 1.625734 | H | -4.23101 | 3.87537 | 3.18698 |
| H | -0.88291 | 2.092744 | 1.16581 | H | 0.21722 | 2.78896 | 2.09347 |
| H | 2.989505 | 0.347862 | 0.2629 | H | 3.35121 | 0.08039 | 0.71486 |
| H | -1.20196 | -1.14221 | -8.8339 | H | -1.82373 | 1.08637 | -7.94682 |
| H | 0.420302 | -0.84168 | -13.2028 | H | -0.58154 | 1.51681 | -12.4317 |
| H | 4.99501 | -0.16927 | -13.9725 | H | 3.90776 | 1.00766 | -13.6847 |
| H | 7.955063 | 0.131244 | -10.3471 | H | 7.13905 | -0.00166 | -10.4408 |
| H | 6.335792 | -0.23399 | -5.97382 | H | 5.87938 | -0.4921 | -5.96086 |
| H | 3.056941 | -5.46847 | 0.844132 | H | 1.76462 | -5.54081 | 0.51632 |
| H | 5.412827 | -0.977 | 3.673453 | H | 5.47167 | -2.3882 | 3.6833 |
| H | 4.148897 | -8.23251 | 3.971249 | H | 9.07173 | -5.26685 | 4.84088 |
| H | 7.140155 | -7.67251 | 2.527074 | H | 8.52927 | -8.51625 | 5.46877 |
| H | 6.951377 | -8.70373 | 5.718423 | H | 8.46963 | -7.38219 | 2.2968 |
| H | 4.496407 | -2.53713 | 8.278737 | H | 4.18292 | -10.338 | 4.974 |
| H | 5.323592 | -5.68193 | 9.131304 | H | 3.97699 | -9.28398 | 1.79453 |
| H | 2.535201 | -5.10325 | 7.35567 | H | 1.60313 | -8.42788 | 4.00693 |
| H | 9.289789 | -4.89239 | 6.941419 | H | 3.02477 | -5.4389 | 7.38715 |
| **1A-7** | | | | **1A-8** | | | |
| Atom | X | Y | Z | Atom | X | Y | Z |
| C | -1.59114 | 3.775402 | -5.84106 | C | -1.89428 | 2.97276 | -6.22053 |
| C | -1.91235 | -2.62355 | -0.17308 | C | -1.68287 | -2.57413 | 0.26552 |
| C | -4.28598 | 6.100439 | 8.775071 | C | -3.75689 | 7.23398 | 8.09268 |
| C | -6.26991 | 2.216547 | 6.830392 | C | -5.74977 | 3.09118 | 6.80246 |
| C | -3.92287 | 3.779908 | 7.176528 | C | -3.42668 | 4.72637 | 6.79854 |
| C | -1.68504 | 3.260499 | 6.119664 | C | -1.23875 | 4.10798 | 5.69167 |
| C | -1.04864 | 1.08772 | 4.4125 | C | -0.64438 | 1.73779 | 4.2527 |
| C | -10.2424 | -0.20321 | -0.01788 | C | -10.0944 | -0.30972 | 0.64708 |
| C | -12.274 | 3.919444 | 1.249129 | C | -12.1325 | 3.92725 | 1.43454 |
| O | -10.2234 | 2.628778 | -0.2382 | O | -10.1427 | 2.46226 | 0.02785 |
| C | -8.5649 | 3.97318 | -1.59582 | C | -8.59125 | 3.61954 | -1.60145 |
| O | -3.51702 | 6.398565 | -1.88569 | O | -3.66018 | 6.08019 | -2.56058 |
| C | -6.40387 | 3.011755 | -3.14149 | C | -6.50192 | 2.47583 | -3.12082 |
| C | -3.80842 | 3.707486 | -1.95237 | C | -3.86182 | 3.4014 | -2.22297 |
| C | -3.65294 | 2.764377 | 0.776591 | C | -3.50456 | 2.86326 | 0.59691 |
| C | -1.04558 | 1.842549 | 1.594825 | C | -0.83042 | 2.10518 | 1.365 |
| C | -0.18807 | -0.28455 | -0.20356 | C | -0.02427 | -0.23046 | -0.18102 |
| C | -0.06064 | 0.757937 | -2.88936 | C | -0.07998 | 0.4428 | -2.98386 |
| C | -1.7413 | 2.711655 | -3.76889 | C | -1.87804 | 2.20952 | -4.0147 |
| C | 2.622614 | -1.05909 | 0.168381 | C | 2.82354 | -0.89447 | 0.12462 |
| O | 3.566411 | -1.46348 | -2.39619 | O | 3.64203 | -1.59895 | -2.41739 |
| C | 2.080307 | -0.15842 | -4.01842 | C | 2.02723 | -0.55729 | -4.1059 |
| C | 3.0688 | -0.04512 | -6.58822 | C | 2.87818 | -0.74817 | -6.72042 |
| C | 1.454223 | 0.139755 | -8.67684 | C | 1.15397 | -0.8893 | -8.72285 |
| C | 2.445962 | 0.161633 | -11.1033 | C | 2.02022 | -1.14806 | -11.1835 |
| C | 5.046905 | 0.014025 | -11.4802 | C | 4.60273 | -1.25501 | -11.6816 |
| C | 6.662797 | -0.18335 | -9.41289 | C | 6.32784 | -1.12912 | -9.69839 |
| C | 5.684841 | -0.23052 | -6.98036 | C | 5.47675 | -0.89497 | -7.22929 |
| C | 3.159892 | -3.32481 | 1.748833 | C | 3.49689 | -2.93248 | 1.94471 |
| C | 4.124335 | -5.48011 | 0.895978 | C | 4.46479 | -5.16255 | 1.31853 |
| C | 4.64195 | -7.76945 | 2.498791 | C | 5.10708 | -7.22542 | 3.16555 |
| C | 7.479832 | -8.36339 | 2.453604 | C | 7.94659 | -7.79901 | 3.03342 |
| C | 3.109657 | -10.0138 | 1.485186 | C | 3.55522 | -9.59248 | 2.53217 |
| O | 3.860254 | -7.19147 | 5.029366 | O | 4.45922 | -6.33708 | 5.64374 |
| H | -1.13936 | -4.07134 | -1.43223 | H | -3.62844 | -2.22869 | -0.33893 |
| H | -2.06612 | -3.4544 | 1.715197 | H | -0.95234 | -4.1594 | -0.84438 |
| H | -3.80721 | -2.15229 | -0.84973 | H | -1.69659 | -3.15616 | 2.25081 |
| H | -5.77059 | 7.311611 | 7.977988 | H | -4.24479 | 6.97558 | 10.0926 |
| H | -4.90057 | 5.591152 | 10.68992 | H | -2.04666 | 8.38734 | 7.98267 |
| H | -2.55167 | 7.213205 | 8.921896 | H | -5.3172 | 8.29938 | 7.2345 |
| H | -5.91352 | 0.398221 | 5.923913 | H | -6.55569 | 2.971 | 8.70875 |
| H | -7.19304 | 1.868466 | 8.653565 | H | -7.20825 | 3.93079 | 5.58472 |
| H | -7.63606 | 3.245015 | 5.650899 | H | -5.40234 | 1.17521 | 6.12132 |
| H | -0.14661 | 4.591375 | 6.45605 | H | 0.28249 | 5.49804 | 5.756 |
| H | 0.835402 | 0.386087 | 4.924015 | H | 1.28677 | 1.15029 | 4.73267 |
| H | -2.36051 | -0.48801 | 4.696913 | H | -1.89001 | 0.18796 | 4.82831 |
| H | -12.0915 | -0.96478 | -0.56937 | H | -8.69125 | -1.37154 | -0.4297 |
| H | -8.80175 | -1.12834 | -1.16872 | H | -9.69372 | -0.58355 | 2.66609 |
| H | -9.94172 | -0.76799 | 1.957832 | H | -11.9516 | -1.1656 | 0.29728 |
| H | -14.1503 | 3.315784 | 0.599989 | H | -12.0675 | 5.94136 | 0.98015 |
| H | -12.1591 | 3.414054 | 3.259637 | H | -14.0303 | 3.21777 | 0.98532 |
| H | -12.161 | 5.975695 | 1.087289 | H | -11.8969 | 3.71155 | 3.48601 |
| H | -8.73297 | 6.024387 | -1.54746 | H | -8.79834 | 5.6545 | -1.83381 |
| H | -3.09352 | 6.883129 | -3.60203 | H | -3.36548 | 6.31963 | -4.354 |
| H | -6.43685 | 3.859496 | -5.03516 | H | -6.68052 | 3.0199 | -5.11584 |
| H | -6.48767 | 0.957197 | -3.40326 | H | -6.53803 | 0.40468 | -3.05812 |
| H | -5.03138 | 1.245905 | 1.067312 | H | -4.82458 | 1.38158 | 1.19273 |
| H | -4.26435 | 4.318678 | 1.993798 | H | -4.07363 | 4.56552 | 1.62147 |
| H | 0.300931 | 3.412336 | 1.3504 | H | 0.45977 | 3.64992 | 0.82968 |
| H | 3.665333 | 0.587551 | 0.913486 | H | 3.8607 | 0.85222 | 0.60079 |
| H | -0.56611 | 0.267574 | -8.38366 | H | -0.85204 | -0.79058 | -8.33605 |
| H | 1.180416 | 0.298285 | -12.7128 | H | 0.67061 | -1.26295 | -12.7249 |
| H | 5.812794 | 0.046638 | -13.3846 | H | 5.26996 | -1.44225 | -13.614 |
| H | 8.691621 | -0.30142 | -9.69813 | H | 8.34282 | -1.21414 | -10.078 |
| H | 6.935591 | -0.39068 | -5.36431 | H | 6.81253 | -0.80257 | -5.67727 |
| H | 2.71158 | -3.14604 | 3.744884 | H | 3.14428 | -2.50986 | 3.92316 |
| H | 4.597616 | -5.68152 | -1.09528 | H | 4.8407 | -5.60739 | -0.65389 |
| H | 8.136979 | -8.70304 | 0.521132 | H | 8.49689 | -8.38666 | 1.12732 |
| H | 8.543391 | -6.7782 | 3.24537 | H | 9.0301 | -6.11603 | 3.54742 |
| H | 7.880037 | -10.0649 | 3.568923 | H | 8.4339 | -9.33546 | 4.33766 |
| H | 3.606176 | -10.4261 | -0.48089 | H | 3.9463 | -10.2442 | 0.60755 |
| H | 1.089549 | -9.58783 | 1.583905 | H | 1.5373 | -9.17546 | 2.69438 |
| H | 3.491246 | -11.7137 | 2.608733 | H | 4.02832 | -11.1342 | 3.83486 |
| H | 4.235441 | -8.64089 | 6.065997 | H | 4.89317 | -7.64911 | 6.82964 |
| **1A-9** | | | |
| Atom | X | Y | Z |
| C | -2.21451 | 4.456956 | -4.88954 |
| C | -2.94694 | -2.92966 | -0.5986 |
| C | -1.84461 | 4.14835 | 10.44094 |
| C | 1.66501 | 1.966192 | 8.040434 |
| C | -1.1275 | 2.393499 | 8.325569 |
| C | -2.88371 | 1.346398 | 6.837948 |
| C | -2.4136 | -0.38876 | 4.638185 |
| C | -7.28449 | 2.446385 | -7.95812 |
| C | -9.28061 | -1.58652 | -6.36122 |
| O | -8.18105 | 0.95415 | -5.71584 |
| C | -8.084 | 1.745363 | -3.31297 |
| O | -3.57698 | 6.382104 | -0.22522 |
| C | -7.04011 | 4.191285 | -2.32848 |
| C | -4.42899 | 3.934261 | -1.03245 |
| C | -4.58766 | 2.318393 | 1.35309 |
| C | -2.11375 | 1.029466 | 2.105494 |
| C | -1.20089 | -0.66355 | -0.07759 |
| C | -0.94933 | 0.874838 | -2.50764 |
| C | -2.47784 | 3.060998 | -3.03757 |
| C | 1.581381 | -1.51324 | 0.22592 |
| O | 2.503907 | -1.6555 | -2.40288 |
| C | 1.131834 | 0.007322 | -3.78502 |
| C | 2.14014 | 0.4719 | -6.30545 |
| C | 0.551138 | 1.128595 | -8.31737 |
| C | 1.548239 | 1.484516 | -10.7146 |
| C | 4.13203 | 1.204246 | -11.1352 |
| C | 5.721171 | 0.537382 | -9.14672 |
| C | 4.735233 | 0.154927 | -6.7473 |
| C | 2.121331 | -3.96025 | 1.49423 |
| C | 3.716473 | -4.14274 | 3.433401 |
| C | 4.27108 | -6.45951 | 4.996323 |
| C | 7.100505 | -7.01975 | 4.942742 |
| C | 2.745544 | -8.77909 | 4.221174 |
| O | 3.72939 | -5.82077 | 7.601074 |
| H | -2.15671 | -4.08944 | -2.12025 |
| H | -3.1949 | -4.12502 | 1.072064 |
| H | -4.80191 | -2.27904 | -1.2359 |
| H | -1.14187 | 3.438611 | 12.25939 |
| H | -0.99048 | 6.018721 | 10.16495 |
| H | -3.89193 | 4.385948 | 10.58106 |
| H | 2.133035 | 0.644691 | 6.526973 |
| H | 2.627983 | 3.760314 | 7.642539 |
| H | 2.478642 | 1.236948 | 9.803518 |
| H | -4.85136 | 1.821119 | 7.217865 |
| H | -0.72833 | -1.54419 | 4.968519 |
| H | -4.00471 | -1.71189 | 4.480119 |
| H | -6.06112 | 1.270586 | -9.15177 |
| H | -8.90009 | 2.989944 | -9.14286 |
| H | -6.22959 | 4.140409 | -7.44565 |
| H | -7.85316 | -2.79149 | -7.26507 |
| H | -9.99582 | -2.56811 | -4.68923 |
| H | -10.8361 | -1.39373 | -7.72049 |
| H | -8.85688 | 0.468382 | -1.89097 |
| H | -3.09984 | 7.272611 | -1.75181 |
| H | -8.32481 | 4.993928 | -0.90923 |
| H | -6.84054 | 5.588215 | -3.83949 |
| H | -6.05253 | 0.872291 | 1.128724 |
| H | -5.21578 | 3.570627 | 2.878292 |
| H | -0.67871 | 2.511773 | 2.375261 |
| H | 2.662537 | 0.021461 | 1.125596 |
| H | -1.45261 | 1.36309 | -7.97998 |
| H | 0.302755 | 1.988361 | -12.2658 |
| H | 4.90521 | 1.499094 | -13.0139 |
| H | 7.735405 | 0.312606 | -9.46858 |
| H | 5.96259 | -0.37398 | -5.19327 |
| H | 1.136794 | -5.61353 | 0.774074 |
| H | 4.705515 | -2.45423 | 4.088837 |
| H | 8.171756 | -5.35972 | 5.550781 |
| H | 7.52661 | -8.57935 | 6.232566 |
| H | 7.697259 | -7.53769 | 3.033085 |
| H | 3.181678 | -10.3369 | 5.506729 |
| H | 3.201551 | -9.3726 | 2.292065 |
| H | 0.711224 | -8.40423 | 4.322479 |
| H | 1.968148 | -5.35269 | 7.69989 |

**Table S4.** Cartesian coordinates for the low-energy reoptimized random research conformers of **1B** at B3LYP-D3(BJ)/6-31G* level of theory in chloroform.

| **1B-1** | | | | **1B-2** | | | |
| --- | --- | --- | --- | --- | --- | --- | --- |
| Atom | X | Y | Z | Atom | X | Y | Z |
| C | 10.41592 | -0.06227 | -5.48721 | C | 9.80599 | -1.22047 | -6.24274 |
| C | 12.39582 | -4.51364 | 0.639736 | C | 12.2869 | -5.30009 | 0.32197 |
| C | 6.346842 | -3.1201 | 7.982868 | C | 9.62826 | 4.6763 | 8.30835 |
| C | 7.768915 | 1.423621 | 7.742606 | C | 13.7733 | 3.46838 | 6.25009 |
| C | 8.354665 | -1.28989 | 7.146279 | C | 11.0758 | 2.76399 | 6.78731 |
| C | 10.43205 | -2.10545 | 5.953358 | C | 9.99661 | 0.6208 | 5.99009 |
| C | 12.6011 | -0.53541 | 5.009094 | C | 11.2414 | -1.39567 | 4.41388 |
| C | 7.695639 | 8.450522 | -3.99192 | C | 13.3783 | 4.52849 | -5.66129 |
| C | 10.17086 | 8.069244 | -8.05957 | C | 15.0911 | 6.37903 | -1.60639 |
| O | 9.872105 | 7.243599 | -5.35681 | O | 12.9428 | 5.04966 | -2.90688 |
| C | 11.46335 | 5.561742 | -4.33888 | C | 10.8524 | 4.4096 | -1.63096 |
| O | 7.435247 | 2.270041 | -2.2077 | O | 5.92592 | -0.60573 | -3.08072 |
| C | 11.40057 | 4.528224 | -1.70744 | C | 8.56148 | 3.08203 | -2.63944 |
| C | 9.988002 | 1.936241 | -1.48932 | C | 8.36692 | 0.15762 | -2.23619 |
| C | 10.0181 | 0.961284 | 1.228333 | C | 8.57428 | -0.62256 | 0.53392 |
| C | 12.62685 | 0.115639 | 2.140865 | C | 11.2697 | -0.72587 | 1.57401 |
| C | 13.71953 | -1.93932 | 0.383118 | C | 12.932 | -2.48752 | -0.03728 |
| C | 13.52895 | -1.13751 | -2.38987 | C | 12.6984 | -1.87933 | -2.85335 |
| C | 11.28375 | 0.111078 | -3.33684 | C | 10.3463 | -1.08893 | -3.97688 |
| C | 16.62988 | -2.19362 | 0.640862 | C | 15.7876 | -1.97307 | 0.33879 |
| O | 17.51791 | -2.63065 | -1.95171 | O | 16.8877 | -2.43959 | -2.17233 |
| C | 15.72789 | -1.75523 | -3.58139 | C | 15.0431 | -2.09227 | -3.92425 |
| C | 16.61483 | -1.55272 | -6.18927 | C | 16.0029 | -1.91702 | -6.50494 |
| C | 19.20616 | -1.17286 | -6.62637 | C | 18.4637 | -0.99315 | -6.88631 |
| C | 20.12141 | -0.97287 | -9.07347 | C | 19.442 | -0.79481 | -9.30829 |
| C | 18.47828 | -1.17205 | -11.121 | C | 18.0008 | -1.54556 | -11.3805 |
| C | 15.9151 | -1.58069 | -10.7016 | C | 15.5801 | -2.50343 | -11.0117 |
| C | 14.97573 | -1.77703 | -8.25747 | C | 14.5744 | -2.69243 | -8.59422 |
| C | 17.63308 | -4.25186 | 2.269875 | C | 17.2006 | -3.51646 | 2.21296 |
| C | 19.00239 | -3.82677 | 4.334978 | C | 18.4908 | -2.48986 | 4.11485 |
| C | 19.95056 | -5.84465 | 6.117489 | C | 19.9118 | -3.86373 | 6.16626 |
| C | 18.41284 | -5.75371 | 8.563326 | C | 20 | -6.72394 | 5.8081 |
| C | 22.76722 | -5.42896 | 6.657019 | C | 18.7693 | -3.181 | 8.72854 |
| O | 19.55508 | -8.32473 | 5.110259 | O | 22.4514 | -2.85895 | 6.23743 |
| H | 13.10751 | -5.82102 | -0.79834 | H | 10.3245 | -5.66276 | -0.21739 |
| H | 12.72439 | -5.35794 | 2.496769 | H | 13.4874 | -6.46942 | -0.89237 |
| H | 10.35762 | -4.31982 | 0.355376 | H | 12.5515 | -5.90537 | 2.28271 |
| H | 6.825856 | -5.06792 | 7.488247 | H | 10.5424 | 5.00806 | 10.1411 |
| H | 6.063499 | -3.01835 | 10.03555 | H | 9.57644 | 6.50765 | 7.33401 |
| H | 4.522173 | -2.65645 | 7.110555 | H | 7.68374 | 4.06963 | 8.65248 |
| H | 6.094856 | 2.042442 | 6.683936 | H | 14.8098 | 3.79183 | 8.01809 |
| H | 7.306734 | 1.636202 | 9.753168 | H | 14.7788 | 2.02991 | 5.16557 |
| H | 9.310367 | 2.719213 | 7.295757 | H | 13.8597 | 5.24838 | 5.18629 |
| H | 10.57408 | -4.13456 | 5.646843 | H | 8.01935 | 0.31509 | 6.47691 |
| H | 12.63479 | 1.268152 | 6.024329 | H | 13.184 | -1.726 | 5.05616 |
| H | 14.37943 | -1.49552 | 5.478162 | H | 10.2249 | -3.18206 | 4.68411 |
| H | 5.926892 | 8.07151 | -5.00653 | H | 13.6399 | 6.31022 | -6.69277 |
| H | 7.458142 | 7.75987 | -2.0654 | H | 15.1238 | 3.4393 | -5.92301 |
| H | 7.92184 | 10.51321 | -3.94999 | H | 11.8507 | 3.46986 | -6.55412 |
| H | 11.78411 | 7.154635 | -8.96956 | H | 14.6739 | 6.76052 | 0.38177 |
| H | 8.466349 | 7.621698 | -9.15454 | H | 16.8233 | 5.23724 | -1.70342 |
| H | 10.42636 | 10.12515 | -8.17944 | H | 15.5261 | 8.18039 | -2.53924 |
| H | 12.93964 | 4.796753 | -5.55616 | H | 10.7689 | 4.93035 | 0.36256 |
| H | 7.420872 | 2.081405 | -4.03431 | H | 6.05821 | -0.71636 | -4.90664 |
| H | 10.42722 | 5.805299 | -0.4007 | H | 8.34274 | 3.40156 | -4.67368 |
| H | 13.34583 | 4.30881 | -1.02491 | H | 6.86583 | 3.84456 | -1.72211 |
| H | 8.664459 | -0.5992 | 1.370076 | H | 7.67203 | -2.48036 | 0.72684 |
| H | 9.292451 | 2.458045 | 2.468502 | H | 7.44519 | 0.69165 | 1.67332 |
| H | 13.91654 | 1.734621 | 1.958732 | H | 12.1013 | 1.16398 | 1.4041 |
| H | 17.42432 | -0.3591 | 1.223173 | H | 16.0589 | 0.05721 | 0.71582 |
| H | 20.48216 | -1.0202 | -5.0299 | H | 19.5812 | -0.41528 | -5.26802 |
| H | 22.12548 | -0.66056 | -9.38765 | H | 21.3357 | -0.05335 | -9.58434 |
| H | 19.19886 | -1.02172 | -13.0375 | H | 18.7707 | -1.39369 | -13.2775 |
| H | 14.62922 | -1.762 | -12.2909 | H | 14.4605 | -3.11413 | -12.6195 |
| H | 12.98205 | -2.09527 | -7.92779 | H | 12.6875 | -3.42343 | -8.30505 |
| H | 17.15948 | -6.17894 | 1.734586 | H | 17.123 | -5.55507 | 1.96602 |
| H | 19.46314 | -1.88707 | 4.868639 | H | 18.5711 | -0.4351 | 4.28881 |
| H | 16.42542 | -6.15142 | 8.157219 | H | 21.1461 | -7.55632 | 7.31238 |
| H | 19.12119 | -7.17174 | 9.892064 | H | 18.1081 | -7.55822 | 5.88949 |
| H | 18.54779 | -3.88759 | 9.443688 | H | 20.8525 | -7.22357 | 3.98826 |
| H | 23.86747 | -5.51911 | 4.904116 | H | 16.8273 | -3.87713 | 8.8646 |
| H | 23.45148 | -6.89077 | 7.949233 | H | 18.7609 | -1.12947 | 8.98552 |
| H | 23.09585 | -3.57542 | 7.516877 | H | 19.8982 | -4.02404 | 10.2428 |
| H | 20.58639 | -8.48679 | 3.613892 | H | 23.1982 | -3.14841 | 4.59862 |
| **1B-3** | | | | **1B-4** | | | |
| Atom | X | Y | Z | Atom | X | Y | Z |
| C | 10.35084 | -0.3284 | -5.56797 | C | 8.5937 | -0.96835 | -5.97614 |
| C | 12.47612 | -4.73529 | 0.556716 | C | 11.2962 | -5.12373 | 0.46576 |
| C | 6.105436 | -3.61612 | 7.762825 | C | 11.0587 | 5.34008 | 8.23874 |
| C | 7.437602 | 0.960207 | 7.672579 | C | 14.6793 | 3.36904 | 5.80931 |
| C | 8.088587 | -1.72312 | 7.011671 | C | 11.9788 | 3.16472 | 6.662 |
| C | 10.19982 | -2.46183 | 5.827962 | C | 10.4672 | 1.2229 | 6.07954 |
| C | 12.34718 | -0.82025 | 4.959357 | C | 11.1706 | -1.01258 | 4.46544 |
| C | 7.259538 | 8.055174 | -4.0617 | C | 12.9302 | 4.23866 | -6.029 |
| C | 9.836173 | 7.813541 | -8.07642 | C | 15.2935 | 6.00499 | -2.27396 |
| O | 9.514251 | 6.952464 | -5.38749 | O | 12.8655 | 4.92163 | -3.27613 |
| C | 11.15184 | 5.32929 | -4.34825 | C | 10.8605 | 4.61561 | -1.76138 |
| O | 7.217054 | 1.853875 | -2.33128 | O | 5.2066 | 0.24803 | -2.44749 |
| C | 11.07517 | 4.269079 | -1.72776 | C | 8.32182 | 3.56892 | -2.45912 |
| C | 9.766633 | 1.619898 | -1.56504 | C | 7.80381 | 0.71029 | -1.90468 |
| C | 9.784683 | 0.613888 | 1.141058 | C | 8.21769 | -0.00715 | 0.85886 |
| C | 12.4106 | -0.11759 | 2.102862 | C | 10.9777 | -0.42428 | 1.61198 |
| C | 13.65027 | -2.0867 | 0.343696 | C | 12.2228 | -2.42447 | -0.09086 |
| C | 13.46338 | -1.27321 | -2.42672 | C | 11.7437 | -1.89571 | -2.89268 |
| C | 11.17542 | -0.12951 | -3.40297 | C | 9.4 | -0.83919 | -3.79033 |
| C | 16.56594 | -2.17764 | 0.649895 | C | 15.1401 | -2.24557 | -0.06296 |
| O | 17.51786 | -2.55318 | -1.92805 | O | 15.8718 | -3.00004 | -2.63952 |
| C | 15.71335 | -1.76321 | -3.58344 | C | 13.9008 | -2.47584 | -4.1995 |
| C | 16.63433 | -1.49154 | -6.17297 | C | 14.5705 | -2.55217 | -6.87456 |
| C | 19.2121 | -0.98526 | -6.55954 | C | 17.0781 | -2.02425 | -7.57381 |
| C | 20.15863 | -0.71521 | -8.98782 | C | 17.789 | -2.08127 | -10.0944 |
| C | 18.56222 | -0.96803 | -11.0662 | C | 16.0258 | -2.69817 | -11.9507 |
| C | 16.0143 | -1.50168 | -10.6969 | C | 13.5511 | -3.26509 | -11.2666 |
| C | 15.04337 | -1.76979 | -8.2722 | C | 12.8142 | -3.19546 | -8.74815 |
| C | 17.67142 | -4.17807 | 2.287184 | C | 16.5744 | -3.81497 | 1.77248 |
| C | 18.99217 | -3.66896 | 4.364861 | C | 18.2994 | -2.83247 | 3.32146 |
| C | 20.0744 | -5.61275 | 6.152536 | C | 19.739 | -4.18528 | 5.37458 |
| C | 19.17806 | -5.03895 | 8.849498 | C | 22.5743 | -3.93965 | 4.90287 |
| C | 22.95948 | -5.56864 | 5.99967 | C | 18.9834 | -6.94895 | 5.69616 |
| O | 19.35277 | -8.12436 | 5.452552 | O | 19.2914 | -2.8241 | 7.70517 |
| H | 13.30978 | -5.9936 | -0.85893 | H | 9.2616 | -5.26885 | 0.12836 |
| H | 12.80183 | -5.55833 | 2.424655 | H | 12.2287 | -6.47298 | -0.79678 |
| H | 10.43894 | -4.66229 | 0.214432 | H | 11.6747 | -5.68669 | 2.42056 |
| H | 5.785553 | -3.57738 | 9.812276 | H | 12.2105 | 5.54873 | 9.95153 |
| H | 4.286502 | -3.16551 | 6.872197 | H | 11.2224 | 7.12661 | 7.19606 |
| H | 6.632305 | -5.53944 | 7.22278 | H | 9.0869 | 5.09794 | 8.80519 |
| H | 5.748268 | 1.560304 | 6.627674 | H | 14.9381 | 5.05235 | 4.62268 |
| H | 6.970473 | 1.115382 | 9.687255 | H | 15.9473 | 3.59687 | 7.43535 |
| H | 8.945885 | 2.30335 | 7.255111 | H | 15.3087 | 1.72606 | 4.73204 |
| H | 10.38104 | -4.47744 | 5.456303 | H | 8.53307 | 1.27546 | 6.78379 |
| H | 12.31384 | 0.963489 | 6.008459 | H | 13.0967 | -1.6477 | 4.89394 |
| H | 14.14338 | -1.7376 | 5.446305 | H | 9.91434 | -2.59718 | 4.9231 |
| H | 7.398117 | 10.12505 | -3.9983 | H | 13.2988 | 5.92811 | -7.17666 |
| H | 5.531074 | 7.611824 | -5.11876 | H | 14.487 | 2.92174 | -6.41081 |
| H | 7.008514 | 7.338362 | -2.14647 | H | 11.1946 | 3.35252 | -6.70267 |
| H | 8.176748 | 7.302282 | -9.21205 | H | 15.1476 | 6.50429 | -0.27347 |
| H | 10.005 | 9.879476 | -8.17488 | H | 16.8484 | 4.6464 | -2.49747 |
| H | 11.50687 | 6.977272 | -8.95768 | H | 15.8444 | 7.70263 | -3.33185 |
| H | 12.68568 | 4.63747 | -5.53812 | H | 11.062 | 5.21771 | 0.20066 |
| H | 7.245926 | 1.67425 | -4.15872 | H | 5.11787 | 0.06878 | -4.2707 |
| H | 10.02471 | 5.492781 | -0.42977 | H | 7.92174 | 3.84774 | -4.47157 |
| H | 13.01294 | 4.118936 | -1.00699 | H | 6.84868 | 4.57726 | -1.40512 |
| H | 8.501787 | -1.00929 | 1.236262 | H | 7.12278 | -1.73031 | 1.22645 |
| H | 8.963674 | 2.059199 | 2.383215 | H | 7.39187 | 1.47191 | 2.05536 |
| H | 13.61982 | 1.567333 | 1.980653 | H | 12.0148 | 1.33881 | 1.28438 |
| H | 17.24222 | -0.30125 | 1.248871 | H | 15.6839 | -0.24268 | 0.11819 |
| H | 20.45219 | -0.79288 | -4.93937 | H | 18.4471 | -1.55232 | -6.1231 |
| H | 22.15109 | -0.30701 | -9.26311 | H | 19.7253 | -1.64531 | -10.6164 |
| H | 19.30801 | -0.76273 | -12.9679 | H | 16.5861 | -2.74719 | -13.9251 |
| H | 14.76619 | -1.72583 | -12.3107 | H | 12.1775 | -3.77123 | -12.7047 |
| H | 13.06185 | -2.18643 | -7.98146 | H | 10.8874 | -3.62136 | -8.21494 |
| H | 17.37748 | -6.12878 | 1.709303 | H | 16.1444 | -5.82408 | 1.8038 |
| H | 19.33753 | -1.70309 | 4.890578 | H | 18.7208 | -0.81549 | 3.21393 |
| H | 17.10912 | -5.10063 | 8.961404 | H | 23.6176 | -4.75675 | 6.49078 |
| H | 19.9577 | -6.44005 | 10.15548 | H | 23.1034 | -4.92536 | 3.16514 |
| H | 19.78658 | -3.15605 | 9.455529 | H | 23.1041 | -1.94895 | 4.7305 |
| H | 23.568 | -6.07772 | 4.091924 | H | 19.4075 | -8.05032 | 3.99664 |
| H | 23.75285 | -6.927 | 7.342125 | H | 16.9596 | -7.1286 | 6.09844 |
| H | 23.68372 | -3.68473 | 6.447658 | H | 20.0341 | -7.75858 | 7.28044 |
| H | 17.5523 | -8.27757 | 5.703124 | H | 17.4951 | -2.85652 | 8.02554 |
| **1B-5** | | | | **1B-6** | | | |
| Atom | X | Y | Z | Atom | X | Y | Z |
| C | 10.70818 | 0.19957 | -6.04213 | C | 9.41802 | -3.14742 | -6.16954 |
| C | 11.86973 | -4.1189 | 0.436003 | C | 11.9444 | -4.50732 | 1.16096 |
| C | 7.501265 | -4.01385 | 8.63898 | C | 16.4533 | 3.66617 | 7.81602 |
| C | 11.45722 | -1.7087 | 10.03608 | C | 13.4342 | 0.06359 | 8.87573 |
| C | 9.704127 | -2.37839 | 7.90493 | C | 14.3349 | 1.98259 | 6.96619 |
| C | 10.00353 | -1.55964 | 5.532372 | C | 13.3018 | 2.20464 | 4.67212 |
| C | 12.11167 | 0.120821 | 4.604594 | C | 11.0751 | 0.76154 | 3.67463 |
| C | 8.024877 | 8.851943 | -4.84569 | C | 10.9853 | 7.29177 | -1.62633 |
| C | 10.65262 | 8.340667 | -8.8023 | C | 6.42397 | 8.61057 | -1.19626 |
| O | 10.21411 | 7.558025 | -6.10591 | O | 8.26362 | 6.65756 | -2.13782 |
| C | 11.70679 | 5.838797 | -5.00282 | C | 7.44381 | 4.57297 | -3.31471 |
| O | 7.515825 | 2.690331 | -3.07852 | O | 5.73059 | -0.76921 | -3.72695 |
| C | 11.50221 | 4.857484 | -2.35826 | C | 8.98406 | 2.43282 | -4.3202 |
| C | 9.994644 | 2.318801 | -2.15063 | C | 8.24819 | -0.14128 | -3.05845 |
| C | 9.774622 | 1.460811 | 0.599567 | C | 8.41034 | -0.01695 | -0.18589 |
| C | 12.28119 | 0.563614 | 1.729346 | C | 11.1169 | 0.26597 | 0.80674 |
| C | 13.37217 | -1.64537 | 0.167839 | C | 12.7433 | -2.00415 | -0.08768 |
| C | 13.44868 | -0.96255 | -2.64066 | C | 12.4903 | -2.32795 | -2.95173 |
| C | 11.37261 | 0.377677 | -3.8206 | C | 10.0728 | -2.08162 | -4.20631 |
| C | 16.22857 | -2.06274 | 0.680041 | C | 15.6432 | -1.54953 | 0.16626 |
| O | 17.30169 | -2.63879 | -1.81665 | O | 16.7154 | -2.37441 | -2.27279 |
| C | 15.69779 | -1.75049 | -3.62094 | C | 14.8273 | -2.66319 | -3.97926 |
| C | 16.7964 | -1.72739 | -6.15528 | C | 15.7293 | -3.12801 | -6.55024 |
| C | 19.42946 | -1.49213 | -6.4 | C | 14.3676 | -4.60808 | -8.27124 |
| C | 20.5421 | -1.47328 | -8.77232 | C | 15.3201 | -5.02295 | -10.6828 |
| C | 19.05599 | -1.71453 | -10.9325 | C | 17.6233 | -3.98255 | -11.4069 |
| C | 16.45075 | -1.98147 | -10.7012 | C | 18.9982 | -2.52934 | -9.69504 |
| C | 15.31448 | -1.99329 | -8.33392 | C | 18.0717 | -2.11483 | -7.27942 |
| C | 16.96599 | -4.1358 | 2.428734 | C | 17.069 | -2.98198 | 2.13195 |
| C | 18.24743 | -3.7655 | 4.559049 | C | 18.5912 | -1.90489 | 3.8181 |
| C | 18.94638 | -5.82172 | 6.414202 | C | 20.1676 | -3.2502 | 5.77693 |
| C | 17.22302 | -5.64265 | 8.726628 | C | 22.933 | -2.48708 | 5.46674 |
| C | 21.72883 | -5.5542 | 7.176025 | C | 19.8857 | -6.11994 | 5.73636 |
| O | 18.50137 | -8.28276 | 5.380747 | O | 19.4471 | -2.29879 | 8.24022 |
| H | 11.86352 | -4.80181 | 2.388137 | H | 9.93782 | -4.88267 | 0.84285 |
| H | 9.91187 | -3.85559 | -0.17519 | H | 12.9956 | -6.08303 | 0.33057 |
| H | 12.6942 | -5.57861 | -0.77774 | H | 12.3023 | -4.4986 | 3.19946 |
| H | 8.153189 | -5.83218 | 9.396845 | H | 16.9631 | 5.04601 | 6.36333 |
| H | 6.379952 | -3.11689 | 10.13692 | H | 15.9253 | 4.70217 | 9.53511 |
| H | 6.255945 | -4.38995 | 7.033827 | H | 18.1296 | 2.53709 | 8.28727 |
| H | 12.05993 | -3.40855 | 11.05954 | H | 14.7764 | -0.11651 | 10.4405 |
| H | 13.14348 | -0.69932 | 9.405741 | H | 11.6147 | 0.61688 | 9.70277 |
| H | 10.47538 | -0.50823 | 11.41584 | H | 13.1543 | -1.80556 | 8.02196 |
| H | 8.567665 | -2.10326 | 4.166334 | H | 14.0339 | 3.67837 | 3.42608 |
| H | 11.90643 | 1.997835 | 5.477491 | H | 10.7833 | -1.00312 | 4.71853 |
| H | 13.93991 | -0.60805 | 5.263562 | H | 9.37345 | 1.90044 | 4.05351 |
| H | 6.289664 | 8.528732 | -5.93458 | H | 11.4446 | 9.16288 | -2.3946 |
| H | 7.679068 | 8.184496 | -2.92761 | H | 11.3262 | 7.39965 | 0.41911 |
| H | 8.323056 | 10.90555 | -4.80574 | H | 12.3156 | 5.93532 | -2.43029 |
| H | 12.26842 | 7.359301 | -9.63489 | H | 6.77164 | 10.4485 | -2.09417 |
| H | 8.979769 | 7.941848 | -9.96288 | H | 4.4672 | 8.06437 | -1.56939 |
| H | 10.9866 | 10.38473 | -8.93137 | H | 6.64239 | 8.90289 | 0.84689 |
| H | 13.20694 | 5.011521 | -6.14649 | H | 5.41079 | 4.3385 | -3.54792 |
| H | 7.649934 | 2.504594 | -4.90002 | H | 5.87605 | -1.71413 | -5.29545 |
| H | 10.52236 | 6.195454 | -1.11905 | H | 11.0148 | 2.76555 | -4.1105 |
| H | 13.40989 | 4.589797 | -1.59123 | H | 8.62891 | 2.18204 | -6.35098 |
| H | 8.347438 | -0.03654 | 0.659469 | H | 7.50589 | -1.70835 | 0.59778 |
| H | 9.015967 | 3.029885 | 1.727027 | H | 7.28073 | 1.60246 | 0.44235 |
| H | 13.6439 | 2.115804 | 1.500843 | H | 11.9335 | 1.95984 | -0.08157 |
| H | 17.08918 | -0.26419 | 1.280097 | H | 16.0407 | 0.48269 | 0.31724 |
| H | 20.58248 | -1.30856 | -4.71558 | H | 12.5752 | -5.41438 | -7.70642 |
| H | 22.57762 | -1.27059 | -8.94005 | H | 14.2533 | -6.17766 | -12.002 |
| H | 19.93105 | -1.70613 | -12.7896 | H | 18.3545 | -4.31205 | -13.2968 |
| H | 15.2861 | -2.19428 | -12.3776 | H | 20.8011 | -1.71851 | -10.2467 |
| H | 13.28769 | -2.19798 | -8.14955 | H | 19.1413 | -0.99099 | -5.93977 |
| H | 16.38655 | -6.03802 | 1.909852 | H | 16.9148 | -5.02771 | 2.02241 |
| H | 18.82345 | -1.85586 | 5.088637 | H | 18.7369 | 0.15022 | 3.88314 |
| H | 17.41914 | -3.79947 | 9.643057 | H | 24.0611 | -3.31675 | 6.98899 |
| H | 15.25397 | -5.88621 | 8.145892 | H | 23.6636 | -3.14243 | 3.64787 |
| H | 17.71798 | -7.1215 | 10.08577 | H | 23.1226 | -0.4303 | 5.55978 |
| H | 22.08769 | -3.71515 | 8.054852 | H | 17.9138 | -6.6831 | 6.0268 |
| H | 22.9599 | -5.71652 | 5.517865 | H | 21.019 | -6.92971 | 7.26273 |
| H | 22.22792 | -7.04181 | 8.522505 | H | 20.5318 | -6.92006 | 3.94113 |
| H | 19.61071 | -8.49094 | 3.947015 | H | 17.6632 | -2.62676 | 8.43809 |
| **1B-7** | | | | **1B-8** | | | |
| Atom | X | Y | Z | Atom | X | Y | Z |
| C | 9.026899 | -1.70639 | -4.85296 | C | 9.48036 | -2.85332 | -5.21995 |
| C | 13.1437 | -4.52162 | 1.42302 | C | 11.9007 | -4.23235 | 1.78413 |
| C | 8.979473 | 4.819535 | 9.355978 | C | 14.9525 | 1.71578 | 9.84147 |
| C | 8.144003 | 0.141321 | 8.906063 | C | 12.2686 | -2.21585 | 9.67965 |
| C | 9.742971 | 2.361823 | 8.14879 | C | 12.8657 | 0.30886 | 8.52522 |
| C | 11.68055 | 2.257795 | 6.52749 | C | 11.7347 | 1.26879 | 6.47739 |
| C | 12.66736 | 0.004795 | 5.118311 | C | 9.6554 | 0.1399 | 4.92444 |
| C | 11.36069 | 4.547512 | -5.82543 | C | 12.4407 | 5.2301 | -5.86973 |
| C | 13.53598 | 7.197947 | -2.50371 | C | 10.2318 | 4.03859 | -9.91485 |
| O | 11.44472 | 5.372656 | -3.11255 | O | 10.1897 | 3.98798 | -7.07449 |
| C | 9.846714 | 4.588385 | -1.3134 | C | 8.26659 | 2.91578 | -5.83006 |
| O | 5.84763 | -1.34749 | -0.91916 | O | 5.31745 | -1.10167 | -3.13086 |
| C | 7.698403 | 2.765893 | -1.59481 | C | 7.95379 | 2.65663 | -3.03433 |
| C | 8.197659 | -0.03066 | -0.78831 | C | 7.61926 | -0.14141 | -2.16241 |
| C | 9.129121 | -0.25599 | 1.936249 | C | 7.55296 | -0.31327 | 0.7213 |
| C | 11.97648 | 0.116829 | 2.288084 | C | 10.0751 | 0.26073 | 2.04082 |
| C | 13.45758 | -1.74873 | 0.617099 | C | 12.2047 | -1.45717 | 0.97466 |
| C | 12.60863 | -1.49862 | -2.12907 | C | 12.1995 | -1.36903 | -1.91808 |
| C | 9.980229 | -1.1954 | -2.78672 | C | 9.83306 | -1.63454 | -3.27417 |
| C | 16.29819 | -1.01766 | 0.355909 | C | 14.9368 | -0.4587 | 1.52288 |
| O | 16.88527 | -1.40885 | -2.30792 | O | 16.2668 | -0.5597 | -0.88297 |
| C | 14.69804 | -1.46846 | -3.6451 | C | 14.5837 | -0.95868 | -2.78092 |
| C | 15.08973 | -1.35686 | -6.3763 | C | 15.6787 | -0.66497 | -5.30262 |
| C | 13.43503 | -2.51344 | -8.08782 | C | 14.5809 | -1.74094 | -7.45939 |
| C | 13.89729 | -2.35534 | -10.6677 | C | 15.6578 | -1.33854 | -9.81974 |
| C | 15.99517 | -1.05447 | -11.5729 | C | 17.8419 | 0.1058 | -10.0695 |
| C | 17.66445 | 0.076275 | -9.87887 | C | 18.9653 | 1.14575 | -7.92878 |
| C | 17.23085 | -0.08576 | -7.29986 | C | 17.9016 | 0.76351 | -5.56319 |
| C | 18.15116 | -2.41237 | 1.952431 | C | 16.4429 | -1.82511 | 3.47378 |
| C | 19.85524 | -4.04234 | 1.091417 | C | 18.5981 | -3.04685 | 3.06513 |
| C | 21.68627 | -5.48231 | 2.719511 | C | 20.0821 | -4.38383 | 5.1079 |
| C | 24.40217 | -4.7679 | 1.997371 | C | 22.4557 | -2.86701 | 5.74545 |
| C | 21.24687 | -8.32717 | 2.36296 | C | 20.7779 | -7.05037 | 4.2131 |
| O | 21.20555 | -4.79478 | 5.29786 | O | 18.6804 | -4.53514 | 7.4287 |
| H | 13.81318 | -4.83887 | 3.35495 | H | 10.0723 | -4.98311 | 1.17472 |
| H | 11.16279 | -5.09919 | 1.293223 | H | 13.3787 | -5.38226 | 0.90863 |
| H | 14.2412 | -5.74616 | 0.168374 | H | 12.058 | -4.4508 | 3.83577 |
| H | 10.17721 | 6.385752 | 8.738814 | H | 15.2975 | 3.56446 | 8.98572 |
| H | 7.010003 | 5.284064 | 8.89723 | H | 14.5118 | 1.99433 | 11.8491 |
| H | 9.089151 | 4.690477 | 11.42364 | H | 16.7116 | 0.61445 | 9.76477 |
| H | 6.173061 | 0.453312 | 8.337074 | H | 10.9394 | -3.34131 | 8.57338 |
| H | 8.76492 | -1.63985 | 8.073729 | H | 14.0135 | -3.30815 | 9.93967 |
| H | 8.135226 | -0.07419 | 10.96881 | H | 11.4574 | -1.96298 | 11.5723 |
| H | 12.66081 | 4.025901 | 6.113724 | H | 12.3492 | 3.14029 | 5.85886 |
| H | 14.73318 | -0.02313 | 5.312101 | H | 9.23631 | -1.80445 | 5.49208 |
| H | 11.9756 | -1.76613 | 5.934811 | H | 7.91374 | 1.21474 | 5.30066 |
| H | 13.16684 | 3.68634 | -6.37268 | H | 12.4094 | 7.27447 | -6.22725 |
| H | 9.875348 | 3.175726 | -6.22898 | H | 12.5576 | 4.93849 | -3.82985 |
| H | 11.09795 | 6.192322 | -7.06354 | H | 14.1893 | 4.50534 | -6.71514 |
| H | 13.52105 | 7.75619 | -0.51358 | H | 8.53784 | 3.17801 | -10.7254 |
| H | 15.38517 | 6.357528 | -2.93497 | H | 10.3857 | 5.98387 | -10.6207 |
| H | 13.38723 | 8.912161 | -3.66316 | H | 11.8923 | 3.01359 | -10.6231 |
| H | 10.11148 | 5.355929 | 0.581758 | H | 6.74562 | 2.0935 | -6.9488 |
| H | 5.576635 | -1.68033 | -2.70148 | H | 5.78973 | -2.08772 | -4.60852 |
| H | 7.009797 | 2.703117 | -3.54619 | H | 6.21803 | 3.61611 | -2.41447 |
| H | 6.111434 | 3.3777 | -0.40868 | H | 9.53493 | 3.51453 | -2.02043 |
| H | 8.578315 | -2.12957 | 2.630552 | H | 6.89372 | -2.21203 | 1.23077 |
| H | 8.111848 | 1.118003 | 3.109313 | H | 6.10273 | 1.00255 | 1.4072 |
| H | 12.47582 | 2.008784 | 1.596451 | H | 10.6205 | 2.21583 | 1.58621 |
| H | 16.48213 | 1.033712 | 0.69246 | H | 14.8213 | 1.55708 | 2.03664 |
| H | 11.7964 | -3.51109 | -7.38154 | H | 12.8945 | -2.88404 | -7.26701 |
| H | 12.60534 | -3.2617 | -11.9796 | H | 14.7865 | -2.17992 | -11.477 |
| H | 16.3384 | -0.93014 | -13.5931 | H | 18.6752 | 0.40737 | -11.9213 |
| H | 19.30988 | 1.087663 | -10.5731 | H | 20.6745 | 2.26856 | -8.10415 |
| H | 18.52716 | 0.791589 | -5.97608 | H | 18.7622 | 1.59703 | -3.90092 |
| H | 18.05434 | -2.03641 | 3.968996 | H | 15.6794 | -1.71805 | 5.37523 |
| H | 19.98861 | -4.42354 | -0.92516 | H | 19.4096 | -3.11034 | 1.17491 |
| H | 24.71015 | -2.75115 | 2.326602 | H | 21.9228 | -1.01112 | 6.48229 |
| H | 25.7598 | -5.84938 | 3.131524 | H | 23.5722 | -3.86304 | 7.17413 |
| H | 24.77148 | -5.17828 | 0.003299 | H | 23.6167 | -2.59803 | 4.05642 |
| H | 22.58994 | -9.41338 | 3.509355 | H | 21.9251 | -6.97905 | 2.49246 |
| H | 21.50878 | -8.87858 | 0.385709 | H | 19.0704 | -8.14416 | 3.78692 |
| H | 19.32449 | -8.82051 | 2.94003 | H | 21.8479 | -8.03 | 5.68685 |
| H | 22.39698 | -5.69652 | 6.33875 | H | 17.2218 | -5.58432 | 7.10749 |

**For compound 2**

**
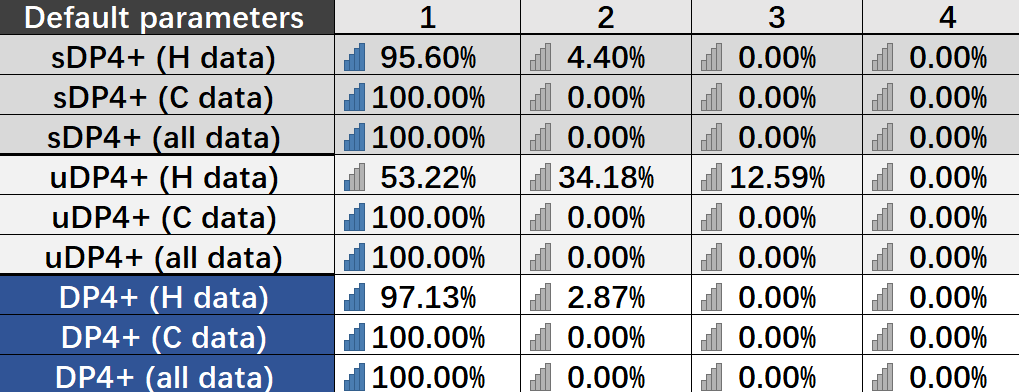
**


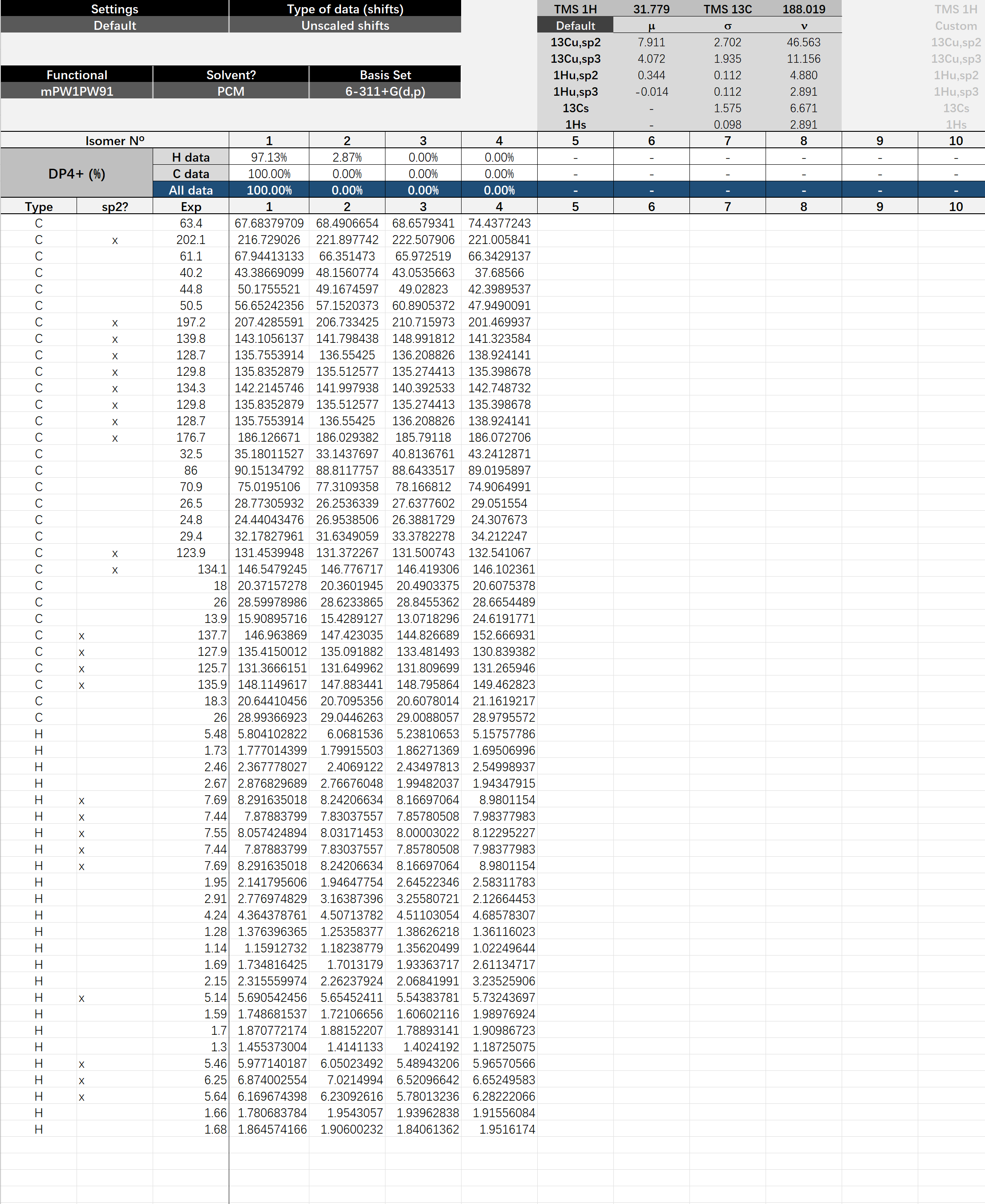


**Figure S40.** The DP4+ analysis of experimental NMR chemical shifts of **2** and calculated experimental NMR chemical shifts of Isomer 1: (1*R**,3*R**,5*S**,6*S**,16*R**)-**2** (**2A**), Isomer 2: (1*R**,3*R**,5*S**,6*S**,16*S**)-**2** (**2B**), Isomer 3: (1*R**,3*S**,5*S**,6*S**,16*R**)-**2** (**2C**), and Isomer 4: (1*R**,3*S**,5*S**,6*S**,16*S**)-**2** (**2D**).

| No. | **2***^b^* | | **Spirohypolactone B***^a^* | | **Spirohypolactone A***^a^* | | **Norhyperpalum H***^a^* | |
| --- | --- | --- | --- | --- | --- | --- | --- | --- |
|  | *δ*_H_ | *δ*_C_ | *δ*_H_ | *δ*_C_ | *δ*_H_ | *δ*_C_ | *δ*_H_ | *δ*_C_ |
| 3 |  | 61.1 |  | 61.3 |  | 60.0 |  | 61.3 |
| 5 | 2.67 m | 44.8 | 2.69 m | 39.1 | 2.25 m | 39.2 | 2.69 m | 39.1 |
| 14 |  | 176.7 |  | 176.0 |  | 176.0 |  | 176.3 |
| 15 | 2.91 dd (13.0, 7.0) | 32.5 | 2.87 dd (12.9, 6.9) | 32.4 | 3.11 dd (13.3, 10.2) | 31.5 | 2.87 dd (12.9, 6.9) | 32.4 |
|  | 1.95 dd (13.1, 9.3) |  | 1.89 dd (12.9, 9.4) |  | 1.77 dd (13.3, 6.5) |  | 1.89 dd (13.0, 9.4) |  |
| 16 | 4.24 dd (9.3, 7.0) | 86.0 | 4.26 dd (9.4, 6.9) | 85.9 | 4.40 dd (10.2, 6.4) | 84.3 | 4.25 dd (9.4, 6.9) | 85.8 |
| 17 |  | 70.9 |  | 70.8 |  | 71.7 |  | 70.8 |
| 18 | 1.28 s | 26.5 | 1.29 s | 26.5 | 1.28 s | 26.3 | 1.29 s | 26.5 |
| 19 | 1.14 s | 24.8 | 1.15 s | 24.8 | 1.16 s | 24.8 | 1.15 s | 24.8 |

*^a^* Reorded in CD_3_OD (400 MHz for ^1^H and 100 MHz for ^13^C NMR)

*^b^* Reorded in CD_3_OD (600 MHz for ^1^H and 125 MHz for ^13^C NMR)

**Figure S41.** The comparison of partial NMR data between **2**, spirohypolactones A and B, and norhyperpalum H in CD_3_OD.

**Table S5.** Gibbs free energies^a^ and equilibrium populations^b^ of low-energy conformers of **2A**.

| Conformers | ∆G(a.u.) | P(%)/100 | G(a.u.) |
| --- | --- | --- | --- |
| **2A-1** | 0.00018 | 21.79 | -1580.922566 |
| **2A-2** | 0.00268 | 1.54 | -1580.920063 |
| **2A-3** | 0.00319 | 0.9 | -1580.919562 |
| **2A-4** | 0.00268 | 1.54 | -1580.920063 |
| **2A-5** | 0.00098 | 9.39 | -1580.921772 |
| **2A-6** | 0.00201 | 3.15 | -1580.920741 |
| **2A-7** | 0.00174 | 4.18 | -1580.921008 |
| **2A-8** | 0.0053 | 0.1 | -1580.917446 |
| **2A-9** | 0.00159 | 4.88 | -1580.921155 |
| **2A-10** | 0.0036 | 0.58 | -1580.919147 |
| **2A-11** | 0.00031 | 19.05 | -1580.92244 |
| **2A-12** | 0.0025 | 1.88 | -1580.920252 |
| **2A-13** | 0.00249 | 1.88 | -1580.920254 |
| **2A-14** | 0.0 | 26.41 | -1580.922748 |
| **2A-15** | 0.00471 | 0.18 | -1580.918038 |
| **2A-16** | 0.00446 | 0.23 | -1580.918288 |
| **2A-17** | 0.00449 | 0.23 | -1580.918255 |
| **2A-18** | 0.00287 | 1.27 | -1580.91988 |
| **2A-19** | 0.00329 | 0.81 | -1580.919462 |

^a^wB97M-V/def2-TZVP, in a.u.
^b^From ∆G values at 298.15K.

**Table S6.** Cartesian coordinates for the low-energy reoptimized random research conformers of **2A** at B3LYP-D3(BJ)/6-31G* level of theory in methanol.

| **2A-1** | | | | **2A-2** | | | |
| --- | --- | --- | --- | --- | --- | --- | --- |
| Atom | X | Y | Z | Atom | X | Y | Z |
| C | 0.531423 | 1.730233 | 1.684954 | C | 0.577582 | 1.721225 | 1.699081 |
| C | -2.241697 | 1.161346 | 2.138569 | C | -2.212866 | 1.241847 | 2.151903 |
| C | -3.118517 | -1.429754 | 1.14905 | C | -3.170596 | -1.325981 | 1.173866 |
| C | -1.284838 | -3.50561 | 2.089946 | C | -1.393785 | -3.45718 | 2.101512 |
| C | 1.539755 | -2.873056 | 1.912586 | C | 1.447251 | -2.908721 | 1.916076 |
| C | 2.201051 | -0.260571 | 3.125121 | C | 2.187364 | -0.320443 | 3.134387 |
| C | -3.183809 | -1.24031 | -1.746201 | C | -3.240459 | -1.127858 | -1.723105 |
| O | -5.578609 | -0.930964 | -2.547366 | O | -5.627395 | -0.780597 | -2.521242 |
| C | -7.356048 | -0.820135 | -0.419497 | C | -7.396324 | -0.672102 | -0.406624 |
| C | -5.888377 | -1.953399 | 1.794998 | C | -5.950927 | -1.772748 | 1.843517 |
| C | 1.090289 | 4.508524 | 2.234985 | C | 1.224896 | 4.480081 | 2.249216 |
| C | 0.115356 | 6.42231 | 0.40127 | C | 0.348101 | 6.421133 | 0.394777 |
| O | 2.278108 | 5.170829 | 4.104495 | O | 2.406133 | 5.106539 | 4.135371 |
| C | 4.964309 | 0.369506 | 2.658935 | C | 4.968079 | 0.226514 | 2.666997 |
| C | 1.659357 | -0.237185 | 5.982232 | C | 1.648973 | -0.28814 | 5.991953 |
| C | 6.066849 | 0.427522 | 0.370914 | C | 6.065754 | 0.273751 | 0.376367 |
| C | 8.725553 | 0.970548 | -0.034408 | C | 8.739673 | 0.733777 | -0.031398 |
| C | 9.984051 | 0.904865 | -2.253549 | C | 9.988539 | 0.654212 | -2.255513 |
| C | 12.765017 | 1.498343 | -2.362711 | C | 12.787433 | 1.15608 | -2.36671 |
| C | 8.809299 | 0.241414 | -4.75919 | C | 8.784585 | 0.06224 | -4.765213 |
| C | 3.124932 | -5.073584 | 3.000946 | C | 2.969911 | -5.158246 | 2.993402 |
| C | 2.922849 | -7.440961 | 1.424651 | C | 2.694149 | -7.513483 | 1.410012 |
| C | 4.266858 | -7.972205 | -0.659333 | C | 4.01128 | -8.072453 | -0.683854 |
| C | 3.821939 | -10.374024 | -2.127491 | C | 3.49313 | -10.455146 | -2.159112 |
| C | 6.284804 | -6.269314 | -1.732499 | C | 6.068391 | -6.420021 | -1.761623 |
| C | -9.758591 | -2.246127 | -1.216439 | C | -9.846359 | -2.023861 | -1.18881 |
| C | -10.966784 | -0.983676 | -3.527614 | C | -9.391319 | -4.757293 | -2.024945 |
| O | -9.122171 | -4.816279 | -1.785481 | O | -11.311356 | -1.951789 | 1.094082 |
| C | -11.606602 | -2.338447 | 0.998915 | C | -11.172203 | -0.522681 | -3.280495 |
| O | -3.659254 | 2.589951 | 3.253377 | O | -3.58824 | 2.725905 | 3.246814 |
| O | -1.416902 | -1.292542 | -3.203142 | O | -1.473737 | -1.19878 | -3.181038 |
| C | -1.005674 | 5.776462 | -1.915717 | C | 0.673542 | 8.976396 | 1.034978 |
| C | -1.889482 | 7.65773 | -3.534147 | C | -0.11605 | 10.876933 | -0.598605 |
| C | -1.667502 | 10.198383 | -2.859358 | C | -1.240524 | 10.248703 | -2.906291 |
| C | -0.549179 | 10.859906 | -0.558036 | C | -1.565956 | 7.715521 | -3.566803 |
| C | 0.337412 | 8.98496 | 1.055378 | C | -0.778518 | 5.80858 | -1.928594 |
| H | 0.891227 | 1.386736 | -0.321384 | H | 0.92172 | 1.366174 | -0.307729 |
| H | -1.663906 | -5.236858 | 1.023226 | H | -1.827962 | -5.174356 | 1.032665 |
| H | -1.790404 | -3.908509 | 4.057932 | H | -1.903147 | -3.849839 | 4.07065 |
| H | 2.013611 | -2.732569 | -0.094976 | H | 1.919778 | -2.776068 | -0.092377 |
| H | -7.778341 | 1.179284 | -0.112121 | H | -7.809575 | 1.328236 | -0.089002 |
| H | -6.433575 | -1.114177 | 3.595448 | H | -6.481224 | -0.844366 | 3.602765 |
| H | -6.208937 | -3.993764 | 1.853734 | H | -6.299298 | -3.798162 | 2.034048 |
| H | 6.100435 | 0.788089 | 4.322473 | H | 6.121964 | 0.588839 | 4.331664 |
| H | -0.318124 | -0.668574 | 6.407183 | H | -0.341135 | -0.657368 | 6.416684 |
| H | 2.831439 | -1.642427 | 6.944604 | H | 2.775843 | -1.733143 | 6.94957 |
| H | 2.088656 | 1.61966 | 6.765592 | H | 2.137397 | 1.551851 | 6.780194 |
| H | 4.925409 | 0.022662 | -1.290936 | H | 4.907022 | -0.075958 | -1.286032 |
| H | 9.795002 | 1.451444 | 1.660315 | H | 9.829588 | 1.157803 | 1.665467 |
| H | 13.131558 | 3.100479 | -3.632405 | H | 13.802112 | -0.47523 | -3.155752 |
| H | 13.531726 | 1.959775 | -0.498655 | H | 13.203543 | 2.760899 | -3.617595 |
| H | 13.836091 | -0.107871 | -3.128315 | H | 13.574589 | 1.568314 | -0.499642 |
| H | 9.751444 | -1.417884 | -5.577117 | H | 9.667353 | -1.617706 | -5.606885 |
| H | 6.791708 | -0.172088 | -4.643751 | H | 6.75442 | -0.283716 | -4.648174 |
| H | 9.071116 | 1.790349 | -6.115949 | H | 9.09487 | 1.61825 | -6.103521 |
| H | 2.502324 | -5.483706 | 4.933918 | H | 2.340997 | -5.556097 | 4.926868 |
| H | 5.092071 | -4.448565 | 3.131665 | H | 4.955032 | -4.591732 | 3.120461 |
| H | 1.494155 | -8.803297 | 2.011901 | H | 1.232374 | -8.838788 | 2.000562 |
| H | 3.281183 | -9.9549 | -4.088228 | H | 5.1921 | -11.6433 | -2.277167 |
| H | 5.553021 | -11.516341 | -2.230565 | H | 1.976769 | -11.575277 | -1.308159 |
| H | 2.331308 | -11.531217 | -1.280698 | H | 2.952393 | -10.013237 | -4.114843 |
| H | 5.733922 | -5.58707 | -3.615097 | H | 5.524588 | -5.713364 | -3.637281 |
| H | 6.66976 | -4.618442 | -0.557957 | H | 6.505702 | -4.786247 | -0.581459 |
| H | 8.061318 | -7.312233 | -1.987317 | H | 7.813972 | -7.509957 | -2.032829 |
| H | -9.646311 | -0.943036 | -5.119527 | H | -11.196837 | -5.632367 | -2.535298 |
| H | -12.649978 | -2.042458 | -4.093328 | H | -8.154845 | -4.833035 | -3.679361 |
| H | -11.524196 | 0.964344 | -3.104688 | H | -8.546016 | -5.882859 | -0.512341 |
| H | -7.948526 | -4.769463 | -3.198348 | H | -12.934537 | -2.722604 | 0.721951 |
| H | -13.332891 | -3.316173 | 0.41735 | H | -10.00068 | -0.427517 | -4.982 |
| H | -10.792884 | -3.357159 | 2.602703 | H | -12.96519 | -1.428395 | -3.784957 |
| H | -12.110697 | -0.428943 | 1.612834 | H | -11.569502 | 1.40373 | -2.638986 |
| H | -1.17873 | 3.821431 | -2.501769 | H | 1.546775 | 9.427549 | 2.833168 |
| H | -2.74715 | 7.136698 | -5.323496 | H | 0.139229 | 12.84589 | -0.080225 |
| H | -2.360506 | 11.659858 | -4.122543 | H | -1.858009 | 11.730163 | -4.185297 |
| H | -0.374217 | 12.834708 | -0.028731 | H | -2.429625 | 7.219975 | -5.360526 |
| H | 1.207535 | 9.46204 | 2.848439 | H | -1.032263 | 3.858974 | -2.503284 |
| **2A-3** | | | | **2A-4** | | | |
| Atom | X | Y | Z | Atom | X | Y | Z |
| C | 0.020951 | 0.69232 | 1.792738 | C | 0.577068 | 1.721554 | 1.698852 |
| C | -2.792462 | 0.76635 | 2.296407 | C | -2.213242 | 1.241533 | 2.151731 |
| C | -4.307 | -1.117994 | 0.671085 | C | -3.170399 | -1.326586 | 1.17376 |
| C | -3.077222 | -3.765385 | 0.812344 | C | -1.393119 | -3.457316 | 2.101428 |
| C | -0.176563 | -3.886055 | 0.634245 | C | 1.447808 | -2.908219 | 1.916029 |
| C | 1.134922 | -1.986098 | 2.483986 | C | 2.187335 | -0.319751 | 3.134226 |
| C | -4.330939 | -0.056936 | -2.031272 | C | -3.240429 | -1.128408 | -1.723177 |
| O | -6.571058 | 1.045701 | -2.519028 | O | -5.627449 | -0.781377 | -2.521199 |
| C | -8.258942 | 0.937412 | -0.320263 | C | -7.396347 | -0.6735 | -0.406512 |
| C | -7.117716 | -1.119625 | 1.356536 | C | -5.950613 | -1.773914 | 1.843513 |
| C | 1.419224 | 2.8916 | 3.028666 | C | 1.2238 | 4.48055 | 2.248942 |
| C | 2.451527 | 4.915268 | 1.352657 | C | 0.346527 | 6.421425 | 0.394546 |
| O | 1.723693 | 2.992999 | 5.319235 | O | 2.404988 | 5.107257 | 4.135044 |
| C | 3.971229 | -1.910067 | 2.04965 | C | 4.967897 | 0.22798 | 2.66681 |
| C | 0.620091 | -2.666793 | 5.262798 | C | 1.648966 | -0.287431 | 5.991802 |
| C | 5.171151 | -0.629351 | 0.213269 | C | 6.065713 | 0.274632 | 0.376235 |
| C | 7.897572 | -0.615232 | -0.090406 | C | 8.7395 | 0.735427 | -0.03149 |
| C | 9.243399 | 0.76245 | -1.765097 | C | 9.98868 | 0.655242 | -2.255405 |
| C | 12.081558 | 0.574367 | -1.850246 | C | 12.787514 | 1.157499 | -2.366385 |
| C | 8.110234 | 2.58901 | -3.629427 | C | 8.785246 | 0.062032 | -4.76505 |
| C | 0.593794 | -6.691634 | 0.969137 | C | 2.970939 | -5.157357 | 2.993502 |
| C | 3.379106 | -7.289881 | 0.834403 | C | 2.695957 | -7.512676 | 1.410116 |
| C | 4.837121 | -7.206656 | -1.234356 | C | 4.013589 | -8.071415 | -0.683491 |
| C | 7.633658 | -7.718242 | -1.094261 | C | 3.496239 | -10.454262 | -2.15878 |
| C | 3.878557 | -6.42395 | -3.795936 | C | 6.070484 | -6.418527 | -1.760964 |
| C | -10.943663 | 0.427025 | -1.307265 | C | -9.846038 | -2.025902 | -1.188693 |
| C | -11.802647 | 2.565407 | -3.063673 | C | -9.390255 | -4.759173 | -2.024959 |
| O | -10.973235 | -1.934908 | -2.632292 | O | -11.310988 | -1.954315 | 1.094235 |
| C | -12.753021 | 0.12876 | 0.922043 | C | -11.172344 | -0.525 | -3.280287 |
| O | -3.810287 | 2.165643 | 3.808667 | O | -3.588957 | 2.725257 | 3.246646 |
| O | -2.64135 | -0.103742 | -3.577219 | O | -1.473769 | -1.199012 | -3.181201 |
| C | 1.53959 | 5.367119 | -1.097886 | C | 0.671512 | 8.976745 | 1.034742 |
| C | 2.594745 | 7.277898 | -2.57345 | C | -0.11858 | 10.877133 | -0.598771 |
| C | 4.591817 | 8.725635 | -1.63662 | C | -1.243115 | 10.24869 | -2.906367 |
| C | 5.512668 | 8.28468 | 0.800656 | C | -1.568085 | 7.715451 | -3.566879 |
| C | 4.43326 | 6.407816 | 2.291404 | C | -0.78013 | 5.808659 | -1.928747 |
| H | 0.215837 | 0.821882 | -0.25585 | H | 0.921355 | 1.366514 | -0.307937 |
| H | -3.89543 | -4.937255 | -0.685322 | H | -1.826845 | -5.174616 | 1.032597 |
| H | -3.689314 | -4.609068 | 2.602514 | H | -1.902414 | -3.850111 | 4.070557 |
| H | 0.376526 | -3.300887 | -1.269926 | H | 1.920296 | -2.775598 | -0.092438 |
| H | -8.167774 | 2.794327 | 0.581216 | H | -7.810153 | 1.326699 | -0.08877 |
| H | -7.424118 | -0.743029 | 3.35854 | H | -6.480995 | -0.845709 | 3.602833 |
| H | -7.948957 | -2.944437 | 0.857669 | H | -6.298565 | -3.799406 | 2.033999 |
| H | 5.100758 | -2.996129 | 3.380616 | H | 6.121477 | 0.591622 | 4.331396 |
| H | -1.403681 | -2.763307 | 5.679328 | H | 2.776278 | -1.73204 | 6.949494 |
| H | 1.448643 | -4.508626 | 5.708171 | H | 2.136882 | 1.552745 | 6.779918 |
| H | 1.451007 | -1.252291 | 6.510169 | H | -0.341026 | -0.657215 | 6.41659 |
| H | 4.079717 | 0.465746 | -1.13434 | H | 4.907231 | -0.076286 | -1.286078 |
| H | 8.936054 | -1.83928 | 1.20139 | H | 9.829099 | 1.160498 | 1.665317 |
| H | 12.8112 | -0.756127 | -0.44528 | H | 13.57423 | 1.571098 | -0.499436 |
| H | 12.727861 | -0.041491 | -3.725652 | H | 13.802569 | -0.474253 | -3.154053 |
| H | 12.959336 | 2.426098 | -1.511658 | H | 13.2037 | 2.761474 | -3.618313 |
| H | 6.050108 | 2.653421 | -3.580543 | H | 9.095755 | 1.61746 | -6.103987 |
| H | 8.795944 | 4.512687 | -3.256743 | H | 9.668264 | -1.618252 | -5.605797 |
| H | 8.699948 | 2.116907 | -5.562785 | H | 6.755064 | -0.283882 | -4.648275 |
| H | -0.425914 | -7.752282 | -0.495605 | H | 2.341939 | -5.555349 | 4.926909 |
| H | -0.155706 | -7.376744 | 2.774243 | H | 4.955897 | -4.590321 | 3.12073 |
| H | 4.278816 | -7.790477 | 2.617428 | H | 1.234365 | -8.838283 | 2.000443 |
| H | 8.22717 | -8.237326 | 0.81818 | H | 1.97976 | -11.574554 | -1.308248 |
| H | 8.190632 | -9.242511 | -2.389385 | H | 2.956065 | -10.012542 | -4.114707 |
| H | 8.703381 | -6.032149 | -1.663849 | H | 5.195413 | -11.642183 | -2.276222 |
| H | 1.820466 | -6.52245 | -3.949768 | H | 5.526326 | -5.711096 | -3.636228 |
| H | 4.445694 | -4.463367 | -4.190379 | H | 6.507977 | -4.785219 | -0.580214 |
| H | 4.697748 | -7.600333 | -5.293809 | H | 7.816032 | -7.508313 | -2.032943 |
| H | -11.853014 | 4.375727 | -2.061498 | H | -8.153768 | -4.834483 | -3.67938 |
| H | -10.518885 | 2.758082 | -4.674349 | H | -8.544635 | -5.884566 | -0.512403 |
| H | -13.699076 | 2.154382 | -3.77699 | H | -11.195529 | -5.634725 | -2.535353 |
| H | -9.820752 | -1.759647 | -4.052455 | H | -12.933837 | -2.72589 | 0.72224 |
| H | -12.215849 | -1.478645 | 2.105115 | H | -11.570158 | 1.401274 | -2.638687 |
| H | -12.766546 | 1.836122 | 2.089228 | H | -10.000886 | -0.429438 | -4.981817 |
| H | -14.668882 | -0.19673 | 0.216417 | H | -12.965095 | -1.431177 | -3.784749 |
| H | -0.004726 | 4.261053 | -1.868661 | H | 1.544789 | 9.428042 | 2.832875 |
| H | 1.864644 | 7.626203 | -4.458745 | H | 0.136363 | 12.846139 | -0.080415 |
| H | 5.429971 | 10.192654 | -2.801385 | H | -1.861002 | 11.730038 | -4.185309 |
| H | 7.068978 | 9.405055 | 1.530795 | H | -2.431798 | 7.219748 | -5.360537 |
| H | 5.120077 | 6.043424 | 4.187858 | H | -1.033469 | 3.859004 | -2.503452 |
| **2A-5** | | | | **2A-6** | | | |
| Atom | X | Y | Z | Atom | X | Y | Z |
| C | 1.276319 | -0.681052 | -1.639708 | C | 0.005055 | 0.58597 | -1.486808 |
| C | -1.286654 | -1.814814 | -2.237659 | C | 2.786156 | 0.40449 | -2.135048 |
| C | -3.4881 | -0.593278 | -0.76714 | C | 4.308627 | -1.250245 | -0.281478 |
| C | -3.331787 | 2.321188 | -0.921778 | C | 2.945108 | -3.796653 | 0.170117 |
| C | -0.691555 | 3.473867 | -0.588839 | C | 0.061637 | -3.692464 | 0.52168 |
| C | 1.304956 | 2.205994 | -2.363008 | C | -1.254258 | -2.114324 | -1.611003 |
| C | -3.277087 | -1.562414 | 1.962556 | C | 4.577501 | 0.296056 | 2.162378 |
| O | -4.981122 | -3.406743 | 2.372109 | O | 6.902826 | 1.31785 | 2.301014 |
| C | -6.460762 | -3.952969 | 0.090696 | C | 8.42833 | 0.687883 | 0.07194 |
| C | -6.052443 | -1.644954 | -1.597769 | C | 7.059822 | -1.558928 | -1.122565 |
| C | 3.437051 | -2.210859 | -2.783945 | C | -1.361759 | 2.583481 | -3.055258 |
| C | 5.062064 | -3.727511 | -1.043259 | C | -2.243691 | 4.927686 | -1.752439 |
| O | 3.862253 | -2.179007 | -5.057189 | O | -1.763445 | 2.273639 | -5.312115 |
| C | 3.910068 | 3.294135 | -1.857223 | C | -4.065498 | -1.839571 | -1.112716 |
| C | 0.658781 | 2.597251 | -5.173651 | C | -0.889524 | -3.323762 | -4.231286 |
| C | 5.515183 | 2.554787 | -0.031258 | C | -5.158722 | -0.184904 | 0.474892 |
| C | 8.019554 | 3.61607 | 0.331825 | C | -7.872986 | -0.00535 | 0.831493 |
| C | 9.810776 | 2.767978 | 1.939651 | C | -9.12424 | 1.745674 | 2.202623 |
| C | 12.349557 | 4.045153 | 2.085502 | C | -11.963595 | 1.688397 | 2.384914 |
| C | 9.506881 | 0.52157 | 3.659725 | C | -7.88015 | 3.883707 | 3.608791 |
| C | -0.830888 | 6.374902 | -0.899084 | C | -0.921774 | -6.429999 | 0.794031 |
| C | -2.30324 | 7.598154 | 1.202368 | C | -3.609277 | -6.623955 | 1.686161 |
| C | -4.628561 | 8.604465 | 1.106282 | C | -5.551093 | -7.833378 | 0.597197 |
| C | -5.836818 | 9.716726 | 3.437389 | C | -8.148569 | -7.752516 | 1.768621 |
| C | -6.249549 | 8.722568 | -1.23408 | C | -5.410286 | -9.258776 | -1.864213 |
| C | -9.199905 | -4.44382 | 0.927003 | C | 11.139747 | 0.181548 | 0.986138 |
| C | -9.324119 | -6.729203 | 2.703741 | C | 12.237182 | 2.540695 | 2.260982 |
| O | -10.15892 | -2.239736 | 2.175319 | O | 11.122459 | -1.893935 | 2.72631 |
| C | -10.86386 | -4.848087 | -1.396818 | C | 12.773776 | -0.644697 | -1.245381 |
| O | -1.623054 | -3.547509 | -3.70919 | O | 3.778865 | 1.461019 | -3.917905 |
| O | -1.810836 | -0.886334 | 3.586997 | O | 2.997125 | 0.653817 | 3.781142 |
| C | 4.298953 | -4.445318 | 1.39506 | C | -4.192989 | 6.315314 | -2.89792 |
| C | 5.913777 | -5.854366 | 2.928129 | C | -5.134749 | 8.488115 | -1.757474 |
| C | 8.312964 | -6.527184 | 2.060958 | C | -4.106759 | 9.329512 | 0.526763 |
| C | 9.088487 | -5.815719 | -0.362939 | C | -2.142437 | 7.983753 | 1.663312 |
| C | 7.465187 | -4.444071 | -1.909468 | C | -1.225026 | 5.781562 | 0.544305 |
| H | 1.424969 | -0.713304 | 0.417374 | H | -0.067287 | 1.108606 | 0.506787 |
| H | -4.606716 | 3.12319 | 0.494167 | H | 3.796951 | -4.696315 | 1.828045 |
| H | -4.097833 | 2.868512 | -2.766582 | H | 3.398213 | -5.013976 | -1.443256 |
| H | -0.063969 | 3.091639 | 1.3477 | H | -0.332843 | -2.706507 | 2.299991 |
| H | -5.646791 | -5.660153 | -0.740903 | H | 8.384819 | 2.348508 | -1.156701 |
| H | -6.075984 | -2.137749 | -3.597844 | H | 7.242848 | -1.571755 | -3.174377 |
| H | -7.524357 | -0.247658 | -1.210294 | H | 7.824443 | -3.314408 | -0.346438 |
| H | 4.498032 | 4.806182 | -3.12576 | H | -5.276074 | -3.113297 | -2.178229 |
| H | 0.667117 | 4.612617 | -5.635929 | H | 1.105975 | -3.608784 | -4.694193 |
| H | 2.04694 | 1.643117 | -6.36065 | H | -1.828752 | -5.163306 | -4.306816 |
| H | -1.204965 | 1.843553 | -5.658864 | H | -1.703669 | -2.1136 | -5.687322 |
| H | 4.95956 | 1.043095 | 1.239082 | H | -3.986795 | 1.104849 | 1.557641 |
| H | 8.482805 | 5.230819 | -0.861647 | H | -8.9877 | -1.435103 | -0.148965 |
| H | 12.72213 | 4.734505 | 4.008763 | H | -12.788691 | 3.455755 | 1.671038 |
| H | 13.875838 | 2.709034 | 1.640367 | H | -12.776367 | 0.115279 | 1.316761 |
| H | 12.488081 | 5.646065 | 0.784223 | H | -12.57664 | 1.517529 | 4.362026 |
| H | 10.895543 | -0.942939 | 3.175556 | H | -8.413792 | 3.84553 | 5.614427 |
| H | 9.880365 | 1.059331 | 5.629184 | H | -5.822112 | 3.870951 | 3.486996 |
| H | 7.641028 | -0.350334 | 3.571851 | H | -8.529963 | 5.707685 | 2.860807 |
| H | -1.632238 | 6.840673 | -2.747325 | H | 0.304168 | -7.356812 | 2.194813 |
| H | 1.103222 | 7.121671 | -0.881843 | H | -0.625853 | -7.456017 | -0.975705 |
| H | -1.345428 | 7.592056 | 3.028893 | H | -3.987868 | -5.620868 | 3.446161 |
| H | -4.601901 | 9.571716 | 5.090038 | H | -9.50828 | -6.86158 | 0.474667 |
| H | -6.305154 | 11.719225 | 3.150168 | H | -8.867126 | -9.663031 | 2.149868 |
| H | -7.624213 | 8.755494 | 3.876622 | H | -8.160699 | -6.686741 | 3.540884 |
| H | -5.317824 | 7.967783 | -2.91327 | H | -3.504267 | -9.352553 | -2.650237 |
| H | -8.004884 | 7.65149 | -0.948299 | H | -6.096171 | -11.203222 | -1.626737 |
| H | -6.814783 | 10.678944 | -1.635101 | H | -6.642436 | -8.372908 | -3.282806 |
| H | -8.65967 | -8.444888 | 1.75541 | H | 12.324512 | 4.129339 | 0.93707 |
| H | -8.149954 | -6.425481 | 4.379595 | H | 11.077291 | 3.113054 | 3.875341 |
| H | -11.277538 | -7.02723 | 3.311707 | H | 14.150369 | 2.137316 | 2.932711 |
| H | -9.105693 | -1.970321 | 3.656652 | H | 10.092836 | -1.376083 | 4.157248 |
| H | -10.879853 | -3.168075 | -2.600756 | H | 12.804998 | 0.813695 | -2.711455 |
| H | -10.190798 | -6.455898 | -2.510078 | H | 14.711662 | -0.965151 | -0.600215 |
| H | -12.804335 | -5.234538 | -0.797079 | H | 12.067107 | -2.401594 | -2.073574 |
| H | 2.446201 | -3.941678 | 2.11353 | H | -4.963643 | 5.637782 | -4.672089 |
| H | 5.300234 | -6.416905 | 4.803175 | H | -6.66674 | 9.528069 | -2.641782 |
| H | 9.576639 | -7.600981 | 3.269793 | H | -4.836828 | 11.027788 | 1.417964 |
| H | 10.955704 | -6.332372 | -1.038979 | H | -1.329793 | 8.640786 | 3.428742 |
| H | 8.032525 | -3.880696 | -3.796617 | H | 0.298208 | 4.766046 | 1.466877 |
| **2A-7** | | | | **2A-8** | | | |
| Atom | X | Y | Z | Atom | X | Y | Z |
| C | -0.038573 | 1.123996 | 0.496435 | C | 1.657766 | -0.886941 | -1.721497 |
| C | -2.576821 | 0.460955 | 1.652084 | C | -0.634307 | -2.478638 | -2.379273 |
| C | -3.845219 | -1.894823 | 0.517598 | C | -3.086172 | -1.673411 | -1.019022 |
| C | -1.925342 | -4.093334 | 0.349034 | C | -3.432461 | 1.231945 | -1.293676 |
| C | 0.741245 | -3.407301 | -0.574796 | C | -1.074045 | 2.836296 | -0.804557 |
| C | 1.887285 | -1.119136 | 0.920707 | C | 1.210158 | 1.958746 | -2.461145 |
| C | -4.84045 | -1.105675 | -2.094561 | C | -2.864628 | -2.357146 | 1.796457 |
| O | -7.348521 | -0.697381 | -1.988852 | O | -4.840739 | -3.764265 | 2.540085 |
| C | -8.337079 | -1.078181 | 0.575244 | C | -6.576714 | -4.331723 | 0.48977 |
| C | -6.277751 | -2.595108 | 1.918488 | C | -5.407725 | -3.169681 | -1.907913 |
| C | 0.784818 | 3.73291 | 1.423395 | C | 4.089107 | -2.020937 | -2.7829 |
| C | -0.224095 | 5.991309 | 0.057302 | C | 5.864718 | -3.271003 | -0.97806 |
| O | 2.178852 | 3.99799 | 3.247954 | O | 4.604879 | -1.887466 | -5.033452 |
| C | 4.330944 | -0.324995 | -0.31493 | C | 3.543231 | 3.496785 | -1.817 |
| C | 2.194127 | -1.714309 | 3.744987 | C | 0.658537 | 2.251195 | -5.304316 |
| C | 6.536241 | 0.181353 | 0.830784 | C | 5.191372 | 3.015342 | 0.056102 |
| C | 8.803697 | 0.867828 | -0.556825 | C | 7.433969 | 4.516697 | 0.553454 |
| C | 11.142228 | 1.253019 | 0.388618 | C | 9.289888 | 3.967454 | 2.216809 |
| C | 13.31443 | 1.906063 | -1.330375 | C | 11.533155 | 5.694106 | 2.507944 |
| C | 11.819615 | 1.054329 | 3.145158 | C | 9.350108 | 1.648202 | 3.865047 |
| C | 2.392919 | -5.82488 | -0.541038 | C | -1.737794 | 5.666714 | -1.108888 |
| C | 4.652976 | -5.718073 | -2.258803 | C | -3.530488 | 6.536113 | 0.920922 |
| C | 7.106183 | -5.87938 | -1.65088 | C | -6.02426 | 6.955487 | 0.730689 |
| C | 9.123944 | -5.659616 | -3.652071 | C | -7.557402 | 7.718945 | 3.010069 |
| C | 8.106245 | -6.144274 | 0.998306 | C | -7.533531 | 6.691447 | -1.67162 |
| C | -10.918945 | -2.38778 | 0.326953 | C | -9.238816 | -3.402357 | 1.222874 |
| C | -12.760058 | -0.725071 | -1.169871 | C | -9.379707 | -0.535359 | 1.59429 |
| O | -10.593861 | -4.771232 | -0.917498 | O | -10.726178 | -4.151902 | -0.918819 |
| C | -11.958928 | -2.980004 | 2.953625 | C | -10.139259 | -4.786674 | 3.601489 |
| O | -3.544316 | 1.667033 | 3.356224 | O | -0.572494 | -4.25071 | -3.840239 |
| O | -3.643606 | -0.812119 | -4.025086 | O | -1.211371 | -1.754904 | 3.26751 |
| C | -1.92619 | 5.832952 | -1.973004 | C | 5.112478 | -4.15287 | 1.40919 |
| C | -2.821263 | 8.024337 | -3.130641 | C | 6.866524 | -5.29345 | 3.011547 |
| C | -2.02171 | 10.391974 | -2.286933 | C | 9.386258 | -5.530588 | 2.265767 |
| C | -0.323659 | 10.568996 | -0.269195 | C | 10.149625 | -4.653502 | -0.107223 |
| C | 0.560566 | 8.385928 | 0.894237 | C | 8.39497 | -3.55109 | -1.724281 |
| H | -0.316501 | 1.200214 | -1.549851 | H | 1.73763 | -0.894269 | 0.338637 |
| H | -2.720512 | -5.542862 | -0.895734 | H | -4.932656 | 1.84668 | -0.018153 |
| H | -1.814581 | -4.947118 | 2.232827 | H | -4.12665 | 1.57483 | -3.213339 |
| H | 0.574112 | -2.800646 | -2.549577 | H | -0.496688 | 2.55637 | 1.165566 |
| H | -8.562638 | 0.802075 | 1.401591 | H | -6.64927 | -6.390918 | 0.362398 |
| H | -6.170717 | -2.14521 | 3.925802 | H | -4.815381 | -4.653849 | -3.209777 |
| H | -6.651556 | -4.61295 | 1.678139 | H | -6.763061 | -1.970327 | -2.893137 |
| H | 4.227582 | -0.14676 | -2.366209 | H | 3.879948 | 5.133271 | -3.021489 |
| H | 3.585077 | -3.213784 | 4.030988 | H | -0.982777 | 1.143199 | -5.900067 |
| H | 2.81507 | -0.037358 | 4.764328 | H | 0.287378 | 4.233648 | -5.761354 |
| H | 0.41142 | -2.334226 | 4.589914 | H | 2.274824 | 1.611923 | -6.41056 |
| H | 6.666253 | 0.020027 | 2.871108 | H | 4.88658 | 1.386734 | 1.264714 |
| H | 8.560369 | 1.046532 | -2.595682 | H | 7.628788 | 6.228513 | -0.577171 |
| H | 12.725499 | 2.021504 | -3.30939 | H | 13.300881 | 4.686334 | 2.092567 |
| H | 14.167173 | 3.723575 | -0.797763 | H | 11.413893 | 7.337463 | 1.258688 |
| H | 14.826298 | 0.48901 | -1.1849 | H | 11.696163 | 6.372899 | 4.463869 |
| H | 12.649101 | 2.833533 | 3.820422 | H | 7.695146 | 0.435911 | 3.662087 |
| H | 10.211594 | 0.601326 | 4.355753 | H | 11.017587 | 0.496168 | 3.418048 |
| H | 13.265137 | -0.408857 | 3.430085 | H | 9.522689 | 2.18228 | 5.863267 |
| H | 1.176288 | -7.386565 | -1.174401 | H | -2.514252 | 6.004187 | -2.995477 |
| H | 2.926407 | -6.282357 | 1.401515 | H | 0.014345 | 6.765601 | -0.977109 |
| H | 4.18395 | -5.458608 | -4.251047 | H | -2.670722 | 6.751761 | 2.782788 |
| H | 10.332461 | -4.013725 | -3.277401 | H | -6.386823 | 7.883784 | 4.706981 |
| H | 10.368211 | -7.322124 | -3.657074 | H | -8.509767 | 9.537077 | 2.696681 |
| H | 8.31523 | -5.449124 | -5.544383 | H | -9.0547 | 6.330418 | 3.388581 |
| H | 9.142404 | -4.425499 | 1.52743 | H | -6.380753 | 6.208026 | -3.313117 |
| H | 6.628197 | -6.447177 | 2.405909 | H | -8.979839 | 5.217254 | -1.456282 |
| H | 9.449007 | -7.72116 | 1.126453 | H | -8.550077 | 8.450969 | -2.093195 |
| H | -13.078439 | 1.081383 | -0.211001 | H | -8.09763 | 0.097374 | 3.088977 |
| H | -12.023246 | -0.327221 | -3.061453 | H | -8.926904 | 0.469183 | -0.152346 |
| H | -14.573836 | -1.698956 | -1.359465 | H | -11.300838 | 0.00072 | 2.149671 |
| H | -9.935974 | -4.407909 | -2.59411 | H | -12.457579 | -3.645403 | -0.583782 |
| H | -13.812581 | -3.877924 | 2.774989 | H | -12.083298 | -4.233555 | 4.052811 |
| H | -10.708538 | -4.271219 | 3.97417 | H | -10.095533 | -6.833463 | 3.302722 |
| H | -12.174336 | -1.248894 | 4.064408 | H | -8.946595 | -4.327295 | 5.226389 |
| H | -2.574155 | 4.022749 | -2.678887 | H | 3.16586 | -3.983274 | 2.0295 |
| H | -4.140352 | 7.878572 | -4.695181 | H | 6.26555 | -5.987425 | 4.846344 |
| H | -2.717394 | 12.094748 | -3.197184 | H | 10.752993 | -6.394416 | 3.52964 |
| H | 0.302131 | 12.408316 | 0.391221 | H | 12.10952 | -4.831368 | -0.688484 |
| H | 1.86971 | 8.486267 | 2.467534 | H | 8.951946 | -2.863682 | -3.573094 |
| **2A-9** | | | | **2A-10** | | | |
| Atom | X | Y | Z | Atom | X | Y | Z |
| C | 0.552745 | 1.728107 | 1.688313 | C | 1.298563 | -0.683648 | -1.648979 |
| C | -2.22793 | 1.199445 | 2.141324 | C | -1.254869 | -1.834134 | -2.256977 |
| C | -3.139599 | -1.385853 | 1.164071 | C | -3.472052 | -0.620885 | -0.802053 |
| C | -1.33176 | -3.483175 | 2.10914 | C | -3.32681 | 2.294617 | -0.950079 |
| C | 1.500061 | -2.887229 | 1.926281 | C | -0.694607 | 3.459045 | -0.600028 |
| C | 2.194923 | -0.281516 | 3.13412 | C | 1.316213 | 2.204122 | -2.36722 |
| C | -3.202351 | -1.206431 | -1.734591 | C | -3.263001 | -1.594712 | 1.928341 |
| O | -5.592731 | -0.895708 | -2.538324 | O | -4.974647 | -3.426083 | 2.342758 |
| C | -7.374526 | -0.784367 | -0.417934 | C | -6.481029 | -3.938586 | 0.090391 |
| C | -5.915395 | -1.872488 | 1.827658 | C | -6.029513 | -1.67709 | -1.65606 |
| C | 1.150252 | 4.499808 | 2.232521 | C | 3.472907 | -2.200592 | -2.784489 |
| C | 0.219511 | 6.422017 | 0.385196 | C | 5.096104 | -3.711983 | -1.037467 |
| O | 2.334379 | 5.149721 | 4.108716 | O | 3.910805 | -2.161983 | -5.055261 |
| C | 4.966493 | 0.310529 | 2.667857 | C | 3.913135 | 3.304759 | -1.846542 |
| C | 1.652452 | -0.245861 | 5.990931 | C | 0.681481 | 2.596848 | -5.180225 |
| C | 6.068162 | 0.358999 | 0.379194 | C | 5.511715 | 2.571874 | -0.012236 |
| C | 8.734288 | 0.864043 | -0.02632 | C | 8.00789 | 3.646744 | 0.367159 |
| C | 9.989882 | 0.788517 | -2.246801 | C | 9.793991 | 2.807158 | 1.985093 |
| C | 12.77925 | 1.341103 | -2.355803 | C | 12.323972 | 4.099487 | 2.149078 |
| C | 8.80327 | 0.153058 | -4.754113 | C | 9.492446 | 0.556866 | 3.700486 |
| C | 3.057804 | -5.106711 | 3.01581 | C | -0.846771 | 6.360134 | -0.904232 |
| C | 2.822336 | -7.473143 | 1.442585 | C | -2.339551 | 7.570207 | 1.190535 |
| C | 4.151218 | -8.020187 | -0.646997 | C | -4.672611 | 8.557332 | 1.083098 |
| C | 3.673063 | -10.417263 | -2.112432 | C | -5.902945 | 9.656244 | 3.408962 |
| C | 6.183186 | -6.340029 | -1.729496 | C | -6.281784 | 8.664683 | -1.265962 |
| C | -9.817033 | -2.11308 | -1.243546 | C | -9.223351 | -4.433553 | 0.914852 |
| C | -11.783007 | -1.865258 | 0.854338 | C | -10.333451 | -2.245684 | 2.451483 |
| O | -10.814472 | -0.747319 | -3.36729 | O | -10.513711 | -4.69088 | -1.458368 |
| C | -9.394732 | -4.881633 | -1.983617 | C | -9.3691 | -6.909585 | 2.409355 |
| O | -3.628393 | 2.652754 | 3.245234 | O | -1.571028 | -3.578939 | -3.718624 |
| O | -1.430409 | -1.263788 | -3.1855 | O | -1.783242 | -0.931516 | 3.547161 |
| C | -0.901768 | 5.786045 | -1.934407 | C | 7.507385 | -4.415298 | -1.891682 |
| C | -1.742558 | 7.67585 | -3.565821 | C | 9.12956 | -5.780207 | -0.337989 |
| C | -1.476658 | 10.215071 | -2.901733 | C | 8.344883 | -6.49818 | 2.081017 |
| C | -0.357752 | 10.866668 | -0.597832 | C | 5.937601 | -5.838793 | 2.936062 |
| C | 0.485607 | 8.983261 | 1.028712 | C | 4.323559 | -4.436493 | 1.395975 |
| H | 0.907949 | 1.374001 | -0.316813 | H | 1.4375 | -0.718173 | 0.408665 |
| H | -1.73458 | -5.212711 | 1.04819 | H | -4.61425 | 3.088485 | 0.459117 |
| H | -1.83938 | -3.873236 | 4.079121 | H | -4.083891 | 2.842012 | -2.798535 |
| H | 1.973403 | -2.755973 | -0.081963 | H | -0.075007 | 3.075289 | 1.338724 |
| H | -7.791923 | 1.21959 | -0.135015 | H | -5.713116 | -5.663477 | -0.749811 |
| H | -6.452922 | -0.958726 | 3.594044 | H | -6.007759 | -2.251618 | -3.633002 |
| H | -6.229658 | -3.906192 | 2.00486 | H | -7.488624 | -0.239271 | -1.404193 |
| H | 6.109762 | 0.707204 | 4.3319 | H | 4.500143 | 4.821608 | -3.109824 |
| H | 2.803173 | -1.666973 | 6.955819 | H | -1.174811 | 1.831568 | -5.675989 |
| H | 2.1086 | 1.605594 | 6.771635 | H | 0.678756 | 4.613058 | -5.639032 |
| H | -0.331373 | -0.647314 | 6.415775 | H | 2.081702 | 1.653971 | -6.362043 |
| H | 4.919736 | -0.024255 | -1.282977 | H | 4.956686 | 1.055866 | 1.253219 |
| H | 9.812076 | 1.322255 | 1.669384 | H | 8.469014 | 5.265765 | -0.821369 |
| H | 13.825777 | -0.277344 | -3.129513 | H | 13.861324 | 2.773192 | 1.712802 |
| H | 13.168398 | 2.943313 | -3.618661 | H | 12.461378 | 5.702649 | 0.850445 |
| H | 13.554212 | 1.782856 | -0.490395 | H | 12.679499 | 4.789101 | 4.075491 |
| H | 9.087999 | 1.703135 | -6.104915 | H | 9.846312 | 1.095179 | 5.673455 |
| H | 9.719043 | -1.51735 | -5.579284 | H | 7.633743 | -0.328438 | 3.596689 |
| H | 6.779587 | -0.229526 | -4.638766 | H | 10.895577 | -0.897042 | 3.22612 |
| H | 2.433117 | -5.506014 | 4.950334 | H | -1.638481 | 6.826365 | -2.756532 |
| H | 5.0331 | -4.507012 | 3.142461 | H | 1.083244 | 7.116824 | -0.872483 |
| H | 1.381216 | -8.819153 | 2.03704 | H | -1.391719 | 7.569682 | 3.022245 |
| H | 5.390767 | -11.578902 | -2.222658 | H | -6.387274 | 11.654928 | 3.121703 |
| H | 2.17321 | -11.557455 | -1.258885 | H | -7.684218 | 8.678868 | 3.837467 |
| H | 3.128574 | -9.992481 | -4.070903 | H | -4.675746 | 9.519853 | 5.06807 |
| H | 6.594471 | -4.695427 | -0.555039 | H | -8.028619 | 7.576666 | -0.992017 |
| H | 7.945582 | -7.403773 | -1.995679 | H | -6.863158 | 10.616417 | -1.666498 |
| H | 5.629751 | -5.648754 | -3.60806 | H | -5.333824 | 7.921862 | -2.941387 |
| H | -13.581054 | -2.655264 | 0.208416 | H | -12.291055 | -2.683362 | 2.964145 |
| H | -11.192515 | -2.887125 | 2.551521 | H | -9.267922 | -1.947985 | 4.197231 |
| H | -12.081227 | 0.122558 | 1.344608 | H | -10.339928 | -0.485059 | 1.369857 |
| H | -9.545816 | -0.831782 | -4.693018 | H | -12.262225 | -5.103086 | -1.084495 |
| H | -7.956733 | -5.039378 | -3.462657 | H | -11.332304 | -7.319365 | 2.926779 |
| H | -8.798351 | -6.037526 | -0.37636 | H | -8.651801 | -8.478995 | 1.267934 |
| H | -11.168134 | -5.656209 | -2.70893 | H | -8.258251 | -6.792549 | 4.149757 |
| H | -1.108819 | 3.831783 | -2.512053 | H | 8.081792 | -3.846853 | -3.77518 |
| H | -2.601227 | 7.162485 | -5.356894 | H | 11.003008 | -6.286467 | -1.004612 |
| H | -2.136105 | 11.683171 | -4.175155 | H | 9.607696 | -7.566651 | 3.295478 |
| H | -0.148738 | 12.840366 | -0.076832 | H | 5.317054 | -6.406616 | 4.807221 |
| H | 1.355493 | 9.452469 | 2.823929 | H | 2.464394 | -3.943189 | 2.105049 |
| **2A-11** | | | | **2A-12** | | | |
| Atom | X | Y | Z | Atom | X | Y | Z |
| C | -0.678157 | 1.539976 | -0.631991 | C | -0.215389 | 1.175793 | -0.725987 |
| C | 1.922316 | 1.322411 | -1.824151 | C | 2.404117 | 0.830516 | -1.838006 |
| C | 3.354719 | -1.123843 | -1.17831 | C | 3.768836 | -1.586208 | -0.977553 |
| C | 1.609863 | -3.429758 | -1.607972 | C | 1.983581 | -3.882973 | -1.257373 |
| C | -1.111573 | -3.159456 | -0.646725 | C | -0.760547 | -3.502711 | -0.386838 |
| C | -2.421743 | -0.676974 | -1.584422 | C | -1.99381 | -1.076802 | -1.55166 |
| C | 4.209921 | -0.882519 | 1.585798 | C | 4.573955 | -1.144915 | 1.777348 |
| O | 6.677164 | -0.270828 | 1.687161 | O | 7.050466 | -0.576688 | 1.88448 |
| C | 7.756799 | 0.027694 | -0.851067 | C | 8.19149 | -0.51012 | -0.643389 |
| C | 5.873957 | -1.305861 | -2.588343 | C | 6.311355 | -1.930847 | -2.314297 |
| C | -1.680881 | 4.226563 | -0.983124 | C | -1.157421 | 3.845275 | -1.299858 |
| C | -0.685502 | 6.232022 | 0.7414 | C | -0.344663 | 5.926075 | 0.430497 |
| O | -3.210221 | 4.754717 | -2.633379 | O | -2.485855 | 4.303619 | -3.135001 |
| C | -4.901026 | -0.419631 | -0.194708 | C | -4.498922 | -0.600593 | -0.272723 |
| C | -2.720631 | -0.648535 | -4.469905 | C | -2.210696 | -1.262507 | -4.438557 |
| C | -7.220071 | -0.192882 | -1.202034 | C | -6.710777 | -0.031269 | -1.37885 |
| C | -9.500109 | 0.008882 | 0.315223 | C | -9.023968 | 0.351126 | 0.048177 |
| C | -11.897952 | 0.244296 | -0.524125 | C | -11.351073 | 0.850356 | -0.872275 |
| C | -14.065332 | 0.432796 | 1.310998 | C | -13.569553 | 1.165585 | 0.882704 |
| C | -12.64923 | 0.341604 | -3.266936 | C | -11.967352 | 1.133681 | -3.635812 |
| C | -2.63285 | -5.565672 | -1.308484 | C | -2.226164 | -5.984351 | -0.905869 |
| C | -1.744164 | -7.853163 | 0.143392 | C | -4.856725 | -6.102676 | 0.182742 |
| C | -2.409645 | -8.463966 | 2.512506 | C | -5.462202 | -6.583318 | 2.596314 |
| C | -1.339147 | -10.768453 | 3.802006 | C | -8.176206 | -6.545314 | 3.462696 |
| C | -4.229889 | -6.942585 | 4.092227 | C | -3.550043 | -7.103278 | 4.642794 |
| C | 10.440506 | -1.083756 | -0.792944 | C | 10.842052 | -1.682472 | -0.433583 |
| C | 12.090001 | 0.349749 | 1.109517 | C | 12.486486 | -0.150158 | 1.394663 |
| O | 10.306553 | -3.704115 | -0.121776 | O | 10.621244 | -4.239548 | 0.431065 |
| C | 11.585856 | -0.982236 | -3.440777 | C | 12.050504 | -1.813256 | -3.051666 |
| O | 2.809201 | 2.927539 | -3.213479 | O | 3.360458 | 2.316993 | -3.310842 |
| O | 2.944278 | -1.130744 | 3.478423 | O | 3.264298 | -1.219788 | 3.654933 |
| C | -1.500877 | 8.724641 | 0.32875 | C | -1.218518 | 8.376795 | -0.099864 |
| C | -0.629806 | 10.687766 | 1.841943 | C | -0.515895 | 10.407247 | 1.41158 |
| C | 1.078544 | 10.188014 | 3.795623 | C | 1.086358 | 10.020035 | 3.477693 |
| C | 1.902161 | 7.719185 | 4.224702 | C | 1.972334 | 7.59573 | 4.020232 |
| C | 1.026017 | 5.748373 | 2.711198 | C | 1.259644 | 5.555664 | 2.512242 |
| H | -0.431661 | 1.187164 | 1.390922 | H | -0.021341 | 0.96171 | 1.320161 |
| H | 2.484663 | -5.081031 | -0.72033 | H | 2.810093 | -5.471082 | -0.218167 |
| H | 1.594341 | -3.801685 | -3.645798 | H | 2.010362 | -4.425291 | -3.256389 |
| H | -1.035132 | -3.040033 | 1.418927 | H | -0.737691 | -3.225844 | 1.665002 |
| H | 7.830292 | 2.059244 | -1.221375 | H | 8.328705 | 1.482681 | -1.171997 |
| H | 5.783754 | -0.429317 | -4.450209 | H | 6.278987 | -1.196943 | -4.238803 |
| H | 6.411472 | -3.291674 | -2.78093 | H | 6.805724 | -3.9369 | -2.344179 |
| H | -4.723994 | -0.444985 | 1.861994 | H | -4.435365 | -0.722289 | 1.783803 |
| H | -0.891272 | -0.821396 | -5.419119 | H | -3.50099 | -2.785163 | -4.973925 |
| H | -3.901695 | -2.223741 | -5.096818 | H | -2.91163 | 0.506331 | -5.223328 |
| H | -3.582968 | 1.115997 | -5.087243 | H | -0.376517 | -1.651388 | -5.311657 |
| H | -7.418405 | -0.150999 | -3.244165 | H | -6.803373 | 0.106732 | -3.423685 |
| H | -9.203577 | -0.039896 | 2.354412 | H | -8.825378 | 0.185222 | 2.093304 |
| H | -15.42972 | -1.103337 | 1.007235 | H | -15.057723 | -0.2186 | 0.45405 |
| H | -13.425488 | 0.352466 | 3.275657 | H | -13.025695 | 0.93806 | 2.864846 |
| H | -15.114311 | 2.204685 | 1.039941 | H | -14.436357 | 3.039662 | 0.656933 |
| H | -13.96922 | -1.198283 | -3.709668 | H | -13.405396 | -0.252184 | -4.203269 |
| H | -13.656864 | 2.109496 | -3.677553 | H | -12.783844 | 3.005965 | -4.007603 |
| H | -11.05195 | 0.203847 | -4.565274 | H | -10.332331 | 0.896681 | -4.871621 |
| H | -2.472886 | -5.942061 | -3.339295 | H | -1.072833 | -7.526498 | -0.137355 |
| H | -4.633454 | -5.193778 | -0.93857 | H | -2.307042 | -6.311171 | -2.949177 |
| H | -0.390837 | -9.073512 | -0.815918 | H | -6.391602 | -5.697725 | -1.121649 |
| H | -2.850561 | -12.074966 | 4.368478 | H | -8.45649 | -5.117429 | 4.945377 |
| H | -0.021739 | -11.792745 | 2.57999 | H | -9.471271 | -6.112349 | 1.909264 |
| H | -0.335226 | -10.245974 | 5.543094 | H | -8.731344 | -8.366899 | 4.291498 |
| H | -5.775039 | -8.141675 | 4.787145 | H | -3.537781 | -5.563698 | 6.036989 |
| H | -3.273928 | -6.186729 | 5.773196 | H | -4.035514 | -8.832281 | 5.683281 |
| H | -5.059797 | -5.352334 | 3.070901 | H | -1.626385 | -7.308055 | 3.919727 |
| H | 11.277893 | 0.252987 | 3.009638 | H | 12.729881 | 1.793019 | 0.724725 |
| H | 13.979349 | -0.48832 | 1.156195 | H | 11.628553 | -0.078652 | 3.27567 |
| H | 12.265848 | 2.344653 | 0.586293 | H | 14.35021 | -1.030961 | 1.552412 |
| H | 9.576782 | -3.782674 | 1.562667 | H | 9.867515 | -4.170251 | 2.105213 |
| H | 13.511937 | -1.731751 | -3.388869 | H | 13.951842 | -2.60992 | -2.894682 |
| H | 10.483762 | -2.117756 | -4.770481 | H | 10.946872 | -3.014786 | -4.320727 |
| H | 11.659875 | 0.964911 | -4.133893 | H | 12.19788 | 0.073507 | -3.88557 |
| H | -2.819785 | 9.077284 | -1.199413 | H | -2.451109 | 8.642358 | -1.715193 |
| H | -1.274182 | 12.606369 | 1.503557 | H | -1.20868 | 12.291111 | 0.985042 |
| H | 1.763065 | 11.718677 | 4.979011 | H | 1.640356 | 11.603742 | 4.659598 |
| H | 3.225245 | 7.322392 | 5.741583 | H | 3.216998 | 7.287173 | 5.621657 |
| H | 1.687163 | 3.849845 | 3.10293 | H | 1.970092 | 3.695085 | 2.990197 |
| **2A-13** | | | | **2A-14** | | | |
| Atom | X | Y | Z | Atom | X | Y | Z |
| C | -0.215189 | 1.175198 | -0.726864 | C | -1.218845 | -1.452028 | 0.428722 |
| C | 2.404539 | 0.831079 | -1.8387 | C | 1.367926 | -1.474474 | 1.671442 |
| C | 3.77058 | -1.584515 | -0.977313 | C | 3.198823 | 0.570234 | 0.712911 |
| C | 1.986599 | -3.882418 | -1.256702 | C | 1.859899 | 3.167555 | 0.70524 |
| C | -0.757812 | -3.503588 | -0.386464 | C | -0.85121 | 3.192953 | -0.319925 |
| C | -1.992442 | -1.078678 | -1.551841 | C | -2.572734 | 1.148984 | 0.952815 |
| C | 4.57515 | -1.1422 | 1.777597 | C | 4.045176 | -0.249674 | -1.940119 |
| O | 7.051462 | -0.573362 | 1.885009 | O | 6.378077 | -1.259686 | -1.840712 |
| C | 8.192832 | -0.506741 | -0.642685 | C | 7.35509 | -1.312917 | 0.75525 |
| C | 6.313405 | -1.928275 | -2.31372 | C | 5.689965 | 0.565856 | 2.183475 |
| C | -1.158927 | 3.843898 | -1.3015 | C | -2.659181 | -3.845001 | 1.16766 |
| C | -0.34956 | 5.925397 | 0.429593 | C | -2.004098 | -6.237695 | -0.186346 |
| O | -2.486073 | 4.301123 | -3.137869 | O | -4.261117 | -3.843429 | 2.833486 |
| C | -4.497662 | -0.603225 | -0.272788 | C | -5.054318 | 1.081392 | -0.453761 |
| C | -2.209523 | -1.265363 | -4.438649 | C | -2.888785 | 1.63754 | 3.795657 |
| C | -6.709573 | -0.033866 | -1.378799 | C | -7.386879 | 1.28738 | 0.526621 |
| C | -9.022681 | 0.348554 | 0.048375 | C | -9.669162 | 1.203684 | -0.998274 |
| C | -11.349752 | 0.848154 | -0.871939 | C | -12.07971 | 1.37981 | -0.180611 |
| C | -13.568128 | 1.163695 | 0.883135 | C | -14.248565 | 1.250814 | -2.01918 |
| C | -11.966209 | 1.131572 | -3.635431 | C | -12.84495 | 1.708169 | 2.540361 |
| C | -2.221897 | -5.986193 | -0.905285 | C | -1.951286 | 5.89124 | -0.109043 |
| C | -4.852909 | -6.105463 | 0.182111 | C | -0.68286 | 7.696775 | -1.899861 |
| C | -5.459368 | -6.585973 | 2.595461 | C | 0.971134 | 9.540621 | -1.357345 |
| C | -8.173812 | -6.548935 | 3.460512 | C | 2.098159 | 11.148354 | -3.425318 |
| C | -3.547978 | -7.104864 | 4.64293 | C | 1.874733 | 10.189738 | 1.266005 |
| C | 10.84371 | -1.6783 | -0.43236 | C | 10.184811 | -0.665907 | 0.633616 |
| C | 12.487403 | -0.145365 | 1.396031 | C | 11.605022 | -2.626551 | -0.958388 |
| O | 10.623479 | -4.235371 | 0.432424 | O | 10.489229 | 1.802653 | -0.43805 |
| C | 12.052598 | -1.808923 | -3.05025 | C | 11.26142 | -0.527328 | 3.308315 |
| O | 3.36009 | 2.317568 | -3.312038 | O | 1.955232 | -2.960829 | 3.326581 |
| O | 3.265188 | -1.216912 | 3.654997 | O | 2.867665 | -0.111664 | -3.899714 |
| C | 1.253401 | 5.556449 | 2.512644 | C | -0.209571 | -6.368307 | -2.137297 |
| C | 1.962713 | 7.597105 | 4.021432 | C | 0.333212 | -8.673616 | -3.291941 |
| C | 1.074691 | 10.020547 | 3.478391 | C | -0.909408 | -10.868578 | -2.52026 |
| C | -0.526175 | 10.406319 | 1.410924 | C | -2.702616 | -10.756979 | -0.581981 |
| C | -1.225446 | 8.375289 | -0.101297 | C | -3.241092 | -8.460174 | 0.574416 |
| H | -0.021052 | 0.96167 | 1.319316 | H | -0.896713 | -1.479852 | -1.613822 |
| H | 2.813991 | -5.46981 | -0.217109 | H | 3.009444 | 4.498106 | -0.382224 |
| H | 2.013841 | -4.425123 | -3.255611 | H | 1.869978 | 3.85411 | 2.659357 |
| H | -0.735326 | -3.226348 | 1.665329 | H | -0.763863 | 2.715404 | -2.335152 |
| H | 8.329522 | 1.486039 | -1.171524 | H | 7.090343 | -3.243802 | 1.441572 |
| H | 6.280983 | -1.194443 | -4.238251 | H | 5.424725 | 0.023537 | 4.152826 |
| H | 6.80856 | -3.93413 | -2.343461 | H | 6.545044 | 2.444531 | 2.089504 |
| H | -4.434042 | -0.725061 | 1.783724 | H | -4.863981 | 0.835507 | -2.494654 |
| H | -3.499518 | -2.78851 | -4.973353 | H | -4.018049 | 0.149033 | 4.65983 |
| H | -2.910951 | 0.503039 | -5.223957 | H | -1.061374 | 1.680826 | 4.76272 |
| H | -0.375309 | -1.6541 | -5.311748 | H | -3.815697 | 3.454656 | 4.124656 |
| H | -6.802226 | 0.10436 | -3.423617 | H | -7.596529 | 1.516972 | 2.554862 |
| H | -8.824002 | 0.182504 | 2.093482 | H | -9.363996 | 0.967704 | -3.023067 |
| H | -15.056726 | -0.219955 | 0.454228 | H | -15.403825 | 2.974248 | -1.925555 |
| H | -13.024329 | 0.935628 | 2.865235 | H | -13.598547 | 1.011089 | -3.967454 |
| H | -14.434344 | 3.038084 | 0.657738 | H | -15.518539 | -0.326533 | -1.557992 |
| H | -13.404402 | -0.254232 | -4.202721 | H | -14.069041 | 0.142563 | 3.14036 |
| H | -12.782737 | 3.003858 | -4.007155 | H | -11.246726 | 1.800346 | 3.841571 |
| H | -10.331344 | 0.894468 | -4.871412 | H | -13.961087 | 3.443045 | 2.772869 |
| H | -1.068102 | -7.527469 | -0.13573 | H | -1.80469 | 6.546986 | 1.846504 |
| H | -2.301679 | -6.313698 | -2.948526 | H | -3.974243 | 5.799309 | -0.552488 |
| H | -6.387316 | -5.701351 | -1.123106 | H | -1.149063 | 7.394937 | -3.886269 |
| H | -8.7286 | -8.370557 | 4.28947 | H | 4.169511 | 11.002432 | -3.432816 |
| H | -8.455406 | -5.120814 | 4.942717 | H | 1.404822 | 10.598106 | -5.294525 |
| H | -9.468306 | -6.116862 | 1.906358 | H | 1.654758 | 13.155905 | -3.133402 |
| H | -4.033072 | -8.834099 | 5.683212 | H | 3.929115 | 9.925227 | 1.406228 |
| H | -1.623853 | -7.308652 | 3.920826 | H | 1.510339 | 12.188212 | 1.691772 |
| H | -3.537231 | -5.565247 | 6.037093 | H | 0.990723 | 9.049354 | 2.740938 |
| H | 12.73032 | 1.797838 | 0.725995 | H | 11.444735 | -4.51541 | -0.127305 |
| H | 11.629161 | -0.073979 | 3.276904 | H | 10.846981 | -2.704621 | -2.881558 |
| H | 14.351366 | -1.0256 | 1.554126 | H | 13.606538 | -2.118415 | -1.05798 |
| H | 9.86979 | -4.166175 | 2.106591 | H | 9.799598 | 1.729237 | -2.139521 |
| H | 13.95414 | -2.605025 | -2.892908 | H | 11.014112 | -2.329231 | 4.292902 |
| H | 10.949506 | -3.010872 | -4.319389 | H | 13.283004 | -0.103925 | 3.216843 |
| H | 12.199539 | 0.077819 | -3.884272 | H | 10.336199 | 0.960278 | 4.405158 |
| H | 1.965542 | 3.696588 | 2.990881 | H | 0.774249 | -4.694769 | -2.790273 |
| H | 3.206357 | 7.289675 | 5.623868 | H | 1.724651 | -8.751397 | -4.797546 |
| H | 1.626036 | 11.604697 | 4.660945 | H | -0.484259 | -12.66085 | -3.425214 |
| H | -1.220489 | 12.289524 | 0.983948 | H | -3.672207 | -12.461391 | 0.022713 |
| H | -2.456944 | 8.639741 | -1.71764 | H | -4.619693 | -8.339061 | 2.085742 |
| **2A-15** | | | | **2A-16** | | | |
| Atom | X | Y | Z | Atom | X | Y | Z |
| C | 1.77132 | 1.480555 | 0.548557 | C | -0.501649 | 0.606831 | -1.194127 |
| C | -0.632612 | 2.348965 | 1.85946 | C | 2.270823 | 0.694966 | -1.918117 |
| C | -3.074777 | 1.054295 | 0.954108 | C | 4.057796 | -0.54419 | 0.028941 |
| C | -2.637704 | -1.85251 | 0.980397 | C | 3.042053 | -3.153533 | 0.879412 |
| C | -0.153559 | -2.759907 | -0.190295 | C | 0.189895 | -3.263179 | 1.380439 |
| C | 2.204683 | -1.432135 | 1.003751 | C | -1.362576 | -2.233636 | -0.920739 |
| C | -3.685293 | 1.9156 | -1.750157 | C | 4.243567 | 1.338935 | 2.234815 |
| O | -6.060497 | 2.793287 | -1.900647 | O | 6.426677 | 2.637888 | 2.095682 |
| C | -7.348688 | 2.802037 | 0.524837 | C | 7.913444 | 1.899713 | -0.1266 |
| C | -5.41107 | 1.854197 | 2.476892 | C | 6.786392 | -0.635422 | -0.930769 |
| C | 3.948634 | 3.239302 | 1.268176 | C | -2.131558 | 2.191264 | -2.96937 |
| C | 4.084577 | 5.751431 | -0.02059 | C | -3.230264 | 4.595952 | -1.976363 |
| O | 5.525514 | 2.655027 | 2.854206 | O | -2.563623 | 1.513349 | -5.137838 |
| C | 4.5008 | -2.1719 | -0.520119 | C | -4.163197 | -2.362025 | -0.337756 |
| C | 2.465252 | -2.056506 | 3.825688 | C | -0.85642 | -3.693734 | -3.389071 |
| C | 6.653442 | -3.206295 | 0.340421 | C | -5.525629 | -0.620894 | 0.913983 |
| C | 8.76085 | -3.865743 | -1.292306 | C | -8.221558 | -0.83143 | 1.383174 |
| C | 10.990148 | -4.893777 | -0.596825 | C | -9.733673 | 0.961733 | 2.388054 |
| C | 12.995373 | -5.459307 | -2.535963 | C | -12.516652 | 0.481765 | 2.738825 |
| C | 11.699329 | -5.56188 | 2.076841 | C | -8.858853 | 3.545965 | 3.197825 |
| C | -0.035359 | -5.677879 | -0.058015 | C | -0.628385 | -5.884783 | 2.396507 |
| C | -1.943253 | -6.872717 | -1.795058 | C | -0.092198 | -8.146823 | 0.7633 |
| C | -4.165922 | -7.931833 | -1.186801 | C | -1.748737 | -9.829324 | -0.16262 |
| C | -5.904757 | -8.946869 | -3.205626 | C | -0.860147 | -12.024271 | -1.751139 |
| C | -5.163055 | -8.197568 | 1.469251 | C | -4.571299 | -9.716989 | 0.209193 |
| C | -9.815103 | 1.269949 | 0.289606 | C | 10.707525 | 1.853903 | 0.668396 |
| C | -9.365103 | -1.51732 | -0.347555 | C | 11.556254 | 4.482986 | 1.541916 |
| O | -10.885314 | 1.512259 | 2.770694 | O | 11.038038 | 0.056688 | 2.667331 |
| C | -11.549449 | 2.500775 | -1.675696 | C | 12.323645 | 0.923721 | -1.53506 |
| O | -0.624777 | 3.901389 | 3.55681 | O | 3.071731 | 1.68087 | -3.83316 |
| O | -2.322712 | 1.843078 | -3.593957 | O | 2.705804 | 1.727882 | 3.886781 |
| C | 2.348047 | 6.538978 | -1.866218 | C | -2.274066 | 5.855195 | 0.155063 |
| C | 2.573888 | 8.921116 | -2.970181 | C | -3.396584 | 8.088434 | 0.989752 |
| C | 4.531482 | 10.536877 | -2.252499 | C | -5.499351 | 9.065755 | -0.268804 |
| C | 6.271285 | 9.767199 | -0.41783 | C | -6.464439 | 7.821374 | -2.390778 |
| C | 6.044996 | 7.394969 | 0.688669 | C | -5.324235 | 5.613906 | -3.248237 |
| H | 1.422265 | 1.657768 | -1.481279 | H | -0.604637 | 1.390712 | 0.710705 |
| H | -4.20838 | -2.771555 | 0.007982 | H | 4.064941 | -3.722462 | 2.587838 |
| H | -2.744445 | -2.454966 | 2.957835 | H | 3.565188 | -4.509193 | -0.592263 |
| H | -0.17058 | -2.238536 | -2.197107 | H | -0.208168 | -1.954489 | 2.934093 |
| H | -7.868094 | 4.767726 | 0.882759 | H | 7.598337 | 3.359398 | -1.553805 |
| H | -4.902556 | 3.366725 | 3.781431 | H | 6.8817 | -0.910255 | -2.970226 |
| H | -6.172694 | 0.295557 | 3.589215 | H | 7.797804 | -2.164419 | 0.022599 |
| H | 4.326409 | -1.808708 | -2.544868 | H | -5.117736 | -4.056872 | -1.013676 |
| H | 4.091284 | -1.083355 | 4.628896 | H | -1.426643 | -5.663776 | -3.17988 |
| H | 0.794346 | -1.46319 | 4.889096 | H | -1.911277 | -2.834948 | -4.937652 |
| H | 2.699119 | -4.090645 | 4.105336 | H | 1.145151 | -3.672327 | -3.91113 |
| H | 6.850858 | -3.563438 | 2.351367 | H | -4.599123 | 1.077117 | 1.595921 |
| H | 8.477368 | -3.468653 | -3.294974 | H | -9.08525 | -2.615322 | 0.818631 |
| H | 12.393397 | -4.942694 | -4.445549 | H | -13.645323 | 1.874899 | 1.69087 |
| H | 14.750696 | -4.435055 | -2.107663 | H | -13.063722 | -1.40805 | 2.102464 |
| H | 13.483878 | -7.477973 | -2.53101 | H | -13.05621 | 0.678591 | 4.734641 |
| H | 10.220121 | -5.142143 | 3.452242 | H | -9.83624 | 5.009579 | 2.097883 |
| H | 12.152783 | -7.582624 | 2.220732 | H | -9.341823 | 3.885426 | 5.187723 |
| H | 13.412666 | -4.537907 | 2.647448 | H | -6.833698 | 3.853036 | 2.96471 |
| H | -0.307303 | -6.295434 | 1.896733 | H | -2.63257 | -5.77684 | 2.881676 |
| H | 1.87013 | -6.275964 | -0.610582 | H | 0.382545 | -6.133596 | 4.196895 |
| H | -1.462478 | -6.761021 | -3.797393 | H | 1.897181 | -8.460909 | 0.327295 |
| H | -5.108986 | -8.710218 | -5.099783 | H | -1.723394 | -11.96729 | -3.639161 |
| H | -6.295522 | -10.964722 | -2.911123 | H | 1.19518 | -12.027721 | -1.981472 |
| H | -7.741955 | -7.980077 | -3.149365 | H | -1.42242 | -13.835055 | -0.904833 |
| H | -5.599081 | -10.182394 | 1.890765 | H | -5.265754 | -11.475647 | 1.064844 |
| H | -3.853346 | -7.509957 | 2.907603 | H | -5.183341 | -8.146031 | 1.3979 |
| H | -6.943519 | -7.150153 | 1.67956 | H | -5.530952 | -9.539196 | -1.623821 |
| H | -8.359376 | -1.729115 | -2.142407 | H | 11.380163 | 5.867072 | 0.012967 |
| H | -8.291684 | -2.462693 | 1.142215 | H | 10.410395 | 5.136147 | 3.135655 |
| H | -11.183144 | -2.491602 | -0.530134 | H | 13.534434 | 4.408493 | 2.13825 |
| H | -12.471945 | 0.591123 | 2.773215 | H | 10.015157 | 0.641124 | 4.077243 |
| H | -13.360328 | 1.499205 | -1.752581 | H | 11.809558 | -1.005251 | -2.07072 |
| H | -11.915617 | 4.474875 | -1.176328 | H | 12.094302 | 2.153267 | -3.182218 |
| H | -10.700518 | 2.439051 | -3.559709 | H | 14.315911 | 0.93453 | -0.982612 |
| H | 0.816566 | 5.320099 | -2.469868 | H | -0.642271 | 5.132971 | 1.164273 |
| H | 1.220502 | 9.511534 | -4.39477 | H | -2.632483 | 9.058752 | 2.627821 |
| H | 4.703322 | 12.389593 | -3.11867 | H | -6.385761 | 10.790761 | 0.40195 |
| H | 7.795969 | 11.020053 | 0.145069 | H | -8.102996 | 8.575498 | -3.368975 |
| H | 7.371759 | 6.768576 | 2.119311 | H | -6.044703 | 4.625895 | -4.893045 |
| **2A-17** | | | | **2A-18** | | | |
| Atom | X | Y | Z | Atom | X | Y | Z |
| C | 0.018869 | -0.692116 | -1.798273 | C | -0.698877 | 1.558725 | -0.647095 |
| C | -2.793297 | -0.757168 | -2.306141 | C | 1.902709 | 1.371355 | -1.84319 |
| C | -4.30264 | 1.135366 | -0.682849 | C | 3.362915 | -1.060461 | -1.202098 |
| C | -3.064493 | 3.778719 | -0.82979 | C | 1.640802 | -3.385263 | -1.625586 |
| C | -0.163325 | 3.890581 | -0.65232 | C | -1.07972 | -3.145725 | -0.655368 |
| C | 1.142746 | 1.981021 | -2.49575 | C | -2.420442 | -0.678485 | -1.590567 |
| C | -4.325249 | 0.074875 | 2.022347 | C | 4.2136 | -0.804821 | 1.564274 |
| O | -6.564869 | -1.021146 | 2.512111 | O | 6.679383 | -0.20383 | 1.672578 |
| C | -8.265806 | -0.884851 | 0.328417 | C | 7.774872 | 0.045452 | -0.846616 |
| C | -7.114309 | 1.145646 | -1.376945 | C | 5.879165 | -1.222965 | -2.62517 |
| C | 1.413579 | -2.898838 | -3.025344 | C | -1.735099 | 4.232765 | -0.997244 |
| C | 2.441486 | -4.91709 | -1.340337 | C | -0.764167 | 6.250556 | 0.726921 |
| O | 1.719947 | -3.008843 | -5.315257 | O | -3.273741 | 4.741737 | -2.645048 |
| C | 3.978299 | 1.894908 | -2.057413 | C | -4.896585 | -0.448678 | -0.19053 |
| C | 0.633191 | 2.655815 | -5.276792 | C | -2.731299 | -0.656296 | -4.474817 |
| C | 5.168575 | 0.622566 | -0.20897 | C | -7.224 | -0.26301 | -1.18681 |
| C | 7.894197 | 0.59691 | 0.100208 | C | -9.498618 | -0.089416 | 0.342229 |
| C | 9.229347 | -0.772299 | 1.790361 | C | -11.904972 | 0.09977 | -0.484233 |
| C | 12.068248 | -0.598376 | 1.87965 | C | -14.064428 | 0.262776 | 1.362676 |
| C | 8.082801 | -2.575449 | 3.669249 | C | -12.673904 | 0.166402 | -3.223077 |
| C | 0.614494 | 6.692887 | -0.998023 | C | -2.575071 | -5.569586 | -1.31178 |
| C | 3.401808 | 7.283426 | -0.871508 | C | -1.651934 | -7.847216 | 0.134121 |
| C | 4.863609 | 7.205407 | 1.194774 | C | -2.296966 | -8.466738 | 2.506599 |
| C | 7.661239 | 7.708728 | 1.046906 | C | -1.192402 | -10.759078 | 3.788975 |
| C | 3.90785 | 6.436258 | 3.761447 | C | -4.125986 | -6.967169 | 4.096996 |
| C | -10.957643 | -0.474261 | 1.327907 | C | 10.464591 | -1.052462 | -0.789686 |
| C | -12.803167 | -0.494005 | -0.890392 | C | 10.52379 | -3.807428 | 0.097465 |
| O | -11.601278 | -2.624722 | 2.85524 | O | 11.229433 | -0.865808 | -3.386283 |
| C | -11.214007 | 1.943608 | 2.902351 | C | 12.162984 | 0.597146 | 0.877935 |
| O | -3.815263 | -2.151888 | -3.819377 | O | 2.771605 | 2.990862 | -3.226894 |
| O | -2.628722 | 0.114752 | 3.561043 | O | 2.937608 | -1.031063 | 3.453934 |
| C | 1.525961 | -5.358046 | 1.11091 | C | -1.617018 | 8.731517 | 0.319863 |
| C | 2.57752 | -7.263627 | 2.595675 | C | -0.771203 | 10.705049 | 1.833862 |
| C | 4.574577 | -8.716881 | 1.667409 | C | 0.949166 | 10.227617 | 3.782582 |
| C | 5.498837 | -8.286914 | -0.77056 | C | 1.810072 | 7.770541 | 4.205858 |
| C | 4.422947 | -6.415369 | -2.270531 | C | 0.959236 | 5.789151 | 2.691681 |
| H | 0.210653 | -0.81585 | 0.250704 | H | -0.442268 | 1.21109 | 1.375318 |
| H | -3.878587 | 4.95668 | 0.665371 | H | 2.537016 | -5.027314 | -0.741954 |
| H | -3.673244 | 4.620125 | -2.622135 | H | 1.621854 | -3.756664 | -3.663504 |
| H | 0.388356 | 3.310742 | 1.253807 | H | -0.998267 | -3.024586 | 1.409894 |
| H | -8.185105 | -2.743676 | -0.569862 | H | 7.889 | 2.071622 | -1.239201 |
| H | -7.414076 | 0.717973 | -3.369892 | H | 5.801797 | -0.265576 | -4.446025 |
| H | -7.915316 | 3.004625 | -0.965051 | H | 6.365852 | -3.203643 | -2.940971 |
| H | 5.115071 | 2.964806 | -3.395279 | H | -4.709201 | -0.461225 | 1.865409 |
| H | 1.471656 | 4.491862 | -5.727367 | H | -3.893716 | -2.247381 | -5.096491 |
| H | 1.457491 | 1.23305 | -6.519173 | H | -3.619194 | 1.096395 | -5.0897 |
| H | -1.389782 | 2.761565 | -5.695017 | H | -0.903319 | -0.805437 | -5.430812 |
| H | 4.068998 | -0.456346 | 1.145106 | H | -7.433945 | -0.234912 | -3.228066 |
| H | 8.941687 | 1.803557 | -1.200628 | H | -9.189534 | -0.120483 | 2.37991 |
| H | 12.713868 | 0.032112 | 3.750419 | H | -13.411577 | 0.206591 | 3.323894 |
| H | 12.936886 | -2.457897 | 1.5608 | H | -15.145751 | 2.014295 | 1.086447 |
| H | 12.807804 | 0.714643 | 0.463484 | H | -15.403577 | -1.29889 | 1.077291 |
| H | 8.673 | -2.089774 | 5.599077 | H | -11.082083 | 0.047468 | -4.529978 |
| H | 6.022372 | -2.627749 | 3.618383 | H | -13.969804 | -1.398745 | -3.648086 |
| H | 8.757259 | -4.506437 | 3.314325 | H | -13.714105 | 1.914043 | -3.639083 |
| H | -0.39907 | 7.761619 | 0.465082 | H | -2.419963 | -5.942828 | -3.343543 |
| H | -0.136563 | 7.373783 | -2.804087 | H | -4.578262 | -5.222118 | -0.932327 |
| H | 4.299301 | 7.773334 | -2.658599 | H | -0.28982 | -9.051335 | -0.833267 |
| H | 8.252436 | 8.217287 | -0.869072 | H | -0.184159 | -10.225527 | 5.524193 |
| H | 8.224774 | 9.237516 | 2.333823 | H | -2.685248 | -12.083087 | 4.36398 |
| H | 8.727475 | 6.022444 | 1.622375 | H | 0.12939 | -11.767814 | 2.558757 |
| H | 4.470572 | 4.475831 | 4.162908 | H | -4.978831 | -5.385512 | 3.081206 |
| H | 4.732985 | 7.616732 | 5.252802 | H | -5.65401 | -8.184073 | 4.798808 |
| H | 1.850303 | 6.540788 | 3.918723 | H | -3.169861 | -6.201964 | 5.773616 |
| H | -12.477404 | 1.118344 | -2.142339 | H | 9.803245 | -3.983284 | 2.026608 |
| H | -12.609693 | -2.237615 | -1.987302 | H | 9.407826 | -5.027178 | -1.142024 |
| H | -14.739603 | -0.384529 | -0.174501 | H | 12.475441 | -4.498606 | 0.077704 |
| H | -10.393312 | -2.67072 | 4.238319 | H | 12.961254 | -1.463908 | -3.486144 |
| H | -13.12467 | 2.030991 | 3.685317 | H | 14.106127 | -0.11892 | 0.851781 |
| H | -9.862469 | 1.951112 | 4.46869 | H | 12.174404 | 2.545848 | 0.183037 |
| H | -10.904107 | 3.647166 | 1.772198 | H | 11.505233 | 0.595409 | 2.838516 |
| H | -0.018217 | -4.247398 | 1.875291 | H | -2.944872 | 9.066895 | -1.204447 |
| H | 1.844683 | -7.603508 | 4.48144 | H | -1.444643 | 12.614462 | 1.500061 |
| H | 5.409985 | -10.179769 | 2.839325 | H | 1.614002 | 11.766472 | 4.96659 |
| H | 7.055094 | -9.411688 | -1.494 | H | 3.142805 | 7.391114 | 5.718752 |
| H | 5.112497 | -6.05955 | -4.167627 | H | 1.649217 | 3.899953 | 3.078572 |
| **2A-19** | | | |  |  |  |  |
| Atom | X | Y | Z |  |  |  |  |
| C | -0.042018 | 1.576706 | -0.173318 |  |  |  |  |
| C | 2.303552 | 0.416704 | -1.351957 |  |  |  |  |
| C | 3.350106 | -1.922371 | 0.029346 |  |  |  |  |
| C | 1.19025 | -3.801806 | 0.636316 |  |  |  |  |
| C | -1.25132 | -2.593024 | 1.625164 |  |  |  |  |
| C | -2.232615 | -0.441837 | -0.165737 |  |  |  |  |
| C | 4.65405 | -0.923294 | 2.424834 |  |  |  |  |
| O | 7.175014 | -0.868909 | 2.090793 |  |  |  |  |
| C | 7.886477 | -1.72567 | -0.449821 |  |  |  |  |
| C | 5.551071 | -3.127162 | -1.411514 |  |  |  |  |
| C | -0.605041 | 4.11606 | -1.430264 |  |  |  |  |
| C | 0.992163 | 6.326206 | -0.685273 |  |  |  |  |
| O | -2.273046 | 4.356348 | -3.011709 |  |  |  |  |
| C | -4.502684 | 0.753791 | 1.076634 |  |  |  |  |
| C | -2.776284 | -1.377261 | -2.860458 |  |  |  |  |
| C | -6.871825 | 0.824762 | 0.171821 |  |  |  |  |
| C | -8.987405 | 1.890106 | 1.559814 |  |  |  |  |
| C | -11.4379 | 1.913841 | 0.849502 |  |  |  |  |
| C | -13.424854 | 3.073832 | 2.524772 |  |  |  |  |
| C | -12.425571 | 0.816105 | -1.583218 |  |  |  |  |
| C | -3.237352 | -4.619889 | 2.353198 |  |  |  |  |
| C | -4.076495 | -6.421938 | 0.322383 |  |  |  |  |
| C | -6.439671 | -6.883373 | -0.475867 |  |  |  |  |
| C | -6.931852 | -8.819796 | -2.511127 |  |  |  |  |
| C | -8.780868 | -5.577015 | 0.48819 |  |  |  |  |
| C | 10.303399 | -3.316607 | -0.185894 |  |  |  |  |
| C | 12.453781 | -1.717897 | 0.916474 |  |  |  |  |
| O | 9.802605 | -5.439301 | 1.419946 |  |  |  |  |
| C | 11.033729 | -4.400445 | -2.759524 |  |  |  |  |
| O | 3.28411 | 1.235569 | -3.265725 |  |  |  |  |
| O | 3.673682 | -0.207088 | 4.366768 |  |  |  |  |
| C | 2.870791 | 6.205794 | 1.185457 |  |  |  |  |
| C | 4.311571 | 8.335151 | 1.761351 |  |  |  |  |
| C | 3.893306 | 10.604848 | 0.483938 |  |  |  |  |
| C | 2.0235 | 10.744799 | -1.378442 |  |  |  |  |
| C | 0.587739 | 8.621431 | -1.956141 |  |  |  |  |
| H | 0.393662 | 1.920854 | 1.819336 |  |  |  |  |
| H | 1.893198 | -5.17177 | 2.021056 |  |  |  |  |
| H | 0.817565 | -4.862989 | -1.09912 |  |  |  |  |
| H | -0.77433 | -1.659945 | 3.411976 |  |  |  |  |
| H | 8.264472 | -0.023206 | -1.557597 |  |  |  |  |
| H | 5.329251 | -2.95151 | -3.451864 |  |  |  |  |
| H | 5.694633 | -5.123626 | -0.898502 |  |  |  |  |
| H | -4.134895 | 1.55296 | 2.944369 |  |  |  |  |
| H | -1.087687 | -2.191185 | -3.733868 |  |  |  |  |
| H | -4.22566 | -2.84101 | -2.846496 |  |  |  |  |
| H | -3.394001 | 0.194988 | -4.039146 |  |  |  |  |
| H | -7.249889 | 0.02132 | -1.676896 |  |  |  |  |
| H | -8.513033 | 2.729719 | 3.381527 |  |  |  |  |
| H | -12.617543 | 3.834978 | 4.269725 |  |  |  |  |
| H | -14.418879 | 4.610671 | 1.543058 |  |  |  |  |
| H | -14.874023 | 1.675788 | 3.033991 |  |  |  |  |
| H | -10.951915 | 0.017505 | -2.785759 |  |  |  |  |
| H | -13.808008 | -0.681964 | -1.189235 |  |  |  |  |
| H | -13.430745 | 2.265349 | -2.677853 |  |  |  |  |
| H | -4.857601 | -3.651369 | 3.186749 |  |  |  |  |
| H | -2.375561 | -5.73205 | 3.88557 |  |  |  |  |
| H | -2.568455 | -7.518173 | -0.555799 |  |  |  |  |
| H | -7.844586 | -7.944261 | -4.158484 |  |  |  |  |
| H | -5.188372 | -9.732108 | -3.148344 |  |  |  |  |
| H | -8.229001 | -10.296124 | -1.840317 |  |  |  |  |
| H | -9.806681 | -4.690928 | -1.083752 |  |  |  |  |
| H | -10.084966 | -6.952565 | 1.335863 |  |  |  |  |
| H | -8.391595 | -4.108575 | 1.880994 |  |  |  |  |
| H | 14.145755 | -2.886751 | 1.129079 |  |  |  |  |
| H | 12.905342 | -0.123388 | -0.323606 |  |  |  |  |
| H | 11.935372 | -0.962644 | 2.771219 |  |  |  |  |
| H | 9.338106 | -4.761936 | 3.063579 |  |  |  |  |
| H | 9.552473 | -5.643102 | -3.490207 |  |  |  |  |
| H | 11.361789 | -2.883994 | -4.127001 |  |  |  |  |
| H | 12.773012 | -5.502814 | -2.575221 |  |  |  |  |
| H | 3.22845 | 4.476087 | 2.222617 |  |  |  |  |
| H | 5.75812 | 8.219281 | 3.211584 |  |  |  |  |
| H | 5.018351 | 12.26029 | 0.937537 |  |  |  |  |
| H | 1.693656 | 12.507638 | -2.375658 |  |  |  |  |
| H | -0.865094 | 8.693071 | -3.399527 |  |  |  |  |

**Table S7.** Gibbs free energies^a^ and equilibrium populations^b^ of low-energy conformers of **2B**.

| Conformers | ∆G(a.u.) | P(%)/100 | G(a.u.) |
| --- | --- | --- | --- |
| **2B-1** | 0.0005 | 17.34 | -1580.920686 |
| **2B-2** | 0.00281 | 1.49 | -1580.918372 |
| **2B-3** | 0.00854 | 0.0 | -1580.912644 |
| **2B-4** | 0.00271 | 1.66 | -1580.918472 |
| **2B-5** | 0.00087 | 11.62 | -1580.920309 |
| **2B-6** | 0.00229 | 2.59 | -1580.918891 |
| **2B-7** | 0.0035 | 0.72 | -1580.917682 |
| **2B-8** | 0.0019 | 3.93 | -1580.919285 |
| **2B-9** | 0.00899 | 0.0 | -1580.912189 |
| **2B-10** | 0.00188 | 4.01 | -1580.919305 |
| **2B-11** | 0.00029 | 21.55 | -1580.920891 |
| **2B-12** | 0.00271 | 1.66 | -1580.918471 |
| **2B-13** | 0.00266 | 1.76 | -1580.918527 |
| **2B-14** | 0.00266 | 1.76 | -1580.918527 |
| **2B-15** | 0.00841 | 0.0 | -1580.91277 |
| **2B-16** | 0.00388 | 0.48 | -1580.917298 |
| **2B-17** | 0.0 | 29.3 | -1580.921182 |
| **2B-18** | 0.00527 | 0.11 | -1580.915909 |
| **2B-19** | 0.01046 | 0.0 | -1580.910724 |

^a^wB97M-V/def2-TZVP, in a.u.
^b^From ∆G values at 298.15K.

**Table S8.** Cartesian coordinates for the low-energy reoptimized random research conformers of **2B** at B3LYP-D3(BJ)/6-31G* level of theory in methanol.

| **2B-1** | | | | **2B-2** | | | |
| --- | --- | --- | --- | --- | --- | --- | --- |
| Atom | X | Y | Z | Atom | X | Y | Z |
| C | -0.17824 | 1.288408 | 1.707787 | C | 0.033924 | 1.518542 | 1.708377 |
| C | -2.45311 | -0.39578 | 2.106899 | C | -2.37548 | 0.047814 | 2.198095 |
| C | -2.2131 | -3.05879 | 0.991534 | C | -2.39853 | -2.64765 | 1.115515 |
| C | 0.286808 | -4.22685 | 2.021612 | C | -0.0024 | -4.00632 | 2.168253 |
| C | 2.619882 | -2.51806 | 1.872248 | C | 2.476095 | -2.52006 | 1.954453 |
| C | 2.172278 | 0.125366 | 3.112683 | C | 2.289989 | 0.192297 | 3.118101 |
| C | -2.12357 | -2.93814 | -1.91222 | C | -2.2949 | -2.57206 | -1.78314 |
| O | -4.01488 | -4.29733 | -2.91245 | O | -4.28794 | -3.77682 | -2.787 |
| C | -5.91853 | -4.92908 | -1.02634 | C | -6.09362 | -4.6017 | -0.88771 |
| C | -4.50726 | -4.75329 | 1.510883 | C | -4.84228 | -4.09816 | 1.690349 |
| C | -0.80363 | 4.037585 | 2.346269 | C | -0.37069 | 4.319056 | 2.273197 |
| C | -2.60268 | 5.398362 | 0.649255 | C | -2.13227 | 5.73311 | 0.575852 |
| O | 0.139409 | 5.113183 | 4.162933 | O | 0.676303 | 5.388783 | 4.0348 |
| C | 4.441072 | 1.822231 | 2.643285 | C | 4.692366 | 1.664282 | 2.559256 |
| C | 1.691322 | -0.10367 | 5.97178 | C | 1.845529 | 0.097576 | 5.990181 |
| C | 5.405571 | 2.339885 | 0.350797 | C | 5.661336 | 2.01695 | 0.23737 |
| C | 7.621203 | 3.905123 | -0.05955 | C | 7.993861 | 3.373524 | -0.25922 |
| C | 8.78025 | 4.370661 | -2.28506 | C | 9.149353 | 3.663597 | -2.51612 |
| C | 11.09397 | 6.023504 | -2.39833 | C | 11.58967 | 5.112921 | -2.72179 |
| C | 7.942825 | 3.321309 | -4.79427 | C | 8.184379 | 2.602485 | -4.9742 |
| C | 4.949433 | -3.91098 | 2.956924 | C | 4.677671 | -4.08841 | 3.066115 |
| C | 5.725407 | -6.13955 | 1.356315 | C | 5.233052 | -6.42643 | 1.530712 |
| C | 7.172059 | -6.05676 | -0.72496 | C | 6.6472 | -6.53639 | -0.57157 |
| C | 7.74244 | -8.41609 | -2.21822 | C | 6.987574 | -8.98279 | -1.99308 |
| C | 8.327466 | -3.66975 | -1.76922 | C | 7.986644 | -4.2929 | -1.70996 |
| C | -8.17289 | -3.10374 | -1.37285 | C | -8.62663 | -3.24312 | -1.37127 |
| C | -9.14922 | -3.23421 | -4.08934 | C | -8.38916 | -0.36137 | -1.23192 |
| O | -7.3217 | -0.5563 | -1.02458 | O | -10.1862 | -4.17125 | 0.645621 |
| C | -10.2844 | -3.75426 | 0.497402 | C | -9.7084 | -4.06077 | -3.92929 |
| O | -4.29074 | 0.271285 | 3.337149 | O | -4.08932 | 0.871156 | 3.495326 |
| O | -0.56717 | -1.88112 | -3.2239 | O | -0.68544 | -1.62108 | -3.11415 |
| C | -3.33923 | 7.853464 | 1.340672 | C | -3.04043 | 4.74405 | -1.71277 |
| C | -5.02775 | 9.211028 | -0.14533 | C | -4.70789 | 6.143636 | -3.19619 |
| C | -6.00814 | 8.129583 | -2.34937 | C | -5.48811 | 8.538892 | -2.41177 |
| C | -5.28629 | 5.693014 | -3.05528 | C | -4.58309 | 9.543727 | -0.1414 |
| C | -3.58832 | 4.332919 | -1.57058 | C | -2.91277 | 8.152091 | 1.335895 |
| H | 0.280566 | 1.18733 | -0.30666 | H | 0.462945 | 1.326425 | -0.30306 |
| H | 0.649614 | -5.98054 | 0.986159 | H | 0.194054 | -5.8187 | 1.189707 |
| H | -0.07237 | -4.77102 | 3.987702 | H | -0.39617 | -4.45056 | 4.152346 |
| H | 3.007962 | -2.17831 | -0.1293 | H | 2.877112 | -2.27617 | -0.05842 |
| H | -6.51868 | -6.85657 | -1.44796 | H | -6.37558 | -6.62055 | -1.20688 |
| H | -3.87222 | -6.63162 | 2.084806 | H | -4.41572 | -5.8834 | 2.632693 |
| H | -5.67149 | -3.99128 | 3.029732 | H | -6.07615 | -3.00935 | 2.927492 |
| H | 5.328903 | 2.643725 | 4.30723 | H | 5.672962 | 2.465845 | 4.180552 |
| H | 3.342992 | -0.91711 | 6.912068 | H | 1.659986 | 2.009185 | 6.736823 |
| H | 1.33276 | 1.757931 | 6.778149 | H | 0.13376 | -0.9511 | 6.484081 |
| H | 0.065957 | -1.30888 | 6.397747 | H | 3.441675 | -0.81939 | 6.931198 |
| H | 4.504886 | 1.528376 | -1.31009 | H | 4.66826 | 1.227585 | -1.38093 |
| H | 8.426251 | 4.754617 | 1.636724 | H | 8.895925 | 4.210227 | 1.394072 |
| H | 10.78104 | 7.652631 | -3.64784 | H | 11.39032 | 6.719651 | -4.02262 |
| H | 11.63131 | 6.731923 | -0.53152 | H | 12.2171 | 5.83661 | -0.88925 |
| H | 12.70854 | 4.984446 | -3.19018 | H | 13.09831 | 3.917064 | -3.50111 |
| H | 7.559997 | 4.86082 | -6.13286 | H | 7.903097 | 4.12226 | -6.35975 |
| H | 9.457233 | 2.177671 | -5.63609 | H | 9.585795 | 1.311695 | -5.79873 |
| H | 6.253622 | 2.143284 | -4.67662 | H | 6.407848 | 1.570476 | -4.78968 |
| H | 4.534647 | -4.56126 | 4.879452 | H | 4.222225 | -4.63962 | 5.010195 |
| H | 6.497697 | -2.54823 | 3.11274 | H | 6.345366 | -2.86888 | 3.171505 |
| H | 4.972051 | -7.97064 | 1.923216 | H | 4.33451 | -8.16633 | 2.168816 |
| H | 7.083238 | -8.22991 | -4.1786 | H | 5.965028 | -10.5432 | -1.09995 |
| H | 9.788273 | -8.7572 | -2.31975 | H | 6.314543 | -8.80296 | -3.94938 |
| H | 6.846782 | -10.0876 | -1.39235 | H | 8.994418 | -9.50309 | -2.11129 |
| H | 10.37405 | -3.90127 | -2.02672 | H | 7.218372 | -3.86583 | -3.59159 |
| H | 7.547432 | -3.24609 | -3.64674 | H | 7.829419 | -2.58266 | -0.56841 |
| H | 8.010961 | -2.01884 | -0.57457 | H | 10.0021 | -4.70719 | -1.98339 |
| H | -9.7863 | -5.14724 | -4.55085 | H | -7.16346 | 0.364026 | -2.73138 |
| H | -7.66448 | -2.69202 | -5.41734 | H | -7.64515 | 0.253391 | 0.592607 |
| H | -10.7434 | -1.93689 | -4.3112 | H | -10.2556 | 0.498011 | -1.48963 |
| H | -7.03471 | -0.26322 | 0.769825 | H | -11.8459 | -3.41628 | 0.441793 |
| H | -10.9286 | -5.7046 | 0.244799 | H | -9.96842 | -6.11286 | -3.97807 |
| H | -9.66984 | -3.53218 | 2.460845 | H | -11.5463 | -3.16055 | -4.24749 |
| H | -11.8895 | -2.48982 | 0.183492 | H | -8.45025 | -3.51345 | -5.47608 |
| H | -2.5663 | 8.656662 | 3.060332 | H | -2.44002 | 2.902519 | -2.37905 |
| H | -5.58777 | 11.10584 | 0.408806 | H | -5.39225 | 5.363366 | -4.96604 |
| H | -7.332 | 9.184729 | -3.50977 | H | -6.79363 | 9.622291 | -3.56661 |
| H | -6.04546 | 4.842943 | -4.76081 | H | -5.18587 | 11.40686 | 0.471936 |
| H | -3.0569 | 2.450914 | -2.17187 | H | -2.19564 | 8.899756 | 3.104291 |
| **2B-3** | | | | **2B-4** | | | |
| Atom | X | Y | Z | Atom | X | Y | Z |
| C | -0.18541 | 1.054395 | 1.687323 | C | 0.102986 | 0.461176 | -1.65302 |
| C | -2.51577 | -0.58588 | 1.913057 | C | 2.584779 | -0.91052 | -1.97031 |
| C | -2.27595 | -3.21209 | 0.715505 | C | 2.85263 | -3.30066 | -0.36195 |
| C | 0.215521 | -4.46595 | 1.632153 | C | 0.512853 | -4.98957 | -0.93463 |
| C | 2.562157 | -2.76799 | 1.67381 | C | -2.08029 | -3.68153 | -0.88653 |
| C | 2.092352 | -0.22651 | 3.101577 | C | -2.15201 | -1.24572 | -2.57117 |
| C | -2.29475 | -2.86803 | -2.17669 | C | 2.924206 | -2.63532 | 2.467071 |
| O | -4.45827 | -3.75721 | -3.15917 | O | 5.06662 | -3.48482 | 3.520928 |
| C | -6.24164 | -4.55283 | -1.20115 | C | 6.927645 | -4.18039 | 1.614361 |
| C | -4.58544 | -4.89921 | 1.157229 | C | 5.350939 | -4.71393 | -0.77075 |
| C | -0.79452 | 3.777449 | 2.431773 | C | 0.25029 | 3.105922 | -2.81783 |
| C | -2.29189 | 5.320877 | 0.603161 | C | 1.843279 | 5.026303 | -1.49466 |
| O | -0.12327 | 4.670492 | 4.454982 | O | -0.88104 | 3.659509 | -4.75579 |
| C | 4.383142 | 1.487065 | 2.851544 | C | -4.58713 | 0.203561 | -2.11489 |
| C | 1.494165 | -0.66393 | 5.912804 | C | -1.90351 | -1.91429 | -5.39014 |
| C | 5.427515 | 2.186647 | 0.643692 | C | -5.19712 | 1.415227 | 0.030893 |
| C | 7.654568 | 3.77489 | 0.436137 | C | -7.60666 | 2.670034 | 0.413795 |
| C | 8.881596 | 4.420411 | -1.70656 | C | -8.44868 | 3.78816 | 2.547519 |
| C | 11.19455 | 6.075167 | -1.61139 | C | -11.0372 | 4.967085 | 2.6445 |
| C | 8.124282 | 3.582016 | -4.31838 | C | -6.97246 | 3.947182 | 4.975108 |
| C | 4.852189 | -4.25814 | 2.710612 | C | -4.07166 | -5.72312 | -1.56561 |
| C | 5.660578 | -6.36164 | 0.963609 | C | -6.79916 | -4.90105 | -1.68425 |
| C | 7.169802 | -6.12833 | -1.06105 | C | -8.29651 | -4.36859 | 0.286889 |
| C | 7.769155 | -8.36791 | -2.71834 | C | -10.9749 | -3.47929 | -0.08658 |
| C | 8.371217 | -3.67695 | -1.88023 | C | -7.40509 | -4.42152 | 2.98757 |
| C | -8.52316 | -2.63768 | -1.00573 | C | 8.867154 | -2.00516 | 1.412971 |
| C | -7.97396 | -0.11062 | -2.31613 | C | 10.01412 | -1.45335 | 4.007153 |
| O | -9.16341 | -2.20628 | 1.582519 | O | 7.587341 | 0.266597 | 0.680275 |
| C | -10.8544 | -3.87682 | -2.18121 | C | 10.94018 | -2.68255 | -0.49047 |
| O | -4.42719 | 0.1167 | 3.006333 | O | 4.206479 | -0.23024 | -3.46815 |
| O | -0.65626 | -1.93671 | -3.48161 | O | 1.300022 | -1.58742 | 3.701956 |
| C | -2.7589 | 4.568228 | -1.89886 | C | 3.078466 | 4.555519 | 0.804872 |
| C | -4.19563 | 6.09243 | -3.49797 | C | 4.564511 | 6.422869 | 1.920223 |
| C | -5.18083 | 8.376045 | -2.62053 | C | 4.823872 | 8.778562 | 0.763436 |
| C | -4.71706 | 9.145441 | -0.1347 | C | 3.59205 | 9.268763 | -1.52366 |
| C | -3.27909 | 7.631436 | 1.459774 | C | 2.116215 | 7.405272 | -2.64189 |
| H | 0.346648 | 1.024627 | -0.30864 | H | -0.16419 | 0.667463 | 0.385856 |
| H | 0.582729 | -6.11098 | 0.433539 | H | 0.513433 | -6.56012 | 0.414222 |
| H | -0.15315 | -5.19854 | 3.534043 | H | 0.837169 | -5.8303 | -2.8001 |
| H | 3.007193 | -2.27697 | -0.28497 | H | -2.44945 | -3.05753 | 1.049172 |
| H | -6.95868 | -6.3707 | -1.85403 | H | 7.860737 | -5.87691 | 2.324178 |
| H | -3.96065 | -6.86524 | 1.262704 | H | 4.991507 | -6.7405 | -0.92818 |
| H | -5.59036 | -4.45744 | 2.895028 | H | 6.279859 | -4.10308 | -2.50493 |
| H | 5.212481 | 2.16754 | 4.607198 | H | -5.9401 | 0.201651 | -3.66337 |
| H | 3.100179 | -1.56302 | 6.854435 | H | -0.17829 | -2.98496 | -5.78577 |
| H | 1.114326 | 1.136421 | 6.839937 | H | -3.51197 | -3.06439 | -5.99433 |
| H | -0.15616 | -1.88147 | 6.182322 | H | -1.86604 | -0.19742 | -6.52622 |
| H | 4.586133 | 1.515415 | -1.10848 | H | -3.8685 | 1.418593 | 1.599908 |
| H | 8.404194 | 4.480658 | 2.221169 | H | -8.86639 | 2.657955 | -1.21708 |
| H | 11.67275 | 6.626844 | 0.323085 | H | -12.2031 | 4.076687 | 4.115207 |
| H | 12.83428 | 5.103661 | -2.43635 | H | -10.9213 | 6.982275 | 3.134464 |
| H | 10.91668 | 7.801772 | -2.73148 | H | -12.0321 | 4.795144 | 0.839709 |
| H | 9.669458 | 2.517544 | -5.20652 | H | -7.99129 | 2.981111 | 6.505135 |
| H | 6.438998 | 2.39304 | -4.35043 | H | -5.08704 | 3.118636 | 4.849145 |
| H | 7.772978 | 5.227077 | -5.53435 | H | -6.76358 | 5.924781 | 5.570784 |
| H | 4.378471 | -5.05128 | 4.564804 | H | -3.85496 | -7.23255 | -0.15807 |
| H | 6.403014 | -2.92398 | 3.015198 | H | -3.55128 | -6.57682 | -3.37964 |
| H | 4.87819 | -8.22452 | 1.362398 | H | -7.59654 | -4.69942 | -3.57204 |
| H | 6.839291 | -10.0913 | -2.05263 | H | -11.4961 | -3.42578 | -2.0879 |
| H | 7.168844 | -8.02275 | -4.67609 | H | -12.3233 | -4.70687 | 0.907016 |
| H | 9.814913 | -8.71722 | -2.78753 | H | -11.2166 | -1.57142 | 0.693858 |
| H | 10.42297 | -3.90547 | -2.09532 | H | -5.64066 | -5.46823 | 3.233335 |
| H | 7.650099 | -3.10076 | -3.74061 | H | -7.08134 | -2.47991 | 3.654088 |
| H | 8.030374 | -2.1219 | -0.56918 | H | -8.83525 | -5.25655 | 4.235407 |
| H | -9.62955 | 1.108978 | -2.10087 | H | 11.38171 | 0.087978 | 3.841535 |
| H | -7.58723 | -0.35702 | -4.32938 | H | 10.98629 | -3.12088 | 4.750949 |
| H | -6.36589 | 0.870404 | -1.47029 | H | 8.543437 | -0.89344 | 5.343365 |
| H | -7.77672 | -1.2413 | 2.328239 | H | 7.148968 | 0.153958 | -1.10409 |
| H | -10.5065 | -4.34614 | -4.16618 | H | 12.33158 | -1.15508 | -0.55793 |
| H | -11.3283 | -5.61785 | -1.16692 | H | 11.91068 | -4.42788 | 0.052204 |
| H | -12.4731 | -2.59493 | -2.07634 | H | 10.17968 | -2.93888 | -2.39774 |
| H | -2.00429 | 2.814159 | -2.64072 | H | 2.907323 | 2.751022 | 1.753125 |
| H | -4.5421 | 5.492351 | -5.42879 | H | 5.52013 | 6.031087 | 3.692646 |
| H | -6.30483 | 9.555585 | -3.86819 | H | 5.982486 | 10.22976 | 1.637569 |
| H | -5.48217 | 10.92093 | 0.552711 | H | 3.791325 | 11.09973 | -2.42857 |
| H | -2.90435 | 8.195907 | 3.393825 | H | 1.15481 | 7.747605 | -4.41894 |
| **2B-5** | | | | **2B-6** | | | |
| Atom | X | Y | Z | Atom | X | Y | Z |
| C | -0.07709 | 1.380301 | -1.5655 | C | 0.031094 | 0.300587 | -1.25463 |
| C | 1.997412 | -0.55126 | -1.93974 | C | 2.40917 | -1.22931 | -1.64458 |
| C | 1.643963 | -3.02393 | -0.47255 | C | 2.800474 | -3.36846 | 0.265089 |
| C | -1.03496 | -4.05772 | -1.11938 | C | 0.350208 | -4.99862 | 0.272574 |
| C | -3.19106 | -2.12948 | -0.98258 | C | -2.16249 | -3.55073 | 0.421384 |
| C | -2.64794 | 0.289909 | -2.5905 | C | -2.3592 | -1.42384 | -1.6362 |
| C | 1.858839 | -2.5453 | 2.386766 | C | 3.2718 | -2.28865 | 2.923945 |
| O | 3.711802 | -3.95884 | 3.383318 | O | 5.503565 | -3.08777 | 3.819851 |
| C | 5.353006 | -5.00168 | 1.433937 | C | 7.07086 | -4.15804 | 1.825069 |
| C | 3.717547 | -4.9877 | -0.97134 | C | 5.174367 | -4.95783 | -0.23031 |
| C | 0.7159 | 3.956404 | -2.6121 | C | 0.120292 | 2.72408 | -2.83428 |
| C | 2.7674 | 5.343414 | -1.25657 | C | 1.914136 | 4.746399 | -2.01759 |
| O | -0.29163 | 4.879288 | -4.4772 | O | -1.21298 | 3.024344 | -4.69817 |
| C | -4.70481 | 2.245048 | -2.15506 | C | -4.7015 | 0.172757 | -1.19848 |
| C | -2.47606 | -0.33324 | -5.42691 | C | -2.43375 | -2.54933 | -4.31763 |
| C | -5.32186 | 3.230802 | 0.103925 | C | -5.05863 | 1.78663 | 0.729621 |
| C | -7.30458 | 5.095129 | 0.455435 | C | -7.39097 | 3.185086 | 1.094509 |
| C | -8.02747 | 6.185506 | 2.646335 | C | -7.94512 | 4.814474 | 2.978503 |
| C | -10.1319 | 8.101086 | 2.688198 | C | -10.4863 | 6.091275 | 3.093072 |
| C | -6.86568 | 5.638686 | 5.185802 | C | -6.16572 | 5.509119 | 5.08798 |
| C | -5.71228 | -3.4248 | -1.68859 | C | -4.3276 | -5.51416 | 0.345434 |
| C | -6.54469 | -5.27091 | 0.305007 | C | -6.90518 | -4.44946 | 0.868051 |
| C | -6.52258 | -7.8045 | 0.204243 | C | -8.99627 | -4.60928 | -0.55422 |
| C | -7.38922 | -9.36309 | 2.429315 | C | -11.4111 | -3.3433 | 0.278536 |
| C | -5.65432 | -9.3316 | -2.03942 | C | -9.162 | -5.92171 | -3.07646 |
| C | 7.78481 | -3.38587 | 1.342424 | C | 9.038997 | -2.14293 | 1.049563 |
| C | 9.00898 | -3.28283 | 3.957829 | C | 10.54619 | -1.2874 | 3.361796 |
| O | 7.134914 | -0.82692 | 0.737749 | O | 7.751358 | 0.066105 | 0.156411 |
| C | 9.635037 | -4.46988 | -0.60222 | C | 10.80923 | -3.19969 | -0.98224 |
| O | 3.756827 | -0.21625 | -3.39836 | O | 3.85335 | -0.85865 | -3.40898 |
| O | 0.54454 | -1.18391 | 3.68278 | O | 1.863758 | -0.97794 | 4.172564 |
| C | 3.621704 | 7.626428 | -2.30827 | C | 2.094246 | 6.920848 | -3.53051 |
| C | 5.542708 | 8.995266 | -1.15126 | C | 3.746051 | 8.861487 | -2.89285 |
| C | 6.641853 | 8.097376 | 1.079032 | C | 5.25142 | 8.653483 | -0.72977 |
| C | 5.805116 | 5.833001 | 2.141436 | C | 5.087785 | 6.501983 | 0.786674 |
| C | 3.874483 | 4.462891 | 0.986995 | C | 3.424575 | 4.559418 | 0.154852 |
| H | -0.33444 | 1.570117 | 0.478057 | H | 0.018754 | 0.828486 | 0.743889 |
| H | -1.44743 | -5.63816 | 0.148361 | H | 0.471867 | -6.32561 | 1.856344 |
| H | -0.91876 | -4.85578 | -3.02696 | H | 0.398059 | -6.14783 | -1.45 |
| H | -3.34931 | -1.50736 | 0.984823 | H | -2.2431 | -2.5972 | 2.257121 |
| H | 5.816362 | -6.91556 | 2.046214 | H | 8.033128 | -5.7772 | 2.664752 |
| H | 2.864751 | -6.84746 | -1.24428 | H | 4.730083 | -6.96307 | -0.02356 |
| H | 4.787825 | -4.53087 | -2.67104 | H | 5.885786 | -4.66468 | -2.14105 |
| H | -5.72864 | 2.87587 | -3.82462 | H | -6.21238 | -0.06446 | -2.57115 |
| H | -1.00816 | -1.73132 | -5.83464 | H | -2.44216 | -1.03603 | -5.71427 |
| H | -4.27597 | -1.09247 | -6.10355 | H | -0.80804 | -3.76903 | -4.70116 |
| H | -2.04476 | 1.373332 | -6.49716 | H | -4.14567 | -3.67783 | -4.57111 |
| H | -4.28772 | 2.619924 | 1.773984 | H | -3.56769 | 2.044073 | 2.122367 |
| H | -8.29612 | 5.653441 | -1.26305 | H | -8.84484 | 2.838794 | -0.32505 |
| H | -9.46699 | 9.916447 | 3.447074 | H | -11.4625 | 5.644962 | 4.871446 |
| H | -10.9134 | 8.43404 | 0.803015 | H | -10.2836 | 8.15795 | 3.044177 |
| H | -11.6747 | 7.480468 | 3.932422 | H | -11.7083 | 5.523125 | 1.524452 |
| H | -6.10187 | 7.379307 | 6.020242 | H | -5.83533 | 7.558699 | 5.110137 |
| H | -8.30918 | 4.943816 | 6.506044 | H | -7.00111 | 5.033664 | 6.928312 |
| H | -5.34452 | 4.246755 | 5.116915 | H | -4.33143 | 4.57281 | 4.961663 |
| H | -5.53445 | -4.3393 | -3.53414 | H | -3.88722 | -6.92495 | 1.808088 |
| H | -7.15493 | -1.94927 | -1.88754 | H | -4.27465 | -6.53366 | -1.45175 |
| H | -7.18298 | -4.39571 | 2.060242 | H | -7.04547 | -3.40573 | 2.638043 |
| H | -5.87344 | -10.6187 | 3.090687 | H | -12.9697 | -4.70721 | 0.428896 |
| H | -7.98477 | -8.17769 | 4.015961 | H | -11.1934 | -2.39717 | 2.104597 |
| H | -8.97868 | -10.5918 | 1.904058 | H | -12.0089 | -1.92019 | -1.1123 |
| H | -5.08883 | -8.17144 | -3.64936 | H | -7.42281 | -6.89617 | -3.61061 |
| H | -4.0369 | -10.5239 | -1.51737 | H | -10.6967 | -7.31924 | -3.07455 |
| H | -7.15343 | -10.621 | -2.67031 | H | -9.61563 | -4.55696 | -4.57521 |
| H | 10.72571 | -2.13444 | 3.87028 | H | 11.93047 | 0.138515 | 2.79248 |
| H | 9.516992 | -5.18028 | 4.606529 | H | 11.5528 | -2.88114 | 4.213511 |
| H | 7.716222 | -2.43845 | 5.328136 | H | 9.29108 | -0.46351 | 4.778853 |
| H | 6.68608 | -0.73043 | -1.045 | H | 7.075125 | -0.27758 | -1.5209 |
| H | 8.850884 | -4.43111 | -2.51678 | H | 9.788609 | -3.68333 | -2.71618 |
| H | 11.36814 | -3.34289 | -0.59895 | H | 12.23346 | -1.77786 | -1.4552 |
| H | 10.12788 | -6.42859 | -0.15148 | H | 11.77976 | -4.90087 | -0.31394 |
| H | 2.752498 | 8.28797 | -4.04226 | H | 0.920196 | 7.043814 | -5.20557 |
| H | 6.191219 | 10.75601 | -1.98208 | H | 3.869964 | 10.53306 | -4.07667 |
| H | 8.1467 | 9.160954 | 1.982737 | H | 6.547671 | 10.16475 | -0.23155 |
| H | 6.654729 | 5.125232 | 3.86922 | H | 6.255627 | 6.328829 | 2.464263 |
| H | 3.259213 | 2.718445 | 1.861116 | H | 3.335323 | 2.918863 | 1.373529 |
| **2B-7** | | | | **2B-8** | | | |
| Atom | X | Y | Z | Atom | X | Y | Z |
| C | 0.538806 | -1.50801 | -1.56652 | C | 0.03129 | 1.513072 | 1.708191 |
| C | -1.94072 | -0.15033 | -2.03548 | C | -2.38391 | 0.048832 | 2.183654 |
| C | -2.25557 | 2.349098 | -0.58854 | C | -2.40643 | -2.64945 | 1.101416 |
| C | 0.094541 | 4.006174 | -1.23373 | C | -0.01522 | -4.0158 | 2.152014 |
| C | 2.663038 | 2.679855 | -1.02651 | C | 2.465261 | -2.53158 | 1.948453 |
| C | 2.775178 | 0.165551 | -2.58136 | C | 2.279395 | 0.176733 | 3.121387 |
| C | -2.36305 | 1.854042 | 2.267881 | C | -2.30872 | -2.56367 | -1.79635 |
| O | -4.51227 | 2.765187 | 3.259602 | O | -4.31307 | -3.75301 | -2.79732 |
| C | -6.22935 | 3.740528 | 1.349531 | C | -6.12844 | -4.57944 | -0.88793 |
| C | -4.75564 | 3.701788 | -1.15871 | C | -4.8567 | -4.08955 | 1.682183 |
| C | 0.399193 | -4.21236 | -2.56501 | C | -0.36747 | 4.313702 | 2.280531 |
| C | -1.3563 | -5.98703 | -1.24074 | C | -2.11577 | 5.739639 | 0.580095 |
| O | 1.646872 | -4.92691 | -4.37496 | O | 0.674353 | 5.372573 | 4.051541 |
| C | 5.244744 | -1.20464 | -2.06385 | C | 4.686595 | 1.646358 | 2.577176 |
| C | 2.518907 | 0.664339 | -5.43566 | C | 1.823457 | 0.072458 | 5.991348 |
| C | 6.041936 | -1.95865 | 0.227852 | C | 5.666656 | 2.003361 | 0.26064 |
| C | 8.418966 | -3.26284 | 0.652213 | C | 8.004062 | 3.3566 | -0.22174 |
| C | 9.348522 | -4.09545 | 2.878009 | C | 9.1714 | 3.648233 | -2.47232 |
| C | 11.86275 | -5.42398 | 2.994105 | C | 11.61564 | 5.092984 | -2.66316 |
| C | 8.038245 | -3.80689 | 5.387449 | C | 8.217119 | 2.592965 | -4.93699 |
| C | 4.79113 | 4.55044 | -1.73749 | C | 4.661556 | -4.10697 | 3.060788 |
| C | 5.104071 | 6.589668 | 0.216699 | C | 5.217449 | -6.44104 | 1.519661 |
| C | 4.448245 | 9.033505 | 0.05162 | C | 6.640792 | -6.54751 | -0.57658 |
| C | 4.86428 | 10.81001 | 2.242988 | C | 6.981629 | -8.99019 | -2.00434 |
| C | 3.256563 | 10.24184 | -2.23741 | C | 7.991034 | -4.30381 | -1.70167 |
| C | -8.67983 | 2.163604 | 1.41888 | C | -8.6485 | -3.22285 | -1.42542 |
| C | -8.21078 | -0.64584 | 0.902134 | C | -10.6848 | -4.2464 | 0.345024 |
| O | -10.1465 | 3.269326 | -0.57826 | O | -9.43006 | -3.92052 | -3.92886 |
| C | -10.0165 | 2.531301 | 3.960737 | C | -8.42937 | -0.34049 | -1.24225 |
| O | -3.48926 | -0.89191 | -3.56849 | O | -4.10851 | 0.876517 | 3.463173 |
| O | -0.79001 | 0.821798 | 3.581662 | O | -0.6975 | -1.61533 | -3.12573 |
| C | -1.87264 | -8.31585 | -2.40212 | C | -3.01618 | 4.76227 | -1.71661 |
| C | -3.52228 | -10.0307 | -1.28555 | C | -4.67205 | 6.17266 | -3.20265 |
| C | -4.67238 | -9.44467 | 1.020291 | C | -5.44832 | 8.567432 | -2.41279 |
| C | -4.15731 | -7.1425 | 2.20178 | C | -4.5508 | 9.560807 | -0.13445 |
| C | -2.51006 | -5.41939 | 1.080417 | C | -2.89202 | 8.158297 | 1.345532 |
| H | 0.802296 | -1.59186 | 0.480733 | H | 0.466777 | 1.325851 | -0.30242 |
| H | 0.08072 | 5.670919 | -0.00709 | H | 0.180335 | -5.8246 | 1.166878 |
| H | -0.18694 | 4.701053 | -3.16394 | H | -0.41233 | -4.46662 | 4.133859 |
| H | 2.940977 | 2.1583 | 0.956944 | H | 2.87183 | -2.28158 | -0.06266 |
| H | -6.68794 | 5.668222 | 1.924896 | H | -6.41093 | -6.59188 | -1.2412 |
| H | -4.40067 | 5.6293 | -1.80335 | H | -4.43968 | -5.87824 | 2.621485 |
| H | -5.80676 | 2.725327 | -2.63506 | H | -6.05085 | -2.97753 | 2.940888 |
| H | 6.428157 | -1.59555 | -3.70118 | H | 5.66109 | 2.441785 | 4.205083 |
| H | 0.765468 | 1.653442 | -5.90527 | H | 1.640065 | 1.981773 | 6.744287 |
| H | 4.095843 | 1.821891 | -6.10439 | H | 0.107002 | -0.97348 | 6.475062 |
| H | 2.539499 | -1.12044 | -6.46524 | H | 3.413416 | -0.85226 | 6.935157 |
| H | 4.855345 | -1.59011 | 1.86735 | H | 4.679744 | 1.220053 | -1.36437 |
| H | 9.551615 | -3.58998 | -1.03838 | H | 8.899448 | 4.188483 | 1.437568 |
| H | 11.65712 | -7.3322 | 3.787735 | H | 11.42612 | 6.702182 | -3.96237 |
| H | 12.73811 | -5.58896 | 1.12801 | H | 12.2352 | 5.812371 | -0.82627 |
| H | 13.17857 | -4.41306 | 4.243263 | H | 13.12571 | 3.895234 | -3.43675 |
| H | 7.715837 | -5.6667 | 6.251613 | H | 7.946366 | 4.115464 | -6.32163 |
| H | 9.237138 | -2.74879 | 6.711315 | H | 9.620322 | 1.300634 | -5.75606 |
| H | 6.219967 | -2.83993 | 5.264397 | H | 6.437579 | 1.564243 | -4.76352 |
| H | 4.42041 | 5.350886 | -3.60729 | H | 4.199945 | -4.66346 | 5.001901 |
| H | 6.559997 | 3.479039 | -1.8847 | H | 6.330617 | -2.89026 | 3.174456 |
| H | 5.915763 | 5.942087 | 1.998843 | H | 4.311928 | -8.18083 | 2.148118 |
| H | 6.103266 | 12.38532 | 1.699563 | H | 6.318273 | -8.80236 | -3.96317 |
| H | 3.072982 | 11.66081 | 2.8587 | H | 8.987702 | -9.5151 | -2.11474 |
| H | 5.714214 | 9.848353 | 3.864779 | H | 5.951031 | -10.5509 | -1.12101 |
| H | 1.38499 | 11.00213 | -1.75874 | H | 7.832779 | -2.59662 | -0.55569 |
| H | 4.393479 | 11.85087 | -2.89037 | H | 10.00672 | -4.7223 | -1.96688 |
| H | 3.021838 | 8.939484 | -3.82055 | H | 7.232738 | -3.86919 | -3.5856 |
| H | -10.0203 | -1.65266 | 0.87366 | H | -10.2944 | -3.74536 | 2.311394 |
| H | -7.05371 | -1.50022 | 2.387983 | H | -10.8146 | -6.30614 | 0.196174 |
| H | -7.28319 | -0.94021 | -0.9181 | H | -12.5139 | -3.43844 | -0.17974 |
| H | -11.7441 | 2.369724 | -0.64198 | H | -8.09888 | -3.36584 | -5.06695 |
| H | -11.801 | 1.480321 | 3.987772 | H | -7.83357 | 0.281016 | 0.635303 |
| H | -8.8455 | 1.846994 | 5.521494 | H | -7.07301 | 0.389384 | -2.62511 |
| H | -10.4334 | 4.534061 | 4.270908 | H | -10.2694 | 0.503899 | -1.65876 |
| H | -0.96841 | -8.73652 | -4.19261 | H | -2.41801 | 2.921594 | -2.38727 |
| H | -3.91847 | -11.8205 | -2.20766 | H | -5.3506 | 5.401257 | -4.97856 |
| H | -5.96224 | -10.7807 | 1.893798 | H | -6.74493 | 9.65929 | -3.56965 |
| H | -5.03322 | -6.68688 | 4.000272 | H | -5.15045 | 11.42356 | 0.483006 |
| H | -2.11908 | -3.659 | 2.051976 | H | -2.18101 | 8.897001 | 3.120142 |
| **2B-9** | | | | **2B-10** | | | |
| Atom | X | Y | Z | Atom | X | Y | Z |
| C | 0.715979 | 0.410734 | 1.452646 | C | -0.32203 | 0.658874 | 0.409369 |
| C | -1.86679 | 1.570419 | 1.817852 | C | -2.64128 | -0.52239 | 1.588275 |
| C | -3.96936 | 0.315431 | 0.260117 | C | -3.37959 | -3.11028 | 0.527835 |
| C | -3.84454 | -2.59611 | 0.602254 | C | -0.99398 | -4.83329 | 0.628047 |
| C | -1.20278 | -3.78533 | 0.510612 | C | 1.468584 | -3.6528 | -0.35282 |
| C | 0.685937 | -2.43246 | 2.33716 | C | 2.06357 | -1.05898 | 0.956177 |
| C | -3.57372 | 1.067714 | -2.52786 | C | -4.28629 | -2.85709 | -2.22531 |
| O | -5.4004 | 2.657892 | -3.28488 | O | -6.64794 | -3.74607 | -2.46326 |
| C | -7.0515 | 3.412326 | -1.20161 | C | -7.86688 | -4.04761 | -0.0135 |
| C | -6.60311 | 1.378533 | 0.821301 | C | -5.65814 | -4.31038 | 1.861004 |
| C | 2.803526 | 2.012675 | 2.638073 | C | -0.14458 | 3.434725 | 1.208665 |
| C | 4.513052 | 3.454312 | 0.915926 | C | -1.88999 | 5.265399 | -0.05166 |
| O | 3.093901 | 2.099319 | 4.930309 | O | 1.335103 | 4.157278 | 2.829398 |
| C | 3.312697 | -3.5473 | 2.055858 | C | 4.270154 | 0.159141 | -0.37549 |
| C | -0.13644 | -2.66569 | 5.118962 | C | 2.495758 | -1.36158 | 3.810761 |
| C | 5.036549 | -2.90108 | 0.304473 | C | 6.335052 | 1.176914 | 0.687715 |
| C | 7.5559 | -3.97865 | 0.15982 | C | 8.404015 | 2.212187 | -0.78988 |
| C | 9.454226 | -3.20144 | -1.35853 | C | 10.62871 | 3.112475 | 0.07695 |
| C | 11.99401 | -4.48191 | -1.27371 | C | 12.6102 | 4.069924 | -1.72765 |
| C | 9.273823 | -1.03262 | -3.19192 | C | 11.36154 | 3.225687 | 2.824139 |
| C | -1.3977 | -6.66208 | 0.978037 | C | 3.611021 | -5.63869 | -0.13953 |
| C | -2.73185 | -7.99415 | -1.14936 | C | 5.803765 | -5.17907 | -1.88671 |
| C | -5.06743 | -8.98108 | -1.15938 | C | 8.23279 | -4.77155 | -1.29811 |
| C | -6.12104 | -10.2169 | -3.50319 | C | 10.16664 | -4.28396 | -3.33466 |
| C | -6.84741 | -8.95981 | 1.065422 | C | 9.255552 | -4.61797 | 1.351239 |
| C | -6.55035 | 6.253213 | -0.43356 | C | -9.64354 | -1.76338 | 0.391606 |
| C | -4.05121 | 7.274675 | -1.49662 | C | -11.5167 | -1.56488 | -1.79985 |
| O | -6.58305 | 6.517754 | 2.253015 | O | -8.19064 | 0.521498 | 0.324776 |
| C | -8.75582 | 7.856472 | -1.39263 | C | -11.0507 | -2.01976 | 2.908183 |
| O | -2.26034 | 3.369499 | 3.210471 | O | -3.77958 | 0.470869 | 3.336973 |
| O | -1.89541 | 0.387587 | -3.93258 | O | -3.09692 | -2.08011 | -4.02484 |
| C | 3.909156 | 3.998424 | -1.61132 | C | -1.70026 | 7.822589 | 0.636637 |
| C | 5.597015 | 5.352513 | -3.11503 | C | -3.29301 | 9.618321 | -0.43136 |
| C | 7.913126 | 6.141309 | -2.12861 | C | -5.11553 | 8.879353 | -2.19766 |
| C | 8.531327 | 5.601256 | 0.383289 | C | -5.32657 | 6.342947 | -2.889 |
| C | 6.83389 | 4.28534 | 1.897742 | C | -3.7191 | 4.542849 | -1.83214 |
| H | 0.985216 | 0.322877 | -0.59029 | H | -0.58971 | 0.578653 | -1.64035 |
| H | -5.03556 | -3.47177 | -0.84245 | H | -1.40666 | -6.54972 | -0.45206 |
| H | -4.73525 | -3.02078 | 2.423092 | H | -0.76346 | -5.43082 | 2.597706 |
| H | -0.44458 | -3.52213 | -1.39881 | H | 1.199946 | -3.25117 | -2.36777 |
| H | -8.95967 | 3.262104 | -1.96409 | H | -8.98287 | -5.77686 | -0.14191 |
| H | -7.99296 | -0.13075 | 0.581646 | H | -5.28483 | -6.3093 | 2.216717 |
| H | -6.78861 | 2.115887 | 2.731115 | H | -6.02723 | -3.40618 | 3.674401 |
| H | 3.809894 | -4.99262 | 3.435821 | H | 4.116224 | 0.174953 | -2.43147 |
| H | -2.02606 | -1.89044 | 5.44487 | H | 2.728277 | 0.481918 | 4.696181 |
| H | -0.15937 | -4.65193 | 5.692509 | H | 0.899109 | -2.30114 | 4.728594 |
| H | 1.176441 | -1.64411 | 6.335093 | H | 4.188164 | -2.48546 | 4.179677 |
| H | 4.571701 | -1.45474 | -1.07388 | H | 6.512575 | 1.181085 | 2.730637 |
| H | 7.932906 | -5.53784 | 1.453152 | H | 8.109858 | 2.207348 | -2.82984 |
| H | 12.49538 | -5.25545 | -3.1346 | H | 11.98767 | 3.944333 | -3.69571 |
| H | 13.48911 | -3.12601 | -0.78492 | H | 13.0948 | 6.048677 | -1.32294 |
| H | 12.03958 | -6.02329 | 0.103723 | H | 14.37275 | 2.987691 | -1.53455 |
| H | 10.62366 | 0.456259 | -2.67307 | H | 11.83954 | 5.170604 | 3.370149 |
| H | 9.788613 | -1.65377 | -5.10405 | H | 9.884456 | 2.551435 | 4.097013 |
| H | 7.4062 | -0.16424 | -3.27697 | H | 13.06506 | 2.088098 | 3.164596 |
| H | -2.33235 | -7.01256 | 2.788642 | H | 2.772631 | -7.47542 | -0.63281 |
| H | 0.524654 | -7.4214 | 1.141548 | H | 4.218504 | -5.81089 | 1.826985 |
| H | -1.6491 | -8.09653 | -2.90161 | H | 5.298159 | -5.17613 | -3.88686 |
| H | -4.77386 | -10.1705 | -5.07166 | H | 9.338212 | -4.38956 | -5.22721 |
| H | -6.62469 | -12.1979 | -3.1378 | H | 11.00121 | -2.39815 | -3.09717 |
| H | -7.86574 | -9.27375 | -4.11797 | H | 11.73172 | -5.64497 | -3.23236 |
| H | -6.02771 | -8.11735 | 2.761228 | H | 9.904794 | -2.68483 | 1.737187 |
| H | -8.56948 | -7.89823 | 0.599109 | H | 7.869022 | -5.11739 | 2.795483 |
| H | -7.45582 | -10.888 | 1.533777 | H | 10.89984 | -5.86268 | 1.581506 |
| H | -3.84426 | 9.240517 | -0.89061 | H | -12.761 | 0.058018 | -1.49727 |
| H | -4.01357 | 7.20816 | -3.55985 | H | -12.6783 | -3.27163 | -1.92978 |
| H | -2.41682 | 6.227048 | -0.78769 | H | -10.5152 | -1.30766 | -3.58676 |
| H | -5.08206 | 5.654586 | 2.896049 | H | -7.23844 | 0.663179 | 1.894107 |
| H | -8.46643 | 9.841089 | -0.89045 | H | -12.1483 | -3.77291 | 2.973475 |
| H | -8.92392 | 7.709315 | -3.44962 | H | -9.75245 | -2.01566 | 4.519522 |
| H | -10.5298 | 7.21542 | -0.54012 | H | -12.3497 | -0.42698 | 3.130526 |
| H | 2.128272 | 3.398438 | -2.42995 | H | -0.2863 | 8.357754 | 2.020051 |
| H | 5.105382 | 5.780022 | -5.06023 | H | -3.1247 | 11.59032 | 0.110806 |
| H | 9.234687 | 7.171964 | -3.31278 | H | -6.36694 | 10.27772 | -3.02888 |
| H | 10.33409 | 6.208017 | 1.152761 | H | -6.74371 | 5.758445 | -4.25238 |
| H | 7.279306 | 3.85516 | 3.851354 | H | -3.92832 | 2.591147 | -2.40935 |
| **2B-11** | | | | **2B-12** | | | |
| Atom | X | Y | Z | Atom | X | Y | Z |
| C | -0.12671 | -1.24256 | 0.609777 | C | -0.11709 | 0.775871 | 0.67919 |
| C | 2.238197 | -0.26193 | 1.883443 | C | -2.54188 | -0.1716 | 1.856244 |
| C | 2.891738 | 2.499682 | 1.310512 | C | -3.30953 | -2.85395 | 1.095041 |
| C | 0.500452 | 4.110959 | 1.904296 | C | -0.99942 | -4.60349 | 1.606451 |
| C | -1.99535 | 3.094551 | 0.845135 | C | 1.563966 | -3.64803 | 0.641218 |
| C | -2.4972 | 0.274167 | 1.590685 | C | 2.181991 | -0.9086 | 1.583058 |
| C | 3.626681 | 2.813853 | -1.48521 | C | -4.01455 | -2.9577 | -1.72492 |
| O | 5.964741 | 3.772991 | -1.68226 | O | -6.38841 | -3.79899 | -2.01156 |
| C | 7.327411 | 3.623537 | 0.70503 | C | -7.78086 | -3.72755 | 0.362255 |
| C | 5.236206 | 3.460491 | 2.72024 | C | -5.71592 | -3.79675 | 2.409588 |
| C | -0.24492 | -4.12474 | 0.835618 | C | 0.108421 | 3.629565 | 1.104896 |
| C | 1.544736 | -5.63076 | -0.7494 | C | -1.499 | 5.314503 | -0.49602 |
| O | -1.72912 | -5.18344 | 2.255454 | O | 1.516547 | 4.529919 | 2.700135 |
| C | -4.73923 | -0.63513 | 0.078066 | C | 4.468889 | 0.054537 | 0.175788 |
| C | -2.85095 | -0.02543 | 4.454757 | C | 2.499405 | -0.81467 | 4.4656 |
| C | -6.92666 | -1.56662 | 0.967305 | C | 6.51269 | 1.197975 | 1.152245 |
| C | -8.99656 | -2.34324 | -0.6616 | C | 8.642375 | 1.990484 | -0.38701 |
| C | -11.2516 | -3.29785 | 0.055033 | C | 10.81324 | 3.07178 | 0.407026 |
| C | -13.206 | -4.01509 | -1.88569 | C | 12.86054 | 3.751541 | -1.44971 |
| C | -12.0378 | -3.73676 | 2.754331 | C | 11.40999 | 3.691686 | 3.119382 |
| C | -4.18364 | 4.877213 | 1.606923 | C | 3.587677 | -5.65629 | 1.313978 |
| C | -4.0428 | 7.402186 | 0.28934 | C | 6.165812 | -5.22138 | 0.181255 |
| C | -4.8495 | 7.899017 | -2.06252 | C | 6.860664 | -5.72114 | -2.20411 |
| C | -4.54315 | 10.48649 | -3.2158 | C | 9.481695 | -5.09518 | -3.1291 |
| C | -6.09316 | 5.971032 | -3.75373 | C | 5.122012 | -6.83337 | -4.16744 |
| C | 9.155188 | 1.349196 | 0.56017 | C | -9.48847 | -1.35688 | 0.331802 |
| C | 10.87694 | 1.604223 | -1.74636 | C | -11.1897 | -1.39017 | -2.00382 |
| O | 7.731043 | -0.9197 | 0.161383 | O | -7.94372 | 0.853988 | 0.080619 |
| C | 10.72672 | 1.172766 | 2.984938 | C | -11.0805 | -1.23666 | 2.746324 |
| O | 3.472625 | -1.54818 | 3.352452 | O | -3.74189 | 1.074419 | 3.38806 |
| O | 2.334753 | 2.384298 | -3.33019 | O | -2.67505 | -2.47486 | -3.52198 |
| C | 3.279692 | -4.53099 | -2.42768 | C | -1.27266 | 7.935412 | -0.13722 |
| C | 4.931034 | -6.04626 | -3.81215 | C | -2.73917 | 9.609571 | -1.53287 |
| C | 4.859431 | -8.6696 | -3.54997 | C | -4.47028 | 8.68459 | -3.3036 |
| C | 3.130401 | -9.78329 | -1.88919 | C | -4.71654 | 6.084455 | -3.6701 |
| C | 1.491011 | -8.27327 | -0.49836 | C | -3.235 | 4.404041 | -2.28348 |
| H | 0.03911 | -0.75087 | -1.39417 | H | -0.2719 | 0.423728 | -1.35362 |
| H | 0.821683 | 6.026042 | 1.18977 | H | -1.40197 | -6.45172 | 0.765471 |
| H | 0.381798 | 4.267797 | 3.966477 | H | -0.91861 | -4.90521 | 3.65415 |
| H | -1.86434 | 3.14349 | -1.22002 | H | 1.462871 | -3.55336 | -1.42423 |
| H | 8.410965 | 5.372057 | 0.851314 | H | -8.95324 | -5.42394 | 0.385515 |
| H | 4.865364 | 5.339077 | 3.491093 | H | -5.44367 | -5.73808 | 3.056311 |
| H | 5.725521 | 2.221226 | 4.291039 | H | -6.17197 | -2.64346 | 4.054048 |
| H | -4.50623 | -0.46102 | -1.96558 | H | 4.388071 | -0.23296 | -1.86371 |
| H | -1.18882 | 0.633177 | 5.493812 | H | 4.134186 | -1.934 | 5.051956 |
| H | -4.47286 | 1.074583 | 5.108625 | H | 2.768292 | 1.125328 | 5.097693 |
| H | -3.15293 | -2.00358 | 4.938518 | H | 0.8362 | -1.56717 | 5.437133 |
| H | -7.17439 | -1.76274 | 2.994976 | H | 6.61701 | 1.506435 | 3.177705 |
| H | -8.65581 | -2.11465 | -2.68157 | H | 8.441206 | 1.632417 | -2.40708 |
| H | -13.7051 | -6.02491 | -1.72747 | H | 12.33946 | 3.259086 | -3.3894 |
| H | -14.9669 | -2.9535 | -1.59243 | H | 13.27868 | 5.785132 | -1.38469 |
| H | -12.5488 | -3.66036 | -3.81389 | H | 14.63597 | 2.777948 | -0.98662 |
| H | -13.7599 | -2.66209 | 3.189434 | H | 11.78805 | 5.722013 | 3.332001 |
| H | -12.5073 | -5.73656 | 3.053941 | H | 9.899117 | 3.186025 | 4.430185 |
| H | -10.594 | -3.20955 | 4.130329 | H | 13.13734 | 2.711045 | 3.723864 |
| H | -4.14824 | 5.173055 | 3.656796 | H | 2.839867 | -7.48505 | 0.685423 |
| H | -5.97184 | 3.928183 | 1.183641 | H | 3.750279 | -5.79629 | 3.373494 |
| H | -3.14593 | 8.930891 | 1.337956 | H | 7.562448 | -4.36363 | 1.420173 |
| H | -3.41496 | 10.3944 | -4.95673 | H | 10.63893 | -4.24879 | -1.63855 |
| H | -6.38305 | 11.28523 | -3.75327 | H | 10.45765 | -6.7854 | -3.83896 |
| H | -3.61919 | 11.80448 | -1.91686 | H | 9.402308 | -3.75491 | -4.71436 |
| H | -7.92873 | 6.665998 | -4.42889 | H | 3.310078 | -7.44746 | -3.38945 |
| H | -4.93573 | 5.636501 | -5.4447 | H | 4.726982 | -5.44489 | -5.66088 |
| H | -6.39592 | 4.151367 | -2.82751 | H | 6.008515 | -8.46167 | -5.10029 |
| H | 9.759245 | 1.636304 | -3.48174 | H | -12.4001 | -3.06772 | -2.00841 |
| H | 12.1721 | -0.00532 | -1.82129 | H | -10.0519 | -1.39043 | -3.72639 |
| H | 11.99154 | 3.34364 | -1.64272 | H | -12.3927 | 0.291005 | -2.00384 |
| H | 6.876179 | -1.37011 | 1.72833 | H | -7.10058 | 1.174465 | 1.685473 |
| H | 11.81703 | 2.904367 | 3.293893 | H | -12.3217 | 0.414521 | 2.665262 |
| H | 9.541125 | 0.857046 | 4.651293 | H | -12.2548 | -2.9283 | 2.950184 |
| H | 12.04613 | -0.41117 | 2.830192 | H | -9.90169 | -1.06795 | 4.438655 |
| H | 3.378031 | -2.5027 | -2.67681 | H | 0.068788 | 8.615998 | 1.254429 |
| H | 6.274163 | -5.17187 | -5.09269 | H | -2.54394 | 11.63167 | -1.24298 |
| H | 6.146914 | -9.84514 | -4.63299 | H | -5.62354 | 9.988601 | -4.39078 |
| H | 3.071217 | -11.8238 | -1.68034 | H | -6.06363 | 5.356984 | -5.03567 |
| H | 0.146801 | -9.10188 | 0.807818 | H | -3.4692 | 2.398444 | -2.6131 |
| **2B-13** | | | | **2B-14** | | | |
| Atom | X | Y | Z | Atom | X | Y | Z |
| C | 0.31018 | -1.39176 | -0.5818 | C | 0.310609 | -1.39162 | -0.58174 |
| C | -2.10572 | -0.55768 | -1.88 | C | -2.10534 | -0.5578 | -1.88005 |
| C | -2.94165 | 2.15983 | -1.2926 | C | -2.94167 | 2.159539 | -1.29261 |
| C | -0.64999 | 3.90165 | -1.92787 | C | -0.65019 | 3.901629 | -1.92797 |
| C | 1.909141 | 3.048329 | -0.86972 | C | 1.909049 | 3.048661 | -0.86982 |
| C | 2.583394 | 0.251638 | -1.57979 | C | 2.583662 | 0.251997 | -1.5797 |
| C | -3.63402 | 2.428765 | 1.51244 | C | -3.634 | 2.428617 | 1.512431 |
| O | -6.01568 | 3.259846 | 1.775921 | O | -6.01563 | 3.259772 | 1.775872 |
| C | -7.35027 | 3.423449 | -0.61955 | C | -7.35038 | 3.4228 | -0.61957 |
| C | -5.35943 | 2.946975 | -2.68711 | C | -5.35952 | 2.946444 | -2.68712 |
| C | 0.565776 | -4.26158 | -0.79037 | C | 0.566427 | -4.26142 | -0.79022 |
| C | -1.23216 | -5.82654 | 0.732286 | C | -1.23164 | -5.82641 | 0.732273 |
| O | 2.127735 | -5.27107 | -2.1611 | O | 2.128554 | -5.27088 | -2.16077 |
| C | 4.872908 | -0.49864 | -0.04977 | C | 4.873199 | -0.49795 | -0.04955 |
| C | 2.965133 | -0.06266 | -4.43825 | C | 2.96556 | -0.06244 | -4.43813 |
| C | 7.119984 | -1.29816 | -0.91882 | C | 7.120089 | -1.29816 | -0.91844 |
| C | 9.225511 | -1.92536 | 0.729071 | C | 9.225617 | -1.92512 | 0.72954 |
| C | 11.54108 | -2.74201 | 0.035686 | C | 11.54097 | -2.74252 | 0.036313 |
| C | 13.52477 | -3.31336 | 1.995143 | C | 13.5247 | -3.31341 | 1.995863 |
| C | 12.371 | -3.15737 | -2.65426 | C | 12.37066 | -3.15901 | -2.65353 |
| C | 3.981634 | 4.950731 | -1.6651 | C | 3.981309 | 4.95126 | -1.66532 |
| C | 3.697559 | 7.478083 | -0.37543 | C | 3.696735 | 7.478814 | -0.37617 |
| C | 4.484233 | 8.049347 | 1.966292 | C | 4.483041 | 8.050643 | 1.965538 |
| C | 4.030809 | 10.62764 | 3.091373 | C | 4.029085 | 10.62907 | 3.090088 |
| C | 5.847739 | 6.218446 | 3.672966 | C | 5.846609 | 6.22024 | 3.6727 |
| C | -9.59801 | 1.568548 | -0.54824 | C | -9.59782 | 1.567515 | -0.54799 |
| C | -8.76582 | -1.17352 | -0.13691 | C | -8.76528 | -1.17445 | -0.13666 |
| O | -10.6933 | 1.872363 | -3.01165 | O | -10.6933 | 1.871067 | -3.01132 |
| C | -11.4832 | 2.387852 | 1.490671 | C | -11.483 | 2.386592 | 1.491054 |
| O | -3.21208 | -1.88664 | -3.39923 | O | -3.2114 | -1.88685 | -3.39941 |
| O | -2.30834 | 2.017107 | 3.339025 | O | -2.3083 | 2.017088 | 3.339038 |
| C | -1.29069 | -8.43913 | 0.27231 | C | -2.86933 | -4.80253 | 2.552185 |
| C | -2.95661 | -9.9926 | 1.583532 | C | -4.53763 | -6.36134 | 3.866756 |
| C | -4.5894 | -8.95444 | 3.383402 | C | -4.58932 | -8.95424 | 3.382895 |
| C | -4.53797 | -6.36149 | 3.86695 | C | -2.95657 | -9.99237 | 1.582976 |
| C | -2.8699 | -4.80264 | 2.552136 | C | -1.29043 | -8.43893 | 0.271992 |
| H | 0.125875 | -0.89652 | 1.416926 | H | 0.126185 | -0.89637 | 1.41697 |
| H | -1.07683 | 5.808113 | -1.24563 | H | -1.07724 | 5.808071 | -1.2458 |
| H | -0.55358 | 4.025988 | -3.99297 | H | -0.55383 | 4.025905 | -3.99307 |
| H | 1.786205 | 3.116245 | 1.195371 | H | 1.786097 | 3.116688 | 1.195278 |
| H | -8.10222 | 5.341901 | -0.725 | H | -8.10271 | 5.341088 | -0.7252 |
| H | -5.03486 | 4.67252 | -3.77071 | H | -5.03509 | 4.671985 | -3.77077 |
| H | -5.97097 | 1.482311 | -3.99814 | H | -5.97095 | 1.481708 | -3.99812 |
| H | 4.619634 | -0.31946 | 1.991083 | H | 4.620046 | -0.31803 | 1.991252 |
| H | 4.526482 | 1.117144 | -5.1005 | H | 1.274065 | 0.48938 | -5.49123 |
| H | 3.379586 | -2.02714 | -4.89566 | H | 4.527081 | 1.117174 | -5.10032 |
| H | 1.273513 | 0.489031 | -5.49122 | H | 3.379862 | -2.02698 | -4.89545 |
| H | 7.391065 | -1.49868 | -2.94316 | H | 7.391029 | -1.49939 | -2.94272 |
| H | 8.858441 | -1.69813 | 2.74465 | H | 8.85876 | -1.697 | 2.745054 |
| H | 15.216 | -2.14415 | 1.701343 | H | 12.83386 | -2.9813 | 3.916335 |
| H | 12.83369 | -2.98228 | 3.915709 | H | 14.15194 | -5.28884 | 1.862363 |
| H | 14.15247 | -5.28858 | 1.860796 | H | 15.21618 | -2.14473 | 1.701396 |
| H | 10.90651 | -2.73334 | -4.04406 | H | 14.02587 | -1.98429 | -3.08953 |
| H | 14.02629 | -1.98251 | -3.08959 | H | 12.96468 | -5.12885 | -2.92983 |
| H | 12.96499 | -5.12711 | -2.93135 | H | 10.90603 | -2.73563 | -4.04338 |
| H | 3.917189 | 5.219758 | -3.71794 | H | 3.917022 | 5.219896 | -3.71822 |
| H | 5.825897 | 4.115576 | -1.24213 | H | 5.825665 | 4.116457 | -1.24202 |
| H | 2.707276 | 8.938973 | -1.4366 | H | 2.706398 | 8.939376 | -1.43774 |
| H | 5.822705 | 11.53922 | 3.611244 | H | 2.914918 | 10.49137 | 4.837041 |
| H | 3.025979 | 11.87397 | 1.781774 | H | 5.82078 | 11.54102 | 3.61001 |
| H | 2.91683 | 10.48978 | 4.838432 | H | 3.024223 | 11.87501 | 1.780139 |
| H | 7.64387 | 7.027334 | 4.32707 | H | 6.249959 | 4.409629 | 2.767353 |
| H | 4.721906 | 5.839252 | 5.375823 | H | 7.642202 | 7.029774 | 4.327471 |
| H | 6.250092 | 4.407671 | 2.767508 | H | 4.720283 | 5.840584 | 5.375129 |
| H | -7.45825 | -1.7975 | -1.60684 | H | -7.4577 | -1.79833 | -1.60664 |
| H | -10.4243 | -2.41273 | -0.1823 | H | -10.4236 | -2.41384 | -0.18202 |
| H | -7.86293 | -1.41755 | 1.707991 | H | -7.8623 | -1.41838 | 1.708218 |
| H | -12.1526 | 0.763551 | -3.09756 | H | -12.1533 | 0.763172 | -3.09656 |
| H | -10.6223 | 2.318539 | 3.369236 | H | -12.1396 | 4.315415 | 1.130907 |
| H | -12.1394 | 4.316836 | 1.130583 | H | -13.1265 | 1.126305 | 1.490886 |
| H | -13.127 | 1.127908 | 1.490162 | H | -10.6218 | 2.317604 | 3.36951 |
| H | -0.01841 | -9.21085 | -1.13687 | H | -2.85171 | -2.80081 | 2.984975 |
| H | -2.9916 | -12.0089 | 1.203933 | H | -5.7926 | -5.54824 | 5.271561 |
| H | -5.89438 | -10.1638 | 4.40624 | H | -5.89449 | -10.1636 | 4.405528 |
| H | -5.79298 | -5.54836 | 5.271712 | H | -2.99177 | -12.0086 | 1.203138 |
| H | -2.85255 | -2.80087 | 2.984686 | H | -0.01819 | -9.21063 | -1.13724 |
| **2B-15** | | | | **2B-16** | | | |
| Atom | X | Y | Z | Atom | X | Y | Z |
| C | 0.076549 | -1.08936 | -0.54915 | C | 0.033985 | 0.682201 | 1.678052 |
| C | -2.30529 | -0.04889 | -1.74297 | C | -2.49698 | -0.58044 | 2.095788 |
| C | -2.85629 | 2.731401 | -1.15708 | C | -2.90286 | -2.9977 | 0.53796 |
| C | -0.4362 | 4.30928 | -1.6721 | C | -0.63261 | -4.76933 | 1.153075 |
| C | 2.055943 | 3.184103 | -0.71604 | C | 2.017836 | -3.5806 | 1.038208 |
| C | 2.455049 | 0.381417 | -1.57322 | C | 2.223104 | -1.08785 | 2.629917 |
| C | -3.68012 | 2.908062 | 1.63137 | C | -2.9539 | -2.38754 | -2.29702 |
| O | -6.15392 | 3.450266 | 1.78961 | O | -5.14443 | -3.13336 | -3.33544 |
| C | -7.37494 | 3.478122 | -0.69097 | C | -6.94336 | -4.08117 | -1.46651 |
| C | -5.16874 | 3.768787 | -2.55459 | C | -5.46765 | -4.25884 | 1.033709 |
| C | 0.106635 | -3.96833 | -0.80113 | C | -0.01423 | 3.364535 | 2.749363 |
| C | -1.52406 | -5.4473 | 0.969628 | C | -1.63869 | 5.25287 | 1.413088 |
| O | 1.355954 | -5.04023 | -2.42201 | O | 1.178926 | 3.972616 | 4.632346 |
| C | 4.716186 | -0.64945 | -0.17111 | C | 4.709902 | 0.238098 | 2.082312 |
| C | 2.696365 | 0.160585 | -4.45527 | C | 1.992811 | -1.63634 | 5.475653 |
| C | 6.811824 | -1.67793 | -1.16853 | C | 5.3512 | 1.294937 | -0.13504 |
| C | 8.908904 | -2.58148 | 0.35648 | C | 7.801908 | 2.435848 | -0.60282 |
| C | 11.0783 | -3.63768 | -0.47315 | C | 8.676281 | 3.376088 | -2.80821 |
| C | 13.0779 | -4.4799 | 1.368704 | C | 11.3053 | 4.450793 | -2.98818 |
| C | 11.72045 | -4.07723 | -3.21007 | C | 7.199193 | 3.421251 | -5.24005 |
| C | 4.27631 | 4.927187 | -1.4732 | C | 3.91827 | -5.68563 | 1.782392 |
| C | 4.217697 | 7.428914 | -0.10653 | C | 6.680513 | -4.98394 | 1.858882 |
| C | 5.080884 | 7.865035 | 2.237264 | C | 8.193248 | -4.60964 | -0.13664 |
| C | 4.856152 | 10.43793 | 3.440862 | C | 10.91003 | -3.82535 | 0.192248 |
| C | 6.31346 | 5.880333 | 3.869781 | C | 7.290917 | -4.74454 | -2.83078 |
| C | -9.15092 | 1.102153 | -1.02337 | C | -9.27865 | -2.34262 | -1.53656 |
| C | -8.60635 | -0.98117 | 0.91835 | C | -11.3399 | -3.4329 | 0.163592 |
| O | -8.92151 | 0.106996 | -3.52374 | O | -10.2798 | -2.43087 | -4.05739 |
| C | -11.8951 | 1.977908 | -0.79011 | C | -8.65621 | 0.396504 | -0.83045 |
| O | -3.66591 | -1.32836 | -3.10457 | O | -4.02456 | 0.16061 | 3.649398 |
| O | -2.37696 | 2.611904 | 3.49327 | O | -1.30916 | -1.4032 | -3.55758 |
| C | -2.80026 | -4.36804 | 3.031894 | C | -2.6836 | 4.830246 | -0.98924 |
| C | -4.33015 | -5.85827 | 4.575917 | C | -4.20916 | 6.659482 | -2.11573 |
| C | -4.60018 | -8.43516 | 4.084732 | C | -4.71099 | 8.923984 | -0.85901 |
| C | -3.32816 | -9.52896 | 2.04221 | C | -3.66822 | 9.366235 | 1.528558 |
| C | -1.80265 | -8.04577 | 0.50128 | C | -2.13904 | 7.546453 | 2.650037 |
| H | -0.01792 | -0.59616 | 1.456442 | H | 0.289532 | 0.807248 | -0.36576 |
| H | -0.70235 | 6.183791 | -0.83934 | H | -0.71424 | -6.39067 | -0.13184 |
| H | -0.33564 | 4.589015 | -3.72215 | H | -0.97764 | -5.51826 | 3.053045 |
| H | 1.979862 | 3.162648 | 1.353076 | H | 2.400043 | -3.0489 | -0.92252 |
| H | -8.54032 | 5.177179 | -0.68202 | H | -7.52001 | -5.9361 | -2.16008 |
| H | -4.86684 | 5.776362 | -2.93703 | H | -5.21113 | -6.23477 | 1.568356 |
| H | -5.52272 | 2.826284 | -4.34656 | H | -6.43962 | -3.30599 | 2.581077 |
| H | 4.579912 | -0.49656 | 1.883033 | H | 6.068665 | 0.281964 | 3.625162 |
| H | 4.3412 | 1.2116 | -5.13213 | H | 2.038623 | 0.124688 | 6.542685 |
| H | 2.892666 | -1.81206 | -5.01154 | H | 0.234787 | -2.62268 | 5.938317 |
| H | 1.02968 | 0.923203 | -5.41353 | H | 3.566036 | -2.82203 | 6.103146 |
| H | 6.960553 | -1.85475 | -3.20757 | H | 4.015666 | 1.246354 | -1.69762 |
| H | 8.670194 | -2.36523 | 2.392299 | H | 9.066275 | 2.487318 | 1.023729 |
| H | 13.46644 | -6.50972 | 1.163557 | H | 12.29948 | 4.367267 | -1.17672 |
| H | 14.87548 | -3.504 | 1.008689 | H | 12.43325 | 3.41942 | -4.39505 |
| H | 12.52593 | -4.12027 | 3.32869 | H | 11.26099 | 6.430324 | -3.61577 |
| H | 10.2468 | -3.45691 | -4.51364 | H | 7.066296 | 5.358262 | -5.97455 |
| H | 13.47525 | -3.08647 | -3.70848 | H | 8.174041 | 2.309442 | -6.69816 |
| H | 12.07153 | -6.09405 | -3.55412 | H | 5.283198 | 2.678479 | -5.05464 |
| H | 4.218955 | 5.263123 | -3.51643 | H | 3.624669 | -7.23495 | 0.433622 |
| H | 6.04547 | 3.924846 | -1.09484 | H | 3.370599 | -6.44598 | 3.629691 |
| H | 3.334709 | 8.995703 | -1.10966 | H | 7.493225 | -4.73321 | 3.734208 |
| H | 3.760884 | 10.34051 | 5.202356 | H | 11.23256 | -1.96564 | -0.67087 |
| H | 6.724092 | 11.18555 | 3.954543 | H | 11.44057 | -3.70814 | 2.188361 |
| H | 3.936978 | 11.79892 | 2.183579 | H | 12.19886 | -5.15354 | -0.74954 |
| H | 5.18299 | 5.543054 | 5.578277 | H | 7.067024 | -2.82275 | -3.58912 |
| H | 6.555435 | 4.070542 | 2.906905 | H | 8.671315 | -5.71171 | -4.03871 |
| H | 8.177935 | 6.52129 | 4.518539 | H | 5.473414 | -5.70802 | -3.02458 |
| H | -6.68411 | -1.71709 | 0.752394 | H | -13.0634 | -2.31121 | -0.04907 |
| H | -9.90511 | -2.55183 | 0.570472 | H | -10.7788 | -3.3888 | 2.151924 |
| H | -8.87287 | -0.31512 | 2.854511 | H | -11.7646 | -5.38774 | -0.36512 |
| H | -7.23879 | -0.64224 | -3.65301 | H | -8.94617 | -1.83571 | -5.17188 |
| H | -12.3292 | 3.372226 | -2.25709 | H | -7.916 | 0.559338 | 1.090682 |
| H | -13.1777 | 0.3716 | -1.01146 | H | -7.26106 | 1.20131 | -2.13054 |
| H | -12.2281 | 2.843828 | 1.059309 | H | -10.3757 | 1.535115 | -0.96893 |
| H | -2.61823 | -2.37643 | 3.474267 | H | -2.30023 | 3.103248 | -2.02116 |
| H | -5.30796 | -5.00147 | 6.162743 | H | -5.00004 | 6.315015 | -3.97722 |
| H | -5.79599 | -9.58975 | 5.28821 | H | -5.90653 | 10.34223 | -1.73704 |
| H | -3.53528 | -11.5333 | 1.654875 | H | -4.05402 | 11.12659 | 2.509898 |
| H | -0.81035 | -8.86023 | -1.09591 | H | -1.31856 | 7.856175 | 4.502327 |
| **2B-17** | | | | **2B-18** | | | |
| Atom | X | Y | Z | Atom | X | Y | Z |
| C | -0.46839 | -1.35508 | 0.379037 | C | -0.04307 | 0.928576 | -0.64703 |
| C | 1.909627 | -0.37556 | 1.631727 | C | 2.414976 | 0.100428 | -1.86323 |
| C | 2.835283 | 2.198194 | 0.684334 | C | 3.325376 | -2.54594 | -1.09986 |
| C | 0.570382 | 4.068775 | 0.857922 | C | 1.094499 | -4.39036 | -1.64949 |
| C | -1.94227 | 3.098495 | -0.20359 | C | -1.51601 | -3.56737 | -0.68169 |
| C | -2.73872 | 0.492049 | 0.952397 | C | -2.26234 | -0.84142 | -1.57571 |
| C | 3.748808 | 2.001141 | -2.06932 | C | 3.990079 | -2.61983 | 1.724354 |
| O | 6.167596 | 2.738088 | -2.25294 | O | 6.397511 | -3.33978 | 2.064509 |
| C | 7.379105 | 2.859581 | 0.215897 | C | 7.765382 | -3.61248 | -0.30209 |
| C | 5.174397 | 3.185948 | 2.084478 | C | 5.783809 | -3.34241 | -2.41567 |
| C | -0.85367 | -4.14158 | 1.057375 | C | -0.36372 | 3.7736 | -1.03563 |
| C | 0.841508 | -6.03098 | -0.18394 | C | 1.286185 | 5.475527 | 0.508875 |
| O | -2.46808 | -4.82684 | 2.561803 | O | -1.8415 | 4.66056 | -2.57386 |
| C | -4.98329 | -0.47271 | -0.52071 | C | -4.58569 | -0.00868 | -0.14372 |
| C | -3.24955 | 0.700567 | 3.801543 | C | -2.59746 | -0.71065 | -4.45456 |
| C | -7.23738 | -1.19006 | 0.399993 | C | -6.6768 | 1.074077 | -1.08868 |
| C | -9.29439 | -2.07519 | -1.18921 | C | -8.82996 | 1.747449 | 0.474599 |
| C | -11.6068 | -2.84633 | -0.43512 | C | -11.044 | 2.763231 | -0.28603 |
| C | -13.5365 | -3.71982 | -2.33616 | C | -13.1086 | 3.317834 | 1.59313 |
| C | -12.4842 | -2.92199 | 2.270759 | C | -11.6746 | 3.425992 | -2.98052 |
| C | -3.99465 | 5.15458 | 0.110379 | C | -3.44169 | -5.65426 | -1.40143 |
| C | -3.53704 | 7.376221 | -1.60156 | C | -6.0438 | -5.35368 | -0.28022 |
| C | -2.72275 | 9.695011 | -0.97677 | C | -6.73429 | -5.93656 | 2.087478 |
| C | -2.32289 | 11.69083 | -2.97385 | C | -9.38639 | -5.44356 | 3.004536 |
| C | -2.12413 | 10.54429 | 1.676629 | C | -4.96479 | -7.01944 | 4.039675 |
| C | 9.000168 | 0.450819 | 0.542202 | C | 9.948537 | -1.68158 | -0.33182 |
| C | 10.87124 | 0.210956 | -1.64679 | C | 9.021966 | 1.05232 | -0.11263 |
| O | 7.402552 | -1.73064 | 0.382804 | O | 11.08269 | -2.11316 | -2.75844 |
| C | 10.40331 | 0.522473 | 3.073084 | C | 11.83453 | -2.29796 | 1.776849 |
| O | 2.951253 | -1.50872 | 3.354619 | O | 3.5012 | 1.371242 | -3.44581 |
| O | 2.529886 | 1.378534 | -3.90881 | O | 2.623743 | -2.15352 | 3.507332 |
| C | 0.511804 | -8.58795 | 0.449885 | C | 2.880185 | 4.600682 | 2.442052 |
| C | 2.04808 | -10.438 | -0.60802 | C | 4.415164 | 6.285605 | 3.764164 |
| C | 3.950089 | -9.75562 | -2.31222 | C | 4.373563 | 8.857322 | 3.178342 |
| C | 4.29773 | -7.22062 | -2.95153 | C | 2.782644 | 9.747703 | 1.265189 |
| C | 2.749561 | -5.36519 | -1.90235 | C | 1.252828 | 8.068343 | -0.05608 |
| H | -0.15827 | -1.21555 | -1.66268 | H | 0.122568 | 0.553845 | 1.37782 |
| H | 1.094049 | 5.81852 | -0.11179 | H | 1.57418 | -6.23554 | -0.84254 |
| H | 0.351022 | 4.555674 | 2.859139 | H | 1.03598 | -4.65412 | -3.70274 |
| H | -1.68546 | 2.786182 | -2.23609 | H | -1.43059 | -3.50588 | 1.385918 |
| H | 8.605862 | 4.51668 | 0.167647 | H | 8.583009 | -5.50661 | -0.27376 |
| H | 4.927455 | 5.187595 | 2.524393 | H | 5.529669 | -5.14691 | -3.38351 |
| H | 5.466131 | 2.182545 | 3.859435 | H | 6.36124 | -1.94859 | -3.81642 |
| H | -4.68127 | -0.56183 | -2.56081 | H | -4.48685 | -0.33723 | 1.88883 |
| H | -4.80958 | 2.004295 | 4.169101 | H | -2.94304 | 1.229174 | -5.05073 |
| H | -3.72846 | -1.14291 | 4.582437 | H | -0.91244 | -1.38264 | -5.4466 |
| H | -1.59157 | 1.40261 | 4.818089 | H | -4.19276 | -1.87932 | -5.05336 |
| H | -7.5512 | -1.12344 | 2.426633 | H | -6.80089 | 1.42355 | -3.10634 |
| H | -8.88929 | -2.11117 | -3.20991 | H | -8.6075 | 1.349578 | 2.485067 |
| H | -12.8125 | -3.6312 | -4.27102 | H | -12.5608 | 2.799427 | 3.518674 |
| H | -14.1255 | -5.67374 | -1.9504 | H | -13.606 | 5.334515 | 1.578968 |
| H | -15.2576 | -2.56205 | -2.23134 | H | -14.8468 | 2.287105 | 1.112497 |
| H | -11.0608 | -2.27847 | 3.618192 | H | -12.1324 | 5.44466 | -3.14173 |
| H | -14.1763 | -1.74437 | 2.515167 | H | -10.1497 | 3.012514 | -4.30734 |
| H | -13.0366 | -4.85265 | 2.797101 | H | -13.3646 | 2.393877 | -3.60482 |
| H | -4.0926 | 5.740655 | 2.090725 | H | -2.61684 | -7.4582 | -0.79826 |
| H | -5.82966 | 4.306272 | -0.34823 | H | -3.58471 | -5.76594 | -3.46422 |
| H | -3.86453 | 6.992285 | -3.6014 | H | -7.46645 | -4.52693 | -1.51069 |
| H | -3.50881 | 13.35618 | -2.61029 | H | -9.37632 | -4.13617 | 4.618946 |
| H | -0.35748 | 12.36075 | -2.97029 | H | -10.5685 | -4.6154 | 1.523289 |
| H | -2.76173 | 10.98542 | -4.86781 | H | -10.2938 | -7.1892 | 3.669795 |
| H | -2.47738 | 9.092302 | 3.099468 | H | -3.12131 | -7.53482 | 3.264099 |
| H | -0.1299 | 11.10286 | 1.821821 | H | -4.64342 | -5.65193 | 5.569682 |
| H | -3.24042 | 12.22098 | 2.177225 | H | -5.78666 | -8.70718 | 4.924932 |
| H | 9.866581 | 0.082158 | -3.44572 | H | 7.711814 | 1.533072 | -1.63302 |
| H | 12.00775 | -1.49786 | -1.39773 | H | 10.64037 | 2.339717 | -0.22378 |
| H | 12.13971 | 1.843617 | -1.71101 | H | 8.089589 | 1.387042 | 1.703252 |
| H | 6.425902 | -1.86648 | 1.937455 | H | 12.50636 | -0.96539 | -2.90553 |
| H | 11.59204 | -1.16021 | 3.243828 | H | 12.56465 | -4.22069 | 1.55059 |
| H | 11.61427 | 2.195257 | 3.206355 | H | 13.43105 | -0.98015 | 1.713394 |
| H | 9.09699 | 0.54803 | 4.677726 | H | 10.94615 | -2.13926 | 3.637163 |
| H | -0.96375 | -9.07951 | 1.784261 | H | 2.934011 | 2.619455 | 2.956507 |
| H | 1.773956 | -12.4089 | -0.10625 | H | 5.638784 | 5.586578 | 5.255359 |
| H | 5.156966 | -11.197 | -3.13579 | H | 5.573331 | 10.16499 | 4.209134 |
| H | 5.77609 | -6.68022 | -4.26717 | H | 2.744776 | 11.74729 | 0.8057 |
| H | 3.063764 | -3.41607 | -2.43941 | H | 0.016448 | 8.72393 | -1.55315 |
| **2B-19** | | | |  |  |  |  |
| Atom | X | Y | Z |  |  |  |  |
| C | 0.166345 | 0.623398 | -0.58154 |  |  |  |  |
| C | 2.605832 | -0.38873 | -1.67267 |  |  |  |  |
| C | 3.277475 | -3.0831 | -0.86479 |  |  |  |  |
| C | 0.935596 | -4.8028 | -1.27014 |  |  |  |  |
| C | -1.62856 | -3.73479 | -0.42971 |  |  |  |  |
| C | -2.14048 | -1.02933 | -1.51406 |  |  |  |  |
| C | 4.072679 | -3.0083 | 1.937017 |  |  |  |  |
| O | 6.565787 | -3.42339 | 2.159923 |  |  |  |  |
| C | 7.818302 | -3.57736 | -0.30029 |  |  |  |  |
| C | 5.652054 | -4.11911 | -2.15498 |  |  |  |  |
| C | 0.018901 | 3.470903 | -1.05597 |  |  |  |  |
| C | 1.48675 | 5.15663 | 0.672805 |  |  |  |  |
| O | -1.1804 | 4.357563 | -2.82017 |  |  |  |  |
| C | -4.44977 | 0.058829 | -0.24258 |  |  |  |  |
| C | -2.33616 | -1.04438 | -4.40812 |  |  |  |  |
| C | -6.41257 | 1.224236 | -1.35074 |  |  |  |  |
| C | -8.57405 | 2.150251 | 0.064402 |  |  |  |  |
| C | -10.6731 | 3.266358 | -0.86331 |  |  |  |  |
| C | -12.7684 | 4.090766 | 0.877521 |  |  |  |  |
| C | -11.14 | 3.788419 | -3.62116 |  |  |  |  |
| C | -3.68525 | -5.71662 | -1.07781 |  |  |  |  |
| C | -6.28266 | -5.16395 | -0.04484 |  |  |  |  |
| C | -7.06433 | -5.54848 | 2.335006 |  |  |  |  |
| C | -9.69366 | -4.8146 | 3.150952 |  |  |  |  |
| C | -5.42052 | -6.63172 | 4.393865 |  |  |  |  |
| C | 9.47644 | -1.14564 | -0.79616 |  |  |  |  |
| C | 8.79779 | 1.050089 | 0.972551 |  |  |  |  |
| O | 9.235184 | -0.36088 | -3.36882 |  |  |  |  |
| C | 12.25804 | -1.85639 | -0.46435 |  |  |  |  |
| O | 3.919233 | 0.842886 | -3.12258 |  |  |  |  |
| O | 2.732242 | -2.63462 | 3.758015 |  |  |  |  |
| C | 2.770425 | 4.287957 | 2.828128 |  |  |  |  |
| C | 4.15046 | 5.965949 | 4.320018 |  |  |  |  |
| C | 4.258702 | 8.523502 | 3.686453 |  |  |  |  |
| C | 2.977101 | 9.408474 | 1.550849 |  |  |  |  |
| C | 1.604689 | 7.737349 | 0.059375 |  |  |  |  |
| H | 0.253074 | 0.285991 | 1.455282 |  |  |  |  |
| H | 1.280909 | -6.59059 | -0.28701 |  |  |  |  |
| H | 0.874698 | -5.25667 | -3.29016 |  |  |  |  |
| H | -1.58888 | -3.54715 | 1.632122 |  |  |  |  |
| H | 9.066866 | -5.20959 | -0.15181 |  |  |  |  |
| H | 5.448411 | -6.16277 | -2.3782 |  |  |  |  |
| H | 5.984572 | -3.30611 | -4.01339 |  |  |  |  |
| H | -4.45974 | -0.14232 | 1.808646 |  |  |  |  |
| H | -2.50566 | 0.876865 | -5.12755 |  |  |  |  |
| H | -0.66288 | -1.89441 | -5.2774 |  |  |  |  |
| H | -3.98475 | -2.12968 | -5.0192 |  |  |  |  |
| H | -6.42508 | 1.447566 | -3.39004 |  |  |  |  |
| H | -8.46632 | 1.8726 | 2.104106 |  |  |  |  |
| H | -14.5557 | 3.155843 | 0.382027 |  |  |  |  |
| H | -12.3422 | 3.665649 | 2.855871 |  |  |  |  |
| H | -13.1141 | 6.132038 | 0.710367 |  |  |  |  |
| H | -11.4411 | 5.818796 | -3.93259 |  |  |  |  |
| H | -9.59623 | 3.179561 | -4.84695 |  |  |  |  |
| H | -12.874 | 2.838327 | -4.25457 |  |  |  |  |
| H | -3.0069 | -7.53626 | -0.35164 |  |  |  |  |
| H | -3.78996 | -5.93823 | -3.13403 |  |  |  |  |
| H | -7.61462 | -4.31746 | -1.36027 |  |  |  |  |
| H | -10.7391 | -6.44863 | 3.892645 |  |  |  |  |
| H | -9.62508 | -3.41675 | 4.686158 |  |  |  |  |
| H | -10.7797 | -3.99566 | 1.592967 |  |  |  |  |
| H | -3.60256 | -7.32267 | 3.698882 |  |  |  |  |
| H | -5.03527 | -5.19934 | 5.84781 |  |  |  |  |
| H | -6.38127 | -8.19986 | 5.355818 |  |  |  |  |
| H | 10.01553 | 2.656605 | 0.513242 |  |  |  |  |
| H | 9.076523 | 0.55143 | 2.956743 |  |  |  |  |
| H | 6.841153 | 1.666625 | 0.735973 |  |  |  |  |
| H | 7.51912 | 0.28893 | -3.57577 |  |  |  |  |
| H | 12.7848 | -3.33609 | -1.8125 |  |  |  |  |
| H | 13.45951 | -0.2072 | -0.79775 |  |  |  |  |
| H | 12.60788 | -2.55682 | 1.450917 |  |  |  |  |
| H | 2.712485 | 2.317116 | 3.382391 |  |  |  |  |
| H | 5.137323 | 5.270512 | 5.978564 |  |  |  |  |
| H | 5.335999 | 9.82499 | 4.851364 |  |  |  |  |
| H | 3.057739 | 11.39743 | 1.052899 |  |  |  |  |
| H | 0.609212 | 8.388058 | -1.60928 |  |  |  |  |

**Table S9.** Gibbs free energies^a^ and equilibrium populations^b^ of low-energy conformers of **2C**.

| Conformers | ∆G(a.u.) | P(%)/100 | G(a.u.) |
| --- | --- | --- | --- |
| **2C-1** | 0.01466 | 0.0 | -1580.907771 |
| **2C-2** | 0.01542 | 0.0 | -1580.907011 |
| **2C-3** | 0.01542 | 0.0 | -1580.907007 |
| **2C-4** | 0.0162 | 0.0 | -1580.906232 |
| **2C-5** | 0.01683 | 0.0 | -1580.905605 |
| **2C-6** | 0.01597 | 0.0 | -1580.906466 |
| **2C-7** | 0.01652 | 0.0 | -1580.905914 |
| **2C-8** | 0.01618 | 0.0 | -1580.906247 |
| **2C-9** | 0.01814 | 0.0 | -1580.904293 |
| **2C-10** | 0.01283 | 0.0 | -1580.909601 |
| **2C-11** | 0.01795 | 0.0 | -1580.904477 |
| **2C-12** | 0.01724 | 0.0 | -1580.905196 |
| **2C-13** | 0.01953 | 0.0 | -1580.902897 |
| **2C-14** | 0.01834 | 0.0 | -1580.904092 |
| **2C-15** | 0.00132 | 16.56 | -1580.921112 |
| **2C-16** | 0.00132 | 16.52 | -1580.92111 |
| **2C-17** | 0.0149 | 0.0 | -1580.907534 |
| **2C-18** | 0.01508 | 0.0 | -1580.907349 |
| **2C-19** | 0.0 | 66.92 | -1580.922431 |

^a^wB97M-V/def2-TZVP, in a.u.
^b^From ∆G values at 298.15K.

**Table S10.** Cartesian coordinates for the low-energy reoptimized random research conformers of **2C** at B3LYP-D3(BJ)/6-31G* level of theory in methanol.

| **2C-1** | | | | **2C-2** | | | |
| --- | --- | --- | --- | --- | --- | --- | --- |
| Atom | X | Y | Z | Atom | X | Y | Z |
| C | 1.316476 | 1.837776 | -1.78196 | C | -2.50859 | -0.20271 | -1.58835 |
| C | -0.31622 | 1.181128 | 0.503552 | C | -0.88793 | -1.30629 | 0.527719 |
| C | -0.14747 | -1.51792 | 1.530058 | C | 0.587854 | 0.548009 | 2.188484 |
| C | 2.192232 | -2.8635 | 0.482407 | C | -0.4332 | 3.238112 | 1.861841 |
| C | 2.310872 | -2.95954 | -2.42032 | C | -0.36717 | 4.193718 | -0.88112 |
| C | 1.433964 | -0.3892 | -3.69501 | C | -1.20281 | 2.100773 | -2.85933 |
| C | 0.081559 | -1.23789 | 4.406652 | C | 0.177277 | -0.35351 | 4.912679 |
| O | -2.05143 | -1.98192 | 5.567815 | O | 2.30145 | -1.36969 | 5.866335 |
| C | -3.88874 | -3.0778 | 3.832055 | C | 4.462293 | -1.11482 | 4.176452 |
| C | -2.67519 | -2.95642 | 1.181969 | C | 3.478067 | 0.264475 | 1.80291 |
| C | 3.891687 | 2.868954 | -0.86609 | C | -5.21541 | 0.265342 | -0.59669 |
| C | 4.027963 | 5.534021 | 0.034185 | C | -6.98011 | -1.92304 | -0.42507 |
| O | 5.782968 | 1.538906 | -0.88513 | O | -5.90657 | 2.383899 | 0.018882 |
| C | -1.20649 | -0.8586 | -4.73242 | C | 1.207567 | 1.20248 | -4.14556 |
| C | 3.080131 | 0.411123 | -5.97117 | C | -2.93612 | 3.121574 | -4.97704 |
| C | -3.35356 | 0.437122 | -4.3167 | C | 2.121082 | -1.15796 | -4.38071 |
| C | -5.80672 | -0.34332 | -5.27383 | C | 4.574039 | -1.73524 | -5.47267 |
| C | -8.04382 | 0.721038 | -4.66155 | C | 5.693503 | -4.0288 | -5.48265 |
| C | -10.4872 | -0.3635 | -5.63141 | C | 8.326009 | -4.35844 | -6.50708 |
| C | -8.30669 | 2.966229 | -2.92081 | C | 4.495655 | -6.38719 | -4.41508 |
| C | 4.939245 | -3.93639 | -3.25422 | C | -1.7796 | 6.749335 | -1.0603 |
| C | 5.517904 | -6.50707 | -2.1867 | C | -0.696 | 8.701841 | 0.713799 |
| C | 7.154261 | -7.08677 | -0.33861 | C | 1.296923 | 10.20466 | 0.267323 |
| C | 7.493435 | -9.78307 | 0.524972 | C | 2.225954 | 12.0482 | 2.232203 |
| C | 8.761732 | -5.18893 | 1.053296 | C | 2.782563 | 10.20046 | -2.1647 |
| C | -6.44072 | -1.69186 | 4.082141 | C | 5.645844 | -3.73026 | 3.672584 |
| C | -7.15731 | -1.37981 | 6.873764 | C | 5.973415 | -5.1925 | 6.153861 |
| O | -6.37672 | 0.728748 | 2.879712 | O | 4.149408 | -5.17665 | 1.948037 |
| C | -8.45634 | -3.21138 | 2.667798 | C | 8.195791 | -3.35133 | 2.358588 |
| O | -1.68786 | 2.748051 | 1.505796 | O | -0.78401 | -3.58288 | 0.911693 |
| O | 1.889932 | -0.43948 | 5.552038 | O | -1.76791 | -0.23059 | 6.10436 |
| C | 1.947705 | 7.18197 | 0.091346 | C | -6.31304 | -4.41067 | -1.06787 |
| C | 2.239089 | 9.651232 | 0.961581 | C | -8.08083 | -6.35206 | -0.85406 |
| C | 4.599495 | 10.50519 | 1.771582 | C | -10.5266 | -5.83574 | -0.01254 |
| C | 6.681824 | 8.877512 | 1.722678 | C | -11.208 | -3.36448 | 0.632801 |
| C | 6.396109 | 6.413084 | 0.863374 | C | -9.44756 | -1.42691 | 0.431322 |
| H | 0.370925 | 3.415487 | -2.71612 | H | -2.67141 | -1.69107 | -3.00668 |
| H | 2.242984 | -4.79004 | 1.226774 | H | 0.675827 | 4.518201 | 3.04806 |
| H | 3.86586 | -1.90312 | 1.21393 | H | -2.37085 | 3.285967 | 2.572649 |
| H | 0.942909 | -4.3934 | -3.03052 | H | 1.612925 | 4.625889 | -1.31093 |
| H | -4.13939 | -5.02714 | 4.464393 | H | 5.830236 | 0.033837 | 5.211693 |
| H | -2.29828 | -4.85053 | 0.456715 | H | 4.33821 | 2.131167 | 1.625786 |
| H | -3.92917 | -2.00941 | -0.14269 | H | 3.931821 | -0.80496 | 0.107799 |
| H | -1.30887 | -2.5165 | -5.95426 | H | 2.325601 | 2.747059 | -4.93097 |
| H | 2.378353 | 2.206085 | -6.72671 | H | -3.46434 | 1.569431 | -6.24092 |
| H | 5.062652 | 0.644106 | -5.4555 | H | -4.6566 | 3.977594 | -4.22997 |
| H | 2.958041 | -0.99035 | -7.48616 | H | -1.94152 | 4.534727 | -6.11194 |
| H | -3.34452 | 2.092945 | -3.10907 | H | 1.077917 | -2.74995 | -3.62076 |
| H | -5.81354 | -1.99138 | -6.50998 | H | 5.615021 | -0.13719 | -6.25177 |
| H | -11.5333 | 1.041829 | -6.74608 | H | 8.348117 | -5.71623 | -8.07793 |
| H | -10.183 | -2.03666 | -6.80797 | H | 9.130244 | -2.57381 | -7.17382 |
| H | -11.7287 | -0.89545 | -4.05339 | H | 9.585878 | -5.13451 | -5.04937 |
| H | -6.67208 | 4.229506 | -2.99261 | H | 2.430859 | -6.34179 | -4.48934 |
| H | -9.98524 | 4.075291 | -3.41056 | H | 5.130293 | -8.06022 | -5.45775 |
| H | -8.5078 | 2.342667 | -0.95322 | H | 5.027221 | -6.63919 | -2.42654 |
| H | 6.367761 | -2.55485 | -2.71219 | H | -3.77426 | 6.442949 | -0.62142 |
| H | 4.970753 | -4.07117 | -5.32107 | H | -1.68457 | 7.43598 | -3.0069 |
| H | 4.427728 | -8.05341 | -3.00982 | H | -1.60136 | 8.846016 | 2.558122 |
| H | 6.280635 | -11.0883 | -0.52561 | H | 4.203955 | 11.66502 | 2.735452 |
| H | 9.4632 | -10.3985 | 0.292768 | H | 2.173232 | 13.99521 | 1.511722 |
| H | 7.056285 | -9.97557 | 2.545979 | H | 1.092936 | 11.9667 | 3.960703 |
| H | 8.316479 | -5.22474 | 3.081026 | H | 4.752455 | 9.648732 | -1.81041 |
| H | 10.77441 | -5.67055 | 0.888253 | H | 2.013121 | 8.912648 | -3.58304 |
| H | 8.50308 | -3.25355 | 0.387861 | H | 2.847872 | 12.10483 | -2.98819 |
| H | -9.01881 | -0.48929 | 7.00516 | H | 6.909185 | -6.9949 | 5.765239 |
| H | -7.23247 | -3.20741 | 7.844444 | H | 7.124175 | -4.13212 | 7.51018 |
| H | -5.78322 | -0.18347 | 7.847696 | H | 4.137389 | -5.5756 | 7.020081 |
| H | -4.74705 | 1.540252 | 3.17385 | H | 2.357362 | -4.94151 | 2.313131 |
| H | -10.2526 | -2.19027 | 2.749049 | H | 9.002442 | -5.20049 | 1.904789 |
| H | -7.94794 | -3.45107 | 0.677025 | H | 7.973622 | -2.30104 | 0.59043 |
| H | -8.72379 | -5.07626 | 3.520873 | H | 9.523723 | -2.33885 | 3.57848 |
| H | 0.092043 | 6.547678 | -0.48704 | H | -4.41577 | -4.8614 | -1.683 |
| H | 0.618562 | 10.90837 | 1.004514 | H | -7.5448 | -8.26971 | -1.34788 |
| H | 4.818694 | 12.43261 | 2.441572 | H | -11.9 | -7.35288 | 0.142557 |
| H | 8.519263 | 9.535726 | 2.355313 | H | -13.1083 | -2.95813 | 1.291004 |
| H | 7.988301 | 5.124709 | 0.815851 | H | -9.94168 | 0.498676 | 0.926674 |
| **2C-3** | | | | **2C-4** | | | |
| Atom | X | Y | Z | Atom | X | Y | Z |
| C | -2.50896 | -0.2036 | -1.58666 | C | -1.6269 | 1.098836 | 1.094042 |
| C | -0.88741 | -1.30655 | 0.52904 | C | 0.843916 | 1.475768 | -0.34085 |
| C | 0.588831 | 0.548215 | 2.188879 | C | 1.181746 | 0.023657 | -2.81954 |
| C | -0.43278 | 3.238125 | 1.862278 | C | -1.36449 | -0.99183 | -3.75395 |
| C | -0.36805 | 4.193355 | -0.88085 | C | -2.73428 | -2.71245 | -1.84994 |
| C | -1.20408 | 2.099994 | -2.85843 | C | -2.55342 | -1.6877 | 0.965833 |
| C | 0.179652 | -0.35287 | 4.913437 | C | 2.244528 | 1.949681 | -4.70755 |
| O | 2.304457 | -1.36839 | 5.866369 | O | 4.683155 | 1.465243 | -5.22726 |
| C | 4.464487 | -1.11354 | 4.175373 | C | 5.577935 | -0.87498 | -4.08137 |
| C | 3.478891 | 0.265019 | 1.801984 | C | 3.314806 | -1.97259 | -2.604 |
| C | -5.21563 | 0.264174 | -0.59439 | C | -3.56596 | 3.107528 | 0.234489 |
| C | -6.98047 | -1.92414 | -0.42354 | C | -3.37263 | 5.705796 | 1.302787 |
| O | -5.9066 | 2.382538 | 0.022074 | O | -5.23502 | 2.578416 | -1.27563 |
| C | 1.20594 | 1.201994 | -4.1455 | C | -0.64347 | -3.36387 | 2.311143 |
| C | -2.93844 | 3.120208 | -4.9756 | C | -5.03991 | -1.89946 | 2.482157 |
| C | 2.11984 | -1.15829 | -4.38068 | C | 1.459891 | -2.70758 | 3.578591 |
| C | 4.572476 | -1.73517 | -5.47363 | C | 3.244664 | -4.52379 | 4.607706 |
| C | 5.692407 | -4.02849 | -5.48389 | C | 5.508304 | -3.95359 | 5.638863 |
| C | 8.32454 | -4.35771 | -6.50944 | C | 7.28631 | -6.00795 | 6.47848 |
| C | 4.495401 | -6.38703 | -4.41568 | C | 6.480481 | -1.28836 | 5.947917 |
| C | -1.78107 | 6.748667 | -1.05983 | C | -5.40947 | -3.286 | -2.8809 |
| C | -0.69702 | 8.701754 | 0.713368 | C | -6.52718 | -5.71457 | -1.91648 |
| C | 1.295249 | 10.20505 | 0.265582 | C | -8.83525 | -6.11778 | -0.9508 |
| C | 2.224843 | 12.04915 | 2.229699 | C | -9.63301 | -8.722 | -0.10671 |
| C | 2.779583 | 10.20092 | -2.16724 | C | -10.8081 | -4.09842 | -0.57523 |
| C | 5.648138 | -3.72894 | 3.671587 | C | 7.955358 | -0.35257 | -2.47844 |
| C | 5.977179 | -5.19051 | 6.153065 | C | 9.834592 | 1.289672 | -3.95311 |
| O | 4.15098 | -5.17598 | 1.948183 | O | 7.328785 | 0.851025 | -0.14065 |
| C | 8.19736 | -3.35002 | 2.356163 | C | 9.152221 | -2.88525 | -1.75369 |
| O | -0.78311 | -3.58306 | 0.913448 | O | 2.491787 | 2.899851 | 0.432887 |
| O | -1.76504 | -0.23014 | 6.105958 | O | 1.118028 | 3.711492 | -5.62722 |
| C | -9.4485 | -1.42785 | 0.431092 | C | -5.1982 | 7.475632 | 0.526293 |
| C | -11.2091 | -3.36538 | 0.631734 | C | -5.15188 | 9.934835 | 1.444873 |
| C | -10.5272 | -5.83678 | -0.01257 | C | -3.27168 | 10.66814 | 3.152216 |
| C | -8.08083 | -6.35329 | -0.85223 | C | -1.44456 | 8.931152 | 3.927779 |
| C | -6.31292 | -4.41192 | -1.06531 | C | -1.49166 | 6.45957 | 3.017509 |
| H | -2.67188 | -1.69219 | -3.00473 | H | -1.20243 | 1.523499 | 3.067245 |
| H | 0.676562 | 4.518558 | 3.047841 | H | -1.05098 | -2.04854 | -5.5063 |
| H | -2.37014 | 3.285763 | 2.573877 | H | -2.56741 | 0.616459 | -4.23057 |
| H | 1.611778 | 4.625868 | -1.31154 | H | -1.72138 | -4.52089 | -1.85917 |
| H | 5.83273 | 0.035574 | 5.209707 | H | 6.111983 | -2.08674 | -5.66533 |
| H | 4.338694 | 2.131777 | 1.623919 | H | 2.685752 | -3.75215 | -3.43659 |
| H | 3.931997 | -0.80479 | 0.106938 | H | 3.836745 | -2.33868 | -0.65018 |
| H | 2.323337 | 2.746683 | -4.93159 | H | -1.08615 | -5.37183 | 2.157173 |
| H | -1.94465 | 4.533614 | -6.11089 | H | -6.58904 | -0.88092 | 1.582245 |
| H | -3.46665 | 1.56786 | -6.23923 | H | -5.60179 | -3.87352 | 2.682338 |
| H | -4.65893 | 3.975757 | -4.228 | H | -4.74928 | -1.11457 | 4.376046 |
| H | 1.077301 | -2.75042 | -3.62016 | H | 1.975869 | -0.73339 | 3.771444 |
| H | 5.612795 | -0.13696 | -6.25329 | H | 2.723494 | -6.50862 | 4.426128 |
| H | 9.128205 | -2.57294 | -7.17652 | H | 6.515859 | -7.89481 | 6.128354 |
| H | 9.585144 | -5.13359 | -5.05227 | H | 9.10371 | -5.85358 | 5.48456 |
| H | 8.346187 | -5.71548 | -8.08032 | H | 7.714873 | -5.83888 | 8.503214 |
| H | 2.430575 | -6.34221 | -4.48963 | H | 7.776845 | -1.16787 | 7.557875 |
| H | 5.130329 | -8.06008 | -5.45816 | H | 7.512226 | -0.68448 | 4.253284 |
| H | 5.027363 | -6.63853 | -2.42719 | H | 4.964229 | 0.082927 | 6.255199 |
| H | -3.77545 | 6.441971 | -0.61991 | H | -5.20717 | -3.45999 | -4.94491 |
| H | -1.68711 | 7.434948 | -3.00662 | H | -6.63796 | -1.66218 | -2.56763 |
| H | -1.60142 | 8.845946 | 2.55816 | H | -5.27136 | -7.34473 | -2.07665 |
| H | 2.171314 | 13.99602 | 1.508912 | H | -10.1357 | -8.72553 | 1.90857 |
| H | 1.0927 | 11.96768 | 3.958774 | H | -11.3241 | -9.35592 | -1.13167 |
| H | 4.203178 | 11.66649 | 2.732026 | H | -8.13735 | -10.1201 | -0.40086 |
| H | 2.010037 | 8.912321 | -3.58481 | H | -10.2082 | -2.23945 | -1.23915 |
| H | 2.843458 | 12.1051 | -2.9913 | H | -12.5609 | -4.59656 | -1.56907 |
| H | 4.749944 | 9.650315 | -1.81385 | H | -11.2997 | -3.93299 | 1.435519 |
| H | 4.141664 | -5.57356 | 7.02038 | H | 9.019555 | 3.144748 | -4.35623 |
| H | 6.912909 | -6.99291 | 5.764377 | H | 11.54508 | 1.551945 | -2.82134 |
| H | 7.128589 | -4.12966 | 7.508475 | H | 10.36324 | 0.40676 | -5.74999 |
| H | 2.3591 | -4.94092 | 2.314119 | H | 5.992522 | 2.093955 | -0.4056 |
| H | 9.004039 | -5.19919 | 1.902484 | H | 10.78811 | -2.53596 | -0.53815 |
| H | 7.974115 | -2.30027 | 0.587818 | H | 7.817549 | -4.06601 | -0.70331 |
| H | 9.525749 | -2.33699 | 3.575103 | H | 9.788615 | -3.91914 | -3.42786 |
| H | -9.94296 | 0.497845 | 0.925709 | H | -6.63918 | 6.875731 | -0.80078 |
| H | -13.1099 | -2.95889 | 1.288474 | H | -6.57364 | 11.28305 | 0.836141 |
| H | -11.9007 | -7.3539 | 0.141898 | H | -3.23245 | 12.58923 | 3.873151 |
| H | -7.54442 | -8.27106 | -1.34516 | H | 0.021467 | 9.497875 | 5.246139 |
| H | -4.41518 | -4.86276 | -1.67892 | H | -0.03185 | 5.156498 | 3.611008 |
| **2C-5** | | | | **2C-6** | | | |
| Atom | X | Y | Z | Atom | X | Y | Z |
| C | -2.45388 | -0.04329 | 1.196881 | C | 1.951584 | 0.422582 | -2.83 |
| C | -0.70983 | 1.61212 | -0.39319 | C | 0.325855 | 1.703743 | -0.83057 |
| C | 0.536276 | 0.401144 | -2.70552 | C | -0.36354 | 0.113472 | 1.494904 |
| C | -0.79528 | -2.07711 | -3.39103 | C | 0.855804 | -2.51505 | 1.378362 |
| C | -0.83544 | -4.02725 | -1.23545 | C | 0.466982 | -3.95128 | -1.12687 |
| C | -1.44027 | -2.7919 | 1.430307 | C | 0.681208 | -2.17446 | -3.49809 |
| C | 0.239026 | 2.329581 | -4.84847 | C | 0.77728 | 1.623628 | 3.699596 |
| O | 2.470267 | 3.396596 | -5.42875 | O | -1.04139 | 2.713879 | 5.089091 |
| C | 4.583329 | 2.268337 | -4.06772 | C | -3.57394 | 1.899583 | 4.362966 |
| C | 3.438693 | 0.193104 | -2.3674 | C | -3.2333 | 0.163495 | 2.042682 |
| C | -5.19315 | 0.204744 | 0.213138 | C | 4.768161 | 0.39116 | -2.07083 |
| C | -6.68867 | 2.471414 | 0.958392 | C | 6.133297 | 2.849282 | -1.90943 |
| O | -6.12354 | -1.42793 | -1.13395 | O | 5.870221 | -1.59118 | -1.62401 |
| C | 1.046904 | -2.74648 | 2.87266 | C | -1.90692 | -1.37243 | -4.49316 |
| C | -3.30545 | -4.33043 | 3.068027 | C | 2.100213 | -3.36266 | -5.7536 |
| C | 2.245477 | -0.78164 | 3.951049 | C | -4.23301 | -1.95737 | -3.65909 |
| C | 4.734718 | -0.96119 | 5.09744 | C | -6.52459 | -0.88699 | -4.73067 |
| C | 6.119111 | 1.011072 | 5.937567 | C | -8.90219 | -1.18303 | -3.85234 |
| C | 8.75684 | 0.625188 | 6.926916 | C | -11.0903 | 0.110441 | -5.1313 |
| C | 5.213762 | 3.717445 | 5.871638 | C | -9.6095 | -2.74434 | -1.58048 |
| C | -2.50241 | -6.28644 | -2.06656 | C | 2.222518 | -6.2944 | -1.16841 |
| C | -2.069 | -8.71294 | -0.62669 | C | 1.75315 | -8.02326 | 1.039038 |
| C | -0.04055 | -10.2227 | -0.81504 | C | 3.234221 | -8.38835 | 3.064406 |
| C | 0.198499 | -12.5809 | 0.766906 | C | 2.464375 | -10.1899 | 5.13641 |
| C | 2.153991 | -9.72481 | -2.56485 | C | 5.726114 | -7.07663 | 3.505259 |
| C | 6.071331 | 4.336457 | -2.65328 | C | -5.28737 | 4.214603 | 3.912404 |
| C | 6.586867 | 6.591072 | -4.40104 | C | -4.92118 | 6.178216 | 6.013069 |
| O | 4.750685 | 5.172581 | -0.44782 | O | -4.82494 | 5.351563 | 1.504887 |
| C | 8.550032 | 3.195045 | -1.69153 | C | -8.03279 | 3.29884 | 3.831465 |
| O | -0.3308 | 3.830773 | 0.131417 | O | -0.38154 | 3.892256 | -1.02597 |
| O | -1.7055 | 2.89367 | -5.90633 | O | 3.004005 | 1.89579 | 4.140099 |
| C | -5.74198 | 4.426687 | 2.483884 | C | 5.12003 | 5.151342 | -2.75619 |
| C | -7.26793 | 6.485062 | 3.095347 | C | 6.524429 | 7.370219 | -2.54665 |
| C | -9.74802 | 6.611139 | 2.206454 | C | 8.941058 | 7.318571 | -1.49085 |
| C | -10.7069 | 4.673521 | 0.685215 | C | 9.96364 | 5.034117 | -0.64049 |
| C | -9.18786 | 2.623826 | 0.064708 | C | 8.572355 | 2.817502 | -0.85478 |
| H | -2.44775 | 0.785427 | 3.085545 | H | 1.826388 | 1.631327 | -4.50291 |
| H | 0.160831 | -2.90803 | -5.02906 | H | 0.125903 | -3.65427 | 2.938505 |
| H | -2.72487 | -1.64062 | -3.97977 | H | 2.878121 | -2.29258 | 1.720558 |
| H | 1.087821 | -4.77331 | -1.08128 | H | -1.45206 | -4.7147 | -1.11469 |
| H | 5.804882 | 1.466766 | -5.52633 | H | -4.29455 | 0.859963 | 5.994485 |
| H | 4.072836 | -1.67802 | -2.96153 | H | -3.90425 | -1.74275 | 2.451368 |
| H | 4.010896 | 0.462221 | -0.4117 | H | -4.3034 | 0.897981 | 0.446894 |
| H | 1.948595 | -4.59827 | 2.971158 | H | -1.78587 | -0.06846 | -6.08818 |
| H | -3.6991 | -3.29272 | 4.816097 | H | 4.035255 | -3.87442 | -5.26475 |
| H | -5.09159 | -4.67894 | 2.098409 | H | 1.107945 | -5.05067 | -6.41628 |
| H | -2.47631 | -6.14723 | 3.587377 | H | 2.161068 | -2.01759 | -7.32688 |
| H | 1.419613 | 1.093316 | 3.88869 | H | -4.46522 | -3.23529 | -2.07451 |
| H | 5.561963 | -2.84612 | 5.179737 | H | -6.25446 | 0.321172 | -6.37733 |
| H | 9.335474 | -1.35856 | 6.846988 | H | -12.4985 | -1.27489 | -5.77153 |
| H | 10.12025 | 1.744582 | 5.830219 | H | -12.0741 | 1.374591 | -3.80985 |
| H | 8.916593 | 1.277245 | 8.8919 | H | -10.4842 | 1.223908 | -6.76445 |
| H | 3.170484 | 3.879033 | 6.14435 | H | -10.5157 | -1.55141 | -0.14336 |
| H | 6.138023 | 4.832548 | 7.350863 | H | -11.0039 | -4.19023 | -2.10193 |
| H | 5.654117 | 4.592212 | 4.043453 | H | -8.00782 | -3.69562 | -0.69453 |
| H | -2.09428 | -6.60367 | -4.07683 | H | 4.181951 | -5.6665 | -1.23174 |
| H | -4.48824 | -5.73405 | -1.97177 | H | 1.862359 | -7.35546 | -2.91068 |
| H | -3.54329 | -9.26741 | 0.696389 | H | -0.03296 | -9.05432 | 0.970766 |
| H | -1.42921 | -12.8368 | 2.01723 | H | 2.3213 | -9.20619 | 6.959812 |
| H | 0.3722 | -14.2692 | -0.42977 | H | 0.642889 | -11.087 | 4.741756 |
| H | 1.910942 | -12.5179 | 1.940339 | H | 3.882629 | -11.6855 | 5.390047 |
| H | 2.600333 | -11.4214 | -3.67307 | H | 6.258374 | -5.79592 | 1.97818 |
| H | 1.801351 | -8.17578 | -3.88426 | H | 5.651577 | -5.97034 | 5.260873 |
| H | 3.866546 | -9.26127 | -1.48569 | H | 7.251314 | -8.46319 | 3.753002 |
| H | 7.728621 | 7.989144 | -3.39245 | H | -2.9976 | 6.930544 | 5.975497 |
| H | 7.612029 | 6.008498 | -6.10327 | H | -6.24634 | 7.739445 | 5.725366 |
| H | 4.815504 | 7.481184 | -4.98237 | H | -5.26435 | 5.359796 | 7.884153 |
| H | 2.942048 | 5.276624 | -0.79174 | H | -3.02862 | 5.246651 | 1.096007 |
| H | 9.57507 | 4.624882 | -0.60502 | H | -8.28849 | 1.843466 | 2.383132 |
| H | 8.193941 | 1.581782 | -0.44674 | H | -8.62207 | 2.531396 | 5.659301 |
| H | 9.74901 | 2.580194 | -3.2604 | H | -9.26363 | 4.892119 | 3.360859 |
| H | -3.81196 | 4.388403 | 3.158124 | H | 3.239287 | 5.239226 | -3.55469 |
| H | -6.51673 | 7.990253 | 4.269445 | H | 5.728006 | 9.140579 | -3.21011 |
| H | -10.9325 | 8.21446 | 2.69456 | H | 10.02672 | 9.052559 | -1.3281 |
| H | -12.6344 | 4.768731 | -0.01152 | H | 11.84049 | 4.990141 | 0.187304 |
| H | -9.89818 | 1.10714 | -1.11534 | H | 9.333886 | 1.029908 | -0.20482 |
| **2C-7** | | | | **2C-8** | | | |
| Atom | X | Y | Z | Atom | X | Y | Z |
| C | 2.488066 | -1.16819 | -2.2988 | C | 2.841154 | 0.032874 | -1.4983 |
| C | 1.680205 | 1.114303 | -0.74364 | C | 1.178288 | 1.428557 | 0.411824 |
| C | 0.232544 | 0.572991 | 1.711423 | C | -0.68385 | -0.14728 | 1.958958 |
| C | 0.015201 | -2.27711 | 2.195217 | C | -0.01106 | -2.97269 | 1.834476 |
| C | -1.01232 | -3.80354 | -0.05954 | C | 0.12581 | -4.05692 | -0.85833 |
| C | 0.119417 | -2.91508 | -2.65755 | C | 1.422429 | -2.18178 | -2.80728 |
| C | 1.879212 | 1.805509 | 3.760181 | C | -0.48148 | 0.700196 | 4.722853 |
| O | 0.766274 | 3.887443 | 4.686969 | O | -2.75086 | 1.407729 | 5.633217 |
| C | -1.8126 | 4.219539 | 3.768739 | C | -4.7217 | 1.301817 | 3.732848 |
| C | -2.27281 | 2.071527 | 1.84847 | C | -3.44305 | 0.495384 | 1.241442 |
| C | 4.902222 | -2.40423 | -1.22395 | C | 5.358434 | -0.68358 | -0.21305 |
| C | 7.314633 | -0.95638 | -1.35389 | C | 7.384207 | 1.267088 | -0.04446 |
| O | 4.852401 | -4.52193 | -0.29895 | O | 5.700569 | -2.80683 | 0.639289 |
| C | -1.71394 | -1.22249 | -4.11064 | C | -0.71635 | -1.1206 | -4.40602 |
| C | 0.823781 | -5.09964 | -4.45995 | C | 3.255635 | -3.50379 | -4.6567 |
| C | -4.06617 | -0.47268 | -3.51574 | C | -1.49958 | 1.280941 | -4.66895 |
| C | -5.52186 | 1.282517 | -5.04648 | C | -3.74277 | 1.987304 | -6.09411 |
| C | -7.77976 | 2.31012 | -4.44769 | C | -4.9832 | 4.210414 | -5.94239 |
| C | -9.03962 | 4.162732 | -6.20186 | C | -7.35981 | 4.65658 | -7.44171 |
| C | -9.22315 | 1.771777 | -2.05423 | C | -4.23678 | 6.352953 | -4.22081 |
| C | -0.77963 | -6.66181 | 0.522044 | C | 1.244774 | -6.75874 | -0.75782 |
| C | -2.20256 | -7.3825 | 2.888715 | C | -0.25817 | -8.48202 | 0.946545 |
| C | -4.70205 | -7.76869 | 3.072302 | C | -2.34282 | -9.78137 | 0.317446 |
| C | -5.92108 | -8.41421 | 5.565856 | C | -3.70478 | -11.402 | 2.225591 |
| C | -6.5047 | -7.58055 | 0.871802 | C | -3.51817 | -9.74931 | -2.27859 |
| C | -2.16462 | 6.907253 | 2.696819 | C | -6.06316 | 3.889965 | 3.646028 |
| C | -0.98553 | 8.874738 | 4.469293 | C | -4.1965 | 6.076138 | 3.313839 |
| O | -1.10088 | 7.147116 | 0.224426 | O | -7.67561 | 3.67085 | 1.468347 |
| C | -5.00245 | 7.383959 | 2.376727 | C | -7.66031 | 4.252657 | 6.032053 |
| O | 2.166518 | 3.278883 | -1.38094 | O | 1.302456 | 3.713027 | 0.68529 |
| O | 3.940045 | 1.092662 | 4.446104 | O | 1.402317 | 0.688626 | 6.0152 |
| C | 7.589552 | 1.32337 | -2.68479 | C | 7.070282 | 3.78515 | -0.82302 |
| C | 9.91449 | 2.561539 | -2.71957 | C | 9.059984 | 5.497087 | -0.59744 |
| C | 11.98005 | 1.546482 | -1.43094 | C | 11.38012 | 4.71663 | 0.385075 |
| C | 11.7224 | -0.72232 | -0.0992 | C | 11.70937 | 2.212786 | 1.166209 |
| C | 9.409453 | -1.96499 | -0.06658 | C | 9.724818 | 0.505734 | 0.958977 |
| H | 3.00748 | -0.41766 | -4.15387 | H | 3.298679 | 1.419617 | -2.95531 |
| H | -1.211 | -2.5795 | 3.831784 | H | -1.41308 | -4.04188 | 2.916318 |
| H | 1.881553 | -2.98799 | 2.714854 | H | 1.808646 | -3.23725 | 2.773859 |
| H | -3.0434 | -3.46086 | -0.13878 | H | -1.82676 | -4.29335 | -1.50848 |
| H | -3.01009 | 4.019092 | 5.438335 | H | -6.07002 | -0.12684 | 4.371333 |
| H | -3.79686 | 0.836425 | 2.484126 | H | -4.39636 | -1.13326 | 0.414003 |
| H | -2.79638 | 2.855219 | 0.020199 | H | -3.54699 | 2.015589 | -0.1376 |
| H | -0.91754 | -0.49675 | -5.8708 | H | -1.77694 | -2.58808 | -5.39423 |
| H | 1.613938 | -4.33182 | -6.21352 | H | 4.095934 | -2.09357 | -5.91852 |
| H | 2.21337 | -6.37043 | -3.62395 | H | 4.774149 | -4.4934 | -3.67417 |
| H | -0.86223 | -6.18513 | -4.96169 | H | 2.244418 | -4.86737 | -5.83674 |
| H | -4.94983 | -1.13266 | -1.78927 | H | -0.50634 | 2.798662 | -3.71641 |
| H | -4.64948 | 1.850512 | -6.82421 | H | -4.53058 | 0.52194 | -7.30957 |
| H | -10.916 | 3.482144 | -6.77447 | H | -8.95446 | 5.090864 | -6.18382 |
| H | -9.34395 | 5.985543 | -5.25485 | H | -7.14677 | 6.295186 | -8.69941 |
| H | -7.92062 | 4.496877 | -7.90791 | H | -7.87008 | 3.015878 | -8.59204 |
| H | -8.30007 | 0.404859 | -0.81501 | H | -5.54632 | 6.442721 | -2.61204 |
| H | -9.50019 | 3.522861 | -0.97403 | H | -2.32925 | 6.171233 | -3.45204 |
| H | -11.1191 | 1.054273 | -2.49858 | H | -4.36216 | 8.171452 | -5.2082 |
| H | 1.215037 | -7.15452 | 0.722752 | H | 3.196071 | -6.6483 | -0.08989 |
| H | -1.50947 | -7.73492 | -1.08656 | H | 1.301194 | -7.5354 | -2.67177 |
| H | -1.0851 | -7.53196 | 4.612175 | H | 0.398362 | -8.62225 | 2.893639 |
| H | -6.90181 | -10.242 | 5.465848 | H | -2.78353 | -11.3503 | 4.07674 |
| H | -4.53567 | -8.50553 | 7.09896 | H | -5.67122 | -10.778 | 2.462954 |
| H | -7.36013 | -7.00662 | 6.076473 | H | -3.79134 | -13.3797 | 1.597686 |
| H | -7.64382 | -9.30763 | 0.706652 | H | -3.70207 | -11.6776 | -3.02485 |
| H | -7.84476 | -6.02059 | 1.160679 | H | -5.4401 | -8.96867 | -2.19501 |
| H | -5.55226 | -7.26387 | -0.93236 | H | -2.44355 | -8.63206 | -3.64204 |
| H | 1.060085 | 8.611507 | 4.587541 | H | -5.24833 | 7.834493 | 3.01358 |
| H | -1.36554 | 10.76937 | 3.733158 | H | -3.02642 | 6.311959 | 5.00086 |
| H | -1.77147 | 8.741427 | 6.380638 | H | -2.94627 | 5.778423 | 1.698905 |
| H | 0.440616 | 6.140342 | 0.104489 | H | -8.67002 | 5.209497 | 1.372056 |
| H | -5.8657 | 5.946375 | 1.164459 | H | -6.47437 | 4.207298 | 7.725968 |
| H | -5.97336 | 7.387682 | 4.202825 | H | -8.63318 | 6.080638 | 5.979566 |
| H | -5.28185 | 9.226399 | 1.481012 | H | -9.08311 | 2.757515 | 6.177158 |
| H | 6.005843 | 2.158655 | -3.67307 | H | 5.270544 | 4.438889 | -1.54153 |
| H | 10.11055 | 4.32115 | -3.75586 | H | 8.795927 | 7.442641 | -1.19244 |
| H | 13.78691 | 2.519268 | -1.46114 | H | 12.92874 | 6.053876 | 0.5454 |
| H | 13.32477 | -1.5126 | 0.909582 | H | 13.51119 | 1.601516 | 1.934178 |
| H | 9.174414 | -3.72408 | 0.957124 | H | 9.944211 | -1.44062 | 1.559975 |
| **2C-9** | | | | **2C-10** | | | |
| Atom | X | Y | Z | Atom | X | Y | Z |
| C | -2.17538 | -1.7842 | 1.385422 | C | 1.787479 | 1.930499 | -1.57287 |
| C | -2.40465 | 0.935679 | 0.47212 | C | -0.04121 | 1.413217 | 0.608603 |
| C | -1.1184 | 1.587372 | -2.0382 | C | -0.52389 | -1.35372 | 1.299379 |
| C | -0.31944 | -0.82217 | -3.43104 | C | 1.524794 | -3.0766 | 0.173575 |
| C | 1.470906 | -2.49075 | -1.863 | C | 1.791893 | -2.88103 | -2.7162 |
| C | 0.589224 | -2.74313 | 0.983438 | C | 1.512542 | -0.06534 | -3.71183 |
| C | -3.10647 | 3.039587 | -3.56247 | C | -0.41353 | -1.55306 | 4.188429 |
| O | -2.50068 | 5.499198 | -3.73184 | O | -2.58365 | -2.49731 | 5.125642 |
| C | -0.01855 | 6.067712 | -2.67769 | C | -4.43296 | -3.03793 | 3.161164 |
| C | 1.029111 | 3.541989 | -1.65712 | C | -3.26939 | -2.12395 | 0.655261 |
| C | -4.29835 | -3.44316 | 0.264868 | C | 4.477952 | 2.295186 | -0.51074 |
| C | -6.90362 | -3.17162 | 1.299965 | C | 5.157503 | 4.796493 | 0.593769 |
| O | -3.85601 | -4.96584 | -1.41837 | O | 6.033174 | 0.583644 | -0.57556 |
| C | 2.224712 | -1.06191 | 2.674153 | C | -1.13044 | 0.127018 | -4.82935 |
| C | 0.778849 | -5.4496 | 2.070059 | C | 3.371557 | 0.599185 | -5.86306 |
| C | 4.755388 | -0.86628 | 2.553096 | C | -3.00652 | 1.741694 | -4.25489 |
| C | 6.239857 | 0.693267 | 4.252415 | C | -5.53253 | 1.562252 | -5.32135 |
| C | 8.774267 | 0.994378 | 4.236658 | C | -7.60196 | 2.83424 | -4.53818 |
| C | 10.0434 | 2.712177 | 6.11676 | C | -10.1275 | 2.402649 | -5.77676 |
| C | 10.54234 | -0.29263 | 2.416158 | C | -7.63047 | 4.723384 | -2.40848 |
| C | 1.996135 | -4.97366 | -3.31841 | C | 4.21918 | -4.27759 | -3.5643 |
| C | 4.399674 | -6.30689 | -2.54486 | C | 4.21791 | -7.00815 | -2.77272 |
| C | 6.763795 | -5.53309 | -3.04295 | C | 5.607211 | -8.09526 | -0.95144 |
| C | 9.025546 | -6.9943 | -2.10861 | C | 5.3589 | -10.8787 | -0.38409 |
| C | 7.383614 | -3.18971 | -4.54501 | C | 7.484309 | -6.71727 | 0.692056 |
| C | -0.23144 | 8.197057 | -0.69672 | C | -6.957 | -1.81227 | 3.926562 |
| C | -1.93984 | 10.32838 | -1.66736 | C | -8.96853 | -2.45367 | 1.956503 |
| O | -1.16718 | 7.266411 | 1.661069 | O | -7.77654 | -2.98336 | 6.234475 |
| C | 2.429477 | 9.190337 | -0.14315 | C | -6.73087 | 1.048569 | 4.317248 |
| O | -3.53967 | 2.54274 | 1.68101 | O | -1.10284 | 3.111757 | 1.744446 |
| O | -5.00748 | 2.170787 | -4.48595 | O | 1.363434 | -1.01778 | 5.519884 |
| C | -7.53356 | -1.53165 | 3.289939 | C | 7.656195 | 5.109682 | 1.445161 |
| C | -10.0193 | -1.39043 | 4.150855 | C | 8.437652 | 7.389084 | 2.48845 |
| C | -11.8946 | -2.88151 | 3.047773 | C | 6.727202 | 9.392606 | 2.707622 |
| C | -11.2848 | -4.51922 | 1.064292 | C | 4.239459 | 9.098542 | 1.88198 |
| C | -8.81006 | -4.66141 | 0.198678 | C | 3.452955 | 6.818272 | 0.822556 |
| H | -2.51517 | -1.71345 | 3.421825 | H | 1.223656 | 3.755464 | -2.35182 |
| H | 0.635466 | -0.28107 | -5.18713 | H | 1.115371 | -5.03726 | 0.683487 |
| H | -2.00908 | -1.87659 | -3.96887 | H | 3.323 | -2.59553 | 1.065473 |
| H | 3.270044 | -1.48751 | -1.80971 | H | 0.209659 | -3.93595 | -3.54225 |
| H | 1.097409 | 6.761799 | -4.26998 | H | -4.69126 | -5.08701 | 3.183068 |
| H | 2.697172 | 2.955564 | -2.71861 | H | -3.31207 | -3.61254 | -0.76926 |
| H | 1.568119 | 3.724801 | 0.320666 | H | -4.32786 | -0.53353 | -0.10527 |
| H | 1.247026 | -0.04719 | 4.178683 | H | -1.50817 | -1.31301 | -6.25633 |
| H | 0.090017 | -5.46184 | 4.023183 | H | 3.074499 | 2.563393 | -6.44507 |
| H | -0.33286 | -6.80416 | 0.982453 | H | 5.338731 | 0.374325 | -5.28684 |
| H | 2.747157 | -6.06189 | 2.096736 | H | 3.033999 | -0.59676 | -7.51556 |
| H | 5.758627 | -1.92927 | 1.108819 | H | -2.70802 | 3.210633 | -2.85904 |
| H | 5.16091 | 1.73094 | 5.668812 | H | -5.76133 | 0.200353 | -6.85037 |
| H | 11.41908 | 1.665058 | 7.267269 | H | -11.5233 | 1.73307 | -4.39282 |
| H | 11.11186 | 4.206935 | 5.148687 | H | -10.8823 | 4.172901 | -6.55708 |
| H | 8.681455 | 3.608149 | 7.388532 | H | -10.0127 | 1.016051 | -7.30612 |
| H | 11.58671 | 1.114305 | 1.302919 | H | -8.29064 | 6.571494 | -3.08391 |
| H | 11.96642 | -1.39478 | 3.44828 | H | -8.97052 | 4.124452 | -0.94104 |
| H | 9.589546 | -1.56455 | 1.10151 | H | -5.79296 | 4.991096 | -1.5087 |
| H | 2.110544 | -4.46757 | -5.32793 | H | 5.859147 | -3.27679 | -2.82309 |
| H | 0.374147 | -6.23676 | -3.14322 | H | 4.336801 | -4.20258 | -5.63108 |
| H | 4.179154 | -8.04011 | -1.45915 | H | 2.887403 | -8.20777 | -3.79643 |
| H | 10.23572 | -5.8084 | -0.90784 | H | 7.177386 | -11.8532 | -0.62096 |
| H | 8.466659 | -8.65628 | -1.01186 | H | 4.784804 | -11.1857 | 1.587899 |
| H | 10.21296 | -7.62533 | -3.69079 | H | 3.96971 | -11.7959 | -1.61176 |
| H | 8.251053 | -1.73886 | -3.33863 | H | 7.645943 | -4.7103 | 0.243493 |
| H | 8.771126 | -3.62302 | -6.02533 | H | 6.938532 | -6.87675 | 2.689054 |
| H | 5.728442 | -2.34771 | -5.44799 | H | 9.368368 | -7.57441 | 0.531225 |
| H | -3.87516 | 9.651576 | -1.92183 | H | -10.8056 | -1.76611 | 2.609568 |
| H | -1.95541 | 11.87059 | -0.28996 | H | -8.55121 | -1.56293 | 0.137782 |
| H | -1.26825 | 11.07116 | -3.47992 | H | -9.09967 | -4.50033 | 1.680087 |
| H | -2.54318 | 6.077879 | 1.352252 | H | -6.47632 | -2.64189 | 7.486458 |
| H | 2.326803 | 10.589 | 1.376127 | H | -6.13977 | 2.031497 | 2.600154 |
| H | 3.684505 | 7.6651 | 0.472672 | H | -8.56972 | 1.793258 | 4.897811 |
| H | 3.25 | 10.09375 | -1.81255 | H | -5.35047 | 1.48188 | 5.794855 |
| H | -6.11689 | -0.33138 | 4.146809 | H | 8.954632 | 3.535133 | 1.265276 |
| H | -10.489 | -0.11437 | 5.686955 | H | 10.373 | 7.611424 | 3.132563 |
| H | -13.8274 | -2.7689 | 3.728467 | H | 7.334089 | 11.17584 | 3.522387 |
| H | -12.7409 | -5.67806 | 0.200332 | H | 2.905475 | 10.6469 | 2.060469 |
| H | -8.30374 | -5.91771 | -1.33846 | H | 1.503551 | 6.612904 | 0.240192 |
| **2C-11** | | | | **2C-12** | | | |
| Atom | X | Y | Z | Atom | X | Y | Z |
| C | -2.11392 | 0.870344 | 1.664087 | C | 2.800688 | -1.16809 | -2.21093 |
| C | 0.435408 | 1.778595 | 0.693894 | C | 1.617698 | 1.177451 | -1.01238 |
| C | 1.16416 | 0.974356 | -1.99544 | C | 0.085984 | 0.752164 | 1.417317 |
| C | -0.96682 | -0.54036 | -3.25106 | C | 0.047782 | -2.0577 | 2.17361 |
| C | -2.0024 | -2.75606 | -1.66481 | C | -0.65586 | -3.88018 | 0.013682 |
| C | -2.27975 | -2.05926 | 1.212084 | C | 0.634281 | -3.16762 | -2.56545 |
| C | 1.537133 | 3.501765 | -3.37703 | C | 1.487044 | 2.242281 | 3.476365 |
| O | 3.992404 | 3.919385 | -3.85334 | O | -0.044 | 3.9436 | 4.581635 |
| C | 5.58041 | 1.742239 | -3.26825 | C | -2.59537 | 3.851846 | 3.579054 |
| C | 3.813845 | -0.25461 | -2.08269 | C | -2.52995 | 2.029611 | 1.308407 |
| C | -4.30669 | 2.500712 | 0.640916 | C | 5.203246 | -2.04551 | -0.82339 |
| C | -4.50275 | 5.172069 | 1.510864 | C | 7.500801 | -0.42074 | -0.98183 |
| O | -5.86079 | 1.629819 | -0.83222 | O | 5.256986 | -4.03731 | 0.351686 |
| C | -0.08366 | -3.06954 | 2.788924 | C | -1.18794 | -1.80598 | -4.34321 |
| C | -4.71827 | -3.04223 | 2.476817 | C | 1.689776 | -5.45169 | -4.04113 |
| C | 1.94439 | -4.43176 | 2.096173 | C | -3.57456 | -1.03634 | -3.94744 |
| C | 3.963619 | -5.10462 | 3.83303 | C | -4.99377 | 0.49586 | -5.73119 |
| C | 6.170362 | -6.24109 | 3.241083 | C | -7.20336 | 1.680877 | -5.25702 |
| C | 8.11764 | -6.75841 | 5.250578 | C | -8.41002 | 3.346482 | -7.22231 |
| C | 6.92188 | -7.06201 | 0.621659 | C | -8.58663 | 1.559993 | -2.76734 |
| C | -4.38881 | -3.79612 | -3.00171 | C | -0.24577 | -6.63385 | 0.91788 |
| C | -4.94815 | -6.51959 | -2.41225 | C | -1.79863 | -7.238 | 3.234387 |
| C | -7.15114 | -7.56538 | -1.72323 | C | -4.25739 | -7.85709 | 3.27049 |
| C | -7.35219 | -10.3686 | -1.24105 | C | -5.62194 | -8.35652 | 5.721941 |
| C | -9.57411 | -6.12153 | -1.32863 | C | -5.87204 | -8.09086 | 0.933007 |
| C | 7.815284 | 2.546629 | -1.57861 | C | -3.46765 | 6.563034 | 2.980346 |
| C | 9.003464 | 5.001529 | -2.56068 | C | -1.67429 | 7.921437 | 1.161186 |
| O | 7.061827 | 2.859197 | 0.99753 | O | -5.89528 | 6.160184 | 1.825368 |
| C | 9.756119 | 0.398126 | -1.5716 | C | -3.77971 | 8.070699 | 5.43157 |
| O | 1.842821 | 3.10016 | 1.959472 | O | 1.830041 | 3.278539 | -1.9215 |
| O | -0.10343 | 4.996487 | -3.92452 | O | 3.671626 | 1.970119 | 4.094447 |
| C | -2.97907 | 6.211082 | 3.419541 | C | 7.624136 | 1.801483 | -2.4275 |
| C | -3.28271 | 8.729894 | 4.128844 | C | 9.841739 | 3.222968 | -2.47997 |
| C | -5.09965 | 10.23336 | 2.947429 | C | 11.95187 | 2.445502 | -1.10131 |
| C | -6.62495 | 9.213819 | 1.044365 | C | 11.84464 | 0.234687 | 0.343751 |
| C | -6.33085 | 6.702356 | 0.33657 | C | 9.636687 | -1.18617 | 0.400482 |
| H | -2.07417 | 1.186585 | 3.706798 | H | 3.413267 | -0.56998 | -4.09265 |
| H | -0.27507 | -1.27112 | -5.05951 | H | -1.29105 | -2.30719 | 3.729337 |
| H | -2.50772 | 0.759759 | -3.69122 | H | 1.909674 | -2.56352 | 2.904794 |
| H | -0.61644 | -4.28105 | -1.78365 | H | -2.69319 | -3.73038 | -0.26999 |
| H | 6.328375 | 1.115454 | -5.08747 | H | -3.78597 | 3.099686 | 5.091253 |
| H | 3.760581 | -1.96224 | -3.23726 | H | -4.04414 | 0.643618 | 1.46981 |
| H | 4.481661 | -0.76412 | -0.20507 | H | -2.80313 | 3.044757 | -0.45921 |
| H | -0.19925 | -2.51346 | 4.773002 | H | -0.32246 | -1.29392 | -6.14552 |
| H | -6.41054 | -2.30147 | 1.56626 | H | 2.575678 | -4.79986 | -5.79599 |
| H | -4.78168 | -5.10168 | 2.403543 | H | 3.097259 | -6.48983 | -2.95224 |
| H | -4.74708 | -2.46346 | 4.465404 | H | 0.159835 | -6.74137 | -4.56078 |
| H | 2.165464 | -5.03398 | 0.150405 | H | -4.51012 | -1.47187 | -2.1793 |
| H | 3.658066 | -4.57048 | 5.799175 | H | -4.11566 | 0.782182 | -7.57271 |
| H | 8.547679 | -8.78698 | 5.360739 | H | -8.65994 | 5.276947 | -6.49791 |
| H | 9.908208 | -5.80561 | 4.805443 | H | -7.28399 | 3.445294 | -8.95373 |
| H | 7.491906 | -6.12444 | 7.116317 | H | -10.3071 | 2.659088 | -7.71302 |
| H | 5.484446 | -6.69121 | -0.81079 | H | -8.18404 | 3.250919 | -1.63134 |
| H | 8.6644 | -6.09559 | 0.0389 | H | -10.6351 | 1.502969 | -3.075 |
| H | 7.344746 | -9.09369 | 0.593202 | H | -8.07351 | -0.08405 | -1.62759 |
| H | -4.00917 | -3.65927 | -5.04328 | H | 1.758371 | -6.92259 | 1.321973 |
| H | -5.99127 | -2.55527 | -2.63815 | H | -0.7467 | -7.92511 | -0.61608 |
| H | -3.32692 | -7.78019 | -2.61864 | H | -0.82703 | -7.08798 | 5.044093 |
| H | -5.54614 | -11.3331 | -1.5347 | H | -7.22419 | -7.05678 | 5.959389 |
| H | -7.97715 | -10.7424 | 0.704171 | H | -6.41631 | -10.275 | 5.756147 |
| H | -8.76476 | -11.2435 | -2.48666 | H | -4.37155 | -8.14409 | 7.355846 |
| H | -9.3931 | -4.10432 | -1.72156 | H | -4.80392 | -7.85374 | -0.81774 |
| H | -11.0803 | -6.87632 | -2.5412 | H | -6.81123 | -9.9408 | 0.871723 |
| H | -10.2331 | -6.33647 | 0.628835 | H | -7.3836 | -6.66717 | 0.948467 |
| H | 9.598301 | 4.818683 | -4.53586 | H | -1.37001 | 6.840712 | -0.57042 |
| H | 7.656252 | 6.562123 | -2.42934 | H | -2.47167 | 9.765177 | 0.657226 |
| H | 10.66188 | 5.46282 | -1.4148 | H | 0.163886 | 8.244937 | 2.049516 |
| H | 5.356493 | 3.560006 | 1.070687 | H | -6.5728 | 7.807545 | 1.387371 |
| H | 11.28312 | 0.871081 | -0.26029 | H | -1.97863 | 8.230793 | 6.435283 |
| H | 8.898941 | -1.37661 | -0.94157 | H | -4.45555 | 9.983954 | 5.015828 |
| H | 10.56934 | 0.115141 | -3.45197 | H | -5.15295 | 7.146827 | 6.673024 |
| H | -1.53939 | 5.087825 | 4.340633 | H | 5.998764 | 2.455298 | -3.48396 |
| H | -2.09769 | 9.516642 | 5.607116 | H | 9.919354 | 4.939797 | -3.60055 |
| H | -5.32792 | 12.19526 | 3.504869 | H | 13.67599 | 3.557861 | -1.14925 |
| H | -8.03613 | 10.38087 | 0.118996 | H | 13.48197 | -0.37169 | 1.421601 |
| H | -7.49462 | 5.880822 | -1.13595 | H | 9.517591 | -2.90283 | 1.512667 |
| **2C-13** | | | | **2C-14** | | | |
| Atom | X | Y | Z | Atom | X | Y | Z |
| C | 1.340528 | 1.572012 | -1.39016 | C | -1.99655 | -2.32287 | 1.468567 |
| C | -0.38274 | 0.807952 | 0.784646 | C | -2.27484 | 0.530932 | 1.198117 |
| C | -0.49409 | -1.99189 | 1.501609 | C | -1.34199 | 1.680276 | -1.28976 |
| C | 1.723744 | -3.43735 | 0.324243 | C | -0.40057 | -0.37299 | -3.1242 |
| C | 1.76621 | -3.24383 | -2.56484 | C | 1.565275 | -2.15575 | -1.93857 |
| C | 1.395801 | -0.42694 | -3.54903 | C | 0.80223 | -3.02887 | 0.817858 |
| C | -0.33872 | -2.06819 | 4.393046 | C | -3.61177 | 3.007733 | -2.50172 |
| O | -2.5824 | -2.69684 | 5.408118 | O | -3.05548 | 5.422924 | -3.06711 |
| C | -4.45861 | -3.39005 | 3.514103 | C | -0.50613 | 6.107281 | -2.31578 |
| C | -3.13997 | -3.11297 | 0.926884 | C | 0.557847 | 3.839557 | -0.82474 |
| C | 3.933716 | 2.348353 | -0.29067 | C | -4.0791 | -3.77018 | 0.042419 |
| C | 4.271945 | 5.00462 | 0.587238 | C | -6.72023 | -3.60071 | 1.009711 |
| O | 5.672124 | 0.834694 | -0.15957 | O | -3.59501 | -5.05181 | -1.82218 |
| C | -1.16727 | -0.41445 | -4.85744 | C | 2.40904 | -1.63379 | 2.774881 |
| C | 3.355095 | 0.40497 | -5.54415 | C | 1.169196 | -5.8827 | 1.332665 |
| C | -3.25195 | 0.932591 | -4.30769 | C | 4.912441 | -1.22674 | 2.614553 |
| C | -5.66822 | 0.593484 | -5.56874 | C | 6.382032 | -0.00657 | 4.583572 |
| C | -7.83852 | 1.759915 | -4.90689 | C | 8.892579 | 0.452075 | 4.560888 |
| C | -10.2808 | 1.170126 | -6.23696 | C | 10.15042 | 1.756225 | 6.754892 |
| C | -8.00554 | 3.654369 | -2.77248 | C | 10.64412 | -0.25597 | 2.432813 |
| C | 3.998473 | -4.81147 | -3.66499 | C | 2.216452 | -4.26273 | -3.86204 |
| C | 6.584271 | -4.33851 | -2.58639 | C | 4.737679 | -5.52848 | -3.42226 |
| C | 7.824519 | -5.75691 | -0.88985 | C | 7.018284 | -4.49794 | -3.83809 |
| C | 10.41968 | -4.99618 | 0.016008 | C | 9.414897 | -5.93197 | -3.26945 |
| C | 6.820184 | -8.14214 | 0.302851 | C | 7.407297 | -1.87564 | -4.88819 |
| C | -6.86583 | -1.78277 | 3.854399 | C | -0.62452 | 8.617618 | -0.84627 |
| C | -7.64296 | -1.69762 | 6.645838 | C | -2.47117 | 8.491968 | 1.380694 |
| O | -6.5202 | 0.732683 | 2.925942 | O | 1.927027 | 8.914439 | 0.030799 |
| C | -8.97282 | -2.92815 | 2.235932 | C | -1.32324 | 10.78299 | -2.63584 |
| O | -1.61664 | 2.379893 | 1.94875 | O | -3.10363 | 1.866857 | 2.878253 |
| O | 1.501045 | -1.61203 | 5.667555 | O | -5.64151 | 2.066977 | -2.97276 |
| C | 6.723092 | 5.738632 | 1.306734 | C | -8.61868 | -4.80869 | -0.40335 |
| C | 7.195176 | 8.183255 | 2.141364 | C | -11.1233 | -4.73034 | 0.382203 |
| C | 5.216687 | 9.930116 | 2.288524 | C | -11.7688 | -3.44368 | 2.599279 |
| C | 2.772954 | 9.215973 | 1.598982 | C | -9.89905 | -2.23734 | 4.015882 |
| C | 2.296758 | 6.770838 | 0.741519 | C | -7.38471 | -2.31197 | 3.232826 |
| H | 0.523307 | 3.29082 | -2.1799 | H | -2.28911 | -2.72397 | 3.475584 |
| H | 1.580563 | -5.42974 | 0.869973 | H | 0.446584 | 0.554752 | -4.7708 |
| H | 3.488837 | -2.7186 | 1.109328 | H | -2.01544 | -1.453 | -3.81578 |
| H | 0.089509 | -4.25992 | -3.23313 | H | 3.284461 | -1.03937 | -1.72879 |
| H | -4.93072 | -5.35999 | 3.913241 | H | 0.555366 | 6.407545 | -4.06292 |
| H | -2.92888 | -4.95105 | 0.015157 | H | 2.445032 | 3.342727 | -1.48687 |
| H | -4.23955 | -1.90842 | -0.32643 | H | 0.70174 | 4.287864 | 1.178647 |
| H | -1.28406 | -1.77653 | -6.4019 | H | 1.444966 | -1.06378 | 4.504854 |
| H | 2.98414 | 2.364514 | -6.10092 | H | 0.562481 | -6.31912 | 3.264058 |
| H | 5.285838 | 0.284417 | -4.83524 | H | 0.079204 | -7.05266 | 0.031167 |
| H | 3.205512 | -0.75855 | -7.24528 | H | 3.165484 | -6.37505 | 1.178537 |
| H | -3.2311 | 2.299481 | -2.77965 | H | 5.909947 | -1.85714 | 0.932805 |
| H | -5.71207 | -0.77338 | -7.10924 | H | 5.313313 | 0.597756 | 6.238802 |
| H | -10.0395 | -0.26249 | -7.7088 | H | 11.63256 | 0.557685 | 7.579315 |
| H | -11.7036 | 0.483743 | -4.88824 | H | 11.09009 | 3.502505 | 6.137861 |
| H | -11.0823 | 2.87666 | -7.1075 | H | 8.80112 | 2.237681 | 8.245826 |
| H | -9.72818 | 4.789184 | -2.93247 | H | 9.701569 | -1.23487 | 0.881383 |
| H | -8.03556 | 2.704293 | -0.92907 | H | 11.56393 | 1.43575 | 1.65735 |
| H | -6.39685 | 4.956854 | -2.75508 | H | 12.1676 | -1.48298 | 3.127036 |
| H | 4.04627 | -4.53875 | -5.71765 | H | 2.216148 | -3.37442 | -5.73749 |
| H | 3.479849 | -6.79447 | -3.38184 | H | 0.69719 | -5.65886 | -3.8957 |
| H | 7.528671 | -2.63669 | -3.24243 | H | 4.685612 | -7.44504 | -2.67695 |
| H | 11.81997 | -6.4757 | -0.38881 | H | 9.021373 | -7.80736 | -2.49141 |
| H | 11.05807 | -3.23897 | -0.86842 | H | 10.56969 | -6.16405 | -4.9795 |
| H | 10.4352 | -4.72437 | 2.075008 | H | 10.59411 | -4.90342 | -1.90433 |
| H | 5.003078 | -8.74483 | -0.46779 | H | 8.73188 | -1.92233 | -6.48449 |
| H | 8.168016 | -9.70302 | 0.067216 | H | 5.655193 | -0.99701 | -5.53725 |
| H | 6.579376 | -7.87895 | 2.348491 | H | 8.252719 | -0.62167 | -3.4652 |
| H | -7.92826 | -3.60109 | 7.409609 | H | -2.31765 | 10.22867 | 2.498369 |
| H | -6.19253 | -0.75578 | 7.775957 | H | -4.42063 | 8.335994 | 0.712105 |
| H | -9.41225 | -0.64362 | 6.830141 | H | -2.08223 | 6.882787 | 2.61523 |
| H | -4.83227 | 1.33903 | 3.356586 | H | 2.007382 | 10.50615 | 0.939519 |
| H | -9.46362 | -4.82992 | 2.883185 | H | -1.37606 | 12.57885 | -1.60515 |
| H | -10.6551 | -1.73411 | 2.374732 | H | 0.075838 | 10.93959 | -4.15204 |
| H | -8.41771 | -3.02096 | 0.245324 | H | -3.18593 | 10.47378 | -3.47963 |
| H | 8.231785 | 4.357495 | 1.187406 | H | -8.0834 | -5.79407 | -2.1181 |
| H | 9.097421 | 8.733318 | 2.678757 | H | -12.5749 | -5.66597 | -0.72568 |
| H | 5.581232 | 11.84123 | 2.941756 | H | -13.7248 | -3.38177 | 3.217493 |
| H | 1.231542 | 10.56379 | 1.726023 | H | -10.3962 | -1.23527 | 5.735693 |
| H | 0.379693 | 6.241104 | 0.263668 | H | -5.96863 | -1.33659 | 4.341075 |
| **2C-15** | | | | **2C-16** | | | |
| Atom | X | Y | Z | Atom | X | Y | Z |
| C | 0.800805 | 0.224802 | -0.89172 | C | 0.801185 | 0.224258 | -0.89178 |
| C | 3.266887 | -0.79315 | -1.91032 | C | 3.267386 | -0.79316 | -1.91071 |
| C | 4.406541 | -2.9032 | -0.26745 | C | 4.408107 | -2.9021 | -0.26718 |
| C | 2.411568 | -5.00808 | 0.030553 | C | 2.413942 | -5.00791 | 0.030428 |
| C | -0.16778 | -4.04774 | 0.96153 | C | -0.16577 | -4.04861 | 0.961513 |
| C | -1.27074 | -1.9559 | -0.81488 | C | -1.26965 | -1.95717 | -0.81482 |
| C | 6.779818 | -3.86427 | -1.59716 | C | 6.782161 | -3.86236 | -1.59603 |
| O | 8.864227 | -2.84914 | -0.52726 | O | 8.865888 | -2.84671 | -0.52524 |
| C | 8.279062 | -1.40368 | 1.746158 | C | 8.279153 | -1.40023 | 1.747184 |
| C | 5.460454 | -1.9099 | 2.268592 | C | 5.460912 | -1.90845 | 2.269174 |
| C | -0.15611 | 2.578306 | -2.26098 | C | -0.15671 | 2.577377 | -2.26103 |
| C | -0.41352 | 4.935135 | -0.74047 | C | -0.4161 | 4.933897 | -0.74033 |
| O | -0.78853 | 2.528461 | -4.48337 | O | -0.78849 | 2.527358 | -4.48361 |
| C | -3.55942 | -0.72976 | 0.372459 | C | -3.55861 | -0.73161 | 0.372629 |
| C | -1.80457 | -2.97477 | -3.4776 | C | -1.80346 | -2.97625 | -3.47745 |
| C | -5.67375 | 0.015704 | -0.81871 | C | -5.6727 | 0.014324 | -0.8187 |
| C | -7.72268 | 1.309701 | 0.470141 | C | -7.72158 | 1.308467 | 0.470094 |
| C | -9.93136 | 2.097071 | -0.53677 | C | -9.92954 | 2.097388 | -0.53718 |
| C | -11.8683 | 3.422899 | 1.0721 | C | -11.8665 | 3.423099 | 1.071775 |
| C | -10.6765 | 1.750438 | -3.26069 | C | -10.6741 | 1.752453 | -3.26147 |
| C | -1.9593 | -6.32374 | 1.38498 | C | -1.95642 | -6.32523 | 1.385209 |
| C | -4.1959 | -5.72194 | 3.02883 | C | -4.19336 | -5.72382 | 3.028755 |
| C | -6.65208 | -5.70744 | 2.410249 | C | -6.64948 | -5.71013 | 2.409928 |
| C | -8.6255 | -4.90114 | 4.301058 | C | -8.6233 | -4.90379 | 4.3003 |
| C | -7.67734 | -6.29723 | -0.1752 | C | -7.67428 | -6.30083 | -0.1755 |
| C | 8.975916 | 1.406028 | 1.388204 | C | 8.973593 | 1.409919 | 1.387832 |
| C | 11.58095 | 1.680639 | 0.159848 | C | 11.57814 | 1.686295 | 0.158943 |
| O | 7.100614 | 2.66228 | -0.11908 | O | 7.096874 | 2.663845 | -0.11964 |
| C | 8.90754 | 2.724823 | 3.958925 | C | 8.904382 | 2.72973 | 3.958028 |
| O | 4.243595 | -0.09081 | -3.87453 | O | 4.242911 | -0.09179 | -3.87585 |
| O | 6.897946 | -5.30527 | -3.36255 | O | 6.901465 | -5.30331 | -3.36142 |
| C | -2.45268 | 6.548812 | -1.25488 | C | 1.334281 | 5.585309 | 1.140765 |
| C | -2.77012 | 8.75974 | 0.1335 | C | 1.039245 | 7.831078 | 2.487855 |
| C | -1.01818 | 9.405743 | 2.004202 | C | -1.02472 | 9.403704 | 2.004809 |
| C | 1.045024 | 7.831934 | 2.486589 | C | -2.77541 | 8.756947 | 0.133205 |
| C | 1.338062 | 5.585753 | 1.139758 | C | -2.45599 | 6.546435 | -1.2554 |
| H | 1.120162 | 0.711711 | 1.088446 | H | 1.120481 | 0.711251 | 1.088341 |
| H | 3.145912 | -6.40064 | 1.374602 | H | 3.148824 | -6.40036 | 1.374301 |
| H | 2.216857 | -5.98805 | -1.78071 | H | 2.219695 | -5.9877 | -1.78098 |
| H | 0.125009 | -3.15654 | 2.816512 | H | 0.126806 | -3.15727 | 2.816461 |
| H | 9.482574 | -2.19959 | 3.22203 | H | 9.483105 | -2.19422 | 3.223716 |
| H | 5.251222 | -3.37725 | 3.707595 | H | 5.252569 | -3.3763 | 3.707805 |
| H | 4.50935 | -0.21889 | 2.953334 | H | 4.508526 | -0.2183 | 2.954224 |
| H | -3.3627 | -0.30508 | 2.378865 | H | -3.36215 | -0.30739 | 2.379158 |
| H | -3.35151 | -4.34015 | -3.42857 | H | -3.35026 | -4.34179 | -3.42824 |
| H | -2.32046 | -1.44677 | -4.75686 | H | -2.31957 | -1.44843 | -4.75683 |
| H | -0.1462 | -3.90767 | -4.28337 | H | -0.14503 | -3.909 | -4.28325 |
| H | -5.88488 | -0.36433 | -2.82318 | H | -5.88352 | -0.36521 | -2.8233 |
| H | -7.40679 | 1.668643 | 2.474702 | H | -7.40616 | 1.666381 | 2.474916 |
| H | -12.2846 | 5.315543 | 0.324763 | H | -13.6671 | 2.387839 | 1.069814 |
| H | -13.6688 | 2.387495 | 1.070558 | H | -11.2368 | 3.623835 | 3.031196 |
| H | -11.2383 | 3.62419 | 3.031383 | H | -12.2826 | 5.315928 | 0.324816 |
| H | -11.0563 | 3.588478 | -4.14785 | H | -11.0535 | 3.591079 | -4.14758 |
| H | -9.2457 | 0.782883 | -4.38897 | H | -9.24316 | 0.785427 | -4.39006 |
| H | -12.4396 | 0.662498 | -3.39457 | H | -12.4373 | 0.664859 | -3.39641 |
| H | -0.8359 | -7.80115 | 2.318091 | H | -0.83254 | -7.80207 | 2.318658 |
| H | -2.53098 | -7.11088 | -0.43744 | H | -2.52771 | -7.11292 | -0.4371 |
| H | -3.70172 | -5.18014 | 4.956967 | H | -3.6995 | -5.18136 | 4.956795 |
| H | -7.79834 | -4.47709 | 6.14888 | H | -7.79642 | -4.47881 | 6.148037 |
| H | -9.60762 | -3.20001 | 3.626923 | H | -9.60589 | -3.2032 | 3.625477 |
| H | -10.0763 | -6.36441 | 4.55431 | H | -10.0736 | -6.36742 | 4.553972 |
| H | -8.39407 | -4.55719 | -1.05421 | H | -6.2695 | -7.11796 | -1.44751 |
| H | -6.27265 | -7.11346 | -1.44788 | H | -9.27269 | -7.61706 | -0.05386 |
| H | -9.27541 | -7.61388 | -0.05375 | H | -8.39041 | -4.561 | -1.05541 |
| H | 13.03986 | 0.74866 | 1.293604 | H | 12.05177 | 3.691463 | -0.00976 |
| H | 11.59785 | 0.831808 | -1.72519 | H | 13.03802 | 0.755953 | 1.292798 |
| H | 12.05627 | 3.685472 | -0.00803 | H | 11.59549 | 0.83674 | -1.72577 |
| H | 7.208079 | 2.03457 | -1.84247 | H | 7.206514 | 2.037694 | -1.84345 |
| H | 9.311601 | 4.735257 | 3.701769 | H | 10.32199 | 1.931361 | 5.233383 |
| H | 7.048954 | 2.552546 | 4.849735 | H | 9.306744 | 4.740391 | 3.699999 |
| H | 10.32442 | 1.924775 | 5.234037 | H | 7.046001 | 2.556272 | 4.849054 |
| H | -3.79235 | 6.018483 | -2.71343 | H | 2.977012 | 4.402769 | 1.475152 |
| H | -4.37581 | 9.98221 | -0.23873 | H | 2.418375 | 8.350687 | 3.915781 |
| H | -1.2602 | 11.13693 | 3.079827 | H | -1.26828 | 11.13456 | 3.080624 |
| H | 2.425144 | 8.350911 | 3.913785 | H | -4.3817 | 9.978478 | -0.23955 |
| H | 2.980249 | 4.402304 | 1.473608 | H | -3.79473 | 6.015514 | -2.71458 |
| **2C-17** | | | | **2C-18** | | | |
| Atom | X | Y | Z | Atom | X | Y | Z |
| C | 2.191258 | 0.409967 | -2.86969 | C | 2.739351 | -1.25438 | -2.23695 |
| C | 0.456393 | 1.79486 | -1.02616 | C | 1.704764 | 1.157503 | -1.03805 |
| C | -0.43284 | 0.276599 | 1.283695 | C | 0.212369 | 0.829231 | 1.431651 |
| C | 0.636756 | -2.4293 | 1.268318 | C | 0.092738 | -1.96127 | 2.243099 |
| C | 0.34362 | -3.86165 | -1.25315 | C | -0.75636 | -3.78241 | 0.137453 |
| C | 0.826183 | -2.12163 | -3.61218 | C | 0.469536 | -3.15001 | -2.49244 |
| C | 0.666032 | 1.670119 | 3.581214 | C | 1.696309 | 2.30882 | 3.43771 |
| O | -1.17021 | 2.377504 | 5.187765 | O | 0.238277 | 4.077876 | 4.533975 |
| C | -3.67033 | 1.597232 | 4.333679 | C | -2.33526 | 4.073511 | 3.55086 |
| C | -3.31369 | 0.47389 | 1.670796 | C | -2.36166 | 2.190943 | 1.329834 |
| C | 4.931758 | 0.23467 | -1.9079 | C | 5.140469 | -2.2241 | -0.9094 |
| C | 6.42296 | 2.61712 | -1.70151 | C | 7.495642 | -0.6925 | -1.1327 |
| O | 5.891803 | -1.79567 | -1.35346 | O | 5.143819 | -4.21195 | 0.273275 |
| C | -1.63654 | -1.18577 | -4.79154 | C | -1.35888 | -1.73039 | -4.21865 |
| C | 2.333642 | -3.40881 | -5.75278 | C | 1.362145 | -5.49364 | -3.98456 |
| C | -4.04483 | -1.61385 | -4.11016 | C | -3.76482 | -1.02033 | -3.82425 |
| C | -6.18975 | -0.41489 | -5.33807 | C | -5.2077 | 0.48072 | -5.61568 |
| C | -8.63267 | -0.54699 | -4.61237 | C | -7.52622 | 1.479493 | -5.2427 |
| C | -10.6474 | 0.865723 | -6.04188 | C | -8.76599 | 3.059032 | -7.25911 |
| C | -9.58292 | -2.02702 | -2.37469 | C | -9.06077 | 1.174402 | -2.86465 |
| C | 1.967037 | -6.29719 | -1.15545 | C | -0.43575 | -6.53988 | 1.063691 |
| C | 1.249188 | -7.97885 | 1.021248 | C | -1.93836 | -7.04524 | 3.436811 |
| C | 2.565356 | -8.41271 | 3.144569 | C | -4.42588 | -7.52183 | 3.561019 |
| C | 1.556341 | -10.1555 | 5.163393 | C | -5.73233 | -7.92666 | 6.061028 |
| C | 5.091349 | -7.23986 | 3.751773 | C | -6.13037 | -7.67538 | 1.281119 |
| C | -5.4587 | 3.876063 | 4.564881 | C | -3.07994 | 6.816248 | 2.945429 |
| C | -8.1225 | 3.07432 | 3.791087 | C | -5.81532 | 6.874032 | 2.022118 |
| O | -5.62027 | 4.53584 | 7.193741 | O | -3.05585 | 8.213224 | 5.273366 |
| C | -4.54616 | 6.164725 | 3.043485 | C | -1.28296 | 8.061874 | 1.046757 |
| O | -0.23781 | 3.961896 | -1.35216 | O | 1.982464 | 3.237258 | -1.97752 |
| O | 2.878346 | 2.092755 | 3.973153 | O | 3.879005 | 1.969799 | 4.026487 |
| C | 8.845065 | 2.450083 | -0.62244 | C | 7.680537 | 1.499723 | -2.6172 |
| C | 10.34523 | 4.590517 | -0.3686 | C | 9.951659 | 2.829908 | -2.73284 |
| C | 9.447359 | 6.934399 | -1.1978 | C | 12.05375 | 1.991293 | -1.37796 |
| C | 7.045927 | 7.120932 | -2.27298 | C | 11.88522 | -0.18975 | 0.105972 |
| C | 5.534985 | 4.977743 | -2.52747 | C | 9.624434 | -1.52074 | 0.224224 |
| H | 2.256652 | 1.600367 | -4.55917 | H | 3.320859 | -0.71233 | -4.14551 |
| H | -0.26901 | -3.51106 | 2.777073 | H | -1.20135 | -2.13246 | 3.84653 |
| H | 2.640709 | -2.32928 | 1.747585 | H | 1.9578 | -2.52524 | 2.92184 |
| H | -1.61041 | -4.51991 | -1.37491 | H | -2.79324 | -3.54661 | -0.07382 |
| H | -4.29853 | 0.16475 | 5.682769 | H | -3.52676 | 3.425624 | 5.108727 |
| H | -4.21048 | -1.37665 | 1.552879 | H | -3.91929 | 0.859206 | 1.539159 |
| H | -4.16294 | 1.677153 | 0.232448 | H | -2.60446 | 3.161459 | -0.46947 |
| H | -1.32906 | 0.091395 | -6.38318 | H | -0.50285 | -1.17495 | -6.0126 |
| H | 2.577252 | -2.08576 | -7.32701 | H | 2.203462 | -4.89899 | -5.78101 |
| H | 4.199591 | -4.01572 | -5.12345 | H | 2.765803 | -6.58153 | -2.93961 |
| H | 1.302998 | -5.04974 | -6.47315 | H | -0.2437 | -6.71817 | -4.42607 |
| H | -4.46168 | -2.85876 | -2.53832 | H | -4.71075 | -1.51809 | -2.07726 |
| H | -5.73806 | 0.751584 | -6.97519 | H | -4.27205 | 0.869503 | -7.40959 |
| H | -9.8664 | 1.926045 | -7.63575 | H | -7.5751 | 3.244078 | -8.93905 |
| H | -12.0911 | -0.43741 | -6.76961 | H | -10.5822 | 2.232836 | -7.83446 |
| H | -11.6397 | 2.198328 | -4.79617 | H | -9.19104 | 4.963507 | -6.54867 |
| H | -8.11197 | -3.09382 | -1.39808 | H | -10.9247 | 0.374825 | -3.30372 |
| H | -10.4676 | -0.75416 | -0.99368 | H | -8.16997 | -0.03184 | -1.44766 |
| H | -11.0565 | -3.36386 | -2.9644 | H | -9.40994 | 3.025909 | -1.99254 |
| H | 3.956969 | -5.77522 | -1.08175 | H | 1.565706 | -6.91271 | 1.406346 |
| H | 1.674717 | -7.3532 | -2.91346 | H | -1.04337 | -7.82547 | -0.43643 |
| H | -0.58334 | -8.90874 | 0.833222 | H | -0.89792 | -6.94107 | 5.211082 |
| H | 2.86752 | -11.7269 | 5.515854 | H | -6.63351 | -9.79602 | 6.138001 |
| H | 1.346039 | -9.15466 | 6.970844 | H | -4.41693 | -7.77568 | 7.650054 |
| H | -0.28192 | -10.9514 | 4.647836 | H | -7.24862 | -6.53551 | 6.341301 |
| H | 5.793764 | -5.99322 | 2.265848 | H | -7.54472 | -6.15548 | 1.326758 |
| H | 4.961714 | -6.1282 | 5.500766 | H | -5.10889 | -7.52915 | -0.50709 |
| H | 6.517805 | -8.70813 | 4.097833 | H | -7.18959 | -9.46023 | 1.275826 |
| H | -8.73512 | 1.420798 | 4.874161 | H | -6.4209 | 8.83837 | 1.802559 |
| H | -9.43841 | 4.623617 | 4.167383 | H | -6.01159 | 5.925686 | 0.195946 |
| H | -8.21379 | 2.619391 | 1.776821 | H | -7.0696 | 5.953384 | 3.386484 |
| H | -3.92429 | 5.000676 | 7.724584 | H | -1.33389 | 8.145651 | 5.909453 |
| H | -2.68879 | 6.787847 | 3.707741 | H | -1.90373 | 10.00389 | 0.706372 |
| H | -4.38023 | 5.750516 | 1.027181 | H | 0.645585 | 8.111706 | 1.793335 |
| H | -5.88647 | 7.718258 | 3.29483 | H | -1.22765 | 7.057441 | -0.7569 |
| H | 9.508081 | 0.61825 | 0.012124 | H | 6.064311 | 2.200053 | -3.6576 |
| H | 12.21035 | 4.441183 | 0.473545 | H | 10.07737 | 4.523294 | -3.88425 |
| H | 10.61761 | 8.609169 | -1.00334 | H | 13.81944 | 3.033001 | -1.47473 |
| H | 6.344364 | 8.938337 | -2.91644 | H | 13.51629 | -0.8434 | 1.165472 |
| H | 3.66594 | 5.169966 | -3.33655 | H | 9.457902 | -3.2136 | 1.366361 |
| **2C-19** | | | |  |  |  |  |
| Atom | X | Y | Z |  |  |  |  |
| C | -0.49715 | -0.75482 | 0.975702 |  |  |  |  |
| C | 2.063403 | -1.16571 | 2.182411 |  |  |  |  |
| C | 4.217597 | 0.249652 | 0.835618 |  |  |  |  |
| C | 3.521728 | 3.07789 | 0.748589 |  |  |  |  |
| C | 0.903831 | 3.577153 | -0.39041 |  |  |  |  |
| C | -1.23951 | 2.15305 | 1.050408 |  |  |  |  |
| C | 6.642933 | -0.19873 | 2.33179 |  |  |  |  |
| O | 8.068554 | -1.98525 | 1.192491 |  |  |  |  |
| C | 7.043512 | -2.74471 | -1.25114 |  |  |  |  |
| C | 4.863446 | -0.88788 | -1.77497 |  |  |  |  |
| C | -2.58329 | -2.43886 | 2.043932 |  |  |  |  |
| C | -3.77799 | -4.28447 | 0.284253 |  |  |  |  |
| O | -3.33094 | -2.22335 | 4.220522 |  |  |  |  |
| C | -3.669 | 2.334834 | -0.44182 |  |  |  |  |
| C | -1.51262 | 3.115403 | 3.772137 |  |  |  |  |
| C | -6.00847 | 2.67571 | 0.487107 |  |  |  |  |
| C | -8.26353 | 2.614663 | -1.07662 |  |  |  |  |
| C | -10.6844 | 2.815382 | -0.29559 |  |  |  |  |
| C | -12.8258 | 2.67195 | -2.16428 |  |  |  |  |
| C | -11.4873 | 3.172484 | 2.4104 |  |  |  |  |
| C | 0.408935 | 6.450574 | -0.57362 |  |  |  |  |
| C | 2.047739 | 7.687028 | -2.5379 |  |  |  |  |
| C | 4.065039 | 9.180378 | -2.1759 |  |  |  |  |
| C | 5.52661 | 10.22372 | -4.39079 |  |  |  |  |
| C | 5.075763 | 9.952373 | 0.373964 |  |  |  |  |
| C | 6.278294 | -5.55864 | -1.20866 |  |  |  |  |
| C | 8.341724 | -7.17576 | 0.015811 |  |  |  |  |
| O | 3.934578 | -5.87878 | 0.12221 |  |  |  |  |
| C | 5.756257 | -6.43526 | -3.91237 |  |  |  |  |
| O | 2.407116 | -2.42986 | 4.076521 |  |  |  |  |
| O | 7.292975 | 0.82314 | 4.265785 |  |  |  |  |
| C | -2.38026 | -5.54788 | -1.58075 |  |  |  |  |
| C | -3.57045 | -7.29072 | -3.1587 |  |  |  |  |
| C | -6.16051 | -7.73975 | -2.92077 |  |  |  |  |
| C | -7.56 | -6.48081 | -1.06528 |  |  |  |  |
| C | -6.36805 | -4.7829 | 0.551124 |  |  |  |  |
| H | -0.28192 | -1.18469 | -1.03212 |  |  |  |  |
| H | 4.951009 | 4.073547 | -0.36609 |  |  |  |  |
| H | 3.634718 | 3.838107 | 2.669212 |  |  |  |  |
| H | 0.901125 | 2.835109 | -2.33247 |  |  |  |  |
| H | 8.592568 | -2.5004 | -2.59333 |  |  |  |  |
| H | 5.49709 | 0.62525 | -3.03062 |  |  |  |  |
| H | 3.275937 | -1.83221 | -2.68149 |  |  |  |  |
| H | -3.45497 | 2.002068 | -2.46702 |  |  |  |  |
| H | 0.275316 | 2.98963 | 4.801138 |  |  |  |  |
| H | -2.11733 | 5.090901 | 3.772105 |  |  |  |  |
| H | -2.89204 | 2.002093 | 4.818203 |  |  |  |  |
| H | -6.24792 | 2.96892 | 2.503747 |  |  |  |  |
| H | -7.9289 | 2.343642 | -3.09191 |  |  |  |  |
| H | -13.9775 | 4.399018 | -2.10548 |  |  |  |  |
| H | -12.1478 | 2.408961 | -4.0998 |  |  |  |  |
| H | -14.1055 | 1.102627 | -1.70341 |  |  |  |  |
| H | -12.7163 | 1.610175 | 3.008429 |  |  |  |  |
| H | -9.90834 | 3.281128 | 3.733524 |  |  |  |  |
| H | -12.6102 | 4.906921 | 2.607782 |  |  |  |  |
| H | 0.680711 | 7.314697 | 1.284318 |  |  |  |  |
| H | -1.58544 | 6.730505 | -1.07112 |  |  |  |  |
| H | 1.533403 | 7.23982 | -4.48441 |  |  |  |  |
| H | 4.73593 | 9.603343 | -6.19836 |  |  |  |  |
| H | 5.535647 | 12.29994 | -4.3643 |  |  |  |  |
| H | 7.514566 | 9.629398 | -4.31318 |  |  |  |  |
| H | 5.137181 | 12.02033 | 0.539693 |  |  |  |  |
| H | 3.968228 | 9.215822 | 1.951299 |  |  |  |  |
| H | 7.028343 | 9.286987 | 0.605937 |  |  |  |  |
| H | 7.782828 | -9.16521 | -0.04009 |  |  |  |  |
| H | 10.14158 | -6.96711 | -0.98408 |  |  |  |  |
| H | 8.637632 | -6.62248 | 1.985547 |  |  |  |  |
| H | 4.212647 | -5.52626 | 1.903948 |  |  |  |  |
| H | 7.462438 | -6.29971 | -5.07159 |  |  |  |  |
| H | 5.135881 | -8.40643 | -3.87994 |  |  |  |  |
| H | 4.266877 | -5.30841 | -4.80049 |  |  |  |  |
| H | -0.35504 | -5.25646 | -1.72739 |  |  |  |  |
| H | -2.47649 | -8.29502 | -4.57485 |  |  |  |  |
| H | -7.08882 | -9.07304 | -4.17495 |  |  |  |  |
| H | -9.57411 | -6.82872 | -0.88272 |  |  |  |  |
| H | -7.42242 | -3.79204 | 2.003879 |  |  |  |  |

**Table S11.** Gibbs free energies^a^ and equilibrium populations^b^ of low-energy conformers of **2D**.

| Conformers | ∆G(a.u.) | P(%)/100 | G(a.u.) |
| --- | --- | --- | --- |
| **2D-1** | 0.0 | 23.43 | -1580.910333 |
| **2D-2** | 1e-05 | 23.27 | -1580.910327 |
| **2D-3** | 0.00135 | 5.58 | -1580.908979 |
| **2D-4** | 0.00224 | 2.19 | -1580.908095 |
| **2D-5** | 0.00254 | 1.59 | -1580.907792 |
| **2D-6** | 0.00098 | 8.28 | -1580.90935 |
| **2D-7** | 0.00246 | 1.73 | -1580.907871 |
| **2D-8** | 0.00098 | 8.28 | -1580.909351 |
| **2D-9** | 0.00098 | 8.28 | -1580.909351 |
| **2D-10** | 0.00305 | 0.93 | -1580.907287 |
| **2D-11** | 0.0047 | 0.16 | -1580.905636 |
| **2D-12** | 0.00389 | 0.38 | -1580.90644 |
| **2D-13** | 0.00178 | 3.54 | -1580.908549 |
| **2D-14** | 0.00163 | 4.18 | -1580.908706 |
| **2D-15** | 0.00453 | 0.19 | -1580.905799 |
| **2D-16** | 0.00401 | 0.34 | -1580.906322 |
| **2D-17** | 0.00171 | 3.82 | -1580.908621 |
| **2D-18** | 0.00171 | 3.83 | -1580.908623 |

^a^wB97M-V/def2-TZVP, in a.u.
^b^From ∆G values at 298.15K.

**Table S12.** Cartesian coordinates for the low-energy reoptimized random research conformers of **2D** at B3LYP-D3(BJ)/6-31G* level of theory in methanol.

| **2D-1** | | | | **2D-2** | | | |
| --- | --- | --- | --- | --- | --- | --- | --- |
| Atom | X | Y | Z | Atom | X | Y | Z |
| C | -2.11249 | -1.89059 | -1.45654 | C | 2.111722 | 1.890983 | -1.45742 |
| C | -0.20528 | -1.5446 | 0.692084 | C | 0.205013 | 1.544677 | 0.691587 |
| C | 0.655185 | 1.159538 | 1.268768 | C | -0.65459 | -1.15968 | 1.26869 |
| C | -1.06025 | 3.096388 | -0.01073 | C | 1.061499 | -3.09604 | -0.01063 |
| C | -1.30235 | 2.770282 | -2.89112 | C | 1.303051 | -2.77033 | -2.89113 |
| C | -1.55857 | -0.10433 | -3.72315 | C | 1.558177 | 0.104224 | -3.7237 |
| C | 0.57737 | 1.453849 | 4.157968 | C | -0.57675 | -1.45351 | 4.157959 |
| O | 2.899292 | 1.107221 | 5.153884 | O | -2.89882 | -1.10724 | 5.153757 |
| C | 4.754439 | 0.528893 | 3.185169 | C | -4.75404 | -0.52985 | 3.184865 |
| C | 3.51469 | 1.445684 | 0.745401 | C | -3.51401 | -1.44702 | 0.745418 |
| C | -4.81655 | -1.73715 | -0.3712 | C | 4.815965 | 1.738633 | -0.37226 |
| C | -5.88264 | -4.01117 | 0.905243 | C | 5.8811 | 4.012883 | 0.904616 |
| O | -6.07287 | 0.196343 | -0.55974 | O | 6.073145 | -0.19424 | -0.56121 |
| C | 0.958822 | -0.83115 | -4.90379 | C | -0.95963 | 0.829981 | -4.90411 |
| C | -3.57835 | -0.55932 | -5.78494 | C | 3.577509 | 0.55955 | -5.78587 |
| C | 2.588647 | -2.65501 | -4.21737 | C | -2.58985 | 2.653529 | -4.21781 |
| C | 5.042441 | -3.02348 | -5.40123 | C | -5.04413 | 3.020937 | -5.401 |
| C | 6.953516 | -4.4493 | -4.49391 | C | -6.95538 | 4.446306 | -4.49335 |
| C | 9.425009 | -4.6288 | -5.8927 | C | -9.42741 | 4.62468 | -5.89135 |
| C | 6.855157 | -5.91727 | -2.056 | C | -6.85672 | 5.914824 | -2.05577 |
| C | -3.40747 | 4.539763 | -3.88888 | C | 3.408609 | -4.5393 | -3.88891 |
| C | -2.89907 | 7.269276 | -3.27837 | C | 2.90152 | -7.26879 | -3.27721 |
| C | -4.06662 | 8.717301 | -1.55535 | C | 4.070243 | -8.71574 | -1.55408 |
| C | -3.29965 | 11.43569 | -1.16428 | C | 3.304415 | -11.4343 | -1.16171 |
| C | -6.17554 | 7.828601 | 0.143434 | C | 6.179488 | -7.82564 | 0.143564 |
| C | 7.248806 | 1.837694 | 3.890612 | C | -7.24815 | -1.839 | 3.890602 |
| C | 8.240064 | 0.827829 | 6.418706 | C | -8.23961 | -0.82865 | 6.418428 |
| O | 6.850507 | 4.512303 | 4.048162 | O | -6.84924 | -4.51347 | 4.048838 |
| C | 9.178128 | 1.424226 | 1.781862 | C | -9.17756 | -1.4265 | 1.78174 |
| O | 0.609433 | -3.33406 | 1.897113 | O | -0.60986 | 3.334051 | 1.896658 |
| O | -1.24925 | 1.928942 | 5.442702 | O | 1.249895 | -1.92796 | 5.442855 |
| C | -8.38985 | -3.85266 | 1.774356 | C | 4.525614 | 6.264932 | 1.274567 |
| C | -9.52079 | -5.8963 | 2.973981 | C | 5.660884 | 8.307608 | 2.49078 |
| C | -8.15707 | -8.13202 | 3.334254 | C | 8.154004 | 8.134211 | 3.334272 |
| C | -5.6635 | -8.30566 | 2.492175 | C | 9.518023 | 5.898504 | 2.975068 |
| C | -4.52747 | -6.26322 | 1.276238 | C | 8.387857 | 3.854656 | 1.775069 |
| H | -1.86327 | -3.82945 | -2.11559 | H | 1.861777 | 3.829653 | -2.11674 |
| H | -0.34081 | 4.99067 | 0.392483 | H | 0.342887 | -4.99055 | 0.392986 |
| H | -2.92794 | 2.979136 | 0.858499 | H | 2.929259 | -2.9779 | 0.85834 |
| H | 0.472834 | 3.449153 | -3.72187 | H | -0.47203 | -3.44997 | -3.72148 |
| H | 5.001062 | -1.52095 | 3.186638 | H | -5.0011 | 1.519941 | 3.185744 |
| H | 4.161579 | 0.399346 | -0.90216 | H | -4.16128 | -0.40158 | -0.90255 |
| H | 3.936426 | 3.451753 | 0.474334 | H | -3.93495 | -3.45337 | 0.475145 |
| H | 1.477679 | 0.375662 | -6.49378 | H | -1.47844 | -0.37745 | -6.49364 |
| H | -3.66496 | -2.58075 | -6.22402 | H | 3.076988 | -0.43269 | -7.52898 |
| H | -5.4518 | 0.064791 | -5.19446 | H | 3.663529 | 2.580979 | -6.2251 |
| H | -3.07792 | 0.432696 | -7.5282 | H | 5.451219 | -0.06405 | -5.1957 |
| H | 2.142736 | -3.89141 | -2.64658 | H | -2.14382 | 3.890604 | -2.64759 |
| H | 5.362909 | -1.97666 | -7.14665 | H | -5.36482 | 1.973632 | -7.1461 |
| H | 9.86225 | -6.60129 | -6.37316 | H | -9.86559 | 6.596955 | -6.37181 |
| H | 9.406807 | -3.52464 | -7.64075 | H | -9.40931 | 3.520407 | -7.63932 |
| H | 10.99448 | -3.95489 | -4.7116 | H | -10.9962 | 3.95021 | -4.70971 |
| H | 5.07441 | -5.71606 | -1.03338 | H | -8.38041 | 5.290645 | -0.79057 |
| H | 7.178064 | -7.93788 | -2.40378 | H | -5.07578 | 5.714039 | -1.03343 |
| H | 8.379152 | -5.29302 | -0.79121 | H | -7.17998 | 7.93533 | -2.40388 |
| H | -5.21566 | 3.919379 | -3.11922 | H | 5.216767 | -3.91786 | -3.12003 |
| H | -3.52016 | 4.34664 | -5.94824 | H | 3.520598 | -4.34688 | -5.94837 |
| H | -1.36171 | 8.126469 | -4.35481 | H | 1.364086 | -8.12695 | -4.35279 |
| H | -4.90002 | 12.71742 | -1.49402 | H | 1.760904 | -11.9908 | -2.42112 |
| H | -2.68654 | 11.75936 | 0.793299 | H | 4.905111 | -12.7155 | -1.49159 |
| H | -1.75643 | 11.99117 | -2.4245 | H | 2.692178 | -11.7574 | 0.796227 |
| H | -6.72625 | 5.86927 | -0.19331 | H | 7.860303 | -9.02249 | -0.0851 |
| H | -5.60752 | 7.99127 | 2.13402 | H | 6.729451 | -5.86628 | -0.19421 |
| H | -7.85605 | 9.025906 | -0.08508 | H | 5.612279 | -7.98768 | 2.134432 |
| H | 8.622038 | -1.20356 | 6.308323 | H | -6.85949 | -1.14109 | 7.927354 |
| H | 6.860046 | 1.141083 | 7.927558 | H | -9.99389 | -1.80377 | 6.914759 |
| H | 9.994601 | 1.802642 | 6.914716 | H | -8.62212 | 1.202607 | 6.30742 |
| H | 5.596874 | 4.781121 | 5.364502 | H | -5.59571 | -4.78165 | 5.365401 |
| H | 9.500432 | -0.5953 | 1.469402 | H | -10.9755 | -2.30215 | 2.306235 |
| H | 8.535387 | 2.272178 | 0.009917 | H | -9.50031 | 0.592868 | 1.468756 |
| H | 10.9763 | 2.299606 | 2.306116 | H | -8.53462 | -2.27477 | 0.010017 |
| H | -9.41642 | -2.10362 | 1.483594 | H | 2.575803 | 6.420921 | 0.678111 |
| H | -11.4596 | -5.755 | 3.630285 | H | 4.594877 | 10.0369 | 2.77723 |
| H | -9.03762 | -9.73176 | 4.271048 | H | 9.033955 | 9.734121 | 4.271325 |
| H | -4.5977 | -10.0349 | 2.779509 | H | 11.45651 | 5.757385 | 3.63249 |
| H | -2.57732 | -6.41936 | 0.680878 | H | 9.41471 | 2.105661 | 1.485039 |
| **2D-3** | | | | **2D-4** | | | |
| Atom | X | Y | Z | Atom | X | Y | Z |
| C | 2.315238 | 0.314543 | -3.0029 | C | -1.98967 | -2.03552 | -1.40781 |
| C | 0.677423 | 1.898022 | -1.22967 | C | -0.10177 | -1.52881 | 0.724092 |
| C | -0.35317 | 0.528188 | 1.112272 | C | 0.615121 | 1.234307 | 1.208382 |
| C | 0.445715 | -2.25766 | 1.174905 | C | -1.12393 | 3.043489 | -0.21955 |
| C | 0.095611 | -3.73902 | -1.30906 | C | -1.26825 | 2.558806 | -3.08202 |
| C | 0.770783 | -2.12171 | -3.70798 | C | -1.43164 | -0.36118 | -3.75872 |
| C | 0.823133 | 1.917014 | 3.384176 | C | 0.377972 | 1.646945 | 4.074898 |
| O | -0.8596 | 3.50973 | 4.434732 | O | 2.661027 | 1.518618 | 5.191837 |
| C | -3.27595 | 3.475375 | 3.090742 | C | 4.64798 | 1.018317 | 3.357059 |
| C | -3.19063 | 1.086901 | 1.459946 | C | 3.479899 | 1.619115 | 0.77779 |
| C | 5.004815 | -0.05866 | -1.95411 | C | -4.70822 | -1.87875 | -0.36163 |
| C | 6.644396 | 2.215101 | -1.66349 | C | -5.69692 | -4.05378 | 1.131921 |
| O | 5.800558 | -2.15186 | -1.37556 | O | -6.03723 | -0.02518 | -0.74939 |
| C | -1.58419 | -1.05684 | -4.99186 | C | 1.123639 | -1.09378 | -4.85215 |
| C | 2.226114 | -3.59115 | -5.76792 | C | -3.39365 | -0.9822 | -5.83458 |
| C | -4.03401 | -1.31069 | -4.37394 | C | 2.727039 | -2.92364 | -4.11763 |
| C | -6.06692 | -0.05188 | -5.72739 | C | 5.162031 | -3.36888 | -5.3112 |
| C | -8.53018 | 0.010246 | -5.06355 | C | 6.985951 | -4.95174 | -4.48873 |
| C | -10.4193 | 1.443224 | -6.63711 | C | 9.42952 | -5.22233 | -5.92172 |
| C | -9.62212 | -1.25748 | -2.76086 | C | 6.804239 | -6.53921 | -2.13236 |
| C | 1.510874 | -6.29456 | -1.10264 | C | -3.38202 | 4.218834 | -4.23685 |
| C | 0.618088 | -7.84103 | 1.109284 | C | -2.95067 | 6.990036 | -3.77151 |
| C | 1.849585 | -8.29962 | 3.277681 | C | -4.19207 | 8.505321 | -2.16163 |
| C | 0.66339 | -9.88513 | 5.329583 | C | -3.49642 | 11.25908 | -1.90944 |
| C | 4.444352 | -7.30204 | 3.907888 | C | -6.32037 | 7.663675 | -0.46326 |
| C | -5.39783 | 3.542847 | 5.075175 | C | 7.008304 | 2.542069 | 4.099192 |
| C | -5.22782 | 5.961186 | 6.661339 | C | 6.489692 | 5.382263 | 4.300837 |
| O | -5.19937 | 1.364972 | 6.674284 | O | 8.709445 | 2.022625 | 2.048157 |
| C | -7.95885 | 3.348479 | 3.755065 | C | 8.093453 | 1.521912 | 6.582578 |
| O | 0.160757 | 4.102675 | -1.63796 | O | 0.816655 | -3.22888 | 1.981091 |
| O | 2.956167 | 1.688818 | 4.170136 | O | -1.54329 | 2.033049 | 5.248675 |
| C | 8.943061 | 1.903039 | -0.36783 | C | -8.18505 | -3.86732 | 2.048854 |
| C | 10.56811 | 3.936681 | -0.02208 | C | -9.24408 | -5.81996 | 3.448915 |
| C | 9.924152 | 6.31504 | -0.9793 | C | -7.82767 | -7.9924 | 3.960938 |
| C | 7.649453 | 6.644241 | -2.27363 | C | -5.35355 | -8.19375 | 3.069189 |
| C | 6.010777 | 4.609993 | -2.6158 | C | -4.2884 | -6.24182 | 1.65643 |
| H | 2.519972 | 1.448469 | -4.72065 | H | -1.6998 | -4.00314 | -1.95675 |
| H | -0.59585 | -3.20652 | 2.685274 | H | -0.47796 | 4.979411 | 0.104211 |
| H | 2.434683 | -2.32584 | 1.719169 | H | -3.01143 | 2.909317 | 0.602352 |
| H | -1.90209 | -4.23936 | -1.45745 | H | 0.515568 | 3.235576 | -3.8958 |
| H | -3.32273 | 5.181593 | 1.931185 | H | 5.084903 | -0.99549 | 3.491825 |
| H | -4.18084 | 1.362001 | -0.32185 | H | 4.258906 | 0.421275 | -0.70162 |
| H | -4.06261 | -0.48113 | 2.483639 | H | 3.807645 | 3.586683 | 0.243726 |
| H | -1.14664 | 0.129138 | -6.62325 | H | 1.671187 | 0.074582 | -6.46119 |
| H | 1.084368 | -5.16536 | -6.46924 | H | -5.29393 | -0.37333 | -5.31929 |
| H | 2.615048 | -2.34062 | -7.37215 | H | -2.87696 | -0.07769 | -7.6202 |
| H | 4.019926 | -4.32524 | -5.06992 | H | -3.42044 | -3.02651 | -6.16001 |
| H | -4.57313 | -2.45783 | -2.76329 | H | 2.253589 | -4.1284 | -2.53034 |
| H | -5.50309 | 0.982015 | -7.41796 | H | 5.533043 | -2.27453 | -7.01725 |
| H | -11.9252 | 0.183889 | -7.31437 | H | 11.04954 | -4.71032 | -4.72771 |
| H | -11.3543 | 2.916155 | -5.51071 | H | 9.732976 | -7.19343 | -6.50133 |
| H | -9.53782 | 2.341718 | -8.27736 | H | 9.469384 | -4.03595 | -7.61474 |
| H | -11.1379 | -2.56722 | -3.30307 | H | 8.401575 | -6.12729 | -0.87256 |
| H | -8.23538 | -2.31093 | -1.65479 | H | 5.067346 | -6.23867 | -1.05978 |
| H | -10.4898 | 0.154704 | -1.51056 | H | 6.93493 | -8.55762 | -2.59982 |
| H | 3.535398 | -5.9351 | -1.00244 | H | -5.19751 | 3.600533 | -3.48246 |
| H | 1.166178 | -7.38029 | -2.83303 | H | -3.43214 | 3.906255 | -6.28392 |
| H | -1.27685 | -8.63288 | 0.908229 | H | -1.40714 | 7.821212 | -4.85947 |
| H | 0.491227 | -8.80571 | 7.09544 | H | -5.11689 | 12.48135 | -2.3486 |
| H | -1.21947 | -10.555 | 4.796184 | H | -2.93838 | 11.70791 | 0.039911 |
| H | 1.839917 | -11.539 | 5.769194 | H | -1.93618 | 11.77903 | -3.16389 |
| H | 4.357907 | -6.10387 | 5.601657 | H | -5.80433 | 7.952461 | 1.527156 |
| H | 5.73996 | -8.86006 | 4.359872 | H | -8.02211 | 8.804642 | -0.79804 |
| H | 5.282119 | -6.18337 | 2.390553 | H | -6.8172 | 5.675569 | -0.69993 |
| H | -6.72939 | 5.965457 | 8.082616 | H | 8.23461 | 6.36997 | 4.817745 |
| H | -5.43751 | 7.644296 | 5.474941 | H | 5.07616 | 5.777401 | 5.756013 |
| H | -3.40265 | 6.080262 | 7.627895 | H | 5.828624 | 6.161125 | 2.504713 |
| H | -3.5712 | 1.4768 | 7.51824 | H | 10.29172 | 2.870728 | 2.427368 |
| H | -9.4709 | 3.440311 | 5.162103 | H | 8.538336 | -0.49008 | 6.396782 |
| H | -8.21359 | 4.90537 | 2.417591 | H | 9.831659 | 2.534848 | 7.075307 |
| H | -8.13577 | 1.56205 | 2.730373 | H | 6.747978 | 1.763285 | 8.134324 |
| H | 9.409589 | 0.046315 | 0.362117 | H | -9.25334 | -2.16818 | 1.637563 |
| H | 12.33332 | 3.6775 | 0.991271 | H | -11.1677 | -5.65671 | 4.143877 |
| H | 11.19307 | 7.906093 | -0.71381 | H | -8.65212 | -9.5213 | 5.054018 |
| H | 7.146803 | 8.488043 | -3.01978 | H | -4.24759 | -9.8742 | 3.47144 |
| H | 4.242569 | 4.91247 | -3.59874 | H | -2.35236 | -6.41691 | 1.021656 |
| **2D-5** | | | | **2D-6** | | | |
| Atom | X | Y | Z | Atom | X | Y | Z |
| C | 2.876877 | -1.49533 | -2.16735 | C | -2.95867 | 0.135535 | -1.45871 |
| C | 1.861135 | 1.077097 | -1.34019 | C | -1.3953 | -1.35004 | 0.469629 |
| C | 0.34557 | 1.11785 | 1.135767 | C | 0.606322 | 0.11832 | 1.962048 |
| C | 0.161893 | -1.5178 | 2.322481 | C | 0.228689 | 2.967539 | 1.690436 |
| C | -0.67082 | -3.62364 | 0.496308 | C | 0.222527 | 3.900281 | -1.06273 |
| C | 0.605241 | -3.40442 | -2.17698 | C | -1.34321 | 2.118867 | -2.90396 |
| C | 1.809711 | 2.896478 | 2.909415 | C | 0.352685 | -0.71202 | 4.733403 |
| O | 0.695432 | 5.1823 | 3.010604 | O | 2.075295 | -2.50914 | 5.288194 |
| C | -1.53148 | 5.305353 | 1.375649 | C | 3.620787 | -3.12865 | 3.079602 |
| C | -2.166 | 2.548634 | 0.769967 | C | 3.276572 | -0.87264 | 1.312445 |
| C | 5.245846 | -2.26708 | -0.66164 | C | -5.34558 | 1.187997 | -0.15156 |
| C | 7.593961 | -0.74542 | -1.00037 | C | -7.53858 | -0.53858 | 0.23179 |
| O | 5.225561 | -4.08243 | 0.771192 | O | -5.4523 | 3.392187 | 0.541956 |
| C | -1.19417 | -2.29074 | -4.14061 | C | 0.594238 | 0.754294 | -4.53023 |
| C | 1.523229 | -5.9495 | -3.27558 | C | -3.07685 | 3.570105 | -4.75491 |
| C | -3.62435 | -1.58976 | -3.91385 | C | 1.031919 | -1.73798 | -4.75494 |
| C | -5.06685 | -0.44948 | -5.95473 | C | 3.091917 | -2.79271 | -6.23876 |
| C | -7.41679 | 0.531724 | -5.7893 | C | 3.935583 | -5.19856 | -6.14663 |
| C | -8.6624 | 1.702562 | -8.06429 | C | 6.123591 | -6.03516 | -7.7622 |
| C | -8.98137 | 0.600357 | -3.41213 | C | 2.864092 | -7.22032 | -4.45166 |
| C | -0.40013 | -6.21716 | 1.823722 | C | -0.50841 | 6.731204 | -1.12937 |
| C | -1.95464 | -6.35602 | 4.213167 | C | 1.245912 | 8.334161 | 0.447926 |
| C | -4.45117 | -6.77833 | 4.350711 | C | 3.490753 | 9.265516 | -0.27502 |
| C | -5.81357 | -6.80208 | 6.853487 | C | 5.096886 | 10.80244 | 1.508243 |
| C | -6.11195 | -7.2358 | 2.079152 | C | 4.61871 | 8.872311 | -2.86259 |
| C | -3.60187 | 6.765322 | 2.799446 | C | 6.337743 | -3.60023 | 3.990342 |
| C | -2.71646 | 9.457486 | 3.399957 | C | 6.414239 | -5.83857 | 5.828356 |
| O | -4.2097 | 5.452967 | 5.089958 | O | 7.280843 | -1.36863 | 5.201552 |
| C | -6.01812 | 6.79842 | 1.218038 | C | 8.044925 | -4.07312 | 1.709679 |
| O | 2.169232 | 2.996631 | -2.57139 | O | -1.70937 | -3.60917 | 0.810059 |
| O | 3.713119 | 2.403189 | 4.072532 | O | -1.13918 | 0.072711 | 6.273112 |
| C | 7.846026 | 1.177613 | -2.81248 | C | -7.5163 | -3.11259 | -0.41647 |
| C | 10.10686 | 2.514967 | -3.01443 | C | -9.6481 | -4.60157 | 0.006608 |
| C | 12.13029 | 1.95368 | -1.41807 | C | -11.8216 | -3.54231 | 1.060734 |
| C | 11.89414 | 0.043313 | 0.393634 | C | -11.8606 | -0.98084 | 1.71281 |
| C | 9.645005 | -1.29693 | 0.596483 | C | -9.7344 | 0.505364 | 1.306264 |
| H | 3.494347 | -1.23973 | -4.12446 | H | -3.61608 | -1.2552 | -2.83304 |
| H | -1.1661 | -1.43503 | 3.905083 | H | 1.738699 | 3.93489 | 2.720111 |
| H | 2.003323 | -1.99065 | 3.124938 | H | -1.5479 | 3.482074 | 2.606786 |
| H | -2.70028 | -3.39842 | 0.218579 | H | 2.186691 | 3.813649 | -1.71776 |
| H | -0.958 | 6.338912 | -0.3158 | H | 2.831314 | -4.85523 | 2.268359 |
| H | -2.9355 | 2.352261 | -1.12763 | H | 3.508775 | -1.38502 | -0.66553 |
| H | -3.54625 | 1.841032 | 2.13461 | H | 4.656397 | 0.590647 | 1.792821 |
| H | -0.30653 | -2.01443 | -5.98324 | H | 1.80624 | 2.04533 | -5.58802 |
| H | -0.07798 | -7.22371 | -3.56723 | H | -4.42432 | 4.782258 | -3.77398 |
| H | 2.410002 | -5.63269 | -5.12013 | H | -1.95586 | 4.741487 | -6.03732 |
| H | 2.895796 | -6.86909 | -2.04523 | H | -4.12988 | 2.215271 | -5.91373 |
| H | -4.59666 | -1.81751 | -2.12436 | H | -0.12913 | -3.07917 | -3.73119 |
| H | -4.10906 | -0.34692 | -7.77596 | H | 4.071543 | -1.4724 | -7.48064 |
| H | -7.4479 | 1.627736 | -9.73586 | H | 5.572098 | -7.61149 | -8.99664 |
| H | -10.446 | 0.737159 | -8.51038 | H | 6.839038 | -4.50147 | -8.95011 |
| H | -9.14979 | 3.688224 | -7.70083 | H | 7.686865 | -6.73422 | -6.5875 |
| H | -9.39668 | 2.565286 | -2.88497 | H | 4.355199 | -7.97589 | -3.21986 |
| H | -10.8147 | -0.32082 | -3.7233 | H | 1.327826 | -6.56436 | -3.24026 |
| H | -8.07885 | -0.30844 | -1.79458 | H | 2.166985 | -8.82252 | -5.57203 |
| H | 1.590125 | -6.55259 | 2.259194 | H | -2.44534 | 6.93525 | -0.43946 |
| H | -0.99281 | -7.70532 | 0.517224 | H | -0.49129 | 7.388967 | -3.08819 |
| H | -0.94741 | -6.00966 | 5.975819 | H | 0.637023 | 8.708856 | 2.37936 |
| H | -4.52766 | -6.442 | 8.433039 | H | 6.952983 | 9.914718 | 1.790088 |
| H | -7.3119 | -5.36472 | 6.898111 | H | 5.465615 | 12.69501 | 0.736816 |
| H | -6.74675 | -8.6287 | 7.178104 | H | 4.198798 | 11.02121 | 3.358552 |
| H | -7.5012 | -5.70578 | 1.873858 | H | 3.376788 | 7.827299 | -4.13856 |
| H | -5.05213 | -7.36228 | 0.311888 | H | 5.073229 | 10.69311 | -3.74987 |
| H | -7.20069 | -8.98817 | 2.306583 | H | 6.407109 | 7.827111 | -2.72297 |
| H | -4.19655 | 10.45826 | 4.440384 | H | 8.352043 | -6.12639 | 6.489202 |
| H | -2.30351 | 10.50833 | 1.665208 | H | 5.765201 | -7.57656 | 4.910287 |
| H | -1.00125 | 9.427048 | 4.556069 | H | 5.202176 | -5.48625 | 7.467296 |
| H | -2.67843 | 5.426145 | 6.10493 | H | 6.172437 | -1.03755 | 6.629346 |
| H | -5.67981 | 7.689821 | -0.61735 | H | 7.37239 | -5.69439 | 0.614656 |
| H | -6.72884 | 4.883128 | 0.901624 | H | 8.105362 | -2.41927 | 0.471492 |
| H | -7.47845 | 7.875133 | 2.209245 | H | 9.968443 | -4.47247 | 2.354047 |
| H | 6.290112 | 1.66466 | -4.04795 | H | -5.8344 | -3.98039 | -1.19141 |
| H | 10.28607 | 3.997134 | -4.42156 | H | -9.60966 | -6.59159 | -0.49058 |
| H | 13.88727 | 3.001762 | -1.58127 | H | -13.4815 | -4.70723 | 1.376597 |
| H | 13.46334 | -0.39241 | 1.64172 | H | -13.5479 | -0.15282 | 2.535926 |
| H | 9.426166 | -2.78249 | 1.990424 | H | -9.72926 | 2.492626 | 1.804456 |
| **2D-7** | | | | **2D-8** | | | |
| Atom | X | Y | Z | Atom | X | Y | Z |
| C | 1.713613 | 0.585679 | -2.90772 | C | -2.95904 | 0.136442 | -1.45851 |
| C | 1.482261 | -0.82875 | -0.39547 | C | -1.39585 | -1.34975 | 0.46962 |
| C | -0.45511 | 0.165283 | 1.532485 | C | 0.606167 | 0.117942 | 1.962042 |
| C | -0.53341 | 3.089742 | 1.656647 | C | 0.229364 | 2.967318 | 1.690802 |
| C | -0.19507 | 4.59581 | -0.8134 | C | 0.223262 | 3.90034 | -1.06227 |
| C | 2.013154 | 3.468392 | -2.42724 | C | -1.34322 | 2.119674 | -2.90361 |
| C | -2.96495 | -1.11138 | 0.786372 | C | 0.352436 | -0.7124 | 4.733456 |
| O | -3.39858 | -3.13293 | 2.27136 | O | 2.074676 | -2.50972 | 5.288193 |
| C | -1.38982 | -3.49899 | 4.14045 | C | 3.61991 | -3.12972 | 3.079511 |
| C | 0.029514 | -0.98624 | 4.154287 | C | 3.276005 | -0.8737 | 1.312247 |
| C | -0.34473 | -0.25846 | -4.80317 | C | -5.34565 | 1.189084 | -0.151 |
| C | -0.61634 | -3.02342 | -5.31319 | C | -7.53902 | -0.53704 | 0.231941 |
| O | -1.68686 | 1.250022 | -5.91488 | O | -5.45173 | 3.393071 | 0.543311 |
| C | 4.405416 | 3.913014 | -0.88252 | C | 0.593867 | 0.754997 | -4.53021 |
| C | 2.406521 | 4.931938 | -4.92207 | C | -3.07668 | 3.571598 | -4.75417 |
| C | 6.274669 | 2.294938 | -0.29154 | C | 1.03111 | -1.73731 | -4.75524 |
| C | 8.489306 | 3.026334 | 1.16076 | C | 3.091106 | -2.79218 | -6.23893 |
| C | 10.3578 | 1.486833 | 1.965703 | C | 3.934508 | -5.1981 | -6.14687 |
| C | 12.56053 | 2.524011 | 3.439205 | C | 6.122544 | -6.03484 | -7.76236 |
| C | 10.44617 | -1.32166 | 1.508532 | C | 2.862557 | -7.22008 | -4.45252 |
| C | -2.74196 | 5.180718 | -2.13158 | C | -0.50651 | 6.731552 | -1.12865 |
| C | -4.20593 | 7.084674 | -0.60453 | C | 1.248748 | 8.333704 | 0.448424 |
| C | -6.45298 | 6.827562 | 0.542886 | C | 3.494066 | 9.263746 | -0.27467 |
| C | -7.59643 | 8.993469 | 2.0064 | C | 5.1011 | 10.79997 | 1.508385 |
| C | -8.04615 | 4.466275 | 0.498217 | C | 4.621792 | 8.869566 | -2.86221 |
| C | -2.6584 | -4.24781 | 6.645452 | C | 6.336794 | -3.60187 | 3.990028 |
| C | -4.12647 | -6.72628 | 6.334833 | C | 6.413016 | -5.84018 | 5.828059 |
| O | -4.33262 | -2.26688 | 7.428275 | O | 7.280457 | -1.37047 | 5.20114 |
| C | -0.6565 | -4.48144 | 8.711541 | C | 8.043778 | -4.07515 | 1.709297 |
| O | 2.742276 | -2.69744 | 0.075487 | O | -1.71014 | -3.60889 | 0.809511 |
| O | -4.39884 | -0.5498 | -0.90331 | O | -1.13911 | 0.072898 | 6.273212 |
| C | 1.362455 | -4.76599 | -5.03685 | C | -7.51709 | -3.111 | -0.4164 |
| C | 0.976444 | -7.30716 | -5.61897 | C | -9.64927 | -4.5996 | 0.006213 |
| C | -1.38553 | -8.1348 | -6.4514 | C | -11.8228 | -3.53997 | 1.059862 |
| C | -3.36862 | -6.40925 | -6.72546 | C | -11.8614 | -0.97849 | 1.712003 |
| C | -2.97947 | -3.86893 | -6.17919 | C | -9.73487 | 0.507297 | 1.305992 |
| H | 3.479129 | -0.10373 | -3.74553 | H | -3.61681 | -1.25403 | -2.83293 |
| H | 1.004688 | 3.603883 | 2.941041 | H | 1.73981 | 3.934073 | 2.720387 |
| H | -2.29129 | 3.666662 | 2.581362 | H | -1.54699 | 3.482283 | 2.607338 |
| H | 0.521625 | 6.451627 | -0.21646 | H | 2.187301 | 3.812976 | -1.71755 |
| H | -0.20841 | -5.04077 | 3.44237 | H | 2.82985 | -4.85613 | 2.268439 |
| H | 2.040368 | -1.22814 | 4.530858 | H | 3.507942 | -1.38645 | -0.66567 |
| H | -0.79603 | 0.249831 | 5.589598 | H | 4.656344 | 0.589232 | 1.792214 |
| H | 4.597428 | 5.864478 | -0.24154 | H | 1.806081 | 2.045989 | -5.58781 |
| H | 0.778709 | 4.787107 | -6.17119 | H | -4.13021 | 2.217131 | -5.91298 |
| H | 2.75848 | 6.930382 | -4.50829 | H | -4.42372 | 4.784029 | -3.77301 |
| H | 4.06695 | 4.191575 | -5.91196 | H | -1.95548 | 4.742725 | -6.03662 |
| H | 6.174198 | 0.340241 | -0.89244 | H | -0.1302 | -3.07842 | -3.73166 |
| H | 8.619249 | 5.026679 | 1.638856 | H | 4.071063 | -1.47182 | -7.4805 |
| H | 14.35306 | 2.125271 | 2.468942 | H | 6.83831 | -4.50115 | -8.95005 |
| H | 12.41414 | 4.56812 | 3.714183 | H | 7.685562 | -6.73435 | -6.58762 |
| H | 12.69562 | 1.622407 | 5.305036 | H | 5.570816 | -7.6109 | -8.99705 |
| H | 12.10591 | -1.82214 | 0.366034 | H | 4.353903 | -7.97771 | -3.22229 |
| H | 10.65267 | -2.33187 | 3.309776 | H | 1.327621 | -6.56375 | -3.23967 |
| H | 8.76807 | -2.06046 | 0.562345 | H | 2.16353 | -8.82101 | -5.57356 |
| H | -3.80888 | 3.455298 | -2.44875 | H | -2.44321 | 6.936383 | -0.43838 |
| H | -2.35391 | 5.999962 | -3.99178 | H | -0.4894 | 7.389379 | -3.08746 |
| H | -3.2583 | 8.908598 | -0.40245 | H | 0.640096 | 8.708865 | 2.379834 |
| H | -7.93039 | 8.458255 | 3.985105 | H | 6.956734 | 9.911289 | 1.790164 |
| H | -6.38271 | 10.66839 | 1.986431 | H | 5.470717 | 12.69227 | 0.736761 |
| H | -9.4467 | 9.525025 | 1.226801 | H | 4.203201 | 11.01931 | 3.35871 |
| H | -8.44898 | 3.833349 | 2.435207 | H | 6.409065 | 7.822451 | -2.72256 |
| H | -9.88881 | 4.851122 | -0.37987 | H | 3.378847 | 7.825934 | -4.1383 |
| H | -7.1564 | 2.903224 | -0.50498 | H | 5.078244 | 10.68994 | -3.74933 |
| H | -5.57849 | -6.54359 | 4.872699 | H | 5.763391 | -7.57803 | 4.910145 |
| H | -5.04583 | -7.21606 | 8.120728 | H | 5.201288 | -5.48751 | 7.46717 |
| H | -2.86375 | -8.27544 | 5.796304 | H | 8.35084 | -6.12851 | 6.488639 |
| H | -5.61244 | -2.11016 | 6.119357 | H | 6.17245 | -1.03934 | 6.629224 |
| H | -1.55596 | -5.05111 | 10.48388 | H | 7.371034 | -5.69647 | 0.614507 |
| H | 0.754728 | -5.90619 | 8.205952 | H | 8.104298 | -2.42148 | 0.47088 |
| H | 0.30476 | -2.67818 | 9.022556 | H | 9.967304 | -4.47452 | 2.353626 |
| H | 3.198532 | -4.15985 | -4.37011 | H | -5.83516 | -3.97911 | -1.19094 |
| H | 2.523459 | -8.64002 | -5.41905 | H | -9.61105 | -6.58961 | -0.49101 |
| H | -1.68326 | -10.1185 | -6.88579 | H | -13.483 | -4.70459 | 1.375348 |
| H | -5.21032 | -7.04926 | -7.36519 | H | -13.5487 | -0.15014 | 2.534709 |
| H | -4.49393 | -2.50442 | -6.38241 | H | -9.72944 | 2.494529 | 1.804294 |
| **2D-9** | | | | **2D-10** | | | |
| Atom | X | Y | Z | Atom | X | Y | Z |
| C | -2.95866 | 0.13572 | -1.45897 | C | -2.53443 | -1.98874 | 1.285596 |
| C | -1.3954 | -1.35014 | 0.469376 | C | -2.47536 | 0.893406 | 1.137409 |
| C | 0.606196 | 0.118036 | 1.962018 | C | -1.18684 | 2.036435 | -1.19401 |
| C | 0.228627 | 2.967282 | 1.690691 | C | -0.54196 | 0.000981 | -3.13975 |
| C | 0.222562 | 3.900365 | -1.06237 | C | 1.103198 | -2.13124 | -2.04585 |
| C | -1.34305 | 2.119224 | -2.90392 | C | 0.140543 | -3.06309 | 0.629301 |
| C | 0.352379 | -0.71248 | 4.733346 | C | -3.03167 | 3.985175 | -2.2904 |
| O | 2.075027 | -2.50948 | 5.288179 | O | -2.37785 | 6.339572 | -1.56806 |
| C | 3.620545 | -3.12902 | 3.079549 | C | -0.13348 | 6.322049 | 0.047899 |
| C | 3.276377 | -0.87295 | 1.312454 | C | 1.05172 | 3.716528 | -0.36776 |
| C | -5.34556 | 1.188054 | -0.15178 | C | -4.7725 | -3.08425 | -0.2226 |
| C | -7.53856 | -0.53855 | 0.231495 | C | -7.36807 | -2.79654 | 0.836174 |
| O | -5.45235 | 3.392227 | 0.541813 | O | -4.44153 | -4.18783 | -2.22913 |
| C | 0.594538 | 0.754883 | -4.5302 | C | 1.888674 | -2.08363 | 2.712403 |
| C | -3.07653 | 3.570734 | -4.75483 | C | 0.051028 | -5.96954 | 0.940092 |
| C | 1.032021 | -1.73738 | -4.75535 | C | 4.428834 | -2.08443 | 2.631578 |
| C | 3.092308 | -2.79202 | -6.23883 | C | 6.013592 | -1.24092 | 4.706348 |
| C | 3.935906 | -5.19788 | -6.14672 | C | 8.564184 | -1.16962 | 4.757597 |
| C | 6.124372 | -6.03436 | -7.76176 | C | 9.94468 | -0.225 | 7.059544 |
| C | 2.863918 | -7.21986 | -4.45235 | C | 10.24583 | -1.98659 | 2.612664 |
| C | -0.50841 | 6.731288 | -1.12868 | C | 1.498785 | -4.18081 | -4.0955 |
| C | 1.246004 | 8.334174 | 0.448612 | C | 3.787338 | -5.83977 | -3.69199 |
| C | 3.490887 | 9.265388 | -0.27436 | C | 6.208795 | -5.14145 | -3.98401 |
| C | 5.097086 | 10.80235 | 1.508825 | C | 8.343481 | -6.95949 | -3.47188 |
| C | 4.618819 | 8.872043 | -2.86195 | C | 7.020784 | -2.54341 | -4.83359 |
| C | 6.337435 | -3.60068 | 3.990295 | C | 1.52582 | 8.578368 | -0.73095 |
| C | 6.41395 | -5.83924 | 5.828019 | C | 0.099175 | 11.06866 | -0.33443 |
| O | 7.280455 | -1.3692 | 5.201823 | O | 2.228475 | 8.312881 | -3.33372 |
| C | 8.044744 | -4.07326 | 1.70965 | C | 3.985066 | 8.541611 | 0.780765 |
| O | -1.70964 | -3.60923 | 0.809602 | O | -3.32678 | 2.238687 | 2.801296 |
| O | -1.13957 | 0.072229 | 6.272989 | O | -4.84271 | 3.565414 | -3.61665 |
| C | -7.51634 | -3.11245 | -0.41715 | C | -7.88445 | -1.60762 | 3.152861 |
| C | -9.6481 | -4.60151 | 0.005943 | C | -10.3665 | -1.41323 | 4.014564 |
| C | -11.8215 | -3.54241 | 1.060435 | C | -12.3508 | -2.40505 | 2.587547 |
| C | -11.8604 | -0.98101 | 1.712852 | C | -11.8538 | -3.591 | 0.277886 |
| C | -9.73429 | 0.505252 | 1.306311 | C | -9.3827 | -3.78303 | -0.58939 |
| H | -3.616 | -1.25489 | -2.83345 | H | -2.90822 | -2.42728 | 3.2696 |
| H | 1.738639 | 3.934504 | 2.720478 | H | 0.477727 | 0.892614 | -4.70638 |
| H | -1.54798 | 3.481739 | 2.607051 | H | -2.29011 | -0.76109 | -3.92539 |
| H | 2.186744 | 3.813815 | -1.71734 | H | 2.959283 | -1.2942 | -1.72239 |
| H | 2.830953 | -4.85554 | 2.268269 | H | -0.80118 | 6.55437 | 1.986964 |
| H | 3.508679 | -1.38534 | -0.6655 | H | 2.016086 | 3.015918 | 1.312065 |
| H | 4.656272 | 0.590267 | 1.792857 | H | 2.411867 | 3.818677 | -1.92073 |
| H | 1.806803 | 2.046063 | -5.58751 | H | 0.975777 | -1.47101 | 4.456182 |
| H | -4.42398 | 4.782903 | -3.77388 | H | -0.68305 | -6.44131 | 2.81854 |
| H | -1.95535 | 4.742117 | -6.03707 | H | -1.15638 | -6.86629 | -0.47032 |
| H | -4.12957 | 2.216019 | -5.91378 | H | 1.952669 | -6.75279 | 0.798045 |
| H | -0.1294 | -3.07864 | -3.7321 | H | 5.360618 | -2.76241 | 0.930461 |
| H | 4.07226 | -1.47157 | -7.4803 | H | 5.004269 | -0.59269 | 6.382248 |
| H | 7.687315 | -6.73343 | -6.58664 | H | 11.20672 | -1.68873 | 7.819534 |
| H | 5.573243 | -7.61065 | -8.99642 | H | 11.15293 | 1.395978 | 6.584503 |
| H | 6.840111 | -4.5006 | -8.9494 | H | 8.643968 | 0.352848 | 8.559388 |
| H | 2.166683 | -8.82168 | -5.57318 | H | 11.54507 | -3.47929 | 3.23904 |
| H | 4.3548 | -7.97598 | -3.22061 | H | 9.208928 | -2.69535 | 0.977004 |
| H | 1.327623 | -6.56397 | -3.24095 | H | 11.43364 | -0.40558 | 1.980943 |
| H | -2.44528 | 6.935217 | -0.4386 | H | 1.692441 | -3.17951 | -5.90273 |
| H | -0.49144 | 7.389218 | -3.08745 | H | -0.20883 | -5.32758 | -4.26427 |
| H | 0.637098 | 8.708952 | 2.380014 | H | 3.425765 | -7.77275 | -3.08833 |
| H | 6.953157 | 9.914596 | 1.790684 | H | 9.501709 | -7.24811 | -5.17096 |
| H | 5.465802 | 12.69486 | 0.737271 | H | 9.622249 | -6.21856 | -2.01329 |
| H | 4.198983 | 11.02122 | 3.359113 | H | 7.64809 | -8.8015 | -2.83881 |
| H | 3.37684 | 7.82696 | -4.13781 | H | 8.373229 | -2.68235 | -6.40093 |
| H | 5.073268 | 10.69279 | -3.74936 | H | 5.442649 | -1.36536 | -5.45379 |
| H | 6.407179 | 7.826807 | -2.72234 | H | 7.999831 | -1.53307 | -3.30618 |
| H | 8.35175 | -6.12713 | 6.488849 | H | -0.3971 | 11.33212 | 1.657677 |
| H | 5.76494 | -7.57714 | 4.909737 | H | -1.64815 | 11.09966 | -1.44117 |
| H | 5.201868 | -5.48714 | 7.466994 | H | 1.288145 | 12.651 | -0.93218 |
| H | 6.171984 | -1.0383 | 6.629605 | H | 0.668421 | 8.324801 | -4.30442 |
| H | 7.372498 | -5.69462 | 0.61459 | H | 3.599452 | 8.640249 | 2.81037 |
| H | 8.10499 | -2.41941 | 0.471456 | H | 5.070082 | 6.824918 | 0.395624 |
| H | 9.968294 | -4.47229 | 2.354121 | H | 5.143451 | 10.17156 | 0.255304 |
| H | -5.83453 | -3.98011 | -1.19243 | H | -6.37736 | -0.79228 | 4.27011 |
| H | -9.60968 | -6.59146 | -0.49154 | H | -10.7485 | -0.4866 | 5.804642 |
| H | -13.4813 | -4.70739 | 1.376333 | H | -14.2806 | -2.25444 | 3.269519 |
| H | -13.5476 | -0.15311 | 2.536215 | H | -13.3944 | -4.36032 | -0.83768 |
| H | -9.7291 | 2.492439 | 1.8048 | H | -8.96228 | -4.69265 | -2.3764 |
| **2D-11** | | | | **2D-12** | | | |
| Atom | X | Y | Z | Atom | X | Y | Z |
| C | 2.457848 | 0.597352 | -2.92373 | C | -1.11722 | -2.62952 | -0.3661 |
| C | 0.672784 | 1.982228 | -1.12388 | C | -1.59483 | 0.032452 | 0.633429 |
| C | -0.45876 | 0.393564 | 1.024649 | C | -1.53287 | 2.214997 | -1.28813 |
| C | 0.428837 | -2.36661 | 0.916854 | C | -0.3946 | 1.331737 | -3.80466 |
| C | 0.31371 | -3.6599 | -1.69292 | C | 2.006067 | -0.19501 | -3.25015 |
| C | 1.042736 | -1.8223 | -3.90815 | C | 1.36299 | -2.83676 | -2.0435 |
| C | 0.540732 | 1.615145 | 3.473594 | C | -4.25687 | 3.189247 | -1.57223 |
| O | -1.25778 | 3.029488 | 4.580101 | O | -4.61306 | 5.254038 | -0.1183 |
| C | -3.59425 | 3.017454 | 3.131849 | C | -2.31289 | 5.973456 | 1.23572 |
| C | -3.33053 | 0.855592 | 1.221536 | C | -0.19688 | 4.516735 | -0.0944 |
| C | 5.098528 | 0.233156 | -1.75522 | C | -3.62247 | -3.52508 | -1.57579 |
| C | 6.634248 | 2.530056 | -1.19726 | C | -5.51 | -4.77549 | 0.109876 |
| O | 5.940581 | -1.8708 | -1.29315 | O | -4.08501 | -3.22432 | -3.81911 |
| C | -1.29472 | -0.71939 | -5.1951 | C | 3.531484 | -3.58542 | -0.30114 |
| C | 2.645337 | -3.06211 | -6.0062 | C | 1.063544 | -4.93004 | -4.04349 |
| C | -3.74774 | -1.04915 | -4.63032 | C | 4.075075 | -2.51961 | 1.940469 |
| C | -5.7969 | 0.329865 | -5.8315 | C | 6.272052 | -3.19507 | 3.440617 |
| C | -8.21518 | 0.414859 | -5.01829 | C | 7.011115 | -2.14775 | 5.647227 |
| C | -10.1348 | 2.046976 | -6.33887 | C | 9.379113 | -3.03311 | 6.95393 |
| C | -9.19238 | -0.97709 | -2.7299 | C | 5.620909 | -0.05556 | 6.985679 |
| C | 1.848749 | -6.1524 | -1.58986 | C | 3.773101 | -0.30034 | -5.56738 |
| C | 0.907736 | -7.90625 | 0.440079 | C | 6.282623 | -1.57936 | -5.09701 |
| C | 2.031378 | -8.47348 | 2.641183 | C | 8.143814 | -0.72188 | -3.60579 |
| C | 0.809154 | -10.2664 | 4.491361 | C | 10.4883 | -2.26779 | -3.13626 |
| C | 4.530558 | -7.40852 | 3.504508 | C | 8.02283 | 1.739151 | -2.17876 |
| C | -5.83914 | 2.833149 | 4.968258 | C | -2.09522 | 8.869872 | 1.114636 |
| C | -5.708 | 0.488241 | 6.658388 | C | -4.37427 | 10.10998 | 2.404055 |
| O | -7.9647 | 2.685839 | 3.280355 | O | -1.95215 | 9.650023 | -1.47573 |
| C | -6.00778 | 5.240207 | 6.568183 | C | 0.374818 | 9.708631 | 2.35125 |
| O | 0.122433 | 4.202917 | -1.36746 | O | -2.0181 | 0.430368 | 2.86418 |
| O | 2.644883 | 1.404822 | 4.340389 | O | -5.91727 | 2.316625 | -2.87128 |
| C | 8.860637 | 2.193377 | 0.213486 | C | -7.58759 | -5.95346 | -1.05391 |
| C | 10.39004 | 4.2444 | 0.805265 | C | -9.429 | -7.1469 | 0.389864 |
| C | 9.721972 | 6.666236 | -0.0169 | C | -9.23692 | -7.15487 | 3.025935 |
| C | 7.518132 | 7.02082 | -1.42225 | C | -7.19758 | -5.96505 | 4.202054 |
| C | 5.974719 | 4.968466 | -2.01101 | C | -5.33216 | -4.79109 | 2.758327 |
| H | 2.711687 | 1.872581 | -4.53238 | H | -0.80994 | -3.79878 | 1.297023 |
| H | -0.67546 | -3.46651 | 2.272486 | H | 0.056524 | 2.997091 | -4.94804 |
| H | 2.378075 | -2.41509 | 1.592478 | H | -1.76895 | 0.202435 | -4.84863 |
| H | -1.63802 | -4.24978 | -2.01727 | H | 3.041807 | 0.843816 | -1.79206 |
| H | -3.68279 | 4.831691 | 2.150799 | H | -2.55341 | 5.349709 | 3.186993 |
| H | -4.18403 | 1.3796 | -0.57187 | H | 1.307381 | 3.994416 | 1.214015 |
| H | -4.25767 | -0.86083 | 1.891947 | H | 0.596683 | 5.686052 | -1.60119 |
| H | -0.84116 | 0.607126 | -6.70958 | H | 4.784063 | -5.0513 | -1.01535 |
| H | 4.436495 | -3.78189 | -5.2854 | H | -0.24916 | -4.40311 | -5.53876 |
| H | 1.602631 | -4.61779 | -6.88221 | H | 2.898008 | -5.35692 | -4.88845 |
| H | 3.051326 | -1.67219 | -7.48679 | H | 0.36512 | -6.66939 | -3.16221 |
| H | -4.28042 | -2.32366 | -3.11754 | H | 2.850011 | -1.04485 | 2.678821 |
| H | -5.28145 | 1.477802 | -7.46256 | H | 7.446041 | -4.69392 | 2.651059 |
| H | -9.31118 | 3.083372 | -7.92722 | H | 8.957514 | -3.73366 | 8.863216 |
| H | -11.7146 | 0.903274 | -7.05152 | H | 10.3234 | -4.54321 | 5.903077 |
| H | -10.9606 | 3.423821 | -5.02144 | H | 10.72402 | -1.46834 | 7.192245 |
| H | -11.0922 | -1.72225 | -3.09128 | H | 6.837218 | 1.616672 | 7.173353 |
| H | -7.99155 | -2.55654 | -2.15875 | H | 3.885033 | 0.526279 | 6.033569 |
| H | -9.34054 | 0.305949 | -1.10355 | H | 5.125793 | -0.63576 | 8.916272 |
| H | 3.841764 | -5.70638 | -1.33667 | H | 4.077298 | 1.664329 | -6.16446 |
| H | 1.662524 | -7.1147 | -3.41512 | H | 2.800451 | -1.22663 | -7.14248 |
| H | -0.92943 | -8.76715 | 0.062992 | H | 6.559954 | -3.40578 | -6.00507 |
| H | 2.040505 | -11.8932 | 4.879139 | H | 10.46676 | -4.04425 | -4.19546 |
| H | 0.478885 | -9.33636 | 6.318698 | H | 12.20404 | -1.21814 | -3.65041 |
| H | -1.00255 | -10.9796 | 3.793032 | H | 10.65914 | -2.72397 | -1.11663 |
| H | 4.284185 | -6.34971 | 5.27377 | H | 9.845063 | 2.72559 | -2.25349 |
| H | 5.874871 | -8.93521 | 3.919757 | H | 6.570525 | 3.018701 | -2.90147 |
| H | 5.397801 | -6.13874 | 2.129404 | H | 7.611987 | 1.380109 | -0.17427 |
| H | -7.35719 | 0.439486 | 7.909146 | H | -4.47123 | 9.587221 | 4.404489 |
| H | -4.01239 | 0.522783 | 7.840005 | H | -6.14065 | 9.524157 | 1.500911 |
| H | -5.70453 | -1.25271 | 5.545433 | H | -4.21277 | 12.16668 | 2.267662 |
| H | -9.47742 | 2.579295 | 4.312843 | H | -3.51856 | 9.11698 | -2.27464 |
| H | -4.32819 | 5.459714 | 7.754546 | H | 0.45426 | 9.088615 | 4.322756 |
| H | -6.17972 | 6.901346 | 5.347133 | H | 2.009128 | 8.937108 | 1.347927 |
| H | -7.66556 | 5.162911 | 7.807368 | H | 0.503907 | 11.77167 | 2.308571 |
| H | 9.347419 | 0.302721 | 0.835119 | H | -7.71479 | -5.91661 | -3.09855 |
| H | 12.09942 | 3.964872 | 1.905245 | H | -11.0149 | -8.06806 | -0.53029 |
| H | 10.91672 | 8.27114 | 0.44027 | H | -10.6759 | -8.08272 | 4.157509 |
| H | 6.996662 | 8.898641 | -2.06329 | H | -7.05438 | -5.95078 | 6.248616 |
| H | 4.258065 | 5.288724 | -3.07609 | H | -3.79068 | -3.83812 | 3.709544 |
| **2D-13** | | | | **2D-14** | | | |
| Atom | X | Y | Z | Atom | X | Y | Z |
| C | -2.04503 | 1.070386 | 1.128582 | C | -1.99806 | -1.73793 | 1.522216 |
| C | 0.50292 | 1.881196 | 0.031111 | C | -2.23907 | 1.112516 | 1.147243 |
| C | 1.430158 | 0.476867 | -2.32483 | C | -1.22632 | 2.167707 | -1.35596 |
| C | -0.69516 | -1.07167 | -3.51765 | C | -0.45949 | 0.043048 | -3.15828 |
| C | -1.94316 | -2.97928 | -1.70882 | C | 1.458122 | -1.81024 | -1.99822 |
| C | -2.43789 | -1.84194 | 1.029305 | C | 0.743288 | -2.58437 | 0.802852 |
| C | 2.419668 | 2.494879 | -4.1641 | C | -3.35465 | 3.777691 | -2.49023 |
| O | 4.954427 | 2.704402 | -3.97867 | O | -2.95442 | 6.248595 | -2.01646 |
| C | 5.96318 | 0.977213 | -2.06793 | C | -0.63766 | 6.64303 | -0.5571 |
| C | 3.881664 | -0.99126 | -1.71663 | C | 0.833078 | 4.167126 | -0.83066 |
| C | -4.16496 | 2.645838 | -0.11262 | C | -4.17949 | -3.17324 | 0.233033 |
| C | -4.63858 | 5.252225 | 0.845295 | C | -6.74657 | -3.0182 | 1.385084 |
| O | -5.44511 | 1.777013 | -1.83255 | O | -3.8278 | -4.43197 | -1.67636 |
| C | -0.54382 | -3.11472 | 2.775212 | C | 2.470441 | -1.24422 | 2.69251 |
| C | -5.0766 | -2.44263 | 2.129978 | C | 0.984113 | -5.44167 | 1.376932 |
| C | 1.293965 | -2.07034 | 4.184218 | C | 4.97848 | -0.90984 | 2.45593 |
| C | 3.114907 | -3.53216 | 5.635309 | C | 6.540658 | 0.259524 | 4.385304 |
| C | 5.271882 | -2.6498 | 6.673196 | C | 9.05775 | 0.667829 | 4.281792 |
| C | 7.017732 | -4.38801 | 8.097386 | C | 10.41429 | 1.91803 | 6.448549 |
| C | 6.167354 | 0.046899 | 6.48712 | C | 10.72415 | -0.04784 | 2.088786 |
| C | -4.24104 | -4.17311 | -3.06383 | C | 1.945579 | -3.99473 | -3.87761 |
| C | -4.95332 | -6.74543 | -2.08704 | C | 4.526969 | -5.15445 | -3.60468 |
| C | -7.24399 | -7.6119 | -1.43531 | C | 5.160598 | -7.59801 | -3.37982 |
| C | -7.58829 | -10.2894 | -0.5172 | C | 7.890946 | -8.38091 | -3.12838 |
| C | -9.63726 | -6.06983 | -1.50496 | C | 3.299653 | -9.75273 | -3.323 |
| C | 8.503206 | -0.04579 | -3.03424 | C | 0.684011 | 9.000008 | -1.62799 |
| C | 10.38819 | 2.122195 | -3.41012 | C | -1.01363 | 11.33024 | -1.34078 |
| O | 8.115489 | -1.35822 | -5.37171 | O | 1.266075 | 8.588837 | -4.24138 |
| C | 9.524384 | -1.99511 | -1.16411 | C | 3.209961 | 9.388851 | -0.2856 |
| O | 1.76171 | 3.562478 | 0.983878 | O | -3.10731 | 2.508413 | 2.759896 |
| O | 1.186691 | 3.760854 | -5.60944 | O | -5.17364 | 3.026788 | -3.64905 |
| C | -3.17231 | 6.417419 | 2.726467 | C | -8.72129 | -4.28663 | 0.13782 |
| C | -3.73741 | 8.866372 | 3.516289 | C | -11.1639 | -4.23076 | 1.100036 |
| C | -5.77152 | 10.16882 | 2.456349 | C | -11.6719 | -2.90245 | 3.328373 |
| C | -7.24154 | 9.023541 | 0.581452 | C | -9.72748 | -1.63239 | 4.578924 |
| C | -6.67475 | 6.588167 | -0.22017 | C | -7.27387 | -1.68795 | 3.621076 |
| H | -1.99765 | 1.623205 | 3.115265 | H | -2.22876 | -2.0463 | 3.552388 |
| H | 0.074308 | -2.1023 | -5.14008 | H | 0.375131 | 0.890343 | -4.85299 |
| H | -2.11935 | 0.227703 | -4.2542 | H | -2.15319 | -0.96194 | -3.77059 |
| H | -0.57169 | -4.51199 | -1.44542 | H | 3.23406 | -0.76249 | -1.87425 |
| H | 6.250076 | 2.091188 | -0.35365 | H | -1.22032 | 6.969781 | 1.395406 |
| H | 3.900072 | -1.82368 | 0.163868 | H | 1.970293 | 3.741524 | 0.833328 |
| H | 4.115755 | -2.49386 | -3.1178 | H | 2.078531 | 4.285572 | -2.47597 |
| H | -0.72479 | -5.16908 | 2.790298 | H | 1.58607 | -0.65436 | 4.459066 |
| H | -6.6001 | -1.75923 | 0.923144 | H | -0.2616 | -6.57824 | 0.192147 |
| H | -5.31508 | -4.4761 | 2.37449 | H | 2.92753 | -6.05984 | 1.083068 |
| H | -5.25812 | -1.54297 | 3.986449 | H | 0.490781 | -5.78773 | 3.358376 |
| H | 1.530903 | -0.036 | 4.214454 | H | 5.911721 | -1.55076 | 0.742003 |
| H | 2.704489 | -5.54242 | 5.824436 | H | 5.541112 | 0.866738 | 6.082231 |
| H | 7.295354 | -3.72509 | 10.04582 | H | 9.126606 | 2.402668 | 7.99202 |
| H | 6.29378 | -6.32348 | 8.168383 | H | 11.90126 | 0.68114 | 7.204802 |
| H | 8.900208 | -4.4274 | 7.221469 | H | 11.36474 | 3.655358 | 5.822893 |
| H | 4.905693 | 1.271769 | 5.407468 | H | 12.21708 | -1.34874 | 2.711438 |
| H | 6.408898 | 0.870841 | 8.376744 | H | 9.707574 | -0.94551 | 0.533942 |
| H | 8.034157 | 0.112926 | 5.579853 | H | 11.68843 | 1.630289 | 1.338482 |
| H | -3.69034 | -4.38523 | -5.05933 | H | 1.819922 | -3.17002 | -5.78335 |
| H | -5.82041 | -2.84885 | -3.05071 | H | 0.426972 | -5.3829 | -3.76036 |
| H | -3.36852 | -8.05949 | -1.94003 | H | 6.068366 | -3.78117 | -3.62322 |
| H | -5.80038 | -11.3288 | -0.48195 | H | 9.165635 | -6.75212 | -3.15974 |
| H | -8.39204 | -10.318 | 1.397787 | H | 8.203789 | -9.41305 | -1.35352 |
| H | -8.91942 | -11.3293 | -1.72497 | H | 8.445158 | -9.6692 | -4.65968 |
| H | -10.4612 | -5.92688 | 0.395775 | H | 3.732961 | -11.1283 | -4.81554 |
| H | -9.35247 | -4.15613 | -2.22098 | H | 3.431595 | -10.7773 | -1.52177 |
| H | -11.065 | -6.99194 | -2.69631 | H | 1.344053 | -9.14317 | -3.56654 |
| H | 9.658566 | 3.504435 | -4.76524 | H | -0.06429 | 12.98227 | -2.14347 |
| H | 12.17474 | 1.370584 | -4.12984 | H | -1.42425 | 11.70906 | 0.652284 |
| H | 10.75633 | 3.104506 | -1.62567 | H | -2.81146 | 11.05706 | -2.32672 |
| H | 7.426971 | -0.13221 | -6.5547 | H | -0.3359 | 8.330395 | -5.10332 |
| H | 9.76618 | -1.15515 | 0.710983 | H | 4.474365 | 7.783946 | -0.5987 |
| H | 8.246277 | -3.61143 | -1.00346 | H | 4.127687 | 11.08989 | -1.01963 |
| H | 11.36262 | -2.68292 | -1.81394 | H | 2.928686 | 9.623474 | 1.749845 |
| H | -1.55846 | 5.457833 | 3.537282 | H | -8.29324 | -5.30334 | -1.58866 |
| H | -2.58688 | 9.755734 | 4.963409 | H | -12.674 | -5.21647 | 0.121358 |
| H | -6.21143 | 12.07213 | 3.085632 | H | -13.5793 | -2.85776 | 4.084426 |
| H | -8.82286 | 10.03371 | -0.2485 | H | -10.1185 | -0.59599 | 6.305767 |
| H | -7.79046 | 5.673375 | -1.67477 | H | -5.8012 | -0.66039 | 4.600684 |
| **2D-15** | | | | **2D-16** | | | |
| Atom | X | Y | Z | Atom | X | Y | Z |
| C | 3.01623 | -1.2598 | -2.20882 | C | -0.14046 | -1.81749 | -3.28618 |
| C | 1.829244 | 1.202782 | -1.27529 | C | 0.207896 | 0.853349 | -2.24958 |
| C | 0.196843 | 1.01497 | 1.117312 | C | 0.147727 | 1.157712 | 0.644771 |
| C | 0.110507 | -1.69328 | 2.148415 | C | -2.08438 | -0.31774 | 1.841202 |
| C | -0.48843 | -3.75399 | 0.18189 | C | -2.86152 | -2.87288 | 0.656423 |
| C | 0.85882 | -3.28774 | -2.42238 | C | -2.74179 | -2.81544 | -2.28354 |
| C | 1.483231 | 2.750426 | 3.067702 | C | 2.770813 | 0.361686 | 1.61595 |
| O | 0.211965 | 4.939032 | 3.310282 | O | 4.247301 | 2.414747 | 1.922243 |
| C | -1.95462 | 5.046357 | 1.621651 | C | 2.880935 | 4.755044 | 1.345428 |
| C | -2.36939 | 2.33388 | 0.694023 | C | 0.105709 | 3.96583 | 1.371136 |
| C | 5.368576 | -1.98483 | -0.65557 | C | 2.211248 | -3.51926 | -2.99517 |
| C | 7.63929 | -0.32309 | -0.82132 | C | 4.703288 | -2.48168 | -3.83093 |
| O | 5.398545 | -3.87461 | 0.677263 | O | 2.070475 | -5.6826 | -2.20868 |
| C | -0.93865 | -2.12392 | -4.36028 | C | -4.67062 | -0.94599 | -3.34819 |
| C | 1.950309 | -5.70006 | -3.64798 | C | -3.41031 | -5.3923 | -3.48266 |
| C | -3.37803 | -1.46776 | -4.10749 | C | -6.71534 | 0.061337 | -2.22741 |
| C | -4.81841 | -0.13631 | -6.02976 | C | -8.45615 | 1.741046 | -3.52877 |
| C | -7.09316 | 0.968891 | -5.68372 | C | -10.514 | 2.886018 | -2.54668 |
| C | -8.33173 | 2.433873 | -7.78412 | C | -12.1329 | 4.585606 | -4.15615 |
| C | -8.53156 | 0.947713 | -3.22154 | C | -11.3887 | 2.614383 | 0.147455 |
| C | -0.06085 | -6.38751 | 1.385445 | C | -1.58091 | -5.12944 | 2.017483 |
| C | -1.67086 | -6.76488 | 3.711428 | C | -2.66501 | -5.43828 | 4.629838 |
| C | -4.1253 | -7.40089 | 3.748765 | C | -1.50077 | -5.24894 | 6.874895 |
| C | -5.55252 | -7.65016 | 6.20278 | C | -2.94921 | -5.63282 | 9.302731 |
| C | -5.67467 | -7.89459 | 1.407302 | C | 1.268273 | -4.68133 | 7.231819 |
| C | -4.18566 | 6.273284 | 3.022855 | C | 3.66193 | 6.725581 | 3.332884 |
| C | -4.89984 | 4.858418 | 5.443579 | C | 6.5072 | 7.249214 | 3.17268 |
| O | -6.18577 | 6.11213 | 1.188851 | O | 3.021615 | 5.808563 | 5.800106 |
| C | -3.58619 | 9.046839 | 3.597663 | C | 2.139132 | 9.146917 | 2.947812 |
| O | 2.097866 | 3.212366 | -2.36341 | O | 0.444808 | 2.679222 | -3.62949 |
| O | 3.386491 | 2.296575 | 4.248422 | O | 3.550548 | -1.74179 | 2.053269 |
| C | 9.65638 | -0.8472 | 0.827107 | C | 4.950595 | -0.48808 | -5.56171 |
| C | 11.83487 | 0.618488 | 0.781292 | C | 7.344988 | 0.350057 | -6.2791 |
| C | 12.03408 | 2.629049 | -0.92326 | C | 9.503932 | -0.77333 | -5.2644 |
| C | 10.04425 | 3.163925 | -2.57011 | C | 9.270919 | -2.76227 | -3.53838 |
| C | 7.853876 | 1.700685 | -2.52504 | C | 6.888226 | -3.61953 | -2.84102 |
| H | 3.689647 | -0.85293 | -4.12121 | H | -0.3276 | -1.5622 | -5.33654 |
| H | -1.29474 | -1.77722 | 3.6629 | H | -3.68507 | 0.982187 | 1.743117 |
| H | 1.937421 | -2.09648 | 3.0205 | H | -1.68392 | -0.59865 | 3.850136 |
| H | -2.51778 | -3.68126 | -0.16901 | H | -4.87773 | -3.09555 | 1.088252 |
| H | -1.40784 | 6.263949 | 0.046506 | H | 3.489124 | 5.349707 | -0.5361 |
| H | -2.96011 | 2.331648 | -1.27336 | H | -1.03817 | 5.0815 | 0.071689 |
| H | -3.82415 | 1.371805 | 1.795769 | H | -0.63753 | 4.158044 | 3.290119 |
| H | -0.02647 | -1.69842 | -6.16197 | H | -4.35275 | -0.47056 | -5.3303 |
| H | 0.434484 | -7.0447 | -4.05799 | H | -2.0041 | -6.83319 | -3.06196 |
| H | 2.859243 | -5.22019 | -5.44605 | H | -5.25826 | -6.01039 | -2.78488 |
| H | 3.345679 | -6.61434 | -2.43883 | H | -3.54696 | -5.19941 | -5.54037 |
| H | -4.35348 | -1.82902 | -2.34266 | H | -7.12269 | -0.37201 | -0.26413 |
| H | -3.9085 | 0.068474 | -7.86651 | H | -8.01679 | 2.100402 | -5.50983 |
| H | -7.17064 | 2.47029 | -9.49464 | H | -11.4218 | 4.725973 | -6.0926 |
| H | -10.1806 | 1.619839 | -8.26463 | H | -14.0893 | 3.892677 | -4.22938 |
| H | -8.69257 | 4.394112 | -7.20056 | H | -12.2227 | 6.49919 | -3.35432 |
| H | -10.5636 | 0.738528 | -3.56993 | H | -11.4653 | 4.472473 | 1.069691 |
| H | -7.94935 | -0.56817 | -1.94588 | H | -13.319 | 1.853364 | 0.207722 |
| H | -8.26859 | 2.741817 | -2.20891 | H | -10.1853 | 1.388367 | 1.289789 |
| H | 1.934414 | -6.59805 | 1.870432 | H | 0.458527 | -4.88765 | 2.050924 |
| H | -0.50134 | -7.84338 | -0.01424 | H | -1.96528 | -6.85901 | 0.949013 |
| H | -0.75013 | -6.41468 | 5.520141 | H | -4.68823 | -5.85228 | 4.657954 |
| H | -4.34791 | -7.25444 | 7.836972 | H | -4.94511 | -6.05133 | 8.956227 |
| H | -7.16825 | -6.3465 | 6.25906 | H | -2.14355 | -7.19321 | 10.41174 |
| H | -6.33628 | -9.56096 | 6.420216 | H | -2.83978 | -3.9449 | 10.50768 |
| H | -7.18646 | -6.48255 | 1.224274 | H | 2.21807 | -6.26723 | 8.179311 |
| H | -4.55801 | -7.84711 | -0.32855 | H | 2.252425 | -4.28868 | 5.465678 |
| H | -6.61153 | -9.74311 | 1.522762 | H | 1.509899 | -3.0354 | 8.475208 |
| H | -3.32498 | 4.841656 | 6.78228 | H | 7.042493 | 8.619742 | 4.625137 |
| H | -5.45534 | 2.90772 | 5.049284 | H | 7.017318 | 8.01818 | 1.319868 |
| H | -6.50165 | 5.803404 | 6.353667 | H | 7.593702 | 5.513901 | 3.469256 |
| H | -7.67831 | 6.871314 | 1.937977 | H | 3.974884 | 4.257619 | 6.049027 |
| H | -3.15715 | 10.06459 | 1.848318 | H | 2.723405 | 10.55897 | 4.34052 |
| H | -5.2093 | 9.95828 | 4.50584 | H | 2.458868 | 9.921247 | 1.056882 |
| H | -1.96498 | 9.204514 | 4.87181 | H | 0.116065 | 8.797476 | 3.187798 |
| H | 9.467209 | -2.41211 | 2.136016 | H | 3.29689 | 0.415065 | -6.35817 |
| H | 13.37768 | 0.202452 | 2.068415 | H | 7.519538 | 1.882602 | -7.63203 |
| H | 13.7361 | 3.77513 | -0.96405 | H | 11.36461 | -0.1042 | -5.81443 |
| H | 10.19481 | 4.723382 | -3.89464 | H | 10.94799 | -3.63551 | -2.74115 |
| H | 6.320654 | 2.167372 | -3.79607 | H | 6.67078 | -5.15445 | -1.50215 |
| **2D-17** | | | | **2D-18** | | | |
| Atom | X | Y | Z | Atom | X | Y | Z |
| C | -2.09254 | -1.90797 | -1.45994 | C | 2.092753 | 1.907716 | -1.46003 |
| C | -0.18953 | -1.53508 | 0.687229 | C | 0.189817 | 1.535134 | 0.687274 |
| C | 0.638151 | 1.181778 | 1.257235 | C | -0.63817 | -1.18161 | 1.257433 |
| C | -1.09736 | 3.094506 | -0.03145 | C | 1.097303 | -3.09454 | -0.03098 |
| C | -1.33257 | 2.756226 | -2.91072 | C | 1.332673 | -2.7566 | -2.9103 |
| C | -1.55549 | -0.12391 | -3.73231 | C | 1.555653 | 0.123435 | -3.73222 |
| C | 0.545401 | 1.484248 | 4.147595 | C | -0.54572 | -1.48392 | 4.147839 |
| O | 2.867091 | 1.177963 | 5.150256 | O | -2.86747 | -1.17731 | 5.150267 |
| C | 4.736194 | 0.628198 | 3.18992 | C | -4.73627 | -0.62732 | 3.189712 |
| C | 3.497016 | 1.49969 | 0.731322 | C | -3.49703 | -1.49934 | 0.731349 |
| C | -4.79838 | -1.7784 | -0.37478 | C | 4.79865 | 1.778259 | -0.37494 |
| C | -5.83664 | -4.05469 | 0.919938 | C | 5.837056 | 4.054736 | 0.919369 |
| O | -6.07707 | 0.138861 | -0.5775 | O | 6.077248 | -0.13909 | -0.57733 |
| C | 0.97131 | -0.82713 | -4.9072 | C | -0.97113 | 0.826553 | -4.90721 |
| C | -3.56729 | -0.6084 | -5.7952 | C | 3.567476 | 0.607602 | -5.79518 |
| C | 2.616224 | -2.63738 | -4.22001 | C | -2.61617 | 2.636716 | -4.22011 |
| C | 5.071921 | -2.98566 | -5.40581 | C | -5.07191 | 2.984751 | -5.40591 |
| C | 6.990259 | -4.4094 | -4.5104 | C | -6.99052 | 4.408073 | -4.51042 |
| C | 9.461188 | -4.56703 | -5.91281 | C | -9.46147 | 4.565325 | -5.91283 |
| C | 6.90062 | -5.89751 | -2.08461 | C | -6.90117 | 5.896146 | -2.08462 |
| C | -3.45606 | 4.499266 | -3.91622 | C | 3.456192 | -4.49982 | -3.91546 |
| C | -2.97722 | 7.236338 | -3.31542 | C | 2.977211 | -7.23681 | -3.31439 |
| C | -4.15735 | 8.676309 | -1.59418 | C | 4.157284 | -8.6767 | -1.59304 |
| C | -3.42028 | 11.40432 | -1.21292 | C | 3.420053 | -11.4046 | -1.2115 |
| C | -6.2524 | 7.768989 | 0.111893 | C | 6.252401 | -7.76934 | 0.112938 |
| C | 7.248621 | 1.857669 | 3.952084 | C | -7.24907 | -1.85615 | 3.951705 |
| C | 9.265892 | 1.143298 | 2.014187 | C | -9.26595 | -1.14143 | 2.013516 |
| O | 8.033923 | 0.732539 | 6.295589 | O | -8.03441 | -0.73068 | 6.295037 |
| C | 7.049 | 4.728858 | 4.274305 | C | -7.05012 | -4.72737 | 4.274115 |
| O | 0.646916 | -3.31031 | 1.897551 | O | -0.64629 | 3.310543 | 1.897576 |
| O | -1.29635 | 1.930193 | 5.421105 | O | 1.295848 | -1.93002 | 5.421558 |
| C | -4.45931 | -6.29252 | 1.295615 | C | 4.459878 | 6.292731 | 1.294608 |
| C | -5.57014 | -8.33824 | 2.529073 | C | 5.570826 | 8.338604 | 2.527718 |
| C | -8.06024 | -8.18198 | 3.384707 | C | 8.060899 | 8.18232 | 3.383432 |
| C | -9.44596 | -5.96056 | 3.019862 | C | 9.446478 | 5.960744 | 3.019015 |
| C | -8.34035 | -3.91379 | 1.802179 | C | 8.340743 | 3.913823 | 1.801688 |
| H | -1.82373 | -3.84654 | -2.11217 | H | 1.82395 | 3.846207 | -2.11249 |
| H | -0.40224 | 4.998843 | 0.367442 | H | 0.402139 | -4.99882 | 0.368096 |
| H | -2.964 | 2.956734 | 0.836756 | H | 2.963918 | -2.95673 | 0.837268 |
| H | 0.435608 | 3.452101 | -3.74245 | H | -0.43548 | -3.45256 | -3.74203 |
| H | 4.99146 | -1.42044 | 3.200826 | H | -4.99111 | 1.421371 | 3.200387 |
| H | 4.162813 | 0.422448 | -0.88917 | H | -4.16263 | -0.42236 | -0.88939 |
| H | 3.870314 | 3.501171 | 0.374338 | H | -3.87049 | -3.50086 | 0.374733 |
| H | 1.481296 | 0.383514 | -6.49714 | H | -1.48103 | -0.38419 | -6.49711 |
| H | -3.07573 | 0.383586 | -7.54099 | H | 3.630755 | 2.631187 | -6.22802 |
| H | -3.63042 | -2.63203 | -6.22785 | H | 5.448483 | 0.002632 | -5.20889 |
| H | -5.44833 | -0.00353 | -5.20892 | H | 3.075807 | -0.38451 | -7.54087 |
| H | 2.180118 | -3.87857 | -2.65011 | H | -2.18012 | 3.878051 | -2.65031 |
| H | 5.386065 | -1.92603 | -7.14467 | H | -5.38588 | 1.925155 | -7.14483 |
| H | 9.436485 | -3.44969 | -7.65239 | H | -9.43651 | 3.448195 | -7.65254 |
| H | 11.02868 | -3.89533 | -4.72786 | H | -11.0288 | 3.893178 | -4.728 |
| H | 9.906664 | -6.53384 | -6.40858 | H | -9.90735 | 6.532101 | -6.4084 |
| H | 7.232177 | -7.91364 | -2.44965 | H | -8.42344 | 5.275322 | -0.81623 |
| H | 8.422816 | -5.27681 | -0.81607 | H | -5.12051 | 5.711744 | -1.05871 |
| H | 5.119888 | -5.71294 | -1.05885 | H | -7.23291 | 7.912259 | -2.4497 |
| H | -5.25791 | 3.862256 | -3.14536 | H | 5.258006 | -3.86277 | -3.14454 |
| H | -3.5653 | 4.297194 | -5.97484 | H | 3.5656 | -4.298 | -5.9741 |
| H | -1.45207 | 8.107558 | -4.39795 | H | 1.45199 | -8.10804 | -4.39682 |
| H | -2.80716 | 11.74086 | 0.742475 | H | 2.80689 | -11.7409 | 0.743928 |
| H | -1.88595 | 11.97337 | -2.47794 | H | 1.885697 | -11.9737 | -2.47647 |
| H | -5.03567 | 12.66685 | -1.54338 | H | 5.035364 | -12.6673 | -1.54181 |
| H | -7.94579 | 8.948346 | -0.11451 | H | 7.945741 | -8.94878 | -0.1134 |
| H | -6.78371 | 5.803409 | -0.21955 | H | 6.78379 | -5.80381 | -0.21866 |
| H | -5.68083 | 7.941408 | 2.100635 | H | 5.680853 | -7.94159 | 2.101702 |
| H | 11.10534 | 1.848906 | 2.641094 | H | -11.1056 | -1.84653 | 2.640284 |
| H | 8.846001 | 1.96482 | 0.16428 | H | -8.84605 | -1.96321 | 0.163726 |
| H | 9.389169 | -0.91291 | 1.813634 | H | -9.38868 | 0.914788 | 1.812786 |
| H | 6.710535 | 1.086914 | 7.519339 | H | -6.71147 | -1.08566 | 7.519103 |
| H | 8.85057 | 5.450229 | 4.985275 | H | -8.85199 | -5.44834 | 4.984733 |
| H | 6.632151 | 5.686091 | 2.489436 | H | -6.63306 | -5.68479 | 2.489391 |
| H | 5.564746 | 5.215648 | 5.630859 | H | -5.56625 | -5.21437 | 5.631013 |
| H | -2.51151 | -6.43524 | 0.689455 | H | 2.5121 | 6.435487 | 0.688388 |
| H | -4.48742 | -10.0564 | 2.819543 | H | 4.488211 | 10.0569 | 2.817873 |
| H | -8.921 | -9.78414 | 4.335625 | H | 8.921749 | 9.784588 | 4.334091 |
| H | -11.3821 | -5.83279 | 3.686797 | H | 11.38261 | 5.832967 | 3.686005 |
| H | -9.38421 | -2.17567 | 1.507512 | H | 9.38448 | 2.175577 | 1.507372 |

**3. The details of ECD calculations for compounds 1–4.**

In general, conformational analyses were carried out *via* random searching in the Sybyl-X 2.0 using the MMFF94S force field with an energy cutoff of 5 kcal/mol.^[1]^ Subsequently, geometry optimizations and frequency analyses were implemented at the B3LYP-D3(BJ)/6-31G* level in PCM methanol using ORCA-5.0.^[2]^ All conformers used for property calculations in this work were characterized to be stable point on potential energy surface (PES) with no imaginary frequencies. The excitation energies, oscillator strengths, and rotational strengths (velocity) of the first 60 excited states were calculated using the TD-DFT methodology at the PBE0/def2-TZVP level in PCM methanol using ORCA-5.0.^[2]^ The ECD spectra were simulated by the overlapping Gaussian function (half the bandwidth at 1/e peak height, sigma = 0.30 for all).^[3]^ Gibbs free energies for conformers were determined by using thermal correction at B3LYP-D3(BJ)/6-31G* level and electronic energies evaluated at the wB97M-V/def2-TZVP level in PCM methanol using ORCA-5.0.^[2]^ To get the ﬁnal spectra, the simulated spectra of the conformers were averaged according to the boltzmann distribution theory and their relative Gibbs free energy (∆G). By comparing the experiment spectra with the calculated model molecules, the absolute configuration was determined.

Reference

[1] Sybyl Software, version X 2.0; Tripos Associates Inc.: St. Louis, MO, 2013.
[2] Neese, F. (2012) The ORCA program system, Wiley Interdiscip. Rev.: Comput. Mol. Sci., 2, 73-78.
[3] Stephens, P. J.; Harada, N. ECD cotton effect approximated by the Gaussian curve and other methods. Chirality 2010, 22, 229–233.

**ECD calculation details of 1.**

**Figure S42.** Optimized geometries of predominant conformers for **1** at the B3LYP-D3(BJ)/6-31G* level in PCM MeOH using ORCA5.0.1.

**Table S13.** Gibbs free energies^a^ and equilibrium populations^b^ of low-energy conformers of **1**.

| Conformers | ∆G(a.u.) | P(%)/100 | G(a.u.) |
| --- | --- | --- | --- |
| **1a** | 0.0 | 30.35 | -1467.543918 |
| **1b** | 0.00216 | 3.09 | -1467.54176 |
| **1c** | 0.00364 | 0.65 | -1467.540282 |
| **1d** | 0.00055 | 16.93 | -1467.543366 |
| **1e** | 0.0013 | 7.69 | -1467.542621 |
| **1f** | 0.0018 | 4.5 | -1467.542114 |
| **1g** | 0.00036 | 20.66 | -1467.543554 |
| **1h** | 0.00094 | 11.24 | -1467.54298 |
| **1i** | 0.00172 | 4.91 | -1467.542197 |

^a^wB97M-V/def2-TZVP, in a.u.
^b^From ∆G values at 298.15K.

**Table S14.** Cartesian coordinates for the low-energy reoptimized random research conformers of **1** at B3LYP-D3(BJ)/6-31G* level of theory in methanol.

| **1a** | | | | **1b** | | | |
| --- | --- | --- | --- | --- | --- | --- | --- |
| Atom | X | Y | Z | Atom | X | Y | Z |
| C | -2.00296 | 4.39723 | -5.13017 | C | -2.92858 | 4.53824 | -4.77574 |
| C | -3.31379 | -2.64486 | -0.39741 | C | -3.34524 | -2.61545 | -0.16785 |
| C | -0.56624 | 4.79051 | 10.1071 | C | 4.43104 | 1.57581 | 8.16447 |
| C | 2.45441 | 2.08473 | 7.57728 | C | 1.08934 | 4.7479 | 6.8592 |
| C | -0.23617 | 2.85973 | 8.05025 | C | 1.87441 | 2.02296 | 7.00901 |
| C | -2.2193 | 1.96228 | 6.76321 | C | 0.45854 | 0.09592 | 6.18554 |
| C | -2.13502 | 0.07563 | 4.64082 | C | -2.11012 | 0.31823 | 4.98083 |
| C | -7.47121 | 2.77311 | -7.76468 | C | -8.24302 | 2.30106 | -7.27382 |
| C | -9.81858 | -0.88402 | -5.78678 | C | -10.1011 | -1.62125 | -5.28443 |
| O | -8.38861 | 1.53842 | -5.37841 | O | -8.94769 | 0.94539 | -4.88115 |
| C | -8.04902 | 2.45835 | -3.04465 | C | -8.64798 | 1.87181 | -2.54466 |
| O | -2.84777 | 6.6891 | -0.51484 | O | -3.88635 | 6.62824 | -0.08636 |
| C | -6.66351 | 4.81356 | -2.28562 | C | -7.51202 | 4.35745 | -1.78845 |
| C | -4.01894 | 4.32018 | -1.1313 | C | -4.79528 | 4.14838 | -0.72726 |
| C | -4.20049 | 2.8483 | 1.34451 | C | -4.70821 | 2.63061 | 1.72381 |
| C | -1.84387 | 1.31899 | 2.01704 | C | -2.13378 | 1.43405 | 2.28042 |
| C | -1.28634 | -0.57872 | -0.11759 | C | -1.47174 | -0.40886 | 0.11478 |
| C | -1.02923 | 0.79623 | -2.64372 | C | -1.4298 | 1.05565 | -2.37817 |
| C | -2.31533 | 3.12832 | -3.19609 | C | -3.0166 | 3.209 | -2.85743 |
| C | 1.39463 | -1.73524 | 0.05287 | C | 1.29617 | -1.36862 | 0.18192 |
| O | 2.09678 | -2.12939 | -2.61906 | O | 2.02829 | -1.46029 | -2.52208 |
| C | 0.83433 | -0.38416 | -4.00325 | C | 0.53945 | 0.16464 | -3.80514 |
| C | 1.69591 | -0.17838 | -6.61065 | C | 1.32498 | 0.58372 | -6.41472 |
| C | 0.04675 | 0.56714 | -8.54153 | C | 3.88209 | 0.31821 | -7.06081 |
| C | 0.8901 | 0.67241 | -11.0204 | C | 4.65686 | 0.65545 | -9.5436 |
| C | 3.38057 | 0.05022 | -11.6048 | C | 2.89193 | 1.22194 | -11.4113 |
| C | 5.02697 | -0.70689 | -9.69682 | C | 0.34447 | 1.4467 | -10.7862 |
| C | 4.19152 | -0.83884 | -7.21456 | C | -0.44112 | 1.13828 | -8.3048 |
| C | 1.74802 | -4.15646 | 1.43135 | C | 1.85353 | -3.91454 | 1.24125 |
| C | 3.46956 | -4.42745 | 3.24846 | C | 3.78066 | -4.34604 | 2.79698 |
| C | 3.88202 | -6.70858 | 4.90487 | C | 4.49982 | -6.89282 | 3.8658 |
| C | 6.61445 | -7.60412 | 4.67247 | C | 4.83297 | -6.68743 | 6.73845 |
| C | 2.0425 | -8.86769 | 4.38976 | C | 6.94668 | -7.79617 | 2.62033 |
| O | 3.6326 | -5.8741 | 7.4985 | O | 2.6527 | -8.78166 | 3.28037 |
| H | -5.11937 | -1.81008 | -0.9572 | H | -3.51725 | -3.73695 | 1.56379 |
| H | -2.76589 | -3.96401 | -1.89544 | H | -5.21571 | -1.92114 | -0.70288 |
| H | -3.58609 | -3.71963 | 1.35001 | H | -2.7204 | -3.87332 | -1.6878 |
| H | -2.55105 | 5.29881 | 10.3712 | H | 4.51173 | 2.32187 | 10.0985 |
| H | 0.17345 | 4.08112 | 11.911 | H | 4.89784 | -0.43704 | 8.23039 |
| H | 0.49513 | 6.5186 | 9.66993 | H | 5.91157 | 2.5595 | 7.09233 |
| H | 2.63587 | 0.62119 | 6.13467 | H | 1.28035 | 5.65582 | 8.71414 |
| H | 3.57431 | 3.72236 | 6.96996 | H | 2.32501 | 5.79581 | 5.5622 |
| H | 3.3322 | 1.38123 | 9.31985 | H | -0.85357 | 5.00193 | 6.21098 |
| H | -4.07958 | 2.69245 | 7.26063 | H | 1.22547 | -1.79963 | 6.38723 |
| H | -0.58829 | -1.2703 | 4.92608 | H | -3.04267 | -1.53143 | 4.98327 |
| H | -3.88785 | -1.0352 | 4.66364 | H | -3.30482 | 1.53845 | 6.1601 |
| H | -6.49959 | 1.38219 | -8.95845 | H | -7.15446 | 4.0206 | -6.9519 |
| H | -9.08595 | 3.46014 | -8.87398 | H | -7.12043 | 1.0538 | -8.49376 |
| H | -6.17567 | 4.3404 | -7.4313 | H | -9.9541 | 2.77653 | -8.34838 |
| H | -10.5298 | -1.67211 | -4.01378 | H | -8.7539 | -2.8857 | -6.22932 |
| H | -11.4287 | -0.58483 | -7.06077 | H | -10.678 | -2.49991 | -3.50515 |
| H | -8.60652 | -2.30422 | -6.69269 | H | -11.7621 | -1.49819 | -6.52149 |
| H | -8.87736 | 1.3746 | -1.49878 | H | -9.30534 | 0.68499 | -0.9922 |
| H | -2.36584 | 7.43811 | -2.11433 | H | -3.5272 | 7.45454 | -1.68012 |
| H | -7.74969 | 5.8472 | -0.85013 | H | -8.65923 | 5.23886 | -0.29978 |
| H | -6.40208 | 6.08469 | -3.89496 | H | -7.44837 | 5.67608 | -3.37981 |
| H | -5.8251 | 1.56694 | 1.28435 | H | -6.16551 | 1.16239 | 1.71809 |
| H | -4.58955 | 4.23415 | 2.8338 | H | -5.20744 | 3.94687 | 3.24548 |
| H | -0.2312 | 2.62942 | 2.11073 | H | -0.71249 | 2.94766 | 2.21098 |
| H | 2.70551 | -0.29041 | 0.7788 | H | 2.51072 | 0.07246 | 1.05761 |
| H | -1.88267 | 1.06443 | -8.0794 | H | 5.24657 | -0.13675 | -5.60039 |
| H | -0.40225 | 1.24729 | -12.5072 | H | 6.64328 | 0.4721 | -10.0251 |
| H | 4.03573 | 0.14911 | -13.5478 | H | 3.49996 | 1.48028 | -13.355 |
| H | 6.9682 | -1.19773 | -10.1461 | H | -1.03841 | 1.86967 | -12.2423 |
| H | 5.46315 | -1.43652 | -5.72223 | H | -2.41667 | 1.32796 | -7.8103 |
| H | 0.52101 | -5.71468 | 0.89464 | H | 0.70123 | -5.48594 | 0.59038 |
| H | 4.69878 | -2.83793 | 3.71847 | H | 4.978 | -2.77157 | 3.38517 |
| H | 6.98978 | -8.2879 | 2.75854 | H | 5.36391 | -8.52992 | 7.51252 |
| H | 7.91568 | -6.05469 | 5.09562 | H | 6.30649 | -5.31562 | 7.21918 |
| H | 6.96227 | -9.13454 | 6.01944 | H | 3.07259 | -6.07096 | 7.64181 |
| H | 2.26374 | -9.60316 | 2.46842 | H | 8.4646 | -6.42445 | 2.91781 |
| H | 0.07963 | -8.2515 | 4.62874 | H | 6.65202 | -8.03039 | 0.58889 |
| H | 2.40571 | -10.4017 | 5.72566 | H | 7.51565 | -9.61155 | 3.43179 |
| H | 1.95486 | -5.18361 | 7.69614 | H | 1.10332 | -8.32316 | 4.12734 |
| **1c** | | | | **1d** | | | |
| Atom | X | Y | Z | Atom | X | Y | Z |
| C | -2.74807 | 2.44665 | -5.87662 | C | -1.92747 | 3.77344 | -5.72511 |
| C | -3.46518 | -2.58553 | -0.09295 | C | -3.19804 | -2.5758 | -0.04072 |
| C | 1.00309 | -1.94197 | 9.94413 | C | -0.59442 | 5.10471 | 10.425 |
| C | -3.15676 | -3.71023 | 8.42321 | C | -0.94389 | 0.33268 | 10.165 |
| C | -1.21276 | -1.65393 | 8.18868 | C | -1.07441 | 2.82414 | 8.80336 |
| C | -1.32649 | 0.27141 | 6.55229 | C | -1.55594 | 3.03083 | 6.33247 |
| C | -3.39392 | 0.88449 | 4.71578 | C | -1.9729 | 0.87064 | 4.52332 |
| C | -5.95208 | 11.103 | -2.53163 | C | -7.39169 | 1.99881 | -8.20495 |
| C | -4.94078 | 11.3326 | -7.19704 | C | -9.69606 | -1.465 | -5.86128 |
| O | -5.54316 | 9.6848 | -4.9602 | O | -8.28374 | 0.99701 | -5.70261 |
| C | -5.71739 | 7.16793 | -5.21696 | C | -7.94105 | 2.14664 | -3.47338 |
| O | -2.12786 | 6.14397 | -1.30733 | O | -2.75752 | 6.65054 | -1.431 |
| C | -6.30723 | 5.30513 | -3.15967 | C | -6.57866 | 4.58103 | -2.96411 |
| C | -3.94852 | 4.19614 | -1.83901 | C | -3.94034 | 4.22905 | -1.75006 |
| C | -4.65394 | 3.06289 | 0.71129 | C | -4.12677 | 3.08067 | 0.88769 |
| C | -2.49262 | 1.66763 | 2.05775 | C | -1.74028 | 1.70053 | 1.75142 |
| C | -1.50928 | -0.47496 | 0.32792 | C | -1.18534 | -0.47918 | -0.08988 |
| C | -0.81398 | 0.61923 | -2.254 | C | -0.95368 | 0.52958 | -2.78476 |
| C | -2.52153 | 2.32009 | -3.5734 | C | -2.24004 | 2.7693 | -3.64148 |
| C | 1.09567 | -1.61873 | 1.10567 | C | 1.50531 | -1.58459 | 0.23227 |
| O | 2.46481 | -1.93652 | -1.26377 | O | 2.21484 | -2.3213 | -2.36404 |
| C | 1.39973 | -0.41414 | -3.0381 | C | 0.92371 | -0.80489 | -3.97164 |
| C | 2.89766 | -0.2011 | -5.35406 | C | 1.76844 | -0.95205 | -6.58777 |
| C | 2.80085 | 1.95027 | -6.88904 | C | 0.09372 | -0.50346 | -8.58723 |
| C | 4.29424 | 2.10317 | -9.03799 | C | 0.91653 | -0.73151 | -11.0646 |
| C | 5.88883 | 0.11631 | -9.70068 | C | 3.41156 | -1.39629 | -11.5798 |
| C | 6.00645 | -2.02196 | -8.17569 | C | 5.08333 | -1.85915 | -9.60105 |
| C | 4.53908 | -2.17606 | -6.00568 | C | 4.26881 | -1.65626 | -7.11646 |
| C | 1.08431 | -4.05808 | 2.51175 | C | 1.87073 | -3.81208 | 1.90562 |
| C | 1.79076 | -6.28056 | 1.57914 | C | 3.46998 | -3.79087 | 3.85047 |
| C | 1.73526 | -8.72697 | 3.05456 | C | 3.89491 | -5.87358 | 5.74856 |
| C | 4.20078 | -10.1861 | 2.62865 | C | 6.66419 | -6.68347 | 5.69258 |
| C | -0.54227 | -10.3039 | 2.23561 | C | 2.14983 | -8.14159 | 5.39642 |
| O | 1.38192 | -8.28481 | 5.7105 | O | 3.5259 | -4.80331 | 8.23797 |
| H | -4.00157 | -3.5046 | 1.68181 | H | -5.01056 | -1.85602 | -0.72337 |
| H | -5.16597 | -1.85324 | -1.0121 | H | -2.6447 | -4.11514 | -1.30916 |
| H | -2.66877 | -4.0382 | -1.33013 | H | -3.45969 | -3.35434 | 1.85989 |
| H | 2.41655 | -0.46189 | 9.65942 | H | 1.25999 | 4.97399 | 11.3471 |
| H | 0.3899 | -1.88691 | 11.925 | H | -0.64914 | 6.85103 | 9.32264 |
| H | 1.91768 | -3.78684 | 9.67398 | H | -2.0015 | 5.24703 | 11.943 |
| H | -2.30859 | -5.55076 | 7.96979 | H | -1.632 | -1.23621 | 9.00907 |
| H | -3.83914 | -3.83137 | 10.3778 | H | 1.00416 | -0.07674 | 10.7574 |
| H | -4.79288 | -3.43372 | 7.19871 | H | -2.08671 | 0.38543 | 11.8938 |
| H | 0.22612 | 1.63166 | 6.58272 | H | -1.61457 | 4.92324 | 5.51769 |
| H | -4.77392 | -0.64982 | 4.57486 | H | -0.6038 | -0.63641 | 4.90663 |
| H | -4.44649 | 2.51092 | 5.47739 | H | -3.85411 | 0.02992 | 4.82031 |
| H | -7.56422 | 12.3959 | -2.72097 | H | -9.01831 | 2.57393 | -9.35952 |
| H | -6.27786 | 9.87157 | -0.91104 | H | -6.09578 | 3.59176 | -8.03509 |
| H | -4.30265 | 12.2835 | -2.09887 | H | -6.4271 | 0.50178 | -9.26881 |
| H | -6.46819 | 12.6963 | -7.53295 | H | -11.3153 | -1.30283 | -7.14827 |
| H | -3.2239 | 12.4469 | -6.85805 | H | -8.4782 | -2.95852 | -6.63103 |
| H | -4.6696 | 10.2216 | -8.91685 | H | -10.3917 | -2.07962 | -4.01502 |
| H | -5.34955 | 6.35641 | -7.06773 | H | -8.74859 | 1.21411 | -1.8217 |
| H | -2.07384 | 7.20746 | -2.8018 | H | -2.26157 | 7.1902 | -3.10847 |
| H | -7.38405 | 3.72227 | -3.9533 | H | -7.68174 | 5.75631 | -1.6559 |
| H | -7.48474 | 6.17487 | -1.69532 | H | -6.31287 | 5.67349 | -4.69866 |
| H | -6.283 | 1.79984 | 0.46991 | H | -5.71965 | 1.76213 | 0.97317 |
| H | -5.29463 | 4.63118 | 1.90849 | H | -4.57668 | 4.62456 | 2.19485 |
| H | -0.93289 | 3.02349 | 2.28143 | H | -0.14304 | 3.02622 | 1.60243 |
| H | 2.14743 | -0.18717 | 2.1963 | H | 2.80663 | -0.05356 | 0.77265 |
| H | 1.56318 | 3.50172 | -6.39385 | H | -1.83949 | 0.02588 | -8.17758 |
| H | 4.21159 | 3.78825 | -10.2065 | H | -0.39465 | -0.38581 | -12.605 |
| H | 7.04281 | 0.24164 | -11.3937 | H | 4.05054 | -1.5598 | -13.5238 |
| H | 7.25404 | -3.57439 | -8.67156 | H | 7.02798 | -2.38141 | -9.99693 |
| H | 4.65096 | -3.83244 | -4.80369 | H | 5.55998 | -2.02219 | -5.56713 |
| H | 0.42076 | -3.92656 | 4.44921 | H | 0.75526 | -5.48161 | 1.46847 |
| H | 2.43705 | -6.4221 | -0.37005 | H | 4.57833 | -2.091 | 4.22637 |
| H | 4.45808 | -10.6342 | 0.62461 | H | 7.89415 | -5.05039 | 5.99757 |
| H | 5.82988 | -9.06103 | 3.23836 | H | 7.01887 | -8.05817 | 7.19677 |
| H | 4.1686 | -11.9514 | 3.7053 | H | 7.12891 | -7.53813 | 3.86912 |
| H | -0.47526 | -10.6736 | 0.20264 | H | 0.16033 | -7.57377 | 5.49396 |
| H | -2.28673 | -9.28053 | 2.66226 | H | 2.50813 | -9.51086 | 6.90184 |
| H | -0.55599 | -12.1101 | 3.24452 | H | 2.47161 | -9.07077 | 3.57579 |
| H | 2.81566 | -7.31434 | 6.2881 | H | 1.82504 | -4.14618 | 8.3096 |
| **1e** | | | | **1f** | | | |
| Atom | X | Y | Z | Atom | X | Y | Z |
| C | -1.31145 | 3.56973 | -6.03395 | C | 1.1859 | 5.79362 | -3.88807 |
| C | -3.1896 | -1.76225 | 0.27357 | C | -3.14842 | -1.29967 | -1.65038 |
| C | 0.38658 | 8.21343 | 8.11412 | C | -0.35958 | -3.34195 | 9.21863 |
| C | 3.31231 | 5.0313 | 6.06887 | C | -4.36907 | -4.35392 | 6.84892 |
| C | 0.64157 | 5.76778 | 6.69361 | C | -2.24225 | -2.51654 | 7.2579 |
| C | -1.38784 | 4.41386 | 6.03114 | C | -1.95178 | -0.33602 | 6.01128 |
| C | -1.36606 | 1.99158 | 4.5401 | C | -3.62868 | 0.79639 | 4.02504 |
| C | -10.3762 | 3.49825 | -0.19975 | C | -9.75861 | 3.09331 | -4.58377 |
| C | -10.475 | -0.32628 | -3.05211 | C | -11.5932 | 7.09453 | -2.74694 |
| O | -9.20797 | 2.13303 | -2.39947 | O | -9.25533 | 5.69438 | -3.55756 |
| C | -7.20524 | 2.9512 | -3.71201 | C | -6.97077 | 6.7588 | -3.33848 |
| O | -1.59274 | 7.05225 | -2.08828 | O | -2.13998 | 7.90345 | -0.72924 |
| C | -5.6862 | 5.30339 | -3.29888 | C | -4.46528 | 5.64636 | -4.0118 |
| C | -3.07383 | 4.77509 | -2.09557 | C | -2.68692 | 5.43478 | -1.67544 |
| C | -3.35289 | 3.9914 | 0.66455 | C | -3.93112 | 3.93486 | 0.47449 |
| C | -1.13753 | 2.43944 | 1.67067 | C | -2.20653 | 2.02404 | 1.79967 |
| C | -0.8903 | 0.00467 | 0.09145 | C | -1.11786 | 0.19825 | -0.20889 |
| C | -0.47396 | 0.70606 | -2.67598 | C | 0.44129 | 1.75291 | -2.07657 |
| C | -1.59631 | 2.91182 | -3.81372 | C | -0.17522 | 4.33302 | -2.67891 |
| C | 1.60492 | -1.45646 | 0.62129 | C | 0.97442 | -1.61284 | 0.80088 |
| O | 2.42101 | -2.34334 | -1.8634 | O | 2.89606 | -1.63738 | -1.18787 |
| C | 1.34396 | -0.85858 | -3.64756 | C | 2.62018 | 0.46373 | -2.60912 |
| C | 2.36021 | -1.26094 | -6.17911 | C | 4.66583 | 0.87562 | -4.41008 |
| C | 4.85987 | -2.10613 | -6.44736 | C | 4.24488 | 2.13202 | -6.70024 |
| C | 5.85528 | -2.55275 | -8.83131 | C | 6.20595 | 2.42924 | -8.41316 |
| C | 4.36366 | -2.19996 | -10.9707 | C | 8.60496 | 1.49449 | -7.86349 |
| C | 1.86955 | -1.40055 | -10.7134 | C | 9.0338 | 0.23188 | -5.59559 |
| C | 0.86706 | -0.92395 | -8.33765 | C | 7.07554 | -0.09417 | -3.88196 |
| C | 1.49835 | -3.62109 | 2.41634 | C | 0.22933 | -4.25687 | 1.40824 |
| C | 1.80263 | -6.03129 | 1.78395 | C | 1.10624 | -6.31588 | 0.26747 |
| C | 1.64709 | -8.215 | 3.59625 | C | 0.33328 | -8.98186 | 0.94107 |
| C | 4.17944 | -9.62652 | 3.64213 | C | 2.48368 | -10.2982 | 2.35013 |
| C | -0.49698 | -9.98591 | 2.771 | C | -0.34686 | -10.4322 | -1.47372 |
| O | 1.10945 | -7.204 | 6.05358 | O | -1.77062 | -9.01596 | 2.65404 |
| H | -4.87295 | -0.85608 | -0.515 | H | -4.33774 | -2.41663 | -0.37573 |
| H | -2.8287 | -3.4799 | -0.82197 | H | -4.36614 | -0.04261 | -2.74582 |
| H | -3.57507 | -2.33562 | 2.22401 | H | -2.23893 | -2.60614 | -2.97028 |
| H | 1.22701 | 9.77643 | 7.03914 | H | 0.52796 | -5.13174 | 8.65495 |
| H | -1.59054 | 8.67762 | 8.49312 | H | 1.12733 | -1.93579 | 9.50292 |
| H | 1.39713 | 8.12728 | 9.92412 | H | -1.28709 | -3.69027 | 11.0411 |
| H | 3.4614 | 3.14532 | 5.24343 | H | -5.52618 | -4.49826 | 8.56484 |
| H | 4.13192 | 6.37941 | 4.72108 | H | -5.62199 | -3.82634 | 5.29508 |
| H | 4.5008 | 5.09161 | 7.767 | H | -3.61892 | -6.25167 | 6.47413 |
| H | -3.23726 | 5.15051 | 6.55731 | H | -0.3342 | 0.83593 | 6.53194 |
| H | 0.20262 | 0.79456 | 5.17174 | H | -5.01747 | -0.56645 | 3.31798 |
| H | -3.10406 | 0.93337 | 4.93732 | H | -4.74705 | 2.30627 | 4.91975 |
| H | -9.44392 | 5.28211 | 0.24855 | H | -8.06384 | 2.13167 | -5.26037 |
| H | -12.3791 | 3.88803 | -0.57306 | H | -10.6368 | 1.90179 | -3.12853 |
| H | -10.3189 | 2.30395 | 1.49728 | H | -11.1082 | 3.18619 | -6.15609 |
| H | -10.4366 | -1.62578 | -1.43359 | H | -12.9049 | 7.32724 | -4.33768 |
| H | -12.4743 | -0.02706 | -3.5189 | H | -12.6194 | 6.03505 | -1.28682 |
| H | -9.56629 | -1.25945 | -4.65571 | H | -11.1433 | 8.96529 | -1.99571 |
| H | -6.54227 | 1.76052 | -5.25976 | H | -6.86813 | 8.65239 | -2.5364 |
| H | -1.12514 | 7.35345 | -3.83171 | H | -0.82124 | 8.55282 | -1.82842 |
| H | -6.65263 | 6.67835 | -2.09288 | H | -3.48233 | 6.85912 | -5.38147 |
| H | -5.33083 | 6.20926 | -5.12976 | H | -4.65094 | 3.77927 | -4.88996 |
| H | -5.10914 | 2.92073 | 0.90123 | H | -5.61894 | 2.96029 | -0.2301 |
| H | -3.54772 | 5.73508 | 1.76572 | H | -4.61603 | 5.31772 | 1.85265 |
| H | 0.59585 | 3.54477 | 1.35 | H | -0.58176 | 3.06509 | 2.58262 |
| H | 3.04869 | -0.08433 | 1.24488 | H | 1.87311 | -0.74021 | 2.465 |
| H | 6.01117 | -2.38696 | -4.77482 | H | 2.3828 | 2.86742 | -7.12233 |
| H | 7.80012 | -3.17927 | -9.02265 | H | 5.86297 | 3.39988 | -10.1881 |
| H | 5.14217 | -2.55473 | -12.8369 | H | 10.1376 | 1.74523 | -9.20599 |
| H | 0.69646 | -1.14182 | -12.377 | H | 10.9006 | -0.50163 | -5.16172 |
| H | -1.06562 | -0.28602 | -8.13769 | H | 7.39758 | -1.08137 | -2.11458 |
| H | 1.13374 | -3.13667 | 4.37824 | H | -1.09838 | -4.43694 | 2.96365 |
| H | 2.18298 | -6.54438 | -0.17093 | H | 2.48919 | -6.14394 | -1.24695 |
| H | 5.67802 | -8.36361 | 4.29774 | H | 4.20413 | -10.3078 | 1.20402 |
| H | 4.07194 | -11.2595 | 4.91511 | H | 2.87449 | -9.30173 | 4.11791 |
| H | 4.6756 | -10.3222 | 1.75761 | H | 1.9559 | -12.2515 | 2.78102 |
| H | -2.29817 | -8.97263 | 2.80531 | H | -1.91089 | -9.50447 | -2.46685 |
| H | -0.61972 | -11.6151 | 4.04736 | H | -0.91188 | -12.3634 | -0.9968 |
| H | -0.17473 | -10.7017 | 0.8564 | H | 1.26532 | -10.5099 | -2.76863 |
| H | 1.02987 | -8.601 | 7.21895 | H | -3.18692 | -8.25405 | 1.79126 |
| **1g** | | | | **1h** | | | |
| Atom | X | Y | Z | Atom | X | Y | Z |
| C | -1.69663 | 3.21962 | -6.2749 | C | -0.01503 | 5.9724 | -3.78342 |
| C | -2.46176 | -1.59002 | 0.61801 | C | -3.7195 | -0.8708 | -0.4133 |
| C | -0.33487 | 8.62552 | 8.03198 | C | 1.34435 | -3.84905 | 9.21082 |
| C | -3.7514 | 5.35709 | 7.37119 | C | -3.21587 | -4.30007 | 7.90154 |
| C | -1.06058 | 6.12768 | 6.88733 | C | -0.87014 | -2.69976 | 7.85299 |
| C | 0.59748 | 4.7854 | 5.53057 | C | -0.66818 | -0.46423 | 6.68905 |
| C | 0.17241 | 2.30369 | 4.22939 | C | -2.65645 | 0.96018 | 5.25593 |
| C | -9.32343 | 4.01163 | 1.26937 | C | -5.1569 | 5.72047 | -7.32114 |
| C | -10.18 | 0.01264 | -1.19376 | C | -8.64886 | 2.69113 | -6.12788 |
| O | -8.72361 | 2.44731 | -1.0241 | O | -6.7773 | 4.64453 | -5.25453 |
| C | -7.03069 | 3.07587 | -2.79716 | C | -6.65891 | 5.31561 | -2.81668 |
| O | -1.04793 | 6.96605 | -2.60344 | O | -1.12625 | 7.90316 | 0.93292 |
| C | -5.36536 | 5.36314 | -2.88562 | C | -4.89668 | 7.17783 | -1.60457 |
| C | -2.59208 | 4.77327 | -2.16798 | C | -2.66037 | 5.95144 | -0.17309 |
| C | -2.36823 | 4.17591 | 0.64098 | C | -3.55736 | 4.31952 | 2.03028 |
| C | -0.05929 | 2.59126 | 1.33819 | C | -1.71962 | 2.22139 | 2.80378 |
| C | -0.18005 | 0.06001 | -0.09997 | C | -1.30284 | 0.45843 | 0.50632 |
| C | -0.26798 | 0.57555 | -2.93705 | C | -0.22764 | 2.01926 | -1.6736 |
| C | -1.54413 | 2.73503 | -3.99777 | C | -0.90516 | 4.61787 | -2.10342 |
| C | 2.32117 | -1.47907 | 0.06105 | C | 0.87893 | -1.49055 | 0.86008 |
| O | 2.64947 | -2.5254 | -2.47295 | O | 2.22065 | -1.49064 | -1.55729 |
| C | 1.297 | -1.11086 | -4.12102 | C | 1.66124 | 0.66209 | -2.80699 |
| C | 1.81918 | -1.70108 | -6.7642 | C | 3.17879 | 1.0505 | -5.07723 |
| C | 4.21267 | -2.63107 | -7.42823 | C | 5.63139 | 0.0489 | -5.18083 |
| C | 4.74221 | -3.25888 | -9.91781 | C | 7.08944 | 0.34683 | -7.34012 |
| C | 2.88565 | -3.00522 | -11.7656 | C | 6.10767 | 1.60826 | -9.43055 |
| C | 0.49526 | -2.12134 | -11.1103 | C | 3.65854 | 2.56947 | -9.3523 |
| C | -0.04234 | -1.46322 | -8.62942 | C | 2.19917 | 2.30397 | -7.19061 |
| C | 2.46376 | -3.54595 | 1.96664 | C | 0.16894 | -4.13159 | 1.52609 |
| C | 2.5563 | -5.9964 | 1.4297 | C | 0.62253 | -6.16248 | 0.11957 |
| C | 2.66429 | -8.0765 | 3.36338 | C | -0.10583 | -8.82412 | 0.85684 |
| C | 5.11656 | -9.58679 | 3.02909 | C | 2.25836 | -10.322 | 1.57058 |
| C | 0.3508 | -9.79765 | 3.048 | C | -1.49426 | -10.093 | -1.34784 |
| O | 2.61927 | -6.91955 | 5.81525 | O | -1.67587 | -8.86159 | 3.06894 |
| H | -4.23261 | -0.65131 | 0.10947 | H | -5.0801 | 0.49571 | -1.15526 |
| H | -2.37882 | -3.37673 | -0.42231 | H | -3.24418 | -2.16814 | -1.95253 |
| H | -2.48605 | -2.04635 | 2.63615 | H | -4.61842 | -1.9808 | 1.08636 |
| H | 1.6259 | 9.12292 | 7.61339 | H | 1.86011 | -5.65784 | 8.33203 |
| H | -1.55724 | 10.1433 | 7.31958 | H | 2.99748 | -2.61022 | 9.17883 |
| H | -0.57429 | 8.5911 | 10.0926 | H | 0.87352 | -4.26345 | 11.188 |
| H | -4.14448 | 5.31547 | 9.40717 | H | -2.77912 | -6.22564 | 7.26472 |
| H | -5.04958 | 6.75178 | 6.54751 | H | -3.92767 | -4.46412 | 9.84336 |
| H | -4.22752 | 3.51251 | 6.58137 | H | -4.74681 | -3.54832 | 6.73801 |
| H | 2.47928 | 5.58881 | 5.27216 | H | 1.14398 | 0.51319 | 6.83527 |
| H | 1.78229 | 1.0661 | 4.65712 | H | -4.31784 | -0.21135 | 4.86489 |
| H | -1.51097 | 1.35138 | 4.96418 | H | -3.34352 | 2.49681 | 6.48003 |
| H | -8.29245 | 5.79628 | 1.33139 | H | -4.2559 | 4.1859 | -8.38847 |
| H | -11.3536 | 4.42881 | 1.35002 | H | -6.3356 | 6.75757 | -8.67872 |
| H | -8.87368 | 2.95525 | 2.99985 | H | -3.67042 | 6.96824 | -6.63043 |
| H | -12.2233 | 0.37001 | -1.23133 | H | -9.84715 | 2.02226 | -4.58275 |
| H | -9.67718 | -1.06857 | -2.88106 | H | -9.87201 | 3.45308 | -7.62027 |
| H | -9.8197 | -1.17419 | 0.4711 | H | -7.66689 | 1.05625 | -6.9463 |
| H | -6.76616 | 1.7552 | -4.3588 | H | -7.99638 | 4.39858 | -1.54335 |
| H | -0.9228 | 7.1416 | -4.42062 | H | -0.22675 | 8.68135 | -0.45865 |
| H | -6.01538 | 6.87478 | -1.63109 | H | -5.92436 | 8.32329 | -0.21186 |
| H | -5.34201 | 6.1131 | -4.81805 | H | -4.09108 | 8.4781 | -2.99645 |
| H | -4.08052 | 3.19646 | 1.26978 | H | -5.39793 | 3.47902 | 1.59742 |
| H | -2.30632 | 5.98563 | 1.64678 | H | -3.85896 | 5.60411 | 3.62795 |
| H | 1.63214 | 3.59316 | 0.65358 | H | 0.11846 | 3.10961 | 3.20975 |
| H | 3.90154 | -0.14645 | 0.34408 | H | 2.22337 | -0.75183 | 2.26909 |
| H | 5.64951 | -2.83353 | -5.98033 | H | 6.38404 | -0.93998 | -3.5504 |
| H | 6.60832 | -3.94975 | -10.4199 | H | 8.99681 | -0.40833 | -7.39425 |
| H | 3.29973 | -3.50222 | -13.714 | H | 7.24799 | 1.83648 | -11.1223 |
| H | -0.96174 | -1.93887 | -12.5438 | H | 2.88141 | 3.53798 | -10.9861 |
| H | -1.89388 | -0.75891 | -8.12174 | H | 0.30237 | 3.06511 | -7.11907 |
| H | 2.48685 | -2.94785 | 3.93081 | H | -0.74074 | -4.33302 | 3.35469 |
| H | 2.54917 | -6.62303 | -0.52908 | H | 1.59195 | -5.97079 | -1.68565 |
| H | 6.74857 | -8.35487 | 3.32854 | H | 3.60355 | -10.3384 | 0.00104 |
| H | 5.19558 | -11.1497 | 4.38953 | H | 3.16768 | -9.45394 | 3.21079 |
| H | 5.23657 | -10.3951 | 1.12814 | H | 1.74865 | -12.2719 | 2.03696 |
| H | -1.38075 | -8.71031 | 3.35009 | H | -3.20419 | -9.03157 | -1.84076 |
| H | 0.41616 | -11.3496 | 4.4207 | H | -2.03271 | -12.0192 | -0.82177 |
| H | 0.29216 | -10.6263 | 1.15278 | H | -0.29495 | -10.1738 | -3.03238 |
| H | 2.73904 | -8.24949 | 7.05335 | H | -3.22067 | -7.98293 | 2.65315 |
| **1i** | | | |  |  |  |  |
| Atom | X | Y | Z |  |  |  |  |
| C | 0.99271 | 5.67824 | -4.16726 |  |  |  |  |
| C | -3.08652 | -1.43365 | -1.51207 |  |  |  |  |
| C | -0.38455 | -2.96633 | 9.3449 |  |  |  |  |
| C | -4.4119 | -4.04766 | 7.03478 |  |  |  |  |
| C | -2.28399 | -2.19825 | 7.37693 |  |  |  |  |
| C | -1.99423 | -0.06668 | 6.04782 |  |  |  |  |
| C | -3.6757 | 0.97034 | 4.01488 |  |  |  |  |
| C | -9.86383 | 2.63984 | -4.7295 |  |  |  |  |
| C | -11.8129 | 6.66294 | -3.06553 |  |  |  |  |
| O | -9.43507 | 5.29444 | -3.81173 |  |  |  |  |
| C | -7.18107 | 6.42911 | -3.63269 |  |  |  |  |
| O | -2.38269 | 7.84799 | -1.09716 |  |  |  |  |
| C | -4.64421 | 5.3598 | -4.25591 |  |  |  |  |
| C | -2.85725 | 5.31921 | -1.91746 |  |  |  |  |
| C | -4.05133 | 3.89194 | 0.30669 |  |  |  |  |
| C | -2.26724 | 2.11514 | 1.73809 |  |  |  |  |
| C | -1.11188 | 0.21922 | -0.16655 |  |  |  |  |
| C | 0.38736 | 1.72615 | -2.12305 |  |  |  |  |
| C | -0.31647 | 4.24488 | -2.86989 |  |  |  |  |
| C | 1.04887 | -1.46173 | 0.93176 |  |  |  |  |
| O | 2.96762 | -1.51696 | -1.06253 |  |  |  |  |
| C | 2.61075 | 0.49083 | -2.5928 |  |  |  |  |
| C | 4.63815 | 0.87983 | -4.42066 |  |  |  |  |
| C | 4.16671 | 1.98057 | -6.77971 |  |  |  |  |
| C | 6.11389 | 2.25506 | -8.51228 |  |  |  |  |
| C | 8.54927 | 1.45307 | -7.91446 |  |  |  |  |
| C | 9.02912 | 0.3456 | -5.57677 |  |  |  |  |
| C | 7.08534 | 0.04155 | -3.84243 |  |  |  |  |
| C | 0.41323 | -4.10148 | 1.67422 |  |  |  |  |
| C | 1.27429 | -6.16226 | 0.52809 |  |  |  |  |
| C | 0.64629 | -8.82821 | 1.28613 |  |  |  |  |
| C | 3.08554 | -10.2487 | 1.94663 |  |  |  |  |
| C | -0.74554 | -10.1433 | -0.8917 |  |  |  |  |
| O | -0.96758 | -8.71694 | 3.46742 |  |  |  |  |
| H | -4.38368 | -0.2848 | -2.63548 |  |  |  |  |
| H | -2.13298 | -2.74547 | -2.79509 |  |  |  |  |
| H | -4.18945 | -2.56325 | -0.17341 |  |  |  |  |
| H | 0.48581 | -4.77874 | 8.8266 |  |  |  |  |
| H | 1.11489 | -1.56151 | 9.5641 |  |  |  |  |
| H | -1.29222 | -3.24726 | 11.1888 |  |  |  |  |
| H | -5.76004 | -3.49015 | 5.57666 |  |  |  |  |
| H | -3.66267 | -5.9179 | 6.54181 |  |  |  |  |
| H | -5.45621 | -4.26484 | 8.8139 |  |  |  |  |
| H | -0.37434 | 1.12415 | 6.51559 |  |  |  |  |
| H | -5.04269 | -0.43876 | 3.36074 |  |  |  |  |
| H | -4.81053 | 2.50728 | 4.83995 |  |  |  |  |
| H | -10.7014 | 1.48273 | -3.22339 |  |  |  |  |
| H | -11.2201 | 2.62804 | -6.29878 |  |  |  |  |
| H | -8.14303 | 1.70191 | -5.37297 |  |  |  |  |
| H | -13.1204 | 6.80116 | -4.67074 |  |  |  |  |
| H | -12.8197 | 5.63282 | -1.57132 |  |  |  |  |
| H | -11.4153 | 8.5717 | -2.38354 |  |  |  |  |
| H | -7.13194 | 8.35665 | -2.91044 |  |  |  |  |
| H | -1.09264 | 8.4826 | -2.23797 |  |  |  |  |
| H | -3.70087 | 6.53179 | -5.68784 |  |  |  |  |
| H | -4.77416 | 3.44592 | -5.03794 |  |  |  |  |
| H | -5.69774 | 2.81773 | -0.34915 |  |  |  |  |
| H | -4.79452 | 5.32058 | 1.60582 |  |  |  |  |
| H | -0.6819 | 3.24939 | 2.4713 |  |  |  |  |
| H | 1.92063 | -0.47204 | 2.54346 |  |  |  |  |
| H | 2.27644 | 2.61508 | -7.2387 |  |  |  |  |
| H | 5.73149 | 3.10459 | -10.3405 |  |  |  |  |
| H | 10.0704 | 1.68661 | -9.2731 |  |  |  |  |
| H | 10.9244 | -0.28376 | -5.10416 |  |  |  |  |
| H | 7.44738 | -0.82607 | -2.02082 |  |  |  |  |
| H | -0.82218 | -4.27259 | 3.30059 |  |  |  |  |
| H | 2.53385 | -5.99686 | -1.08959 |  |  |  |  |
| H | 4.38435 | -10.2769 | 0.33617 |  |  |  |  |
| H | 4.0273 | -9.32995 | 3.54034 |  |  |  |  |
| H | 2.66292 | -12.2132 | 2.45765 |  |  |  |  |
| H | -2.51486 | -9.15397 | -1.29533 |  |  |  |  |
| H | -1.17386 | -12.1102 | -0.39444 |  |  |  |  |
| H | 0.40997 | -10.1609 | -2.60816 |  |  |  |  |
| H | -1.36455 | -10.4317 | 3.93634 |  |  |  |  |

**ECD calculation details of 2.**

**Figure S43.** Optimized geometries of predominant conformers for **3** at the B3LYP-D3(BJ)/6-31G* level in PCM MeOH using ORCA5.0.1.

**Table S15.** Gibbs free energies^a^ and equilibrium populations^b^ of low-energy conformers of **2**.

| Conformers | ∆G(a.u.) | P(%)/100 | G(a.u.) |
| --- | --- | --- | --- |
| **2a** | 0.0 | 55.78 | -1580.930357 |
| **2b** | 0.00763 | 0.02 | -1580.92273 |
| **2c** | 0.00352 | 1.34 | -1580.926837 |
| **2d** | 0.00071 | 26.31 | -1580.929647 |
| **2e** | 0.00152 | 11.2 | -1580.928841 |
| **2f** | 0.00382 | 0.97 | -1580.926536 |
| **2g** | 0.0024 | 4.38 | -1580.927954 |

^a^wB97M-V/def2-TZVP, in a.u.
^b^From ∆G values at 298.15K.

**Table S16.** Cartesian coordinates for the low-energy reoptimized random research conformers of **2** at B3LYP-D3(BJ)/6-31G* level of theory in MeOH.

| **2a** | | | | **2b** | | | |
| --- | --- | --- | --- | --- | --- | --- | --- |
| Atom | X | Y | Z | Atom | X | Y | Z |
| C | -1.40349 | 0.4001 | 0.83706 | C | -0.89289 | -1.56153 | -0.50837 |
| C | -0.20776 | -1.20787 | 2.8864 | C | 0.0155 | -3.4385 | 1.42343 |
| C | 1.86874 | -3.03473 | 1.96737 | C | 2.91662 | -3.72821 | 1.52935 |
| C | 3.76243 | -1.52956 | 0.31475 | C | 4.24485 | -1.1364 | 1.52862 |
| C | 2.59717 | 0.23902 | -1.64049 | C | 3.169 | 0.82225 | -0.30276 |
| C | 0.65338 | 2.11951 | -0.45653 | C | 0.26119 | 1.15278 | -0.00164 |
| C | 0.64765 | -5.13743 | 0.40212 | C | 3.68079 | -5.30475 | -0.79677 |
| O | 1.16901 | -7.42434 | 1.40702 | O | 4.00875 | -7.74158 | -0.11324 |
| C | 2.81481 | -7.26765 | 3.60433 | C | 3.61165 | -8.10113 | 2.6011 |
| C | 3.18352 | -4.43532 | 4.1304 | C | 3.71454 | -5.44014 | 3.71747 |
| C | -3.72588 | 1.7519 | 1.90128 | C | -3.73026 | -1.57983 | -1.15114 |
| C | -6.06436 | 0.2112 | 2.28907 | C | -5.71602 | -0.49295 | 0.5376 |
| O | -3.69596 | 3.98538 | 2.44359 | O | -4.33108 | -2.3469 | -3.23638 |
| C | -0.64485 | 3.48167 | -2.5904 | C | -0.71424 | 2.799 | -2.10914 |
| C | 1.91797 | 3.90215 | 1.43911 | C | -0.4145 | 2.16663 | 2.62009 |
| C | -1.00346 | 5.97862 | -2.82725 | C | -2.37377 | 4.7055 | -1.90195 |
| C | -2.28354 | 7.09881 | -4.96392 | C | -3.34026 | 6.08957 | -4.0475 |
| C | -2.77393 | 9.56914 | -5.34187 | C | -5.13431 | 7.89788 | -4.01162 |
| C | -4.13993 | 10.4357 | -7.67192 | C | -5.95549 | 9.17185 | -6.40849 |
| C | -2.0572 | 11.6496 | -3.55418 | C | -6.46647 | 8.80476 | -1.67787 |
| C | 4.73831 | 1.5753 | -3.1084 | C | 4.63344 | 3.33286 | -0.04626 |
| C | 6.07139 | -0.2226 | -4.84707 | C | 7.34359 | 3.09888 | -0.88023 |
| C | 8.31841 | -1.33694 | -4.51962 | C | 8.15688 | 3.10196 | -3.27478 |
| C | 9.33342 | -3.17127 | -6.43944 | C | 10.9052 | 2.76574 | -3.90406 |
| C | 10.0145 | -0.94216 | -2.27567 | C | 6.44691 | 3.39511 | -5.52268 |
| C | 5.24741 | -8.74792 | 2.9971 | C | 5.55899 | -10.0322 | 3.53595 |
| C | 4.62016 | -11.4749 | 2.28046 | C | 5.11211 | -10.5298 | 6.33512 |
| O | 6.55257 | -7.51757 | 0.9584 | O | 5.06833 | -12.3874 | 2.28399 |
| C | 7.02854 | -8.68543 | 5.25806 | C | 8.28543 | -9.21607 | 3.03088 |
| O | -0.76541 | -1.02759 | 5.10006 | O | -1.34731 | -4.7079 | 2.76626 |
| O | -0.62009 | -4.93702 | -1.48326 | O | 3.97037 | -4.58357 | -2.93273 |
| C | -6.27595 | -2.32179 | 1.55458 | C | -7.60412 | 0.94417 | -0.63546 |
| C | -8.5056 | -3.64431 | 1.98549 | C | -9.52268 | 2.02512 | 0.78733 |
| C | -10.541 | -2.45896 | 3.15211 | C | -9.61898 | 1.62633 | 3.38892 |
| C | -10.3452 | 0.0631 | 3.88919 | C | -7.7796 | 0.15352 | 4.55618 |
| C | -8.12588 | 1.38657 | 3.46093 | C | -5.81673 | -0.88275 | 3.14763 |
| H | -2.0134 | -0.90542 | -0.65546 | H | -0.01522 | -2.18148 | -2.29095 |
| H | 5.03137 | -2.87044 | -0.62197 | H | 6.2499 | -1.43057 | 1.09121 |
| H | 4.94682 | -0.43491 | 1.61943 | H | 4.16616 | -0.39248 | 3.4657 |
| H | 1.54326 | -0.91914 | -3.00713 | H | 3.49254 | 0.12605 | -2.22962 |
| H | 1.80431 | -8.22231 | 5.14197 | H | 1.7244 | -8.92965 | 2.81649 |
| H | 2.33892 | -3.89808 | 5.93664 | H | 2.45715 | -5.2555 | 5.34408 |
| H | 5.18931 | -3.95458 | 4.16087 | H | 5.64371 | -4.95275 | 4.28906 |
| H | -1.37139 | 2.24012 | -4.07522 | H | -0.07363 | 2.24662 | -3.99385 |
| H | 2.83204 | 2.84742 | 2.96608 | H | -2.44767 | 2.39967 | 2.85756 |
| H | 3.35915 | 5.04828 | 0.49802 | H | 0.2236 | 0.88635 | 4.11555 |
| H | 0.52948 | 5.15162 | 2.30777 | H | 0.47366 | 4.00817 | 2.92752 |
| H | -0.32597 | 7.22136 | -1.34017 | H | -3.06659 | 5.22347 | -0.03932 |
| H | -2.9188 | 5.77244 | -6.40852 | H | -2.53665 | 5.57604 | -5.87462 |
| H | -4.62437 | 8.86007 | -8.91694 | H | -7.979 | 8.86286 | -6.75024 |
| H | -5.89025 | 11.4328 | -7.17157 | H | -5.69177 | 11.2262 | -6.27588 |
| H | -2.98428 | 11.7848 | -8.74567 | H | -4.90656 | 8.48507 | -8.04973 |
| H | -3.74953 | 12.6845 | -2.94644 | H | -6.02643 | 10.8023 | -1.32695 |
| H | -1.07987 | 10.9854 | -1.86578 | H | -8.52054 | 8.69709 | -1.9449 |
| H | -0.83599 | 13.0293 | -4.50838 | H | -5.99466 | 7.73084 | 0.01739 |
| H | 6.05363 | 2.45506 | -1.7732 | H | 4.5864 | 3.96528 | 1.92734 |
| H | 3.89844 | 3.11193 | -4.22117 | H | 3.64393 | 4.78232 | -1.15014 |
| H | 5.01109 | -0.71791 | -6.54588 | H | 8.74912 | 2.8479 | 0.60458 |
| H | 11.1367 | -2.5034 | -7.21845 | H | 11.1797 | 1.11212 | -5.12664 |
| H | 9.7174 | -5.01378 | -5.56473 | H | 11.627 | 4.40366 | -4.95276 |
| H | 8.01521 | -3.45351 | -8.005 | H | 12.0593 | 2.51929 | -2.20802 |
| H | 11.9225 | -0.41472 | -2.89371 | H | 7.19799 | 4.82257 | -6.82506 |
| H | 9.33531 | 0.51174 | -0.97962 | H | 6.34523 | 1.61517 | -6.58539 |
| H | 10.2057 | -2.71104 | -1.20509 | H | 4.52212 | 3.9367 | -5.00973 |
| H | 3.67291 | -12.4486 | 3.84189 | H | 5.50961 | -8.84394 | 7.46459 |
| H | 3.36771 | -11.5465 | 0.63628 | H | 3.15662 | -11.1167 | 6.666 |
| H | 6.36544 | -12.4905 | 1.83782 | H | 6.35927 | -12.0523 | 6.96527 |
| H | 5.63203 | -7.87276 | -0.57734 | H | 5.24221 | -12.1066 | 0.48875 |
| H | 7.66151 | -6.76381 | 5.67225 | H | 8.82057 | -7.54323 | 4.12338 |
| H | 6.0994 | -9.45318 | 6.93934 | H | 9.54648 | -10.7613 | 3.5712 |
| H | 8.69838 | -9.83169 | 4.84995 | H | 8.5772 | -8.80482 | 1.02421 |
| H | -4.72603 | -3.29332 | 0.6264 | H | -7.51193 | 1.2221 | -2.66584 |
| H | -8.65056 | -5.60628 | 1.40127 | H | -10.9558 | 3.17013 | -0.13398 |
| H | -12.2806 | -3.49683 | 3.48633 | H | -11.1339 | 2.45258 | 4.50133 |
| H | -11.9311 | 0.99189 | 4.8031 | H | -7.86994 | -0.1924 | 6.57745 |
| H | -7.93257 | 3.34921 | 4.02373 | H | -4.39649 | -2.04339 | 4.05817 |
| **2c** | | | | **2d** | | | |
| Atom | X | Y | Z | Atom | X | Y | Z |
| C | -0.4007 | 0.68265 | 1.38677 | C | -1.0176 | 0.12204 | 0.66894 |
| C | 0.97552 | -1.36751 | 2.82689 | C | 0.08388 | -1.63499 | 2.64105 |
| C | 2.87468 | -2.88755 | 1.21848 | C | 2.42895 | -3.11957 | 1.76885 |
| C | 4.49581 | -1.14111 | -0.4521 | C | 4.34554 | -1.31474 | 0.52616 |
| C | 3.11784 | 1.07127 | -1.70325 | C | 3.24457 | 0.67327 | -1.25916 |
| C | 1.51878 | 2.62117 | 0.23708 | C | 0.99879 | 2.17981 | -0.06954 |
| C | 1.2336 | -4.70005 | -0.34843 | C | 1.4547 | -5.14821 | -0.0628 |
| O | 1.24746 | -7.0217 | 0.7099 | O | 1.21802 | -7.37529 | 1.16446 |
| C | 2.84571 | -7.09818 | 2.95321 | C | 2.02623 | -7.15399 | 3.79882 |
| C | 4.4381 | -4.69299 | 2.84545 | C | 3.53961 | -4.70257 | 3.91347 |
| C | -2.4977 | 1.8571 | 2.98639 | C | -3.59114 | 1.08723 | 1.55731 |
| C | -5.14838 | 1.00073 | 2.51385 | C | -5.76197 | -0.72007 | 1.44524 |
| O | -2.04981 | 3.44928 | 4.58105 | O | -3.88283 | 3.22137 | 2.35796 |
| C | 0.08869 | 4.69341 | -1.11896 | C | -0.16085 | 3.76513 | -2.12691 |
| C | 3.18279 | 3.82443 | 2.29333 | C | 1.84081 | 3.72033 | 2.23021 |
| C | -2.19524 | 4.46629 | -2.19813 | C | -0.60478 | 6.25877 | -2.07948 |
| C | -3.51836 | 6.51231 | -3.42932 | C | -1.70503 | 7.61474 | -4.17862 |
| C | -5.9086 | 6.45355 | -4.30832 | C | -2.24756 | 10.1008 | -4.28795 |
| C | -7.05632 | 8.73726 | -5.53778 | C | -3.39594 | 11.2326 | -6.62151 |
| C | -7.63899 | 4.21265 | -4.13906 | C | -1.7957 | 11.948 | -2.1852 |
| C | 5.03367 | 2.69952 | -3.18042 | C | 5.38462 | 2.41045 | -2.21659 |
| C | 6.08764 | 1.31469 | -5.41585 | C | 7.25914 | 1.03111 | -3.85327 |
| C | 8.42885 | 0.41573 | -5.73979 | C | 6.94356 | 0.45404 | -6.29514 |
| C | 9.14539 | -0.96357 | -8.12025 | C | 8.90903 | -1.00088 | -7.73885 |
| C | 10.5319 | 0.64209 | -3.84448 | C | 4.63822 | 1.15606 | -7.79785 |
| C | 4.29701 | -9.6114 | 2.94289 | C | 3.40547 | -9.59528 | 4.56894 |
| C | 5.76859 | -10.0416 | 0.49616 | C | 5.70959 | -10.1395 | 2.93585 |
| O | 5.99608 | -9.31286 | 5.042 | O | 4.34227 | -9.19753 | 7.08747 |
| C | 2.46864 | -11.7942 | 3.41855 | C | 1.56628 | -11.8177 | 4.50369 |
| O | 0.59479 | -1.87535 | 5.02597 | O | -0.75935 | -1.84707 | 4.76149 |
| O | 0.01971 | -4.22743 | -2.2175 | O | 0.89542 | -4.90715 | -2.25773 |
| C | -7.11185 | 2.5462 | 3.38258 | C | -5.57101 | -3.16054 | 0.44162 |
| C | -9.61046 | 1.8749 | 2.96815 | C | -7.6608 | -4.75299 | 0.41988 |
| C | -10.1835 | -0.36922 | 1.71217 | C | -9.9584 | -3.9291 | 1.39588 |
| C | -8.24516 | -1.93415 | 0.87091 | C | -10.1651 | -1.50096 | 2.39727 |
| C | -5.7363 | -1.24795 | 1.24984 | C | -8.08413 | 0.08948 | 2.42246 |
| H | -1.229 | -0.27827 | -0.25312 | H | -1.28394 | -1.00483 | -1.05461 |
| H | 5.40863 | -2.28403 | -1.92087 | H | 5.73686 | -2.45122 | -0.50871 |
| H | 6.02178 | -0.40228 | 0.7462 | H | 5.37615 | -0.37194 | 2.06234 |
| H | 1.76816 | 0.30808 | -3.0844 | H | 2.48374 | -0.32348 | -2.91356 |
| H | 1.58659 | -7.05609 | 4.59881 | H | 0.29513 | -6.9767 | 4.93492 |
| H | 4.78096 | -3.94738 | 4.7372 | H | 3.34639 | -3.7777 | 5.74635 |
| H | 6.25879 | -5.05045 | 1.93271 | H | 5.54509 | -5.05139 | 3.54745 |
| H | 1.02383 | 6.53011 | -1.17456 | H | -0.68296 | 2.70411 | -3.82304 |
| H | 4.21204 | 2.40796 | 3.39746 | H | 3.24483 | 5.13426 | 1.67974 |
| H | 4.56763 | 5.09734 | 1.43177 | H | 0.23248 | 4.67677 | 3.09379 |
| H | 2.01958 | 4.90413 | 3.60871 | H | 2.69407 | 2.51111 | 3.67672 |
| H | -3.15821 | 2.65184 | -2.12501 | H | -0.13538 | 7.31443 | -0.38225 |
| H | -2.46895 | 8.27621 | -3.61814 | H | -2.1365 | 6.47377 | -5.84075 |
| H | -5.72945 | 10.3177 | -5.62988 | H | -5.22917 | 12.1053 | -6.19053 |
| H | -7.68354 | 8.29699 | -7.46663 | H | -2.19092 | 12.7447 | -7.37657 |
| H | -8.74348 | 9.35884 | -4.50126 | H | -3.68007 | 9.82473 | -8.10607 |
| H | -8.21546 | 3.59964 | -6.0358 | H | -0.99709 | 11.0916 | -0.48924 |
| H | -6.82377 | 2.5934 | -3.15695 | H | -0.51345 | 13.453 | -2.81484 |
| H | -9.37841 | 4.73624 | -3.13638 | H | -3.57383 | 12.8758 | -1.65495 |
| H | 6.5379 | 3.3395 | -1.91173 | H | 6.37849 | 3.2306 | -0.59263 |
| H | 4.05027 | 4.39664 | -3.85742 | H | 4.53267 | 3.99507 | -3.2443 |
| H | 4.7286 | 0.97963 | -6.93115 | H | 8.99947 | 0.40748 | -2.94421 |
| H | 9.81043 | -2.88172 | -7.69074 | H | 8.10557 | -2.74842 | -8.51713 |
| H | 7.55634 | -1.10565 | -9.43232 | H | 9.60247 | 0.10151 | -9.35382 |
| H | 10.7073 | -0.00128 | -9.0894 | H | 10.5244 | -1.50003 | -6.55109 |
| H | 11.1842 | -1.24299 | -3.2713 | H | 3.55853 | -0.546 | -8.29334 |
| H | 12.1695 | 1.59625 | -4.68716 | H | 3.37447 | 2.4387 | -6.78845 |
| H | 9.99681 | 1.67304 | -2.14046 | H | 5.18289 | 2.05376 | -9.58581 |
| H | 7.13277 | -8.52865 | 0.1511 | H | 5.17599 | -10.2579 | 0.94353 |
| H | 6.82082 | -11.8198 | 0.63136 | H | 7.15559 | -8.68247 | 3.16265 |
| H | 4.49319 | -10.1748 | -1.12503 | H | 6.53933 | -11.9418 | 3.51205 |
| H | 6.80032 | -10.9194 | 5.34395 | H | 2.92619 | -9.22304 | 8.23685 |
| H | 3.49281 | -13.5946 | 3.48045 | H | 0.88085 | -12.1451 | 2.58076 |
| H | 1.48837 | -11.5303 | 5.22034 | H | 2.5089 | -13.5296 | 5.17857 |
| H | 1.06337 | -11.9135 | 1.90614 | H | -0.08017 | -11.4497 | 5.70942 |
| H | -6.62169 | 4.27874 | 4.36522 | H | -3.80881 | -3.85281 | -0.34895 |
| H | -11.1218 | 3.099 | 3.62478 | H | -7.49062 | -6.63988 | -0.36875 |
| H | -12.1413 | -0.89816 | 1.3923 | H | -11.5894 | -5.1759 | 1.37516 |
| H | -8.68266 | -3.69278 | -0.0919 | H | -11.9568 | -0.85476 | 3.16223 |
| H | -4.25827 | -2.50222 | 0.5732 | H | -8.20204 | 1.98421 | 3.19866 |
| **2e** | | | | **2f** | | | |
| Atom | X | Y | Z | Atom | X | Y | Z |
| C | -0.71186 | 1.10853 | 1.33646 | C | -1.63979 | 0.18759 | 0.63207 |
| C | 0.58871 | -0.92164 | 2.87591 | C | -0.53277 | -1.35817 | 2.77681 |
| C | 2.43495 | -2.58302 | 1.35252 | C | 1.73915 | -3.04041 | 2.04917 |
| C | 4.23927 | -0.88376 | -0.17536 | C | 3.65968 | -1.43935 | 0.5281 |
| C | 3.03088 | 1.37324 | -1.51922 | C | 2.55052 | 0.28394 | -1.50359 |
| C | 1.3215 | 2.98452 | 0.26094 | C | 0.41626 | 2.03739 | -0.46322 |
| C | 0.79324 | -4.27952 | -0.32884 | C | 0.7419 | -5.22365 | 0.42669 |
| O | 0.55862 | -6.58696 | 0.74095 | O | 1.15707 | -7.46027 | 1.57683 |
| C | 1.9917 | -6.74623 | 3.09199 | C | 2.54134 | -7.19346 | 3.92714 |
| C | 3.79228 | -4.49396 | 3.03807 | C | 2.93425 | -4.34632 | 4.33864 |
| C | -2.86635 | 2.30874 | 2.83349 | C | -4.1234 | 1.39053 | 1.49214 |
| C | -5.12142 | 0.69963 | 3.37897 | C | -6.38583 | -0.29172 | 1.68845 |
| O | -2.77658 | 4.48894 | 3.55974 | O | -4.27563 | 3.62071 | 2.02794 |
| C | -0.03417 | 5.02307 | -1.22598 | C | -0.76857 | 3.36626 | -2.68281 |
| C | 2.85993 | 4.20166 | 2.39977 | C | 1.41229 | 3.85094 | 1.55858 |
| C | -1.40536 | 4.62002 | -3.32174 | C | -1.24505 | 5.84336 | -2.91775 |
| C | -2.68759 | 6.609 | -4.68201 | C | -2.39535 | 6.92854 | -5.14435 |
| C | -4.07816 | 6.37476 | -6.80361 | C | -2.98579 | 9.3749 | -5.53517 |
| C | -5.29944 | 8.64207 | -7.99218 | C | -4.19056 | 10.2043 | -7.96572 |
| C | -4.55256 | 3.93277 | -8.16428 | C | -2.54452 | 11.4612 | -3.66717 |
| C | 5.11726 | 2.9486 | -2.81485 | C | 4.71584 | 1.7593 | -2.79093 |
| C | 6.22397 | 1.58703 | -5.03727 | C | 6.34441 | 0.05979 | -4.3689 |
| C | 8.53337 | 0.58417 | -5.27502 | C | 8.65987 | -0.81134 | -3.84042 |
| C | 9.30318 | -0.76017 | -7.65906 | C | 10.0092 | -2.56386 | -5.62608 |
| C | 10.5495 | 0.65599 | -3.27523 | C | 10.1247 | -0.18935 | -1.48729 |
| C | 3.20077 | -9.37933 | 3.24036 | C | 4.96648 | -8.78855 | 3.73382 |
| C | 4.79362 | -10.0094 | 0.91672 | C | 6.66889 | -7.94613 | 1.55914 |
| O | 4.76793 | -9.17658 | 5.45018 | O | 6.17127 | -8.32061 | 6.12295 |
| C | 1.14917 | -11.3729 | 3.63004 | C | 4.28535 | -11.5809 | 3.47556 |
| O | 0.29162 | -1.19643 | 5.13022 | O | -1.31638 | -1.25075 | 4.92499 |
| O | -0.24257 | -3.74428 | -2.2871 | O | -0.30037 | -5.10291 | -1.59805 |
| C | -6.93414 | 1.64748 | 5.05632 | C | -8.60607 | 0.75202 | 2.68113 |
| C | -9.06595 | 0.24222 | 5.64135 | C | -10.765 | -0.7091 | 2.93424 |
| C | -9.42372 | -2.129 | 4.55079 | C | -10.7406 | -3.23878 | 2.19845 |
| C | -7.63847 | -3.08448 | 2.87398 | C | -8.54623 | -4.2937 | 1.20773 |
| C | -5.49417 | -1.68295 | 2.29222 | C | -6.37687 | -2.83281 | 0.95248 |
| H | -1.52391 | 0.17762 | -0.33184 | H | -2.03562 | -1.14575 | -0.90831 |
| H | 5.22778 | -2.04889 | -1.57631 | H | 5.04448 | -2.70516 | -0.35101 |
| H | 5.68663 | -0.20305 | 1.14833 | H | 4.70383 | -0.29173 | 1.90582 |
| H | 1.80741 | 0.62091 | -3.01526 | H | 1.67032 | -0.91537 | -2.95444 |
| H | 0.63589 | -6.54585 | 4.64872 | H | 1.35276 | -8.01877 | 5.409 |
| H | 4.13244 | -3.7644 | 4.93627 | H | 2.00338 | -3.72911 | 6.07372 |
| H | 5.60445 | -5.00668 | 2.18318 | H | 4.94159 | -3.8956 | 4.50037 |
| H | 0.09495 | 6.93584 | -0.47572 | H | -1.29375 | 2.11343 | -4.24128 |
| H | 1.60889 | 5.23721 | 3.67014 | H | -0.11912 | 5.00027 | 2.31883 |
| H | 3.88799 | 2.80207 | 3.5248 | H | 2.25165 | 2.82347 | 3.14621 |
| H | 4.23921 | 5.52956 | 1.6173 | H | 2.85763 | 5.0983 | 0.76397 |
| H | -1.57794 | 2.69998 | -4.04675 | H | -0.77268 | 7.09607 | -1.36139 |
| H | -2.49048 | 8.49536 | -3.87512 | H | -2.82927 | 5.59414 | -6.65472 |
| H | -4.62666 | 8.907 | -9.93801 | H | -4.47203 | 8.62536 | -9.26753 |
| H | -7.3565 | 8.3933 | -8.11256 | H | -6.03244 | 11.0916 | -7.60792 |
| H | -4.91608 | 10.3723 | -6.9311 | H | -3.02575 | 11.6354 | -8.9161 |
| H | -3.64416 | 2.30341 | -7.28746 | H | -1.32684 | 12.9233 | -4.49495 |
| H | -6.58973 | 3.55242 | -8.26487 | H | -4.33904 | 12.3897 | -3.19612 |
| H | -3.88385 | 4.06201 | -10.1243 | H | -1.67959 | 10.8249 | -1.90811 |
| H | 6.57195 | 3.4507 | -1.43186 | H | 5.83197 | 2.7503 | -1.35766 |
| H | 4.26376 | 4.72342 | -3.47019 | H | 3.87104 | 3.21777 | -4.00126 |
| H | 4.93067 | 1.36615 | -6.62976 | H | 5.47663 | -0.57467 | -6.12928 |
| H | 10.9539 | 0.15307 | -8.52274 | H | 11.8045 | -1.73687 | -6.25645 |
| H | 9.85697 | -2.71901 | -7.25586 | H | 10.4825 | -4.35294 | -4.68593 |
| H | 7.77649 | -0.79068 | -9.05021 | H | 8.8689 | -2.98962 | -7.29504 |
| H | 12.2697 | 1.55052 | -4.01194 | H | 9.14756 | 1.12902 | -0.23845 |
| H | 9.98238 | 1.6697 | -1.57117 | H | 10.5168 | -1.91951 | -0.40834 |
| H | 11.0824 | -1.27299 | -2.72491 | H | 11.9687 | 0.6263 | -1.97319 |
| H | 6.32184 | -8.64479 | 0.65072 | H | 5.70824 | -8.14072 | -0.26302 |
| H | 5.6517 | -11.8784 | 1.15763 | H | 7.27356 | -5.98446 | 1.7992 |
| H | 3.63038 | -10.0547 | -0.79141 | H | 8.36803 | -9.12854 | 1.50534 |
| H | 5.41167 | -10.837 | 5.83431 | H | 7.60921 | -9.43134 | 6.25456 |
| H | 1.99572 | -13.2565 | 3.79952 | H | 3.23377 | -11.9245 | 1.72931 |
| H | 0.07785 | -10.9741 | 5.35363 | H | 6.00339 | -12.7381 | 3.4256 |
| H | -0.15541 | -11.4037 | 2.02572 | H | 3.13082 | -12.1905 | 5.07976 |
| H | -6.6197 | 3.49791 | 5.883 | H | -8.58302 | 2.72277 | 3.24818 |
| H | -10.4565 | 0.9894 | 6.95313 | H | -12.4754 | 0.11778 | 3.71148 |
| H | -11.0965 | -3.22869 | 5.00621 | H | -12.4325 | -4.38463 | 2.39708 |
| H | -7.9147 | -4.92498 | 2.00943 | H | -8.5185 | -6.26138 | 0.62571 |
| H | -4.14336 | -2.46594 | 0.96052 | H | -4.69775 | -3.70525 | 0.16036 |
| **2g** | | | |  |  |  |  |
| Atom | X | Y | Z |  |  |  |  |
| C | -0.5396 | -0.285 | 0.47379 |  |  |  |  |
| C | 0.5008 | -1.7675 | 2.68785 |  |  |  |  |
| C | 2.49922 | -3.76155 | 1.98037 |  |  |  |  |
| C | 4.4757 | -2.64064 | 0.16059 |  |  |  |  |
| C | 3.46855 | -0.89864 | -1.92362 |  |  |  |  |
| C | 1.67665 | 1.19953 | -0.85784 |  |  |  |  |
| C | 1.01498 | -5.94874 | 0.78141 |  |  |  |  |
| O | 0.62617 | -7.78157 | 2.5152 |  |  |  |  |
| C | 1.80303 | -7.14138 | 4.92597 |  |  |  |  |
| C | 3.63951 | -4.99831 | 4.32827 |  |  |  |  |
| C | -2.80609 | 1.28057 | 1.3464 |  |  |  |  |
| C | -5.28191 | -0.05567 | 1.61764 |  |  |  |  |
| O | -2.62237 | 3.51627 | 1.84513 |  |  |  |  |
| C | 0.5247 | 2.6104 | -3.03916 |  |  |  |  |
| C | 3.03666 | 2.9313 | 1.02069 |  |  |  |  |
| C | 0.29806 | 5.12084 | -3.28358 |  |  |  |  |
| C | -0.77669 | 6.28929 | -5.50518 |  |  |  |  |
| C | -0.9487 | 8.78059 | -6.00787 |  |  |  |  |
| C | -2.07442 | 9.69463 | -8.44536 |  |  |  |  |
| C | -0.04313 | 10.8375 | -4.27931 |  |  |  |  |
| C | 5.7449 | 0.1228 | -3.44899 |  |  |  |  |
| C | 5.10744 | 1.01265 | -6.06104 |  |  |  |  |
| C | 5.4325 | 3.31134 | -7.05853 |  |  |  |  |
| C | 4.63103 | 3.87289 | -9.72555 |  |  |  |  |
| C | 6.43841 | 5.55679 | -5.65097 |  |  |  |  |
| C | 2.94401 | -9.55927 | 6.05207 |  |  |  |  |
| C | 4.7569 | -10.8912 | 4.24262 |  |  |  |  |
| O | 4.27803 | -8.61456 | 8.22207 |  |  |  |  |
| C | 0.82629 | -11.3469 | 6.86206 |  |  |  |  |
| O | -0.14435 | -1.42812 | 4.85951 |  |  |  |  |
| O | 0.21567 | -6.09997 | -1.34633 |  |  |  |  |
| C | -7.36911 | 1.37441 | 2.39467 |  |  |  |  |
| C | -9.71113 | 0.2459 | 2.71242 |  |  |  |  |
| C | -10.0036 | -2.33504 | 2.27051 |  |  |  |  |
| C | -7.94222 | -3.77502 | 1.50464 |  |  |  |  |
| C | -5.59354 | -2.64513 | 1.17261 |  |  |  |  |
| H | -1.17938 | -1.67111 | -0.93238 |  |  |  |  |
| H | 5.50568 | -4.21338 | -0.71493 |  |  |  |  |
| H | 5.85842 | -1.60952 | 1.31506 |  |  |  |  |
| H | 2.32622 | -2.05935 | -3.21344 |  |  |  |  |
| H | 0.29755 | -6.46469 | 6.18046 |  |  |  |  |
| H | 3.79482 | -3.6885 | 5.91297 |  |  |  |  |
| H | 5.52068 | -5.73329 | 3.88074 |  |  |  |  |
| H | -0.20272 | 1.40618 | -4.55085 |  |  |  |  |
| H | 3.91768 | 1.84837 | 2.54779 |  |  |  |  |
| H | 4.51378 | 4.01993 | 0.06872 |  |  |  |  |
| H | 1.70528 | 4.24227 | 1.88956 |  |  |  |  |
| H | 0.98055 | 6.33491 | -1.77581 |  |  |  |  |
| H | -1.48657 | 4.98114 | -6.9323 |  |  |  |  |
| H | -3.70746 | 10.9261 | -8.0914 |  |  |  |  |
| H | -0.70372 | 10.8343 | -9.51019 |  |  |  |  |
| H | -2.69209 | 8.13113 | -9.64649 |  |  |  |  |
| H | 0.73379 | 10.1372 | -2.50315 |  |  |  |  |
| H | 1.41696 | 11.9752 | -5.21916 |  |  |  |  |
| H | -1.59669 | 12.1362 | -3.82812 |  |  |  |  |
| H | 7.09533 | -1.44946 | -3.62723 |  |  |  |  |
| H | 6.72195 | 1.58057 | -2.35547 |  |  |  |  |
| H | 4.28362 | -0.43846 | -7.27573 |  |  |  |  |
| H | 3.20337 | 5.38013 | -9.73854 |  |  |  |  |
| H | 6.23068 | 4.55338 | -10.858 |  |  |  |  |
| H | 3.83371 | 2.21002 | -10.6579 |  |  |  |  |
| H | 7.8719 | 6.54596 | -6.77499 |  |  |  |  |
| H | 4.89259 | 6.9053 | -5.31631 |  |  |  |  |
| H | 7.27135 | 5.08172 | -3.82468 |  |  |  |  |
| H | 5.53713 | -12.5736 | 5.16368 |  |  |  |  |
| H | 3.77958 | -11.4984 | 2.52529 |  |  |  |  |
| H | 6.33959 | -9.67129 | 3.71798 |  |  |  |  |
| H | 4.83975 | -10.0449 | 9.20056 |  |  |  |  |
| H | -0.30353 | -11.9319 | 5.23159 |  |  |  |  |
| H | 1.6144 | -13.0522 | 7.73624 |  |  |  |  |
| H | -0.41217 | -10.4155 | 8.23158 |  |  |  |  |
| H | -7.0973 | 3.37763 | 2.74035 |  |  |  |  |
| H | -11.3182 | 1.37374 | 3.3112 |  |  |  |  |
| H | -11.8385 | -3.22071 | 2.5225 |  |  |  |  |
| H | -8.15936 | -5.78572 | 1.15672 |  |  |  |  |
| H | -4.0259 | -3.81849 | 0.56114 |  |  |  |  |

**ECD calculation details of 3.**

**Figure S44.** Optimized geometries of predominant conformers for **3** at the B3LYP-D3(BJ)/6-31G* level in PCM MeOH using ORCA5.0.1.

**Table S17.** Gibbs free energies^a^ and equilibrium populations^b^ of low-energy conformers of **3**.

| Conformers | ∆G(a.u.) | P(%)/100 | G(a.u.) |
| --- | --- | --- | --- |
| **3a** | 0.00209 | 3.04 | -1853.502224 |
| **3b** | 0.00092 | 10.55 | -1853.503399 |
| **3c** | 0.0 | 27.9 | -1853.504318 |
| **3d** | 0.00047 | 17.0 | -1853.50385 |
| **3e** | 0.00023 | 21.81 | -1853.504085 |
| **3f** | 0.00074 | 12.74 | -1853.503578 |
| **3g** | 0.00131 | 6.96 | -1853.503007 |

^a^wB97M-V/def2-TZVP, in a.u.
^b^From ∆G values at 298.15K.

**Table S18.** Cartesian coordinates for the low-energy reoptimized random research conformers of **3** at B3LYP-D3(BJ)/6-31G* level of theory in MeOH.

| **3a** | | | | **3b** | | | |
| --- | --- | --- | --- | --- | --- | --- | --- |
| Atom | X | Y | Z | Atom | X | Y | Z |
| C | 2.13182 | -9.54973 | -0.26553 | C | 1.3564 | -7.94015 | 1.54352 |
| C | 0.43278 | -7.28258 | -1.10421 | C | -0.31199 | -5.86164 | 0.27516 |
| C | 1.71736 | -4.6739 | -0.7776 | C | 0.70416 | -3.14781 | 0.62373 |
| C | 2.56259 | -4.35198 | 1.97147 | C | 1.07222 | -2.60045 | 3.44128 |
| C | 4.31395 | -6.47633 | 2.79492 | C | 2.79454 | -4.53691 | 4.68677 |
| C | 3.02953 | -9.02712 | 2.48236 | C | 1.73209 | -7.18785 | 4.35691 |
| C | 4.41593 | -10.0766 | -1.98314 | C | 3.92817 | -8.39128 | 0.27391 |
| C | 0.50844 | -11.9608 | -0.22619 | C | -0.10945 | -10.4487 | 1.45952 |
| O | 4.78615 | -10.8746 | 3.38155 | O | 3.4013 | -8.85439 | 5.67727 |
| C | -0.80647 | -7.55625 | -3.74837 | C | -1.04212 | -6.41701 | -2.5044 |
| C | -3.11469 | -9.22664 | -3.88104 | C | -3.55061 | -7.70232 | -2.78597 |
| C | -5.38208 | -8.74751 | -2.87034 | C | -4.13711 | -9.72537 | -4.18097 |
| C | -5.99723 | -6.41653 | -1.3703 | C | -2.28434 | -11.214 | -5.73553 |
| C | -7.52986 | -10.5867 | -3.14861 | C | -6.80937 | -10.6853 | -4.31102 |
| C | 3.7829 | -4.06533 | -2.70567 | C | 3.00862 | -2.46761 | -0.98295 |
| C | 3.66973 | -1.72294 | 2.34907 | C | 1.93462 | 0.12214 | 3.78217 |
| C | 1.87626 | 0.23672 | 1.32527 | C | 0.23832 | 1.86341 | 2.30903 |
| C | 1.82357 | 2.7224 | 2.30122 | C | 0.00281 | 4.43572 | 2.93806 |
| C | -0.11169 | 4.43149 | 1.79183 | C | -1.90604 | 5.99407 | 2.00513 |
| C | -2.04448 | 3.63958 | 0.15833 | C | -3.59504 | 4.94104 | 0.2651 |
| C | -1.92894 | 1.27961 | -1.1154 | C | -3.21018 | 2.46964 | -0.72717 |
| C | -0.02296 | -0.4526 | -0.35976 | C | -1.35991 | 0.92213 | 0.44153 |
| O | 3.60368 | 3.39179 | 4.0685 | O | 1.55042 | 5.50776 | 4.71238 |
| C | -0.23213 | 7.04083 | 2.93811 | C | -2.11037 | 8.72122 | 2.83976 |
| C | 0.29955 | 9.0615 | 1.00759 | C | 0.21534 | 10.2727 | 2.23608 |
| C | -1.21027 | 10.9409 | 0.25219 | C | 0.97702 | 10.9499 | -0.07597 |
| C | -0.34031 | 12.7781 | -1.73691 | C | 3.37878 | 12.4153 | -0.46725 |
| C | -3.83747 | 11.4092 | 1.21302 | C | -0.41396 | 10.2501 | -2.44959 |
| O | -3.9735 | 5.23192 | -0.22282 | O | -5.51334 | 6.37624 | -0.56835 |
| C | -3.7645 | 0.78638 | -3.11174 | C | -4.71121 | 1.68625 | -2.89456 |
| C | -3.13096 | -0.83705 | -5.32349 | C | -3.69155 | -0.1055 | -4.81516 |
| O | -5.84573 | 1.89321 | -3.14032 | O | -6.80681 | 2.66946 | -3.34427 |
| C | -5.04432 | -2.24324 | -6.47466 | C | -5.32283 | -1.81161 | -5.99026 |
| C | -4.5268 | -3.67092 | -8.61504 | C | -4.44013 | -3.36647 | -7.91286 |
| C | -2.11673 | -3.63394 | -9.6724 | C | -1.94747 | -3.16702 | -8.7314 |
| C | -0.22344 | -2.16893 | -8.57684 | C | -0.33081 | -1.41903 | -7.60615 |
| C | -0.71945 | -0.79527 | -6.39606 | C | -1.18906 | 0.08766 | -5.63508 |
| C | 6.17694 | 3.73195 | 3.15031 | C | 4.26916 | 5.41087 | 4.30862 |
| C | 6.54943 | 6.15046 | 1.72706 | C | 4.95784 | 5.40767 | 1.57344 |
| C | 6.74209 | 8.44327 | 2.77989 | C | 6.78781 | 4.07283 | 0.45111 |
| C | 7.11352 | 10.7654 | 1.19444 | C | 7.24125 | 4.32508 | -2.34253 |
| C | 6.52259 | 8.92933 | 5.5637 | C | 8.56378 | 2.29155 | 1.76983 |
| O | -0.32656 | -2.85812 | -1.20811 | O | -1.39069 | -1.55599 | -0.23398 |
| H | -1.1316 | -7.24211 | 0.26673 | H | -2.08832 | -5.85135 | 1.36218 |
| H | 0.82145 | -4.47526 | 3.10729 | H | -0.81919 | -2.77901 | 4.2939 |
| H | 4.82779 | -6.23524 | 4.78828 | H | 2.95952 | -4.13667 | 6.71371 |
| H | 6.09176 | -6.44559 | 1.72729 | H | 4.71781 | -4.44388 | 3.91416 |
| H | 1.32484 | -9.03357 | 3.69361 | H | -0.15508 | -7.23091 | 5.25583 |
| H | 3.86544 | -10.0945 | -3.97559 | H | 3.72089 | -8.68857 | -1.76168 |
| H | 5.94587 | -8.71474 | -1.75449 | H | 5.26385 | -6.84766 | 0.55687 |
| H | 5.20369 | -11.9324 | -1.53144 | H | 4.79967 | -10.0876 | 1.06869 |
| H | -0.01401 | -12.5435 | -2.13668 | H | -0.3826 | -11.0937 | -0.4841 |
| H | 1.56753 | -13.5412 | 0.59832 | H | 0.93342 | -11.9436 | 2.44697 |
| H | -1.23997 | -11.6921 | 0.84928 | H | -1.9851 | -10.2641 | 2.31972 |
| H | 3.91488 | -12.4613 | 3.56462 | H | 2.59941 | -10.4819 | 5.81637 |
| H | -1.33373 | -5.661 | -4.38878 | H | -1.18638 | -4.60482 | -3.49903 |
| H | 0.58718 | -8.24013 | -5.11736 | H | 0.45614 | -7.46997 | -3.46119 |
| H | -2.91547 | -10.9849 | -4.93488 | H | -5.08816 | -6.77655 | -1.76687 |
| H | -7.74502 | -5.54831 | -2.07218 | H | -0.33731 | -10.5603 | -5.53934 |
| H | -6.35323 | -6.90439 | 0.6172 | H | -2.3496 | -13.2227 | -5.21774 |
| H | -4.49977 | -5.00013 | -1.428 | H | -2.78405 | -11.1123 | -7.74745 |
| H | -6.97498 | -12.2655 | -4.21862 | H | -6.92585 | -12.65 | -3.65209 |
| H | -8.22341 | -11.1997 | -1.29066 | H | -8.09246 | -9.53947 | -3.16667 |
| H | -9.14045 | -9.70144 | -4.11213 | H | -7.50339 | -10.6795 | -6.26731 |
| H | 4.07153 | -2.01969 | -2.79579 | H | 3.14662 | -0.41176 | -1.17406 |
| H | 5.588 | -4.9285 | -2.20887 | H | 4.75899 | -3.12769 | -0.11498 |
| H | 3.24082 | -4.71159 | -4.58943 | H | 2.86212 | -3.28214 | -2.87482 |
| H | 5.52929 | -1.64314 | 1.42297 | H | 3.91209 | 0.29654 | 3.17039 |
| H | 4.00864 | -1.36067 | 4.361 | H | 1.88059 | 0.6414 | 5.78881 |
| H | -2.08887 | 7.31055 | 3.80638 | H | -3.78501 | 9.54779 | 1.95484 |
| H | 1.17961 | 7.14176 | 4.44719 | H | -2.42867 | 8.78005 | 4.88487 |
| H | 2.14734 | 8.89635 | 0.11395 | H | 1.38089 | 10.8243 | 3.83963 |
| H | 1.58526 | 12.3692 | -2.36786 | H | 4.33789 | 12.8138 | 1.31961 |
| H | -1.59265 | 12.7167 | -3.39045 | H | 4.69191 | 11.3635 | -1.68626 |
| H | -0.39589 | 14.727 | -1.02577 | H | 3.00261 | 14.2163 | -1.42531 |
| H | -3.96693 | 13.2801 | 2.10128 | H | -2.3217 | 9.55398 | -2.08891 |
| H | -5.17829 | 11.4056 | -0.36927 | H | -0.53493 | 11.8762 | -3.72953 |
| H | -4.47021 | 9.99306 | 2.56877 | H | 0.61158 | 8.76364 | -3.47937 |
| H | -5.21222 | 4.29412 | -1.28587 | H | -6.54253 | 5.26447 | -1.68895 |
| H | -6.92605 | -2.20842 | -5.66019 | H | -7.27563 | -1.91047 | -5.3686 |
| H | -6.00629 | -4.80938 | -9.46688 | H | -5.69958 | -4.73666 | -8.77746 |
| H | -1.71794 | -4.73423 | -11.3589 | H | -1.26548 | -4.36614 | -10.2517 |
| H | 1.64622 | -2.10331 | -9.4227 | H | 1.60575 | -1.23689 | -8.26369 |
| H | 0.75053 | 0.34372 | -5.52644 | H | 0.06473 | 1.44225 | -4.73422 |
| H | 7.32912 | 3.6586 | 4.86512 | H | 4.94425 | 7.14352 | 5.2284 |
| H | 6.70718 | 2.12784 | 1.95505 | H | 5.06689 | 3.80081 | 5.33728 |
| H | 6.64251 | 6.00401 | -0.32387 | H | 3.82476 | 6.69201 | 0.42976 |
| H | 7.24608 | 10.3153 | -0.81759 | H | 7.06869 | 2.47939 | -3.27684 |
| H | 5.53726 | 12.087 | 1.46915 | H | 5.90175 | 5.63024 | -3.22041 |
| H | 8.83939 | 11.7668 | 1.76017 | H | 9.16403 | 5.00222 | -2.72713 |
| H | 6.28992 | 7.2108 | 6.67975 | H | 10.5071 | 3.00677 | 1.64512 |
| H | 8.17768 | 9.96786 | 6.25767 | H | 8.13129 | 2.01334 | 3.76784 |
| H | 4.87446 | 10.1388 | 5.91981 | H | 8.56165 | 0.44081 | 0.83247 |
| **3c** | | | | **3d** | | | |
| Atom | X | Y | Z | Atom | X | Y | Z |
| C | 0.88209 | -9.19347 | 0.48444 | C | 2.82988 | -7.97803 | -0.99008 |
| C | -0.31348 | -6.75182 | -0.66393 | C | 0.74851 | -5.91768 | -1.32956 |
| C | 1.41659 | -4.40376 | -0.5621 | C | 1.78455 | -3.27265 | -1.98684 |
| C | 2.26466 | -3.94294 | 2.1662 | C | 3.68689 | -2.45734 | 0.03448 |
| C | 3.56859 | -6.26181 | 3.2597 | C | 5.81479 | -4.37248 | 0.31544 |
| C | 1.81075 | -8.54907 | 3.17802 | C | 4.77856 | -6.98579 | 0.96641 |
| C | 3.05489 | -10.3424 | -1.06667 | C | 4.19474 | -8.74839 | -3.4406 |
| C | -1.19698 | -11.2132 | 0.71009 | C | 1.59057 | -10.3647 | 0.10658 |
| O | 3.02249 | -10.7484 | 4.18954 | O | 6.78275 | -8.74328 | 1.41546 |
| C | -1.51598 | -7.12232 | -3.30776 | C | -1.46826 | -6.71626 | -3.06691 |
| C | -4.29449 | -7.66539 | -3.21848 | C | -3.70024 | -7.71322 | -1.63749 |
| C | -5.58083 | -9.50104 | -4.38336 | C | -5.04074 | -9.812 | -2.0586 |
| C | -4.41779 | -11.5126 | -6.01576 | C | -4.50198 | -11.7026 | -4.10772 |
| C | -8.40576 | -9.69055 | -4.13066 | C | -7.29625 | -10.4495 | -0.45029 |
| C | 3.59167 | -4.38904 | -2.46527 | C | 2.76782 | -2.96936 | -4.68735 |
| C | 3.82988 | -1.53227 | 2.29637 | C | 4.56962 | 0.23216 | -0.46624 |
| C | 2.41416 | 0.58872 | 1.03027 | C | 2.31403 | 1.91467 | -0.86468 |
| C | 2.78109 | 3.12887 | 1.75152 | C | 2.46419 | 4.52968 | -0.40379 |
| C | 1.18477 | 5.08776 | 1.02466 | C | 0.37829 | 6.13717 | -0.51938 |
| C | -0.83374 | 4.48905 | -0.58077 | C | -1.96778 | 5.0454 | -1.07749 |
| C | -1.12691 | 2.02199 | -1.60259 | C | -2.20351 | 2.4537 | -1.74354 |
| C | 0.43497 | 0.06837 | -0.62786 | C | -0.02782 | 0.90204 | -1.51038 |
| O | 4.65988 | 3.69172 | 3.45908 | O | 4.82853 | 5.43577 | 0.16478 |
| C | 1.48035 | 7.74125 | 2.02709 | C | 0.53599 | 8.95776 | -0.12082 |
| C | 0.05531 | 8.07395 | 4.46703 | C | -0.34171 | 9.75823 | 2.47173 |
| C | -1.94795 | 9.54971 | 4.90899 | C | 0.65818 | 11.5427 | 3.95701 |
| C | -3.1581 | 9.6163 | 7.48244 | C | -0.45354 | 12.1034 | 6.513 |
| C | -3.19019 | 11.2327 | 2.99042 | C | 2.90118 | 13.1482 | 3.27665 |
| O | -2.4261 | 6.35402 | -1.20902 | O | -3.98791 | 6.57879 | -1.07362 |
| C | -3.01901 | 1.63725 | -3.56666 | C | -4.64092 | 1.56574 | -2.67151 |
| C | -2.71739 | -0.34989 | -5.53939 | C | -4.84429 | -0.53817 | -4.53344 |
| O | -4.84879 | 3.10719 | -3.79009 | O | -6.63206 | 2.69645 | -2.11192 |
| C | -4.85384 | -1.59682 | -6.45315 | C | -6.86096 | -2.22899 | -4.3655 |
| C | -4.62917 | -3.33206 | -8.41034 | C | -7.1845 | -4.07803 | -6.20075 |
| C | -2.2855 | -3.76889 | -9.52298 | C | -5.54993 | -4.19335 | -8.25942 |
| C | -0.15905 | -2.47693 | -8.65966 | C | -3.57447 | -2.46453 | -8.46728 |
| C | -0.36665 | -0.79451 | -6.6557 | C | -3.20198 | -0.66146 | -6.59626 |
| C | 7.23151 | 3.54264 | 2.47721 | C | 5.20902 | 6.34075 | 2.755 |
| C | 7.67806 | 5.29191 | 0.30175 | C | 4.1376 | 4.59822 | 4.69954 |
| C | 8.36473 | 7.72121 | 0.47415 | C | 5.39143 | 2.8103 | 5.97861 |
| C | 8.70026 | 9.31101 | -1.85162 | C | 4.05838 | 1.18097 | 7.88304 |
| C | 8.80005 | 9.09193 | 2.9192 | C | 8.153 | 2.23281 | 5.66527 |
| O | -0.27146 | -2.32066 | -1.24651 | O | -0.42657 | -1.62361 | -1.74945 |
| H | -1.87225 | -6.25481 | 0.6245 | H | -0.0575 | -5.65892 | 0.57269 |
| H | 0.5146 | -3.61972 | 3.24784 | H | 2.61881 | -2.4343 | 1.82403 |
| H | 4.10886 | -5.87775 | 5.22942 | H | 7.10267 | -3.77654 | 1.82805 |
| H | 5.32543 | -6.69125 | 2.24399 | H | 6.95488 | -4.45703 | -1.42097 |
| H | 0.11211 | -8.10342 | 4.31324 | H | 3.79666 | -6.85363 | 2.79493 |
| H | 3.48953 | -12.2244 | -0.33683 | H | 2.86201 | -9.05094 | -4.99093 |
| H | 2.52841 | -10.5449 | -3.05543 | H | 5.57987 | -7.35933 | -4.08772 |
| H | 4.79814 | -9.24418 | -0.97917 | H | 5.1878 | -10.5407 | -3.14741 |
| H | -0.47871 | -12.8368 | 1.76729 | H | 3.05626 | -11.7163 | 0.65401 |
| H | -2.86344 | -10.4681 | 1.68845 | H | 0.44797 | -9.9188 | 1.77489 |
| H | -1.80953 | -11.865 | -1.15252 | H | 0.34547 | -11.2686 | -1.27441 |
| H | 3.44653 | -10.4063 | 5.92593 | H | 7.8442 | -8.77133 | -0.06611 |
| H | -1.28622 | -5.35532 | -4.36547 | H | -2.11326 | -5.0404 | -4.0998 |
| H | -0.50293 | -8.56501 | -4.38524 | H | -0.83485 | -8.06072 | -4.50259 |
| H | -5.37978 | -6.31145 | -2.10103 | H | -4.32433 | -6.48709 | -0.09869 |
| H | -4.95105 | -13.3979 | -5.33139 | H | -4.28614 | -13.6033 | -3.30313 |
| H | -5.13231 | -11.3707 | -7.95923 | H | -6.09798 | -11.8036 | -5.43077 |
| H | -2.3584 | -11.417 | -6.09375 | H | -2.8077 | -11.2709 | -5.20304 |
| H | -8.95413 | -11.5106 | -3.297 | H | -9.00958 | -10.6176 | -1.61015 |
| H | -9.1734 | -8.1789 | -2.9496 | H | -7.04147 | -12.2812 | 0.49183 |
| H | -9.32589 | -9.59416 | -5.98895 | H | -7.63488 | -9.01636 | 0.99908 |
| H | 5.21383 | -5.46583 | -1.78482 | H | 4.70223 | -3.6556 | -4.89121 |
| H | 2.99368 | -5.17944 | -4.27658 | H | 1.56711 | -3.98515 | -6.02551 |
| H | 4.22071 | -2.44666 | -2.79516 | H | 2.76151 | -0.97057 | -5.21648 |
| H | 5.67647 | -1.8815 | 1.40715 | H | 5.84096 | 0.28707 | -2.11118 |
| H | 4.21756 | -1.01833 | 4.26621 | H | 5.67467 | 0.92801 | 1.13512 |
| H | 3.49223 | 8.07679 | 2.36922 | H | 2.46574 | 9.58071 | -0.51201 |
| H | 0.85164 | 9.08526 | 0.59304 | H | -0.68556 | 9.85892 | -1.53797 |
| H | 0.75931 | 6.91204 | 6.0177 | H | -2.00705 | 8.75678 | 3.15144 |
| H | -3.16737 | 11.5456 | 8.24644 | H | -2.09077 | 10.9093 | 6.91381 |
| H | -5.14096 | 9.01366 | 7.37581 | H | 0.95414 | 11.8043 | 8.00864 |
| H | -2.17802 | 8.38956 | 8.82559 | H | -1.04593 | 14.0874 | 6.64514 |
| H | -5.204 | 10.7613 | 2.83369 | H | 4.39331 | 12.9685 | 4.70869 |
| H | -3.09091 | 13.2151 | 3.5963 | H | 3.7088 | 12.6941 | 1.43325 |
| H | -2.36721 | 11.0728 | 1.109 | H | 2.35974 | 15.1506 | 3.2468 |
| H | -3.79926 | 5.54103 | -2.21162 | H | -5.48184 | 5.46115 | -1.32695 |
| H | -6.67772 | -1.20206 | -5.60042 | H | -8.15461 | -2.08432 | -2.77947 |
| H | -6.28613 | -4.34699 | -9.07001 | H | -8.71743 | -5.43108 | -6.02723 |
| H | -2.11533 | -5.10941 | -11.0683 | H | -5.82009 | -5.62282 | -9.70788 |
| H | 1.66613 | -2.79123 | -9.54576 | H | -2.31772 | -2.52782 | -10.0897 |
| H | 1.28524 | 0.20703 | -5.95929 | H | -1.65794 | 0.68457 | -6.73856 |
| H | 8.41066 | 3.99278 | 4.11078 | H | 7.25703 | 6.54599 | 2.90129 |
| H | 7.64408 | 1.59242 | 1.91118 | H | 4.34359 | 8.20786 | 2.96334 |
| H | 7.34648 | 4.50745 | -1.57223 | H | 2.12543 | 4.83166 | 5.0696 |
| H | 7.35695 | 10.8919 | -1.84418 | H | 4.95833 | 1.33648 | 9.74535 |
| H | 10.598 | 10.1458 | -1.90493 | H | 2.06592 | 1.68931 | 8.0707 |
| H | 8.41171 | 8.2196 | -3.58064 | H | 4.17863 | -0.81776 | 7.33736 |
| H | 8.42376 | 7.93851 | 4.58624 | H | 9.17761 | 2.62966 | 7.42455 |
| H | 10.749 | 9.79419 | 3.01884 | H | 8.41551 | 0.21102 | 5.28263 |
| H | 7.56995 | 10.7589 | 3.01836 | H | 9.04557 | 3.29008 | 4.13615 |
| **3e** | | | | **3f** | | | |
| Atom | X | Y | Z | Atom | X | Y | Z |
| C | 1.12894 | -8.87575 | 0.51347 | C | 2.80078 | -8.25786 | 0.03265 |
| C | -0.17001 | -6.43915 | -0.53018 | C | 0.76642 | -6.31534 | -0.86695 |
| C | 1.43954 | -4.01866 | -0.24141 | C | 1.44327 | -3.53043 | -0.27997 |
| C | 2.20607 | -3.68438 | 2.5273 | C | 1.9574 | -3.23481 | 2.55218 |
| C | 3.58743 | -5.99455 | 3.53919 | C | 4.02722 | -5.0197 | 3.44634 |
| C | 1.94066 | -8.33752 | 3.28355 | C | 3.34115 | -7.76053 | 2.87602 |
| C | 3.40222 | -9.80591 | -1.03433 | C | 5.28802 | -8.20879 | -1.47407 |
| C | -0.83692 | -11.016 | 0.53629 | C | 1.71878 | -10.9459 | -0.18745 |
| O | 3.32685 | -10.3776 | 4.38927 | O | 5.19268 | -9.45036 | 3.88652 |
| C | -1.26857 | -6.7026 | -3.22814 | C | -0.14533 | -6.65013 | -3.63327 |
| C | -3.97971 | -7.52091 | -3.28953 | C | -2.02433 | -8.74885 | -4.07649 |
| C | -5.05375 | -9.29361 | -4.73429 | C | -4.42968 | -8.79828 | -3.3005 |
| C | -3.65871 | -10.9358 | -6.58377 | C | -5.66114 | -6.72787 | -1.79661 |
| C | -7.85196 | -9.77803 | -4.61579 | C | -6.105 | -11.024 | -3.85862 |
| C | 3.65357 | -3.77618 | -2.08653 | C | 3.50425 | -2.37743 | -1.94702 |
| C | 3.65704 | -1.21869 | 2.83728 | C | 2.46862 | -0.47025 | 3.16236 |
| C | 2.22543 | 0.90813 | 1.60018 | C | 0.45351 | 1.17282 | 2.0174 |
| C | 2.56471 | 3.44049 | 2.36478 | C | -0.06206 | 3.60726 | 2.95993 |
| C | 0.88997 | 5.37019 | 1.72854 | C | -2.21061 | 4.9783 | 2.30136 |
| C | -1.12419 | 4.76833 | 0.12262 | C | -3.82579 | 3.93556 | 0.48574 |
| C | -1.33786 | 2.33902 | -1.00503 | C | -3.20897 | 1.65265 | -0.78855 |
| C | 0.27871 | 0.39912 | -0.09569 | C | -1.12457 | 0.23806 | 0.13019 |
| O | 4.47491 | 4.08172 | 3.98534 | O | 1.46143 | 4.67457 | 4.76527 |
| C | 1.1144 | 7.99982 | 2.81004 | C | -2.71876 | 7.5806 | 3.36028 |
| C | -0.37079 | 8.23087 | 5.22714 | C | -1.58772 | 9.5544 | 1.65142 |
| C | -2.40027 | 9.66808 | 5.67439 | C | 0.28905 | 11.1687 | 2.16501 |
| C | -3.66882 | 9.62884 | 8.21998 | C | 1.24541 | 12.9655 | 0.17861 |
| C | -3.61656 | 11.4076 | 3.79043 | C | 1.62581 | 11.3758 | 4.66114 |
| O | -2.77315 | 6.60717 | -0.43266 | O | -5.90087 | 5.25605 | -0.13353 |
| C | -3.19105 | 1.98525 | -3.00491 | C | -4.70537 | 0.95094 | -2.99345 |
| C | -2.79748 | 0.1035 | -5.06517 | C | -3.56285 | -0.4316 | -5.16485 |
| O | -5.06551 | 3.40525 | -3.19055 | O | -6.92627 | 1.69972 | -3.23531 |
| C | -4.87436 | -1.18272 | -6.05839 | C | -5.05423 | -2.10384 | -6.5583 |
| C | -4.565 | -2.80877 | -8.09653 | C | -4.0687 | -3.29819 | -8.67717 |
| C | -2.19759 | -3.0955 | -9.20789 | C | -1.61829 | -2.75952 | -9.47278 |
| C | -0.13217 | -1.76268 | -8.26253 | C | -0.1524 | -1.03047 | -8.1344 |
| C | -0.42299 | -0.18981 | -6.18093 | C | -1.10848 | 0.10928 | -5.9715 |
| C | 7.07339 | 3.43754 | 3.27187 | C | 4.12433 | 5.01733 | 4.17528 |
| C | 7.52507 | 3.35407 | 0.48367 | C | 4.5318 | 5.63613 | 1.45398 |
| C | 7.74548 | 5.37064 | -1.02929 | C | 6.36134 | 4.77142 | -0.06152 |
| C | 8.09684 | 5.06137 | -3.82661 | C | 6.47748 | 5.5832 | -2.78202 |
| C | 7.58845 | 8.0538 | -0.13327 | C | 8.44848 | 3.01261 | 0.72455 |
| O | -0.33731 | -1.9833 | -0.82945 | O | -0.89006 | -2.14041 | -0.81448 |
| H | -1.78703 | -6.09487 | 0.7359 | H | -0.89013 | -6.68362 | 0.33587 |
| H | 0.41871 | -3.49704 | 3.57919 | H | 0.18664 | -3.7832 | 3.50016 |
| H | 4.047 | -5.72267 | 5.54189 | H | 4.30257 | -4.81607 | 5.49131 |
| H | 5.39191 | -6.29083 | 2.55929 | H | 5.84218 | -4.53125 | 2.55619 |
| H | 0.19115 | -8.0078 | 4.37959 | H | 1.60128 | -8.22131 | 3.91741 |
| H | 4.00743 | -11.6499 | -0.32554 | H | 4.93442 | -8.16873 | -3.50962 |
| H | 2.90908 | -10.033 | -3.02931 | H | 6.5025 | -6.59879 | -1.02708 |
| H | 5.03668 | -8.55655 | -0.92169 | H | 6.36293 | -9.93571 | -1.08989 |
| H | -0.04428 | -12.7315 | 1.38805 | H | 1.61537 | -11.5539 | -2.1589 |
| H | -2.54053 | -10.4762 | 1.58443 | H | 2.94473 | -12.2622 | 0.8326 |
| H | -1.42373 | -11.5139 | -1.38061 | H | -0.18911 | -11.0607 | 0.6056 |
| H | 2.19813 | -11.7879 | 4.60933 | H | 6.81185 | -8.98717 | 3.18942 |
| H | -1.19217 | -4.83638 | -4.12528 | H | -1.00456 | -4.86377 | -4.22373 |
| H | -0.08036 | -7.93346 | -4.38675 | H | 1.47617 | -6.93222 | -4.88878 |
| H | -5.2176 | -6.43627 | -2.04432 | H | -1.35257 | -10.3738 | -5.14763 |
| H | -4.06921 | -12.9392 | -6.23137 | H | -6.05668 | -7.3732 | 0.13792 |
| H | -4.29044 | -10.54 | -8.52107 | H | -4.51406 | -5.01788 | -1.68109 |
| H | -1.6127 | -10.6768 | -6.52746 | H | -7.49636 | -6.23046 | -2.62451 |
| H | -8.79239 | -8.51713 | -3.27598 | H | -5.10891 | -12.4919 | -4.91842 |
| H | -8.72969 | -9.51674 | -6.4787 | H | -6.82383 | -11.8648 | -2.10217 |
| H | -8.24248 | -11.737 | -4.05174 | H | -7.76846 | -10.4406 | -4.95341 |
| H | 5.32186 | -4.78886 | -1.41871 | H | 3.4102 | -0.31232 | -1.84882 |
| H | 3.14792 | -4.49961 | -3.95308 | H | 5.392 | -2.94289 | -1.34007 |
| H | 4.17153 | -1.78408 | -2.29383 | H | 3.24926 | -2.93572 | -3.91763 |
| H | 5.56503 | -1.44801 | 2.04154 | H | 4.35132 | 0.04505 | 2.44936 |
| H | 3.90755 | -0.79591 | 4.85144 | H | 2.50836 | -0.19162 | 5.21655 |
| H | 3.10799 | 8.37096 | 3.21852 | H | -4.76788 | 7.86078 | 3.45763 |
| H | 0.4862 | 9.37554 | 1.40534 | H | -1.97291 | 7.7111 | 5.27671 |
| H | 0.31254 | 7.02332 | 6.75194 | H | -2.39229 | 9.58692 | -0.2462 |
| H | -3.71365 | 11.5286 | 9.05351 | H | 3.25371 | 12.6151 | -0.21904 |
| H | -5.64288 | 9.01294 | 8.04618 | H | 0.19128 | 12.7895 | -1.58953 |
| H | -2.70732 | 8.36245 | 9.53946 | H | 1.10843 | 14.9318 | 0.82632 |
| H | -5.62066 | 10.9215 | 3.56871 | H | 1.29442 | 13.2415 | 5.50687 |
| H | -3.55278 | 13.3669 | 4.47147 | H | 1.04609 | 9.93709 | 6.01966 |
| H | -2.7483 | 11.3279 | 1.92435 | H | 3.67755 | 11.2182 | 4.38425 |
| H | -4.11164 | 5.79671 | -1.48335 | H | -6.87241 | 4.16831 | -1.31883 |
| H | -6.71928 | -0.90268 | -5.20537 | H | -6.97937 | -2.45958 | -5.94726 |
| H | -6.17477 | -3.85468 | -8.82182 | H | -5.21486 | -4.64559 | -9.71691 |
| H | -1.96165 | -4.34987 | -10.8158 | H | -0.85664 | -3.67615 | -11.1442 |
| H | 1.71068 | -1.95855 | -9.14626 | H | 1.74344 | -0.57447 | -8.77787 |
| H | 1.18148 | 0.84273 | -5.42047 | H | 0.02838 | 1.45147 | -4.91174 |
| H | 8.19489 | 4.91255 | 4.18973 | H | 4.67085 | 6.60815 | 5.3871 |
| H | 7.58208 | 1.6304 | 4.14409 | H | 5.20341 | 3.36207 | 4.79189 |
| H | 7.6126 | 1.48846 | -0.38071 | H | 3.14559 | 6.95067 | 0.67882 |
| H | 6.53324 | 5.9583 | -4.8532 | H | 8.28023 | 6.51748 | -3.20593 |
| H | 9.8352 | 5.99956 | -4.4575 | H | 6.36017 | 3.93158 | -4.03417 |
| H | 8.18406 | 3.07236 | -4.37775 | H | 4.93988 | 6.87616 | -3.26219 |
| H | 7.56876 | 8.22496 | 1.92206 | H | 10.2812 | 3.96912 | 0.55173 |
| H | 9.17253 | 9.16149 | -0.88137 | H | 8.2815 | 2.33908 | 2.66745 |
| H | 5.85227 | 8.9328 | -0.85232 | H | 8.52656 | 1.36938 | -0.53991 |
| **3g** | | | |  |  |  |  |
| Atom | X | Y | Z |  |  |  |  |
| C | 3.4456 | -7.86498 | -2.66041 |  |  |  |  |
| C | 1.41867 | -5.72763 | -2.86046 |  |  |  |  |
| C | 2.51614 | -3.02397 | -2.78117 |  |  |  |  |
| C | 4.17104 | -2.68327 | -0.43402 |  |  |  |  |
| C | 6.241 | -4.67281 | -0.2899 |  |  |  |  |
| C | 5.11631 | -7.32702 | -0.30561 |  |  |  |  |
| C | 5.1234 | -8.17602 | -5.01311 |  |  |  |  |
| C | 2.0951 | -10.4006 | -2.22826 |  |  |  |  |
| O | 7.03481 | -9.19708 | 0.05067 |  |  |  |  |
| C | -0.49261 | -6.05938 | -5.05455 |  |  |  |  |
| C | -2.78194 | -7.57384 | -4.35538 |  |  |  |  |
| C | -3.80521 | -9.53717 | -5.57262 |  |  |  |  |
| C | -2.74312 | -10.7251 | -7.92275 |  |  |  |  |
| C | -6.17966 | -10.7645 | -4.60421 |  |  |  |  |
| C | 3.7925 | -2.14952 | -5.22387 |  |  |  |  |
| C | 5.1274 | 0.02569 | -0.30057 |  |  |  |  |
| C | 2.97425 | 1.83468 | -0.70691 |  |  |  |  |
| C | 3.21669 | 4.41727 | -0.12985 |  |  |  |  |
| C | 1.33559 | 6.19093 | -0.64741 |  |  |  |  |
| C | -0.96241 | 5.28318 | -1.59622 |  |  |  |  |
| C | -1.41869 | 2.65617 | -1.92292 |  |  |  |  |
| C | 0.66597 | 0.99228 | -1.65329 |  |  |  |  |
| O | 5.48854 | 5.18455 | 0.86422 |  |  |  |  |
| C | 1.72193 | 9.00351 | -0.33557 |  |  |  |  |
| C | 0.31905 | 10.0802 | 1.89715 |  |  |  |  |
| C | 1.21895 | 11.5893 | 3.71631 |  |  |  |  |
| C | -0.46673 | 12.4774 | 5.82761 |  |  |  |  |
| C | 3.89019 | 12.5426 | 3.87744 |  |  |  |  |
| O | -2.75357 | 6.99441 | -2.14204 |  |  |  |  |
| C | -3.98864 | 1.84008 | -2.5097 |  |  |  |  |
| C | -5.01522 | -0.62669 | -1.61134 |  |  |  |  |
| O | -5.49865 | 3.31417 | -3.56044 |  |  |  |  |
| C | -4.21741 | -1.70377 | 0.66553 |  |  |  |  |
| C | -5.45532 | -3.80238 | 1.64091 |  |  |  |  |
| C | -7.47105 | -4.8733 | 0.32936 |  |  |  |  |
| C | -8.24857 | -3.83314 | -1.96012 |  |  |  |  |
| C | -7.04766 | -1.70264 | -2.91082 |  |  |  |  |
| C | 5.35343 | 5.84621 | 3.5585 |  |  |  |  |
| C | 4.97537 | 3.555 | 5.16038 |  |  |  |  |
| C | 2.74997 | 2.66313 | 5.97927 |  |  |  |  |
| C | 2.60906 | 0.20495 | 7.39349 |  |  |  |  |
| C | 0.24042 | 3.9117 | 5.53746 |  |  |  |  |
| O | 0.2839 | -1.42527 | -2.42419 |  |  |  |  |
| H | 0.31517 | -5.85805 | -1.09801 |  |  |  |  |
| H | 2.89684 | -2.98108 | 1.18893 |  |  |  |  |
| H | 7.33612 | -4.42301 | 1.45303 |  |  |  |  |
| H | 7.58014 | -4.4458 | -1.86418 |  |  |  |  |
| H | 3.89472 | -7.52153 | 1.36668 |  |  |  |  |
| H | 6.46529 | -6.63248 | -5.29785 |  |  |  |  |
| H | 6.1992 | -9.93797 | -4.86032 |  |  |  |  |
| H | 3.98819 | -8.33104 | -6.73278 |  |  |  |  |
| H | 3.48663 | -11.8479 | -1.73398 |  |  |  |  |
| H | 0.71197 | -10.2635 | -0.69323 |  |  |  |  |
| H | 1.08306 | -11.0092 | -3.92335 |  |  |  |  |
| H | 8.31241 | -8.93535 | -1.22256 |  |  |  |  |
| H | -1.1678 | -4.1674 | -5.57694 |  |  |  |  |
| H | 0.43972 | -6.81894 | -6.73495 |  |  |  |  |
| H | -3.74488 | -6.89677 | -2.66362 |  |  |  |  |
| H | -0.94792 | -9.89753 | -8.51266 |  |  |  |  |
| H | -2.45083 | -12.7595 | -7.63349 |  |  |  |  |
| H | -4.07693 | -10.5422 | -9.50143 |  |  |  |  |
| H | -7.67099 | -10.7416 | -6.04741 |  |  |  |  |
| H | -5.84344 | -12.7579 | -4.13261 |  |  |  |  |
| H | -6.89225 | -9.80607 | -2.91816 |  |  |  |  |
| H | 3.83968 | -0.08481 | -5.30037 |  |  |  |  |
| H | 5.73913 | -2.81954 | -5.35662 |  |  |  |  |
| H | 2.75948 | -2.82153 | -6.87997 |  |  |  |  |
| H | 6.62678 | 0.33816 | -1.70662 |  |  |  |  |
| H | 5.99198 | 0.38581 | 1.54261 |  |  |  |  |
| H | 3.74695 | 9.38841 | -0.23351 |  |  |  |  |
| H | 1.00908 | 9.92336 | -2.05595 |  |  |  |  |
| H | -1.66995 | 9.55395 | 1.98224 |  |  |  |  |
| H | -2.39247 | 11.7574 | 5.62538 |  |  |  |  |
| H | 0.27197 | 11.86 | 7.66659 |  |  |  |  |
| H | -0.54864 | 14.5496 | 5.88968 |  |  |  |  |
| H | 3.91553 | 14.615 | 3.79356 |  |  |  |  |
| H | 4.73694 | 12.0196 | 5.69898 |  |  |  |  |
| H | 5.11031 | 11.8347 | 2.37263 |  |  |  |  |
| H | -4.14819 | 6.03246 | -2.96419 |  |  |  |  |
| H | -2.66083 | -0.85481 | 1.69899 |  |  |  |  |
| H | -4.85646 | -4.60003 | 3.43506 |  |  |  |  |
| H | -8.43665 | -6.51594 | 1.09454 |  |  |  |  |
| H | -9.80675 | -4.67592 | -2.99655 |  |  |  |  |
| H | -7.67787 | -0.82875 | -4.65615 |  |  |  |  |
| H | 7.18225 | 6.73214 | 3.93401 |  |  |  |  |
| H | 3.86183 | 7.25155 | 3.83844 |  |  |  |  |
| H | 6.66942 | 2.45724 | 5.57032 |  |  |  |  |
| H | 4.46305 | -0.68244 | 7.60248 |  |  |  |  |
| H | 1.7935 | 0.48291 | 9.27966 |  |  |  |  |
| H | 1.35463 | -1.11903 | 6.40139 |  |  |  |  |
| H | -0.90059 | 3.86493 | 7.26671 |  |  |  |  |
| H | 0.38839 | 5.86668 | 4.89685 |  |  |  |  |
| H | -0.8207 | 2.87199 | 4.08565 |  |  |  |  |

**ECD calculation details of 4.**

**Figure S45.** Optimized geometries of predominant conformers for **4** at the B3LYP-D3(BJ)/6-31G* level in PCM MeOH using ORCA5.0.3.

**Table S19.** Gibbs free energies^a^ and equilibrium populations^b^ of low-energy conformers of **4**.

| Conformers | ∆G(a.u.) | P(%)/100 | G(a.u.) |
| --- | --- | --- | --- |
| **4-1** | 0.00392 | 1.23 | -1733.533039 |
| **4-2** | 0.00598 | 0.14 | -1733.530978 |
| **4-3** | 0.00409 | 1.02 | -1733.532863 |
| **4-4** | 0.00339 | 2.14 | -1733.533565 |
| **4-5** | 0.00859 | 0.01 | -1733.528367 |
| **4-6** | 0.00545 | 0.24 | -1733.531509 |
| **4-7** | 0.00557 | 0.21 | -1733.531388 |
| **4-8** | 0.00533 | 0.27 | -1733.531626 |
| **4-9** | 0.00601 | 0.13 | -1733.530948 |
| **4-10** | 0.00655 | 0.07 | -1733.530405 |
| **4-11** | 0.00389 | 1.26 | -1733.533067 |
| **4-12** | 0.00592 | 0.15 | -1733.531032 |
| **4-13** | 0.00722 | 0.04 | -1733.529739 |
| **4-14** | 0.00758 | 0.03 | -1733.529374 |
| **4-15** | 0.0016 | 14.19 | -1733.535351 |
| **4-16** | 0.0 | 77.66 | -1733.536956 |
| **4-17** | 0.00604 | 0.13 | -1733.530918 |
| **4-18** | 0.00742 | 0.03 | -1733.529539 |
| **4-19** | 0.00594 | 0.14 | -1733.531011 |
| **4-20** | 0.00733 | 0.03 | -1733.529626 |
| **4-21** | 0.00588 | 0.15 | -1733.531076 |
| **4-22** | 0.00758 | 0.03 | -1733.529376 |
| **4-23** | 0.00654 | 0.08 | -1733.530413 |
| **4-24** | 0.00777 | 0.02 | -1733.529181 |
| **4-25** | 0.00724 | 0.04 | -1733.529712 |
| **4-26** | 0.00626 | 0.1 | -1733.530691 |
| **4-27** | 0.01013 | 0.0 | -1733.526824 |
| **4-28** | 0.00516 | 0.33 | -1733.5318 |
| **4-29** | 0.00602 | 0.13 | -1733.530938 |

^a^wB97M-V/def2-TZVP, in a.u.
^b^From ∆G values at 298.15K.

**Table S20.** Cartesian coordinates for the low-energy reoptimized random research conformers of **4** at B3LYP-D3(BJ)/6-31G* level of theory in MeOH.

| **4-1** | | | | **4-2** | | | |
| --- | --- | --- | --- | --- | --- | --- | --- |
| Atom | X | Y | Z | Atom | X | Y | Z |
| C | -20.3297 | 1.46881 | 0.96332 | C | -21.4997 | -1.12237 | 0.66242 |
| C | -20.6375 | -1.06549 | 0.6966 | C | -21.0914 | -3.04894 | 2.30868 |
| C | -19.4021 | -2.78363 | 2.54534 | C | -18.7099 | -3.07589 | 3.80368 |
| C | -18.0459 | -1.56549 | 4.58906 | C | -17.1428 | -0.84078 | 3.57322 |
| C | -17.8331 | 0.97127 | 4.79991 | C | -17.7108 | 1.09784 | 2.00901 |
| C | -18.7091 | 2.80166 | 2.87266 | C | -19.7149 | 0.99735 | 0.0551 |
| C | -22.6052 | -2.03976 | -1.07036 | C | -23.2507 | -4.73051 | 2.99498 |
| O | -24.718 | -1.0549 | -1.11325 | O | -25.3214 | -3.78535 | 3.49679 |
| C | -21.9996 | -4.19785 | -2.75567 | C | -22.8997 | -7.50992 | 3.06052 |
| C | -23.985 | -5.52902 | -3.91627 | C | -20.8298 | -8.66733 | 1.86663 |
| C | -23.4658 | -7.55429 | -5.5148 | C | -20.6011 | -11.2928 | 1.86841 |
| C | -20.9573 | -8.24774 | -5.99537 | C | -22.4222 | -12.7775 | 3.07987 |
| C | -18.9718 | -6.9064 | -4.88134 | C | -24.489 | -11.6363 | 4.27554 |
| C | -19.4877 | -4.89409 | -3.26064 | C | -24.7349 | -9.0159 | 4.25379 |
| C | -20.6113 | 4.8573 | 3.69319 | C | -21.62 | 3.20203 | -0.08866 |
| C | -22.0828 | 5.38634 | 1.22828 | C | -24.0721 | 1.91264 | -0.99767 |
| O | -21.563 | 3.19932 | -0.39337 | O | -23.6387 | -0.79515 | -0.62693 |
| C | -24.9885 | 5.46762 | 1.50513 | C | -26.4575 | 2.53678 | 0.56736 |
| C | -26.2096 | 5.94268 | -1.07462 | C | -28.7321 | 1.08434 | -0.47646 |
| C | -25.7535 | 7.50372 | 3.39497 | C | -26.9539 | 5.37476 | 0.53801 |
| O | -25.8202 | 3.0992 | 2.4945 | O | -26.0053 | 1.8241 | 3.13085 |
| C | -16.3497 | 4.02218 | 1.51908 | C | -18.4652 | 0.40391 | -2.56949 |
| C | -15.3279 | 2.39229 | -0.55715 | C | -16.5631 | 2.32599 | -3.40197 |
| C | -16.7865 | -2.78471 | 6.82485 | C | -14.7478 | -0.21702 | 4.97022 |
| C | -15.6142 | -0.4726 | 8.18086 | C | -14.1252 | 2.46452 | 3.96562 |
| O | -16.5717 | 1.76938 | 6.84216 | O | -16.1593 | 3.08199 | 2.15982 |
| C | -12.6934 | -0.38148 | 8.20547 | C | -11.6102 | 2.72449 | 2.51948 |
| C | -11.574 | -0.43998 | 5.54943 | C | -11.3324 | 5.40614 | 1.46741 |
| C | -11.7925 | 1.94898 | 9.6544 | C | -9.39748 | 2.07978 | 4.24948 |
| O | -11.8482 | -2.63211 | 9.43925 | O | -11.5807 | 0.93433 | 0.49804 |
| O | -19.648 | -5.1103 | 2.45203 | O | -18.1812 | -4.80882 | 5.28791 |
| C | -15.4521 | 2.89836 | -3.04792 | C | -14.3757 | 1.88689 | -4.62871 |
| C | -14.6118 | 0.95818 | -4.96555 | C | -12.828 | 4.08517 | -5.58722 |
| C | -16.5135 | 5.31145 | -4.13029 | C | -13.3959 | -0.6909 | -5.32195 |
| C | -16.7128 | 0.25281 | -6.8451 | C | -13.492 | 4.78215 | -8.36022 |
| C | -15.979 | -1.88221 | -8.5808 | C | -16.0981 | 5.88107 | -8.59968 |
| C | -17.5605 | -3.24494 | -10.0323 | C | -18.2463 | 4.66304 | -9.21177 |
| C | -16.6221 | -5.36715 | -11.6762 | C | -20.7592 | 6.00127 | -9.18191 |
| C | -20.3633 | -2.77784 | -10.1538 | C | -18.3849 | 1.90762 | -9.90555 |
| H | -25.9311 | -4.9685 | -3.5247 | H | -19.4149 | -7.51197 | 0.91092 |
| H | -25.0143 | -8.6 | -6.39312 | H | -18.9978 | -12.1867 | 0.9237 |
| H | -20.5504 | -9.83557 | -7.25129 | H | -22.2329 | -14.8342 | 3.09078 |
| H | -17.0163 | -7.42678 | -5.28176 | H | -25.9086 | -12.7994 | 5.22169 |
| H | -17.9352 | -3.84391 | -2.40457 | H | -26.3415 | -8.09982 | 5.16731 |
| H | -19.6752 | 6.55252 | 4.43241 | H | -21.0123 | 4.70729 | -1.37532 |
| H | -21.8877 | 4.09013 | 5.13794 | H | -21.8918 | 3.99877 | 1.80658 |
| H | -21.3922 | 7.0665 | 0.22615 | H | -24.4263 | 2.2036 | -3.02157 |
| H | -28.2807 | 5.91876 | -0.88304 | H | -28.3806 | -0.96442 | -0.43753 |
| H | -25.6611 | 4.46548 | -2.43095 | H | -29.1446 | 1.6392 | -2.43864 |
| H | -25.6389 | 7.78665 | -1.85073 | H | -30.409 | 1.48116 | 0.68837 |
| H | -24.996 | 7.10571 | 5.28987 | H | -28.6844 | 5.79335 | 1.61372 |
| H | -25.0675 | 9.37146 | 2.78989 | H | -25.3883 | 6.43178 | 1.40606 |
| H | -27.8274 | 7.58117 | 3.53657 | H | -27.2107 | 6.05984 | -1.40866 |
| H | -25.5114 | 1.78652 | 1.22671 | H | -25.8724 | -0.01863 | 3.21943 |
| H | -16.9259 | 5.87394 | 0.79352 | H | -17.6312 | -1.49358 | -2.46919 |
| H | -14.9338 | 4.36987 | 3.0041 | H | -20.0162 | 0.30729 | -3.96238 |
| H | -14.5541 | 0.56251 | 0.02929 | H | -17.0748 | 4.30042 | -3.05971 |
| H | -18.1524 | -3.77658 | 8.04304 | H | -14.9887 | -0.22833 | 7.03591 |
| H | -15.3385 | -4.1744 | 6.28406 | H | -13.2426 | -1.57542 | 4.50525 |
| H | -16.2857 | -0.29685 | 10.1387 | H | -14.2285 | 3.90453 | 5.45773 |
| H | -9.49845 | -0.47172 | 5.67978 | H | -12.9253 | 5.88554 | 0.22267 |
| H | -12.1721 | -2.13345 | 4.50375 | H | -11.2616 | 6.81027 | 3.0008 |
| H | -12.1297 | 1.24301 | 4.46868 | H | -9.57521 | 5.54511 | 0.36367 |
| H | -12.4349 | 3.70046 | 8.73716 | H | -7.61339 | 2.29228 | 3.2011 |
| H | -9.71448 | 1.97327 | 9.74624 | H | -9.50894 | 0.11978 | 4.93305 |
| H | -12.5362 | 1.92907 | 11.6006 | H | -9.34592 | 3.34952 | 5.89538 |
| H | -12.3676 | -2.55046 | 11.1956 | H | -12.8871 | 1.42518 | -0.70412 |
| H | -12.983 | 1.69779 | -6.04902 | H | -13.1391 | 5.75681 | -4.38675 |
| H | -13.9495 | -0.75607 | -3.98479 | H | -10.799 | 3.62117 | -5.47742 |
| H | -15.3868 | 5.93696 | -5.76823 | H | -14.6199 | -2.24143 | -4.68694 |
| H | -18.4618 | 5.02913 | -4.81739 | H | -13.1821 | -0.85666 | -7.38994 |
| H | -16.5592 | 6.86943 | -2.76042 | H | -11.4975 | -0.98598 | -4.5101 |
| H | -18.4374 | -0.24143 | -5.78185 | H | -12.0902 | 6.17672 | -9.02069 |
| H | -17.2193 | 1.93735 | -7.96914 | H | -13.2635 | 3.09872 | -9.56104 |
| H | -13.9588 | -2.34795 | -8.65416 | H | -16.2601 | 7.8819 | -8.07357 |
| H | -17.506 | -7.17618 | -11.1211 | H | -22.0516 | 5.10666 | -7.80512 |
| H | -17.1406 | -5.05961 | -13.6757 | H | -20.574 | 8.01132 | -8.67964 |
| H | -14.5571 | -5.59314 | -11.5668 | H | -21.7099 | 5.87373 | -11.0366 |
| H | -21.0359 | -1.44276 | -8.71374 | H | -16.5271 | 0.99325 | -10.0272 |
| H | -20.8931 | -2.01136 | -12.0238 | H | -19.5172 | 0.86108 | -8.49857 |
| H | -21.4207 | -4.55971 | -9.93332 | H | -19.3554 | 1.65656 | -11.7364 |
| **4-3** | | | | **4-4** | | | |
| Atom | X | Y | Z | Atom | X | Y | Z |
| C | -21.4181 | 1.7884 | 1.01857 | C | -20.6995 | 1.52416 | 0.76221 |
| C | -21.5046 | -0.71315 | 0.4506 | C | -20.8836 | -1.03459 | 0.87652 |
| C | -19.4471 | -2.37899 | 1.39667 | C | -19.4825 | -2.40358 | 2.89245 |
| C | -17.7033 | -1.21866 | 3.16267 | C | -18.1305 | -0.83471 | 4.68089 |
| C | -17.7531 | 1.26848 | 3.74693 | C | -18.0719 | 1.71358 | 4.53664 |
| C | -19.3913 | 3.14606 | 2.46872 | C | -19.0972 | 3.19661 | 2.39829 |
| C | -23.9236 | -1.80625 | -0.52159 | C | -22.86 | -2.33868 | -0.65374 |
| O | -25.9323 | -1.21935 | 0.50718 | O | -25.0179 | -1.45923 | -0.74121 |
| C | -23.911 | -3.57517 | -2.69081 | C | -22.2144 | -4.67549 | -2.06438 |
| C | -26.1087 | -4.95922 | -3.25424 | C | -24.1788 | -6.15527 | -3.07111 |
| C | -26.154 | -6.58122 | -5.32648 | C | -23.6307 | -8.34441 | -4.42535 |
| C | -24.014 | -6.8188 | -6.86721 | C | -21.1132 | -9.06784 | -4.80237 |
| C | -21.8282 | -5.43339 | -6.32971 | C | -19.1475 | -7.58976 | -3.83593 |
| C | -21.7756 | -3.82419 | -4.24406 | C | -19.6925 | -5.4028 | -2.47108 |
| C | -21.0835 | 4.83911 | 4.14158 | C | -21.0687 | 5.2703 | 2.96663 |
| C | -23.4268 | 5.31382 | 2.47275 | C | -22.6267 | 5.36328 | 0.50114 |
| O | -23.2683 | 3.41309 | 0.4677 | O | -22.0597 | 2.97642 | -0.78713 |
| C | -25.9938 | 4.86079 | 3.78969 | C | -25.5234 | 5.3818 | 0.85943 |
| C | -28.1557 | 5.26725 | 1.90892 | C | -26.8377 | 5.41146 | -1.71812 |
| C | -26.265 | 6.61717 | 6.05808 | C | -26.3135 | 7.65797 | 2.43984 |
| O | -26.0475 | 2.33024 | 4.72039 | O | -26.2319 | 3.16733 | 2.22786 |
| C | -17.7396 | 4.81387 | 0.64368 | C | -16.8266 | 4.30392 | 0.81247 |
| C | -16.0493 | 3.34528 | -1.09038 | C | -15.6676 | 2.4153 | -0.94958 |
| C | -15.5818 | -2.45786 | 4.58911 | C | -16.6294 | -1.6591 | 6.9457 |
| C | -14.6573 | -0.26562 | 6.29363 | C | -15.5298 | 0.88553 | 7.90098 |
| O | -16.0206 | 2.00515 | 5.43411 | O | -16.7781 | 2.85557 | 6.38037 |
| C | -11.7928 | 0.27546 | 6.29837 | C | -12.66 | 1.19364 | 7.58048 |
| C | -10.8203 | 1.08901 | 3.70401 | C | -11.8396 | 3.88578 | 8.24373 |
| C | -11.1873 | 2.26765 | 8.29211 | C | -11.2776 | -0.7212 | 9.25039 |
| O | -10.5779 | -1.99944 | 7.11036 | O | -12.1624 | 0.69642 | 4.96492 |
| O | -19.3153 | -4.65363 | 0.85506 | O | -19.5822 | -4.73113 | 3.13147 |
| C | -16.5321 | 2.62606 | -3.48215 | C | -15.9043 | 2.39635 | -3.48414 |
| C | -14.584 | 1.10983 | -4.92149 | C | -14.7966 | 0.26448 | -5.02822 |
| C | -18.9527 | 3.18888 | -4.86564 | C | -17.3086 | 4.3793 | -4.97049 |
| C | -15.3642 | -1.6638 | -5.37135 | C | -16.7378 | -1.02898 | -6.75852 |
| C | -13.3426 | -3.17951 | -6.65891 | C | -15.711 | -3.36022 | -8.03495 |
| C | -13.4201 | -4.25003 | -8.96633 | C | -17.0563 | -5.02005 | -9.41327 |
| C | -11.1998 | -5.71966 | -9.97657 | C | -15.8494 | -7.31087 | -10.5893 |
| C | -15.6477 | -4.12098 | -10.7342 | C | -19.8422 | -4.73044 | -9.90969 |
| H | -27.7647 | -4.75024 | -2.04257 | H | -26.1321 | -5.57073 | -2.75959 |
| H | -27.8581 | -7.66719 | -5.75019 | H | -25.1638 | -9.49713 | -5.18929 |
| H | -24.054 | -8.08966 | -8.49493 | H | -20.6837 | -10.7867 | -5.86315 |
| H | -20.1622 | -5.60936 | -7.53454 | H | -17.1852 | -8.13888 | -4.15392 |
| H | -20.0798 | -2.74281 | -3.82499 | H | -18.1536 | -4.247 | -1.73866 |
| H | -20.1354 | 6.60106 | 4.68185 | H | -20.1935 | 7.0939 | 3.42005 |
| H | -21.6235 | 3.8013 | 5.85413 | H | -22.2714 | 4.67004 | 4.5474 |
| H | -23.3968 | 7.17465 | 1.55554 | H | -22.0358 | 6.89713 | -0.76487 |
| H | -28.177 | 7.22041 | 1.19228 | H | -26.2841 | 3.7563 | -2.84846 |
| H | -29.9751 | 4.88754 | 2.84272 | H | -26.3561 | 7.12623 | -2.79352 |
| H | -27.9716 | 3.97892 | 0.28815 | H | -28.9 | 5.35841 | -1.45121 |
| H | -24.7855 | 6.26665 | 7.47642 | H | -28.3826 | 7.67543 | 2.65377 |
| H | -26.1538 | 8.60887 | 5.47007 | H | -25.4725 | 7.59314 | 4.33981 |
| H | -28.1101 | 6.30443 | 6.96673 | H | -25.7346 | 9.43261 | 1.5231 |
| H | -26.0674 | 1.16637 | 3.27958 | H | -25.8861 | 1.68545 | 1.17479 |
| H | -19.0347 | 6.0583 | -0.39872 | H | -17.5329 | 5.95188 | -0.22347 |
| H | -16.591 | 6.02254 | 1.88732 | H | -15.4346 | 4.99893 | 2.19325 |
| H | -14.2322 | 2.78073 | -0.28163 | H | -14.624 | 0.8681 | -0.05106 |
| H | -16.2091 | -4.08229 | 5.72309 | H | -17.8114 | -2.57818 | 8.3917 |
| H | -14.1027 | -3.1529 | 3.29577 | H | -15.1336 | -3.0028 | 6.42121 |
| H | -15.2572 | -0.57321 | 8.25996 | H | -16.0302 | 1.27505 | 9.8753 |
| H | -8.75258 | 1.29965 | 3.77979 | H | -9.78635 | 4.08366 | 7.95977 |
| H | -11.2709 | -0.31595 | 2.23675 | H | -12.8049 | 5.27573 | 7.04036 |
| H | -11.6444 | 2.90949 | 3.13982 | H | -12.2579 | 4.32984 | 10.2329 |
| H | -12.2094 | 4.034 | 7.9013 | H | -11.7843 | -2.6743 | 8.75184 |
| H | -9.14684 | 2.67082 | 8.31206 | H | -11.7152 | -0.41866 | 11.262 |
| H | -11.7423 | 1.58839 | 10.1794 | H | -9.22177 | -0.50659 | 9.00174 |
| H | -10.5974 | -3.19899 | 5.72571 | H | -10.3742 | 0.95561 | 4.66558 |
| H | -14.2412 | 1.99954 | -6.77971 | H | -13.2451 | 1.00182 | -6.22221 |
| H | -12.7744 | 1.14613 | -3.8906 | H | -13.9448 | -1.14795 | -3.75579 |
| H | -20.0267 | 1.43957 | -5.22206 | H | -17.6698 | 6.11296 | -3.88988 |
| H | -20.2062 | 4.47899 | -3.83093 | H | -16.2402 | 4.90214 | -6.68281 |
| H | -18.5494 | 4.02315 | -6.73438 | H | -19.1533 | 3.65606 | -5.61767 |
| H | -15.7837 | -2.52279 | -3.51443 | H | -18.4305 | -1.51414 | -5.6401 |
| H | -17.1285 | -1.7122 | -6.46072 | H | -17.3979 | 0.33285 | -8.19464 |
| H | -11.5918 | -3.40411 | -5.56648 | H | -13.6807 | -3.7177 | -7.80495 |
| H | -11.7486 | -7.68155 | -10.4368 | H | -16.155 | -7.34799 | -12.655 |
| H | -10.4965 | -4.88402 | -11.7567 | H | -13.8014 | -7.39068 | -10.2331 |
| H | -9.62138 | -5.78812 | -8.62346 | H | -16.7088 | -9.06354 | -9.84642 |
| H | -16.3929 | -6.03314 | -11.1182 | H | -20.7477 | -3.31593 | -8.68866 |
| H | -17.2076 | -2.95443 | -10.0203 | H | -20.1763 | -4.16214 | -11.8927 |
| H | -15.0638 | -3.34848 | -12.5833 | H | -20.8358 | -6.54292 | -9.64686 |
| **4-5** | | | | **4-6** | | | |
| Atom | X | Y | Z | Atom | X | Y | Z |
| C | -21.6117 | -0.49582 | 0.97204 | C | -21.9145 | -1.41433 | 0.47643 |
| C | -21.6891 | -2.68451 | 2.31042 | C | -21.5375 | -3.19954 | 2.28159 |
| C | -19.686 | -3.19542 | 4.21236 | C | -19.3999 | -2.88485 | 4.07955 |
| C | -17.9292 | -1.1441 | 4.65595 | C | -18.0499 | -0.50785 | 3.90764 |
| C | -17.9857 | 1.04079 | 3.33316 | C | -18.577 | 1.26454 | 2.14431 |
| C | -19.5687 | 1.4642 | 1.06121 | C | -20.2772 | 0.84562 | -0.04239 |
| C | -24.0679 | -4.20341 | 2.31809 | C | -23.5803 | -5.07687 | 2.79303 |
| O | -26.1067 | -3.13407 | 2.6792 | O | -25.7871 | -4.34903 | 2.97222 |
| C | -23.9562 | -6.96911 | 1.88756 | C | -22.9452 | -7.79181 | 3.07724 |
| C | -26.0987 | -8.45047 | 2.41266 | C | -20.6285 | -8.76427 | 2.21833 |
| C | -26.0601 | -11.0427 | 1.9593 | C | -20.1199 | -11.3419 | 2.42267 |
| C | -23.8892 | -12.1741 | 0.95352 | C | -21.9076 | -12.9604 | 3.50567 |
| C | -21.7573 | -10.7094 | 0.40763 | C | -24.2195 | -12.0037 | 4.36778 |
| C | -21.7838 | -8.11709 | 0.8809 | C | -24.7424 | -9.43408 | 4.14147 |
| C | -21.193 | 3.88453 | 0.94748 | C | -22.3506 | 2.83071 | -0.56402 |
| C | -23.6062 | 3.04357 | -0.46026 | C | -24.5461 | 1.24834 | -1.65289 |
| O | -23.4582 | 0.28336 | -0.55921 | O | -23.8886 | -1.37951 | -1.0881 |
| C | -26.1205 | 3.64568 | 0.89822 | C | -27.1407 | 1.69048 | -0.38356 |
| C | -28.3448 | 2.65599 | -0.66545 | C | -29.137 | -0.02453 | -1.5849 |
| C | -26.3584 | 6.48521 | 1.32751 | C | -27.8997 | 4.46069 | -0.61031 |
| O | -26.0979 | 2.46008 | 3.32355 | O | -26.9095 | 1.13231 | 2.24697 |
| C | -17.7399 | 1.18941 | -1.2791 | C | -18.6427 | 0.23775 | -2.44735 |
| C | -18.9446 | 1.51402 | -3.81701 | C | -16.812 | 2.27746 | -3.15398 |
| C | -15.8171 | -1.0104 | 6.55043 | C | -15.9614 | 0.45407 | 5.57737 |
| C | -14.5806 | 1.56979 | 5.90197 | C | -15.5314 | 3.15535 | 4.53156 |
| O | -16.2556 | 2.7476 | 4.01483 | O | -17.2731 | 3.41383 | 2.36773 |
| C | -11.9209 | 1.41374 | 4.74732 | C | -12.8559 | 3.71964 | 3.53619 |
| C | -11.0383 | 4.01932 | 3.85262 | C | -12.7504 | 6.37897 | 2.39929 |
| C | -10.0679 | 0.33775 | 6.6896 | C | -10.93 | 3.44604 | 5.66179 |
| O | -12.1578 | -0.25944 | 2.63309 | O | -12.2221 | 1.88471 | 1.65787 |
| O | -19.6232 | -5.17858 | 5.45945 | O | -18.9184 | -4.46368 | 5.74119 |
| C | -18.7552 | 3.5392 | -5.34447 | C | -14.3801 | 1.97432 | -3.84998 |
| C | -19.9627 | 3.46507 | -7.94299 | C | -12.8293 | 4.23926 | -4.6349 |
| C | -17.3457 | 5.92067 | -4.70122 | C | -13.0608 | -0.54304 | -4.02554 |
| C | -18.3567 | 1.99168 | -9.89487 | C | -12.1315 | 4.25825 | -7.47379 |
| C | -15.7588 | 3.07099 | -10.2741 | C | -14.4108 | 4.10589 | -9.17226 |
| C | -13.5571 | 2.19755 | -9.3441 | C | -15.9069 | 6.0388 | -9.87666 |
| C | -11.1155 | 3.58308 | -9.82161 | C | -18.2106 | 5.61718 | -11.4937 |
| C | -13.2944 | -0.12929 | -7.72607 | C | -15.4677 | 8.74024 | -9.09653 |
| H | -27.7795 | -7.54054 | 3.18831 | H | -19.2371 | -7.50615 | 1.36249 |
| H | -27.7218 | -12.1912 | 2.38648 | H | -18.3234 | -12.0937 | 1.7372 |
| H | -23.8611 | -14.2075 | 0.59223 | H | -21.4996 | -14.9778 | 3.67705 |
| H | -20.0702 | -11.5931 | -0.38902 | H | -25.6121 | -13.2711 | 5.21488 |
| H | -20.1252 | -6.97306 | 0.44315 | H | -26.5393 | -8.66125 | 4.79656 |
| H | -20.225 | 5.43214 | -0.03037 | H | -21.7351 | 4.31089 | -1.87572 |
| H | -21.6589 | 4.4875 | 2.87597 | H | -22.9228 | 3.71091 | 1.2244 |
| H | -23.6586 | 3.71668 | -2.41875 | H | -24.7069 | 1.40752 | -3.71513 |
| H | -28.1814 | 0.60533 | -0.96605 | H | -28.5929 | -2.02388 | -1.4138 |
| H | -28.4274 | 3.58334 | -2.52587 | H | -29.3918 | 0.41246 | -3.60297 |
| H | -30.1279 | 3.02923 | 0.33854 | H | -30.9591 | 0.2412 | -0.61761 |
| H | -28.1826 | 6.90778 | 2.2337 | H | -29.774 | 4.74563 | 0.2459 |
| H | -24.8468 | 7.19698 | 2.56425 | H | -26.5511 | 5.70187 | 0.37058 |
| H | -26.2686 | 7.51242 | -0.47847 | H | -27.9957 | 5.0399 | -2.60538 |
| H | -26.1846 | 0.62755 | 3.08597 | H | -26.6404 | -0.68731 | 2.44618 |
| H | -16.2278 | 2.57696 | -0.97803 | H | -17.6715 | -1.56594 | -2.11656 |
| H | -16.8702 | -0.69634 | -1.1414 | H | -19.984 | -0.08185 | -4.01233 |
| H | -20.067 | -0.09137 | -4.48909 | H | -17.5687 | 4.20417 | -3.1566 |
| H | -16.5029 | -1.0624 | 8.51481 | H | -16.4791 | 0.48221 | 7.5909 |
| H | -14.4828 | -2.58608 | 6.31395 | H | -14.2547 | -0.71998 | 5.38113 |
| H | -14.5374 | 2.86013 | 7.52495 | H | -16.0932 | 4.61841 | 5.89379 |
| H | -12.3282 | 4.79189 | 2.42009 | H | -14.0961 | 6.56891 | 0.82723 |
| H | -10.9342 | 5.36075 | 5.43975 | H | -13.2116 | 7.82154 | 3.82596 |
| H | -9.13912 | 3.86791 | 3.01281 | H | -10.8379 | 6.75959 | 1.67635 |
| H | -10.6348 | -1.55969 | 7.32125 | H | -9.02542 | 3.84675 | 4.92757 |
| H | -9.92657 | 1.57356 | 8.35755 | H | -10.9254 | 1.5192 | 6.44157 |
| H | -8.17427 | 0.19278 | 5.83645 | H | -11.3323 | 4.7812 | 7.20417 |
| H | -10.5111 | -0.42969 | 1.85054 | H | -13.4186 | 2.07106 | 0.27185 |
| H | -21.8358 | 2.55939 | -7.83272 | H | -13.8764 | 5.98224 | -4.19252 |
| H | -20.2725 | 5.40791 | -8.63107 | H | -11.0686 | 4.27619 | -3.51417 |
| H | -16.271 | 5.79547 | -2.93038 | H | -14.0218 | -2.04357 | -2.96027 |
| H | -16.0268 | 6.42453 | -6.23319 | H | -12.9291 | -1.17316 | -6.01039 |
| H | -18.6787 | 7.51781 | -4.53008 | H | -11.1081 | -0.39572 | -3.31549 |
| H | -18.2554 | 0.00452 | -9.2896 | H | -11.0348 | 5.98878 | -7.84823 |
| H | -19.3813 | 2.01105 | -11.7134 | H | -10.857 | 2.66185 | -7.87941 |
| H | -15.6896 | 4.82856 | -11.3755 | H | -14.9504 | 2.20695 | -9.80388 |
| H | -9.72618 | 2.35612 | -10.7836 | H | -19.9341 | 6.29273 | -10.5268 |
| H | -11.3947 | 5.28539 | -10.9839 | H | -18.0873 | 6.69779 | -13.2767 |
| H | -10.228 | 4.1631 | -8.02204 | H | -18.4787 | 3.60943 | -11.967 |
| H | -11.8412 | -1.40875 | -8.50581 | H | -15.5419 | 10.0119 | -10.7494 |
| H | -12.6436 | 0.3902 | -5.81112 | H | -16.9777 | 9.37854 | -7.80278 |
| H | -15.0601 | -1.19933 | -7.52115 | H | -13.6502 | 9.03179 | -8.13644 |
| **4-7** | | | | **4-8** | | | |
| Atom | X | Y | Z | Atom | X | Y | Z |
| C | -20.5996 | 1.58539 | 0.74068 | C | -21.4699 | -2.58894 | 0.50731 |
| C | -20.7301 | -0.97571 | 0.8708 | C | -21.1489 | -3.79816 | 2.74754 |
| C | -19.2586 | -2.30684 | 2.86097 | C | -19.0449 | -3.00028 | 4.43237 |
| C | -17.8879 | -0.70408 | 4.60441 | C | -17.6567 | -0.78263 | 3.62174 |
| C | -17.8766 | 1.84388 | 4.4435 | C | -18.0904 | 0.39884 | 1.39629 |
| C | -18.9887 | 3.29657 | 2.32745 | C | -19.8058 | -0.57392 | -0.59384 |
| C | -22.7138 | -2.32696 | -0.60832 | C | -23.2337 | -5.42536 | 3.734 |
| O | -24.8911 | -1.49346 | -0.64915 | O | -25.4273 | -4.641 | 3.67056 |
| C | -22.0513 | -4.6551 | -2.0257 | C | -22.6551 | -7.9624 | 4.77385 |
| C | -24.0068 | -6.17511 | -2.98867 | C | -20.3322 | -9.15477 | 4.29147 |
| C | -23.4447 | -8.35593 | -4.3506 | C | -19.8732 | -11.5794 | 5.21656 |
| C | -20.922 | -9.03138 | -4.77834 | C | -21.719 | -12.8207 | 6.64483 |
| C | -18.9658 | -7.51393 | -3.85453 | C | -24.0382 | -11.6433 | 7.13375 |
| C | -19.5246 | -5.33471 | -2.48287 | C | -24.5098 | -9.23141 | 6.19169 |
| C | -20.986 | 5.33348 | 2.93884 | C | -21.867 | 1.22978 | -1.58805 |
| C | -22.6107 | 5.38246 | 0.51498 | C | -24.0465 | -0.57017 | -2.2934 |
| O | -22.0257 | 3.00411 | -0.78042 | O | -23.4388 | -2.95618 | -1.02616 |
| C | -25.497 | 5.33961 | 0.94947 | C | -26.6714 | 0.2075 | -1.27942 |
| C | -26.8782 | 5.33572 | -1.59307 | C | -28.6484 | -1.76948 | -2.02376 |
| C | -26.2945 | 7.60174 | 2.54622 | C | -27.3885 | 2.80855 | -2.29122 |
| O | -26.1214 | 3.11399 | 2.34056 | O | -26.5298 | 0.39992 | 1.40841 |
| C | -16.7852 | 4.44183 | 0.6759 | C | -18.2189 | -1.78961 | -2.80589 |
| C | -15.6249 | 2.56429 | -1.09679 | C | -16.6128 | 0.07789 | -4.19768 |
| C | -16.3174 | -1.48744 | 6.8367 | C | -15.6072 | 0.60914 | 5.01415 |
| C | -15.2091 | 1.07798 | 7.72367 | C | -15.1429 | 2.9144 | 3.2722 |
| O | -16.5485 | 3.01822 | 6.24262 | O | -16.7308 | 2.49277 | 1.0195 |
| C | -12.3596 | 1.41658 | 7.2723 | C | -12.3973 | 3.28575 | 2.39081 |
| C | -11.5442 | 4.1271 | 7.86245 | C | -12.2407 | 5.38748 | 0.41028 |
| C | -10.8802 | -0.45884 | 8.90289 | C | -10.7449 | 3.90235 | 4.67315 |
| O | -11.9725 | 0.88946 | 4.64423 | O | -11.4501 | 0.97218 | 1.36607 |
| O | -19.3143 | -4.63425 | 3.11633 | O | -18.6244 | -4.04178 | 6.48811 |
| C | -15.917 | 2.5187 | -3.62504 | C | -17.1065 | 1.1862 | -6.43523 |
| C | -14.7925 | 0.40072 | -5.17593 | C | -15.2846 | 3.12375 | -7.47462 |
| C | -17.4002 | 4.45598 | -5.09515 | C | -19.4259 | 0.68802 | -8.00335 |
| C | -16.7381 | -0.95329 | -6.8535 | C | -16.3519 | 5.84175 | -7.43988 |
| C | -15.6763 | -3.25976 | -8.14655 | C | -14.4617 | 7.74201 | -8.36831 |
| C | -17.0027 | -4.95186 | -9.50369 | C | -13.3585 | 9.62769 | -7.06323 |
| C | -15.758 | -7.20909 | -10.7051 | C | -11.4438 | 11.3378 | -8.29683 |
| C | -19.8032 | -4.73109 | -9.9529 | C | -13.883 | 10.2307 | -4.3316 |
| H | -25.9641 | -5.62755 | -2.63757 | H | -18.8957 | -8.19456 | 3.16666 |
| H | -24.9707 | -9.53944 | -5.08099 | H | -18.0702 | -12.5057 | 4.82486 |
| H | -20.4811 | -10.7437 | -5.84509 | H | -21.351 | -14.7157 | 7.37931 |
| H | -17.0001 | -8.02592 | -4.21141 | H | -25.4766 | -12.6152 | 8.2514 |
| H | -17.9932 | -4.14843 | -1.78439 | H | -26.3117 | -8.29394 | 6.5505 |
| H | -20.1361 | 7.17673 | 3.35987 | H | -21.2191 | 2.36248 | -3.19607 |
| H | -22.134 | 4.71807 | 4.55403 | H | -22.4585 | 2.49897 | -0.05819 |
| H | -22.0869 | 6.9241 | -0.77087 | H | -24.1341 | -0.96128 | -4.32894 |
| H | -26.3169 | 3.69182 | -2.73609 | H | -28.1358 | -3.64152 | -1.27823 |
| H | -26.4623 | 7.05966 | -2.68109 | H | -28.8284 | -1.91661 | -4.09136 |
| H | -28.9312 | 5.23723 | -1.27308 | H | -30.4991 | -1.2376 | -1.23701 |
| H | -28.3575 | 7.57639 | 2.81222 | H | -29.278 | 3.33338 | -1.59731 |
| H | -25.4051 | 7.55756 | 4.42466 | H | -26.0456 | 4.26591 | -1.66303 |
| H | -25.776 | 9.38653 | 1.61319 | H | -27.4306 | 2.80614 | -4.37047 |
| H | -25.7758 | 1.63687 | 1.28087 | H | -26.2478 | -1.28837 | 2.11049 |
| H | -17.555 | 6.0637 | -0.35633 | H | -17.0261 | -3.26332 | -1.94981 |
| H | -15.3761 | 5.1826 | 2.01518 | H | -19.5621 | -2.73704 | -4.07311 |
| H | -14.5284 | 1.04817 | -0.2087 | H | -14.8587 | 0.62103 | -3.25017 |
| H | -17.4546 | -2.39103 | 8.32796 | H | -16.1793 | 1.19223 | 6.92574 |
| H | -14.8288 | -2.83065 | 6.29179 | H | -13.8944 | -0.55978 | 5.19129 |
| H | -15.6256 | 1.47955 | 9.71474 | H | -15.835 | 4.67948 | 4.12204 |
| H | -9.5069 | 4.34429 | 7.49143 | H | -13.3725 | 4.91628 | -1.26729 |
| H | -12.5751 | 5.48765 | 6.67991 | H | -12.9389 | 7.1897 | 1.1775 |
| H | -11.8859 | 4.59641 | 9.86061 | H | -10.2648 | 5.64535 | -0.1851 |
| H | -11.3848 | -2.42397 | 8.45196 | H | -8.77108 | 4.13595 | 4.06002 |
| H | -11.231 | -0.13482 | 10.9281 | H | -10.8059 | 2.37769 | 6.08537 |
| H | -8.84047 | -0.22338 | 8.55885 | H | -11.3663 | 5.6665 | 5.58012 |
| H | -10.2015 | 1.15725 | 4.26318 | H | -12.3761 | 0.598 | -0.16923 |
| H | -13.2845 | 1.16458 | -6.40833 | H | -13.5237 | 3.08915 | -6.36427 |
| H | -13.8817 | -0.98088 | -3.91055 | H | -14.7765 | 2.63172 | -9.44027 |
| H | -17.7679 | 6.19392 | -4.02349 | H | -20.5752 | -0.88354 | -7.28562 |
| H | -16.3881 | 4.98067 | -6.84069 | H | -20.6503 | 2.37337 | -8.08739 |
| H | -19.246 | 3.68808 | -5.68501 | H | -18.8898 | 0.25783 | -9.97278 |
| H | -18.388 | -1.48133 | -5.691 | H | -18.047 | 5.91535 | -8.65593 |
| H | -17.4732 | 0.38209 | -8.27784 | H | -16.9892 | 6.28374 | -5.51051 |
| H | -13.6345 | -3.56778 | -7.94969 | H | -13.8924 | 7.50127 | -10.3501 |
| H | -16.0947 | -7.24706 | -12.7661 | H | -9.61903 | 11.269 | -7.28329 |
| H | -13.7032 | -7.23816 | -10.3815 | H | -11.1013 | 10.8299 | -10.2846 |
| H | -16.561 | -8.98553 | -9.95579 | H | -12.0657 | 13.3305 | -8.23132 |
| H | -20.7236 | -3.3436 | -8.71218 | H | -14.5253 | 12.2071 | -4.1312 |
| H | -20.1844 | -4.16584 | -11.9283 | H | -15.3158 | 9.00255 | -3.47055 |
| H | -20.7461 | -6.56889 | -9.67904 | H | -12.1352 | 10.0747 | -3.20254 |
| **4-9** | | | | **4-10** | | | |
| Atom | X | Y | Z | Atom | X | Y | Z |
| C | -20.8152 | -0.13738 | 0.73752 | C | -22.3251 | -1.71482 | 0.89611 |
| C | -20.9186 | -2.52402 | 1.67622 | C | -21.5783 | -3.70287 | 2.33646 |
| C | -19.0245 | -3.32296 | 3.58987 | C | -18.9962 | -3.68315 | 3.44629 |
| C | -17.3556 | -1.34882 | 4.49244 | C | -17.5778 | -1.36795 | 3.09664 |
| C | -17.3714 | 1.02252 | 3.54233 | C | -18.4364 | 0.59666 | 1.70087 |
| C | -18.8309 | 1.81096 | 1.2848 | C | -20.7394 | 0.50125 | 0.1054 |
| C | -23.2608 | -4.0503 | 1.28416 | C | -23.5203 | -5.52421 | 3.26816 |
| O | -25.3403 | -3.08129 | 1.69518 | O | -25.5136 | -4.71696 | 4.16672 |
| C | -23.0668 | -6.70435 | 0.41076 | C | -23.0421 | -8.28029 | 3.10044 |
| C | -20.8204 | -7.64712 | -0.64528 | C | -21.1432 | -9.25565 | 1.52143 |
| C | -20.7145 | -10.1275 | -1.5291 | C | -20.7948 | -11.8605 | 1.32652 |
| C | -22.8404 | -11.6878 | -1.34575 | C | -22.3226 | -13.5064 | 2.72141 |
| C | -25.0851 | -10.7612 | -0.2935 | C | -24.2177 | -12.5473 | 4.29998 |
| C | -25.2024 | -8.27775 | 0.56925 | C | -24.5855 | -9.9472 | 4.47841 |
| C | -20.5146 | 4.1861 | 1.50283 | C | -22.7025 | 2.63341 | 0.443 |
| C | -22.8347 | 3.56015 | -0.15221 | C | -25.2448 | 1.2984 | -0.07273 |
| O | -22.5965 | 0.86813 | -0.73736 | O | -24.667 | -1.40683 | 0.01611 |
| C | -25.4246 | 3.85101 | 1.16635 | C | -27.306 | 1.71899 | 1.95535 |
| C | -27.5469 | 3.10327 | -0.65283 | C | -29.7014 | 0.25878 | 1.24632 |
| C | -25.7732 | 6.55984 | 2.08463 | C | -27.8698 | 4.53067 | 2.23822 |
| O | -25.4697 | 2.25291 | 3.34115 | O | -26.361 | 0.84608 | 4.32802 |
| C | -16.8682 | 2.00308 | -0.95212 | C | -19.9829 | 0.09 | -2.74177 |
| C | -17.9864 | 2.59755 | -3.48165 | C | -18.3048 | 2.1083 | -3.79474 |
| C | -15.3391 | -1.5267 | 6.48467 | C | -15.0642 | -0.65996 | 4.21982 |
| C | -14.2283 | 1.18447 | 6.48281 | C | -14.855 | 2.1652 | 3.48358 |
| O | -15.7094 | 2.60636 | 4.59479 | O | -16.9741 | 2.65045 | 1.7405 |
| C | -11.4274 | 1.37674 | 5.71154 | C | -12.3834 | 2.97258 | 2.21128 |
| C | -10.6222 | 4.14569 | 5.4932 | C | -12.565 | 5.70015 | 1.26762 |
| C | -9.78882 | -0.00904 | 7.63652 | C | -10.2136 | 2.68137 | 4.10274 |
| O | -11.0736 | 0.12064 | 3.34309 | O | -12.0307 | 1.29317 | 0.12063 |
| O | -18.9849 | -5.48802 | 4.48444 | O | -18.1859 | -5.45009 | 4.75439 |
| C | -17.9205 | 4.82759 | -4.70623 | C | -16.0003 | 1.76894 | -4.81669 |
| C | -19.1415 | 5.07422 | -7.27589 | C | -14.5286 | 3.99465 | -5.82679 |
| C | -16.6707 | 7.17862 | -3.71142 | C | -14.7329 | -0.7723 | -5.0759 |
| C | -17.2442 | 5.41107 | -9.4771 | C | -13.9754 | 3.8554 | -8.68765 |
| C | -15.34 | 3.30296 | -9.63336 | C | -12.397 | 6.03918 | -9.57983 |
| C | -15.6148 | 1.08919 | -10.8563 | C | -13.1167 | 8.00738 | -11.0226 |
| C | -13.5859 | -0.90559 | -10.78 | C | -11.2803 | 10.0729 | -11.7104 |
| C | -17.9294 | 0.38072 | -12.3527 | C | -15.7374 | 8.36234 | -12.0741 |
| H | -19.165 | -6.42758 | -0.80009 | H | -19.9593 | -7.97354 | 0.42374 |
| H | -18.9693 | -10.8493 | -2.36297 | H | -19.3272 | -12.6125 | 0.08451 |
| H | -22.7501 | -13.6352 | -2.02784 | H | -22.0381 | -15.547 | 2.5764 |
| H | -26.7426 | -11.9842 | -0.15071 | H | -25.4082 | -13.8363 | 5.38835 |
| H | -26.9404 | -7.52687 | 1.38794 | H | -26.0614 | -9.17174 | 5.69299 |
| H | -19.5428 | 5.89563 | 0.85346 | H | -22.3895 | 4.22845 | -0.84105 |
| H | -21.0865 | 4.44197 | 3.4794 | H | -22.6387 | 3.3206 | 2.39866 |
| H | -22.8197 | 4.57443 | -1.95896 | H | -25.9924 | 1.70075 | -1.9657 |
| H | -27.3062 | 1.14332 | -1.30273 | H | -29.2976 | -1.77075 | 1.04451 |
| H | -27.5836 | 4.34435 | -2.32239 | H | -30.5008 | 0.94705 | -0.547 |
| H | -29.3823 | 3.24872 | 0.31554 | H | -31.131 | 0.49456 | 2.73885 |
| H | -27.6378 | 6.75714 | 2.98567 | H | -28.4969 | 5.34557 | 0.43046 |
| H | -24.3271 | 7.07998 | 3.48433 | H | -29.3828 | 4.80293 | 3.63983 |
| H | -25.666 | 7.90043 | 0.49829 | H | -26.1976 | 5.58082 | 2.88839 |
| H | -25.5074 | 0.49008 | 2.7791 | H | -26.188 | -0.9948 | 4.25825 |
| H | -15.4929 | 3.44183 | -0.36421 | H | -19.0989 | -1.77996 | -2.89358 |
| H | -15.866 | 0.18022 | -1.03012 | H | -21.7643 | 0.00382 | -3.82028 |
| H | -18.9767 | 1.03364 | -4.40712 | H | -19.0536 | 4.0378 | -3.71835 |
| H | -16.0914 | -2.04983 | 8.35206 | H | -14.9956 | -0.91774 | 6.28078 |
| H | -13.9041 | -2.94042 | 5.96715 | H | -13.5206 | -1.78114 | 3.3882 |
| H | -14.523 | 2.15885 | 8.29195 | H | -15.1867 | 3.40162 | 5.11986 |
| H | -11.7651 | 5.14121 | 4.06949 | H | -14.1061 | 5.90088 | -0.10866 |
| H | -10.844 | 5.1369 | 7.30886 | H | -12.9072 | 7.01698 | 2.84181 |
| H | -8.62471 | 4.2524 | 4.92341 | H | -10.7808 | 6.24855 | 0.34585 |
| H | -7.79088 | 0.12605 | 7.07409 | H | -8.42055 | 3.23241 | 3.19949 |
| H | -10.2884 | -2.02345 | 7.75321 | H | -10.0343 | 0.71263 | 4.74709 |
| H | -9.99832 | 0.8321 | 9.52624 | H | -10.4963 | 3.89876 | 5.76568 |
| H | -11.9412 | 1.08949 | 2.05136 | H | -10.4378 | 1.70938 | -0.68155 |
| H | -20.297 | 3.38474 | -7.65488 | H | -15.5482 | 5.76486 | -5.42429 |
| H | -20.4338 | 6.71525 | -7.2651 | H | -12.7109 | 4.09671 | -4.80151 |
| H | -15.2304 | 7.87774 | -5.04735 | H | -15.3606 | -2.13712 | -3.64228 |
| H | -18.0711 | 8.71235 | -3.51278 | H | -15.1096 | -1.61643 | -6.94693 |
| H | -15.7511 | 6.91056 | -1.87159 | H | -12.6676 | -0.58406 | -4.90877 |
| H | -18.3377 | 5.56276 | -11.242 | H | -12.9558 | 2.08068 | -9.09103 |
| H | -16.247 | 7.22617 | -9.25398 | H | -15.7796 | 3.74922 | -9.71711 |
| H | -13.5911 | 3.58138 | -8.55629 | H | -10.4338 | 6.02439 | -8.90531 |
| H | -11.9506 | -0.31719 | -9.63683 | H | -11.9599 | 11.9311 | -11.0421 |
| H | -14.3245 | -2.69118 | -9.98764 | H | -9.39082 | 9.74477 | -10.9045 |
| H | -12.9087 | -1.36221 | -12.7022 | H | -11.0837 | 10.2406 | -13.7829 |
| H | -17.4081 | -0.22622 | -14.2805 | H | -16.5458 | 10.1714 | -11.4167 |
| H | -18.8937 | -1.24923 | -11.473 | H | -15.686 | 8.47604 | -14.1583 |
| H | -19.311 | 1.9219 | -12.511 | H | -17.0539 | 6.84721 | -11.5454 |
| **4-11** | | | | **4-12** | | | |
| Atom | X | Y | Z | Atom | X | Y | Z |
| C | -20.5563 | 1.20199 | 1.05239 | C | -20.4481 | 0.09262 | 0.58025 |
| C | -20.7233 | -1.34273 | 0.75685 | C | -20.3497 | -2.33143 | 1.40241 |
| C | -19.3911 | -3.00936 | 2.58512 | C | -18.5974 | -2.99924 | 3.48646 |
| C | -18.1 | -1.73934 | 4.63922 | C | -17.3007 | -0.88726 | 4.65433 |
| C | -18.0249 | 0.80229 | 4.87916 | C | -17.4845 | 1.50766 | 3.77975 |
| C | -19.0097 | 2.60234 | 2.97546 | C | -18.7617 | 2.21844 | 1.38925 |
| C | -22.6354 | -2.403 | -1.02148 | C | -22.3971 | -4.10368 | 0.60378 |
| O | -24.798 | -1.53302 | -1.0555 | O | -24.6208 | -3.46608 | 0.86924 |
| C | -21.9136 | -4.50769 | -2.72848 | C | -21.7229 | -6.55537 | -0.56724 |
| C | -23.8246 | -5.92907 | -3.90693 | C | -23.644 | -8.3162 | -1.0842 |
| C | -23.1974 | -7.91185 | -5.51987 | C | -23.0746 | -10.5786 | -2.30131 |
| C | -20.655 | -8.47217 | -5.99639 | C | -20.5811 | -11.0981 | -3.02235 |
| C | -18.7444 | -7.03927 | -4.8657 | C | -18.6609 | -9.35294 | -2.51979 |
| C | -19.3676 | -5.06836 | -3.23192 | C | -19.2262 | -7.08845 | -1.29576 |
| C | -21.0262 | 4.53381 | 3.82361 | C | -20.6959 | 4.40549 | 1.44267 |
| C | -22.5188 | 5.01638 | 1.36255 | C | -22.7457 | 3.58546 | -0.4649 |
| O | -21.887 | 2.87641 | -0.28248 | O | -22.1731 | 0.94828 | -1.04619 |
| C | -25.4247 | 4.94839 | 1.63429 | C | -25.4841 | 3.57172 | 0.55049 |
| C | -26.6643 | 5.39517 | -0.94152 | C | -27.2972 | 2.68182 | -1.52257 |
| C | -26.292 | 6.91976 | 3.54827 | C | -26.221 | 6.19448 | 1.48694 |
| O | -26.1396 | 2.52958 | 2.59254 | O | -25.6085 | 1.89653 | 2.66499 |
| C | -16.7312 | 3.97337 | 1.63106 | C | -16.5994 | 2.65286 | -0.62387 |
| C | -15.605 | 2.40969 | -0.44226 | C | -17.5294 | 2.75722 | -3.29177 |
| C | -16.7652 | -2.91281 | 6.85496 | C | -15.4944 | -0.92522 | 6.84637 |
| C | -15.6954 | -0.55597 | 8.22533 | C | -14.6832 | 1.88485 | 7.02916 |
| O | -16.7948 | 1.64288 | 6.92187 | O | -16.1298 | 3.21776 | 5.05082 |
| C | -12.7844 | -0.28575 | 8.22324 | C | -11.8603 | 2.39295 | 6.5282 |
| C | -11.6808 | -0.24695 | 5.55456 | C | -11.3372 | 5.23614 | 6.44831 |
| C | -12.0343 | 2.08275 | 9.68518 | C | -10.2629 | 1.12453 | 8.56565 |
| O | -11.8054 | -2.37719 | 9.62798 | O | -11.1646 | 1.25893 | 4.17406 |
| O | -19.5044 | -5.34516 | 2.46807 | O | -18.3796 | -5.19566 | 4.27523 |
| C | -15.7287 | 2.91796 | -2.9324 | C | -17.7147 | 4.78797 | -4.80791 |
| C | -14.7708 | 1.02827 | -4.8461 | C | -18.8258 | 4.60658 | -7.44092 |
| C | -16.8973 | 5.2793 | -4.01817 | C | -16.9119 | 7.4152 | -4.07032 |
| C | -16.8067 | 0.23553 | -6.76199 | C | -20.2941 | 2.20057 | -8.1258 |
| C | -15.9479 | -1.85577 | -8.49383 | C | -18.7075 | -0.13254 | -8.48938 |
| C | -17.4434 | -3.29312 | -9.96449 | C | -19.3615 | -2.51133 | -7.87165 |
| C | -16.3813 | -5.36088 | -11.6026 | C | -17.6387 | -4.71241 | -8.40121 |
| C | -20.2646 | -2.96738 | -10.1147 | C | -21.8562 | -3.15132 | -6.62519 |
| H | -25.7977 | -5.47224 | -3.51618 | H | -25.5801 | -7.87747 | -0.52437 |
| H | -24.6874 | -9.02854 | -6.41183 | H | -24.5713 | -11.9448 | -2.69661 |
| H | -20.1632 | -10.0276 | -7.26239 | H | -20.1356 | -12.8712 | -3.98327 |
| H | -16.7636 | -7.45578 | -5.26355 | H | -16.7194 | -9.75617 | -3.09242 |
| H | -17.8738 | -3.94553 | -2.36367 | H | -17.7322 | -5.71449 | -0.94027 |
| H | -20.1893 | 6.26684 | 4.5936 | H | -19.8383 | 6.21815 | 0.92183 |
| H | -22.2609 | 3.67067 | 5.25015 | H | -21.5024 | 4.56044 | 3.34626 |
| H | -21.9132 | 6.74142 | 0.38189 | H | -22.6338 | 4.63668 | -2.24674 |
| H | -28.7317 | 5.26436 | -0.75443 | H | -26.7984 | 0.77201 | -2.17533 |
| H | -26.0406 | 3.96624 | -2.31696 | H | -27.2558 | 3.96269 | -3.16157 |
| H | -26.1866 | 7.276 | -1.69141 | H | -29.2387 | 2.62752 | -0.77762 |
| H | -25.5159 | 6.53673 | 5.43867 | H | -26.0583 | 7.59719 | -0.04005 |
| H | -25.7008 | 8.82699 | 2.96697 | H | -28.1937 | 6.17142 | 2.14681 |
| H | -28.3673 | 6.89125 | 3.68899 | H | -25.0238 | 6.80717 | 3.07243 |
| H | -25.7586 | 1.24909 | 1.31196 | H | -25.3849 | 0.16498 | 2.05918 |
| H | -17.4233 | 5.78512 | 0.90582 | H | -15.6128 | 4.38261 | -0.03985 |
| H | -15.3441 | 4.40722 | 3.12057 | H | -15.2668 | 1.06659 | -0.41566 |
| H | -14.7478 | 0.61852 | 0.14649 | H | -18.1571 | 0.94172 | -4.03978 |
[truncated: 52,971 more chars]
